# Tracing the international arrivals of SARS-CoV-2 Omicron variants after Aotearoa New Zealand reopened its border

**DOI:** 10.1038/s41467-022-34186-9

**Published:** 2022-10-29

**Authors:** Jordan Douglas, David Winter, Andrea McNeill, Sam Carr, Michael Bunce, Nigel French, James Hadfield, Joep de Ligt, David Welch, Jemma L. Geoghegan

**Affiliations:** 1 Centre for Computational Evolution,School of Computer Science, University of Auckland, Auckland, New Zealand; 2 Institute of Environmental Science and Research, Wellington, New Zealand; 3 Tāwharau Ora/School of Veterinary Science, Massey University, Palmerston North, New Zealand; 4 Te Niwha, Infectious Diseases Research Platform, Institute of Environmental Science and Research, Palmerston North, New Zealand; 5 Fred Hutchinson Cancer Research Centre, Seattle, WA USA; 6 Department of Microbiology and Immunology, University of Otago, Dunedin, New Zealand

**Keywords:** Phylogenomics, Epidemiology, SARS-CoV-2

## Abstract

In the second quarter of 2022, there was a global surge of emergent SARS-CoV-2 lineages that had a distinct growth advantage over then-dominant Omicron BA.1 and BA.2 lineages. By generating 10,403 Omicron genomes, we show that Aotearoa New Zealand observed an influx of these immune-evasive variants (BA.2.12.1, BA.4, and BA.5) through the border. This is explained by the return to significant levels of international travel following the border’s reopening in March 2022. We estimate one Omicron transmission event from the border to the community for every ~5,000 passenger arrivals at the current levels of travel and restriction. Although most of these introductions did not instigate any detected onward transmission, a small minority triggered large outbreaks. Genomic surveillance at the border provides a lens on the rate at which new variants might gain a foothold and trigger new waves of infection.

## Abstract (web summary)

In March 2022, Aotearoa New Zealand re-opened its border allowing quarantine-free travel for many travellers. Here, the authors describe circulating Omicron sub-variants before and after the reopening of the border and show that the rate of viral introductions grew roughly linearly with the increase in daily international travel.

## Introduction

At the beginning of the coronavirus disease 2019 (COVID-19) pandemic, Aotearoa, New Zealand, closed its borders in order to quell the addition of further outbreaks in the community^1,2^ (March 2020). These border control measures greatly limited arrivals and required those who were able to enter to spend at least 14 days at a dedicated managed isolation and quarantine (MIQ) facility upon arrival^3^. Due to its geographical isolation, the New Zealand border was able to be tightly regulated. Coupled with a stringent local response (including stay-at-home orders, contact tracing, and isolation of cases^4^), this strategy resulted in the elimination of COVID-19 in New Zealand by May 2020^5,6^. This elimination phase, which lasted until late 2021, saw several small but quickly contained outbreaks, which leaked from MIQ facilities, cargo vessels, and other channels through the border^3^. Between May 2020 and July 2021, the country recorded a total of only 1390 cases and five deaths. Real-time genomic surveillance played a pivotal role in sustaining this state of elimination^3,7^.

**Fig. 1:**
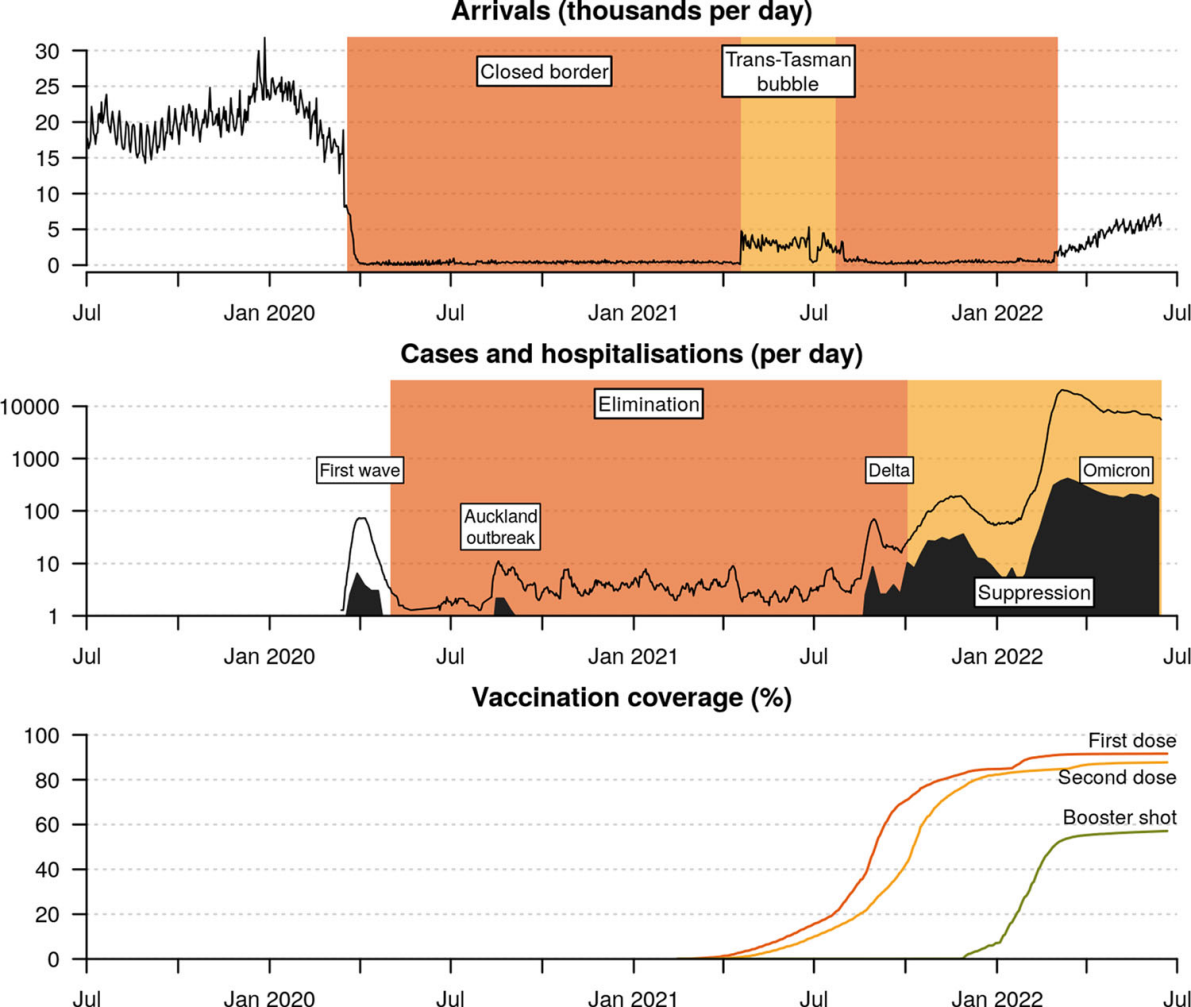
Timeline of the New Zealand COVID-19 pandemic. Top: International arrivals per day. *Middle:* Daily cases (black line) are averaged across a 1-week period, and include cases in both MIQ (managed isolation and quarantine) facilities as well as the community, while daily hospitalisations (filled curve) are averaged across weekly reports and describe cases hospitalised due to COVID-19. Bottom: Vaccination coverage is expressed as a percentage of the eligible community (5+ years of age; or 94% of the total population). The genomic epidemiology of the first three waves have been characterised elsewhere - first wave;^1^ Auckland August outbreak^7^; and, Delta wave^10^.

The border restrictions remained until the trans-Tasman travel ‘bubble’ opened in April 2021, enabling quarantine-free travel between New Zealand and Australia (Fig. 1), which at the time was also pursuing an elimination strategy^8,9^. However, the travel bubble was suspended in July 2021 due to Australia’s difficulty in controlling the emergent Delta variant of concern (VoC). Shortly afterwards, the Delta variant entered the New Zealand community; it likely leaked from a MIQ facility via a traveller from Australia^10^. Unlike previous variants, Delta spread widely and quickly and was unable to be fully controlled, thus leading New Zealand (following a nationwide vaccine rollout, Fig. 1) to abandon its elimination strategy in favour of suppression by early October 2021^11^. By early 2022, the even-more infectious Omicron VoC (BA.1 and BA.2) had entered the community and quickly outcompeted Delta as it had done globally^12–14^. Border controls were gradually relaxed and the MIQ system was abandoned in favour of pre-departure and on-arrival testing. In the first half of 2022, New Zealand recorded ~1.2 million COVID-19 cases.

Unlike other severe acute respiratory syndrome coronavirus 2 (SARS-CoV-2) variants, Omicron includes multiple subvariants, termed BA.1–BA.5. Omicron variants are characterised by at least 50 nonsynonymous mutations compared with ancestral genomes, with a large proportion of these concentrated in the receptor binding domain of the spike protein, resulting in Omicron having an effective reproduction number ~3–4 times that of Delta^15^.

Shortly after BA.2 triggered additional waves across the globe, three further Omicron lineages—BA.2.12.1, BA.4, and BA.5—were linked to another rise in cases globally^16,17^. Both BA.4 and BA.5, which only differ from one another outside of the spike protein, possess spike mutations L452R and F486V, offering both increased binding affinity and enhanced immune escape with an estimated growth advantage of 0.08 and 0.14 per day over BA.2, respectively^18^. The ability to seemingly evade vaccine- and infection-induced immunity provides BA.4 and BA.5 with this growth advantage. Although their severity in humans remains unclear, infection experiments on hamsters suggest that BA.4 and BA.5 may spread more efficiently through lung cells and may be more pathogenic than BA.2^19^. The apparent continued genomic diversification of Omicron lineages highlights the need to tightly monitor its evolution and dispersal.

From March 2022, MIQ ceased for fully vaccinated New Zealand citizens, residents, and work visa holders. Persons arriving in New Zealand were instead required to undertake COVID-19 rapid antigen tests on days 0–1 and 5–6 of arrival, without the need to quarantine. New arrivals who tested positive were required to self-isolate for 7 days (previously 10–14) and undergo a nasopharyngeal swab and PCR test, which could also be sent for whole-genome sequencing. Household contacts were also required to self-isolate. Because of these more relaxed border settings, the rate of international arrivals rose from fewer than 500 to over 5000 per day between March and June 2022 (Fig. 1). Similarly, community cases were required to self-isolate for 7 days after testing positive (or developing symptoms; whichever came first) as were their household contacts. Other close contacts were encouraged to monitor their symptoms but they were not required to quarantine or be tested.

The move from elimination to suppression and then reopening of the borders was precipitated by the hard-to-control Delta outbreak. It also balanced the economic and social burden of ongoing restrictions against health outcomes in a population with high levels of primary, secondary, and booster vaccination^11,20^. By late 2021, over 80% of the eligible population (5 years+) had received two doses of the Pfizer-BioNTech (BNT162b2) vaccine. By 15 Jun 2022, the figure was 88% with two doses and 57% had received at least one booster, albeit with some significant waning of immunity given the plateau since early 2022 (Fig. 1). These figures were comparable to Australia, with 84% fully vaccinated and 54% boosted^21^.

Reopening the border increases the risk of emergent COVID-19 variants more rapidly entering the community. Each novel introduction of COVID-19 comes with the risk of triggering an outbreak. On 31 July 2022, New Zealand fully opened to vaccinated tourists and travellers from anywhere in the world, without the need for pre-departure testing or on-arrival isolation. Arrivals have returned to about 60% of previous levels at 10,000–11,000 arrivals per day and may eventually return to pre-pandemic levels of 15,000-30,000 daily arrivals. In this study, we evaluate the impact that recent changes to the border are having on New Zealand’s ability to control COVID-19. Specifically, we monitor the arrival of Omicron variants (BA.1, BA.2, BA.2.12.1, BA.4 and BA.5) from overseas into the New Zealand community. This article was written from the perspective of 1 August 2022 and analyses New Zealand genomic data up until 15 June 2022.

## Results

### Omicron genomics and sampling

Diagnostic labs sent positive nasopharyngeal samples to undergo genomic sequencing, where cases that were linked to the border or admitted to the hospital were sequenced with high priority. According to the New Zealand Ministry of Health, who provided the epidemiological metadata used here, border cases are defined as those who tested positive in MIQ, or within seven days of arriving in New Zealand, while community cases are all other New Zealand cases. Due to limitations on referral and sequencing capacity, many nasopharyngeal samples were not referred for sequencing, and some that were could not be processed. Geographic diversity was prioritised for sample inclusion. Diagnostic labs were requested to refer all border and hospital samples for sequencing, but due to disruptions in case classification and data sources, no systematic subsampling was possible. As such, a retrospective analysis of what was sampled is the most appropriate way of describing the sampling process, and is detailed below.

**Fig. 2:**
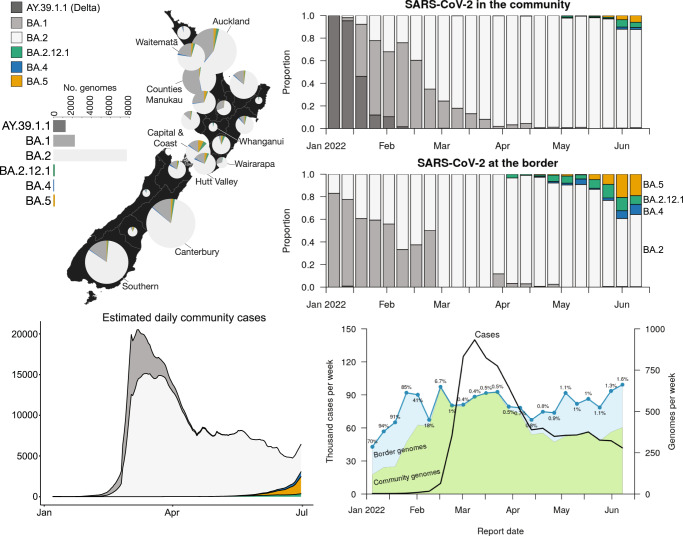
Summary of Omicron genomic sequencing. Top left: Omicron variant distribution by New Zealand district health board, for cases reported between 8 Dec 2021 and 15 June 2022. The Delta lineage (AY.39.1.1) is omitted from the map. Top right: New Zealand genome sequencing, coloured by lineage. A border case is one either in managed isolation after arriving in New Zealand (MIQ (managed isolation and quarantine) era), or one with overseas travel history in the past seven days (post-MIQ era). BA.2.12.1 is not included in BA.2 metrics. Bottom left: Estimated number of community cases of each variant. Bottom right: Number and percentage of cases which were referred to genomic sequencing each week. Almost 100% of the genomes were labelled as ‘community’ between Feb-Apr due to missing epidemiological data.

We generated 10,403 high-quality SARS-CoV-2 genomes, designated as the Omicron VoC, sampled between 8 Dec 2021 and 15 Jun 2022 from both border cases and community cases (Fig. 2). Lineages were designated as BA.1 (1565 from the community and 708 from the border), BA.2 (5951 and 1735), BA.2.12.1 (50 and 125), BA.4 (15 and 66) and BA.5 (47 and 141). Using a multinomial model, we estimated that around 13% of the total cases in this period were BA.1, 85% BA.2, 0.52% BA.2.12.1, 0.25% BA.2, and 0.49% BA.5. Here, and throughout the remainder of this article, when we report BA.2 lineages, we are excluding BA.2.12.1 unless specified otherwise. On average, 536 genomes were produced each week since the start of the year, and weekly numbers remained quite consistent throughout the Omicron outbreak (Fig. 2; bottom right panel). In the weeks prior to this outbreak, the majority of cases were referred to sequencing. However, this dropped to under 0.5% during the peak of the Omicron wave in March 2022 (Fig. 2).

This sample represents 0.8% of the 1,247,900 reported cases in this period nationwide. However, the proportion sequenced varies between regions: the Whanganui district health board saw the smallest proportion of its cases referred for genomic sequencing, at 0.35%, while the Southern district saw the most, at 1.8% (Fig. S1). But despite this disparity across regions, the national average of variant proportions was congruent with those attained through regional wastewater samples. On the week ending 12 Jun 2022, wastewater samples estimated 97% as BA.2 (inclusive of BA.2.12.1), and 3% as BA.4/5 combined^22^. In this same week, 94% of the whole-genome sequencing samples were BA.2/BA.2.12.1 and 6% were BA.4/5.

Hospitalised and border cases were oversampled. Hospitalised persons with COVID-19 made up just 0.6% of the case count, but 11% of the genome count (between 16 May and 15 Jun; complete epidemiological data are unavailable before this period). Similarly, border cases made up just 0.7% of the case count, but 25% of the genome count (between 1 Jan and 15 Jun). However, during the peak of the Omicron outbreak, the sequencing of border genomes declined greatly (Fig. 2; bottom right panel). This decline is explained by missing data, as travel history was largely omitted from the New Zealand Ministry of Health’s epidemiological data collection process during this period, meaning some border cases were incorrectly classified as community cases.

By February 2022, Omicron BA.1 and BA.2 had outcompeted the prevailing Delta VoC (B.1.617.2; lineage AY.39.1.1) in the community, and BA.2 subsequently outcompeted BA.1. As of June 15 2022, BA.2.12.1, BA.4 and BA.5 were on the rise in New Zealand, while BA.2 continued to dominate (Fig. 2). The data presented here were too early in their outbreaks to make reliable growth advantage estimates, however using public genomic data extended up until 31 July 2022 (https://github.com/ESR-NZ/nz-sars-cov2-variants), we estimated the growth advantage per day over BA.2 in New Zealand as 0.08 for BA.4 and 0.10 for BA.5. These are comparable to estimates made in South Africa (0.08 and 0.14 respectively^18^).

### Counting omicron introductions using phylodynamics

We describe a framework for tracing SARS-CoV-2 introductions from overseas into the community. Here, a ‘*global’* case is defined as one who tested positive either overseas, during their managed isolation period after arriving in New Zealand, or within seven days of arriving in New Zealand (after the MIQ system was abolished). A *‘community’* case is one based in New Zealand, and without any recent (i.e. within the past 7 days) overseas travel history. The New Zealand Ministry of Health has annotated all of its cases with such labelling. Thus, we define an *‘introduction’* (or an *‘arrival’*) as a transmission event from the *global* pool to the *community* pool. The large sample of both border and overseas genomes should facilitate the detection of introduction events into the community.

**Fig. 3:**
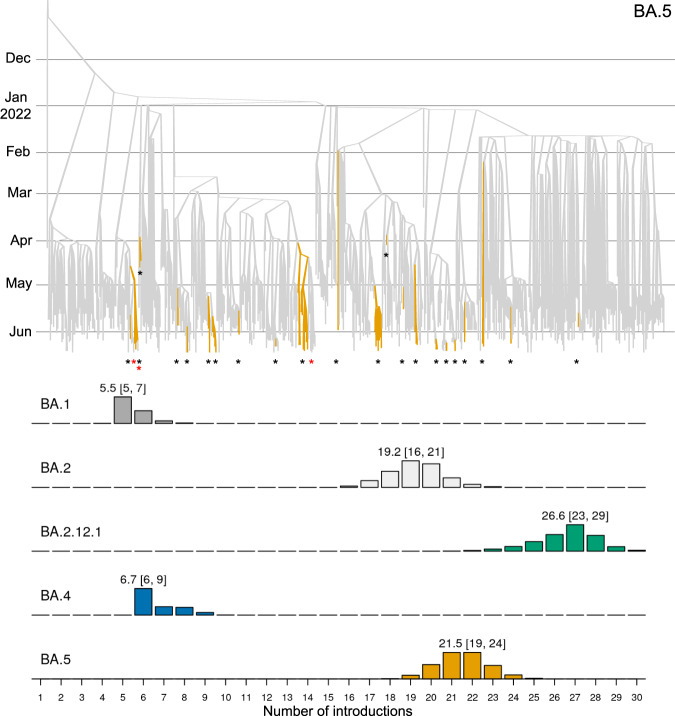
Discrete phylogeographical analysis of Omicron in New Zealand. Top: summary tree of the BA.5 analysis. Lineages are coloured by world (grey) or community (orange). Introductions from the world into the community are indicated by black stars *, while export events from the community to the border (i.e. new arrivals who acquired their infection from the New Zealand community) are indicated by red stars *. Similar trees for other variants can be found in Supporting Information. Bottom: posterior distribution of introduction counts (across all trees). The y-axes are proportional to Bayesian posterior support. The means [and 95% credible intervals] are indicated.

**Fig. 4:**
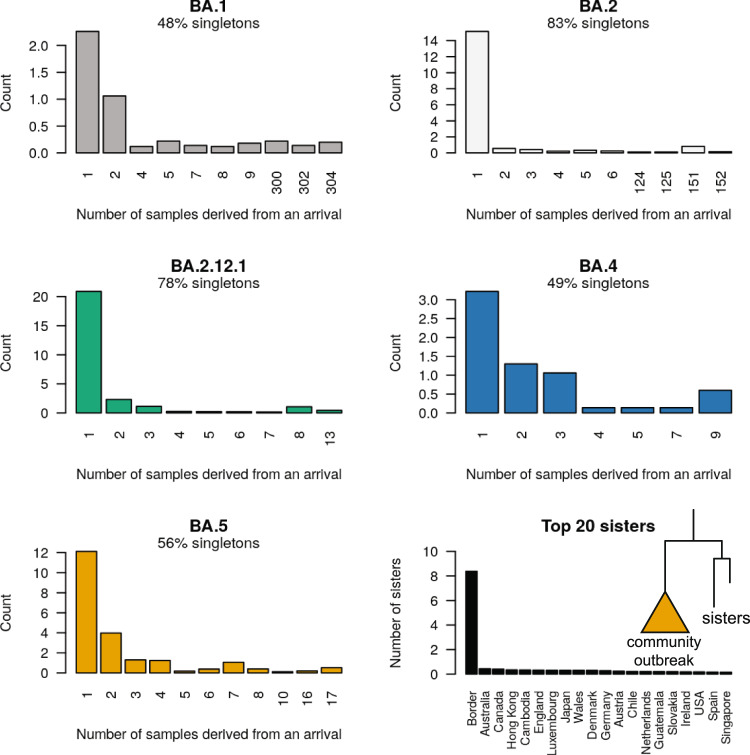
Posterior distribution of sample count (i.e. tree leaf count; or clade size) resulting from each introduction event. Many of these introductions were singletons and did not lead to any detected secondary infection. Bottom right: the number of times each region was in the sister clade to a community outbreak Sister genomes are down-weighted by their clade size (see Methods).

We estimated the number of SARS-CoV-2 introductions into the New Zealand community using both global and local genomic sequences (as described in Methods). These results show that BA.1 and BA.4 were only introduced a few times, while BA.2, BA.2.12.1, and BA.5 were introduced significantly more frequently (Fig. 3). The majority of these introductions were singletons and did not lead to any secondary infections in the community. BA.1 and BA.2 were each associated with 1 or 2 large outbreaks represented by over 100 genomic samples, while the outbreaks detected among the younger variants have thus far been smaller as they are much more recent (Fig. 4). The majority (62%) of these introductions were linked to the New Zealand border cases, as opposed to overseas cases (Fig. 4; bottom right panel), reflecting likely routes of entry and our oversampling protocol for border cases.

Based on our phylogenetic results, while the majority (75% across all five analyses) of transitions were in the direction of introductions to New Zealand, there were several instances of New Zealand border cases which appear to have been acquired within the New Zealand community (depicted by red stars in the tree at the top of Fig. 3). Most of these 'export' events were BA.2 (Fig. S3), which tallies with infection after arrival in New Zealand by what has been the predominant local variant. There were no export events from the community overseas in any of our summary trees.

### Tracing Omicron arrivals from the border

**Fig. 5:**
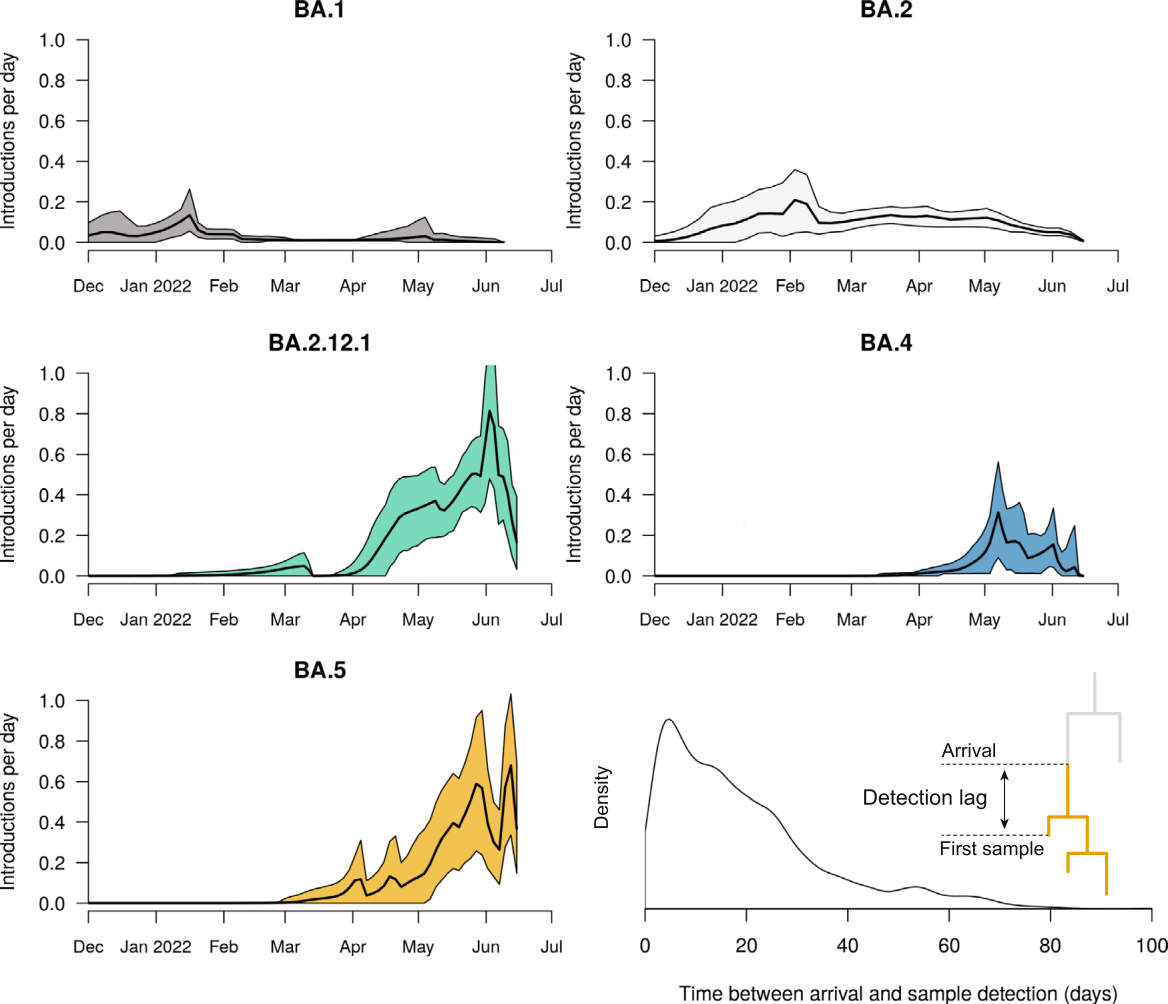
Estimated arrival rate of Omicron subvariants into New Zealand. Mean estimates (black line) and 95% credible intervals (shaded) are indicated. Bottom right: Lag time between when a lineage is estimated to have entered the community (i.e. an introduction), and when the first case is detected (i.e. sampling). The sample date is when the case tested positive, and not the date of genomic sequencing. The meaning of lag time is illustrated in the figure.

The first quarter of 2022 was characterised by several BA.1 and BA.2 introductions (Fig. 5), some of which spread widely through the New Zealand population. This is consistent with epidemiological metadata, which linked many cases during this period to a small number of superspreader events. These introductions coincided with the period of missing travel data in early 2022 (Fig. 2; bottom right panel). Thus, the number of introductions here may be an overestimate, with many border cases having been misclassified as community cases during this period.

In contrast, the second quarter was characterised by BA.2.12.1, BA.4 and BA.5 introductions. Notably, we estimated 19-24 introductions of BA.5, and 23-29 for BA.2.12.1, into the community since they first arrived in April 2022. These are higher than the 16-21 BA.2 introductions despite ongoing BA.2 introductions since late 2021. In our sample, there was an average detection lag of 19 days (95% credible interval of 0.3−55 days), between an estimated introduction time and the lineage being detected via testing (Fig. 5). This is similar to the 14-day detection lag reported by^23^, and likely something of an overestimate due to our global sampling protocol—a more comprehensive global genome sample would reduce this lag.

**Fig. 6:**
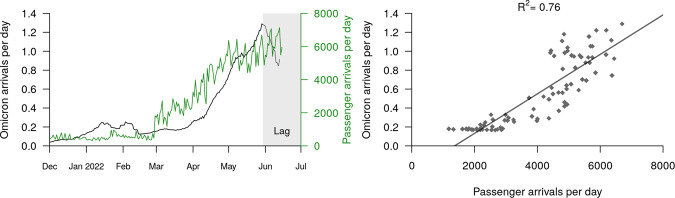
Estimated rate of Omicron arrival into the community. Left: Estimated Omicron arrivals (into the community) and recorded passenger arrivals (into the country). The black curve is equal to a smoothed sum of the five black curves in Fig. 5. The Omicron arrival rate appears to drop off at the start of June, but this is simply due to a lag between lineages arriving at the border and then being detected in the community by genomic surveillance. Right: Omicron arrivals can be explained by passenger arrivals into New Zealand. This linear model was built from the two curves in the left panel, restricted from after the border opened (3 Mar) until available genomic data starts to lag (30 May).

The most recent surge of Omicron introductions into the community (namely BA.2.12.1 and BA.5) can be largely explained by the relaxation of New Zealand’s border restrictions, including an end to the MIQ system in early March 2022. We compared the estimated rate of Omicron arrivals with the recorded number of border crossings into New Zealand (i.e. passenger arrivals). These results show a strong correlation between the daily arrival rate of passengers into the country and the estimated daily arrival rate of Omicron into the community (Fig. 6). We fit a linear regression model to these data and identified a strong positive linear relationship between the two (*R*^2^ = 0.76). The slope coefficient was 0.000209, indicating that, under the currently enforced border control measures, there is ~1 Omicron arrival per 5000 passenger arrivals, or around two per day at levels of travel seen at the start of August.

## Discussion

Since the start of the COVID-19 pandemic, highly transmissible and immune-evasive SARS-CoV-2 variants have emerged worldwide^24,25^. By the start of August, BA.5 had risen to be the successor of BA.2 as the globally dominant lineage^12^. There is also a significant proportion of global cases that are of the BA.2.12.1 and BA.4 lineages.

In this study, we focused on the introduction of emergent variants (BA.2.12.1, BA.4 and BA.5) into New Zealand. Travel restrictions were greatly relaxed in March 2022 and this reopening led to the international traveller arrival rate increasing by orders of magnitude since the start of the year, and will likely increase further following the border’s full reopening to tourists on 31 July 2022 (Fig. 1) and a return of capacity in the tourism sector. In order to evaluate lineages entering the community, we described a framework for using genomic data to identify the trajectories of variants from overseas, through the New Zealand border pre-departure and on-arrival testing requirements, and into the community.

We distinguish between New Zealand cases with recent overseas travel (i.e. the border), compared to those without (i.e. the community) and can therefore trace introductions directly into the population. Border cases were sequenced with higher priority than community cases, facilitating the detection of novel introductions into the community - indeed, the majority of introduction events were derived from border cases (Fig. 4; bottom right panel). This protocol yielded an estimated average detection lag of 19 days between lineages being introduced into the community and then being detected, but this estimate could be further improved by subsampling global genomes that are more closely related to community cases, or by including even more locally acquired genomes in the analysis. This framework is based on real-time genomic surveillance coupled with Bayesian phylogenetic inference. Recent computational advancements - such as the BICEPS, ORC, and online packages for BEAST 2^26–28^—have made rapid Bayesian phylogenetic inference on large genomic datasets more feasible.

We showed that the first quarter of 2022 was characterised by the introduction, and widespread transmission, of Omicron BA.1 and BA.2 into the country (Fig. 2), while the second quarter was characterised by multiple introductions of BA.2.12.1, BA.4 and BA.5. We estimated at least six (for BA.1) and 27 (BA.2.12.1) introductions of each variant (Fig. 3). The preponderance of recent introductions were of the BA.2.12.1 and BA.5 variants, reflecting trends in overseas ‘feeder’ countries. This may also reflect their higher transmissibility and ability to evade immunity. Community introductions of Omicron variants surged after the New Zealand borders reopened in March 2022, and grew roughly linearly with the daily international arrival rate. Under the current border settings where arrivals are required to be vaccinated and self-test on arrival, we estimated there is approximately one transmission event into the community for every 5000 passenger arrivals into the country (Fig. 6). Epidemiological models from earlier in the year predicted that a second wave was likely to arise in August or September 2022 due to the nation’s waning population immunity, but they noted that a variant with a growth advantage could bring that wave forward^29,30^. It turned out that BA.4 and BA.5 were the new variants that caused the wave, with case data showing a peak of the second wave, dominated by BA.5, occured in mid-July with cases now declining.

Congruent with previous phylodynamic studies worldwide, we found that while some introductions into the country triggered widespread outbreaks, around half of the introductions did not instigate any detectable onward transmission at all (Fig. 4)^2,23,31–37^. This speaks to the highly stochastic nature of disease transmission^38^ and emphasises how a greater rate of international travel, and therefore a greater rate of viral importation (Fig. 6), leads to a higher chance of a large community outbreak being triggered. Among these introductions were three large outbreaks with over 100 samples, associated with at least two superspreader events: a wedding for BA.1 (Fig. S2) and a music festival for BA.2 (Fig. S3). However, despite the prevalence of Omicron in New Zealand, we did not detect any infections originating from the community to the rest of the world (only from the community to the border). This contrasts with a recent study in Brazil which estimated around one export event to the rest of the world for every 10 introductions^36^, as well as studies performed in Colombia^14^, Jordan^33^, Rwanda^37^, Belarus^39^ and Europe^40^. This discrepancy is perhaps due to the small population size of New Zealand at a global scale (five million people), and comparatively low global sequencing rates.

Although the existing literature on COVID-19 phylodynamics is vast^41^, we believe this study is among the first to directly link temporal viral introduction rates to international traveller arrival rates. This link is intuitive and likely to generalise to other parts of the world, but is more readily established in nations where travel across the border is highly regulated^33^. Prolonged genomic surveillance throughout the border reopening has placed Aotearoa, New Zealand, in an excellent position to study this system.

The analyses performed here come with their limitations. First, due to the overwhelming availability of both global genomic data on the GISAID as well as local New Zealand data produced here, subsampling was necessary. Our methodologies are only as powerful as their subsampling strategies, which are in turn only as powerful as the underlying processes by which infections are detected and then sequenced by COVID-19 surveillance programmes, both locally and globally. Second, the pool of genomic sequences is not a representative sample of the global pandemic due to the wide disparity in real-time genomic sequencing outputs across different parts of the world^7,42^. Finally, the reliability of the epidemiological annotations of New Zealand cases into community and border is contingent on the New Zealand Ministry of Health’s internal protocols, which are beyond our control and have varied in quality during different stages of the pandemic. Still, with these caveats noted, we believe our results are robust and are generally consistent with previous studies.

Overall, we have demonstrated how pathogen surveillance at the border can measure the effectiveness of border control measures and provide advance warning of potential outbreaks. This approach is not restricted to COVID-19—it can also be applied to seasonal influenza virus, respiratory syncytial virus, or the ongoing global monkeypox outbreak^43^, for example. As new pathogens continue to emerge around the world, monitoring their global transmission and tracing their arrival into unexposed communities remain important tasks for genomic surveillance.

## Method

### Genomic sequencing and epidemiology

For cases reported between 8 December 2021 and 15 June 2022, ~0.8% of all COVID-19 cases were referred to the Institute of Environmental Science and Research, New Zealand. In brief, viral extracts were prepared from respiratory tract samples in which SARS-CoV-2 was detected by rRT-PCR. Extracted RNA was subjected to whole-genome sequencing using the Oxford Nanopore Technologies R9.4 chemistry by following the Midnight protocol v6^44^, which contains a 1200-bp primer set tiling the SARS-CoV-2 genome. Consensus genomes were generated through a standardised pipeline (https://github.com/ESR-NZ/NZ_SARS-CoV-2_genomics) based on the original ARTIC bioinformatics pipeline (https://artic.network/ncov-2019/ncov2019-bioinformatics-sop.html; v1.2.1). Genomes were designated into lineages using Pangolin version 4.0.6^45^. Here we report high-quality genomes which have less than 10% ambiguous characters. Recombinant Omicron genomes - XE (*n* = 1), XAG (*n* = 1) and XAC (*n* = 4)—were detected at low frequencies but were not included in this study.

We estimated the number of community cases belonging to each variant (Fig. 2; bottom left panel) using a multinomial model. In this model (*nnet* package^46^), the variant associated with each sequenced case was treated as a response variable and the report date and district health board of that case were set as predictors. We used the fitted model to predict the proportions of each variant for a given district health board and date, and then multiplied these values by the corresponding reported case numbers to estimate the total number of cases for each variant. Growth advantages per day of BA.4 and BA.5, relative to BA.2, were estimated using a multinomial logistic regression model, as described by^18^, from public data that is the 7-day rolling average of variant counts.

We use two distinct definitions of COVID-19 hospitalisations throughout this article. These definitions and data were provided by the New Zealand Ministry of Health. First, hospitalisations *for* COVID-19 (as shown in the middle panel of Fig. 1) are determined by evaluating clinical codes entered in the National Minimum Dataset (NMDS) for hospitalisations nationwide and excluding hospitalisations that are highly unlikely to be related to COVID-19. The NMDS provides a robust estimate of hospitalisations for COVID-19, however, there is often a delay before data are finalised. This delay can vary but can be approximately 60 days or more. Second, hospital admissions *with* COVID-19 (as detailed in Omicron genomics and sampling) included individuals who tested positive for COVID-19 in the seven days prior to admission or whilst in hospital; excluding hospitalisations that were admitted and discharged within 24 h, and those where admitted was highly unlikely to be related to COVID-19 infection. This dataset includes data from districts with tertiary hospitals, the districts are Auckland, Canterbury, Southern, Counties Manukau, Waikato, Capital & Coast, Waitemata and Northland. It provides a preliminary assessment of hospitalisations and is subject to revision as more comprehensive and more accurate data become available. Data on hospital admissions with COVID-19 are provided to the Institute of Environmental Science and Research in an identifiable form daily.

### Inferring SARS-CoV-2 introductions

We infer introductions from genomic data as follows:

1. Retrieve high-quality SARS-CoV-2 genomes from around the world (e.g. from the GISAID EpiCov database^47^). These genomes should be from the respective lineage and during an appropriate time frame (in our case 1 January–15 June 2022).
2. Sample, without replacement, *N*_*1*_ high-quality SARS-CoV-2 genomes from this global pool. In order to reduce geographical sampling bias, genomes are sampled uniformly across locations (e.g. England is equally likely to be sampled as Hong Kong, provided that genomes are available for either location). The genomic sampling disparity between different parts of the world is vast^42^, and this protocol should relieve some of this effect. New Zealand genomes are omitted from this sample. These genomes are added to the *global* pool.
3. Sample, without replacement, *N*_*2*_ high-quality SARS-CoV-2 genomes from the available New Zealand genomes. In order to reduce population sampling bias, genomes are sampled linearly through time (e.g. 5 January 2022 is equally likely to be sampled as 5 May 2022, assuming that at least one genome was sampled on either date). The *x* genomes, which are labelled as *border* cases, are added to the *global* pool, and the remaining *N*_*2*_*-x* are added to the *community* pool. This labelling was provided as epidemiological case metadata by the New Zealand Ministry of Health.
4. Generate a multiple sequence alignment from the two sampled pools (here, we used NextAlign^48^ with Wuhan-Hu-1 (NC_045512.2) as a reference).
5. Run a Bayesian two deme discrete phylogeographic analysis on the alignment (described in the next subsection).
6. Omicron introductions are estimated as transitions from the *global* deme to the community deme in the inferred phylogenetic trees.
7. The time of an introduction of a clade of community cases is approximated as the mid-point of the branch immediately above the most recent common ancestor of the clade.

We applied this procedure for each of BA.1, BA.2, BA.2.12.1, BA.4 and BA.5, where *N*_1_ = 400 and *N*_2_ = 400. It is important that the *community* pool is not significantly larger than the *global* pool, else the discrete phylogeography model can become unreliable^2,49^. We have used a similar procedure for inferring introductions into New Zealand in previous work^2^, as have others for Brazil^36^, Rwanda^37^ and Europe^40^, and has been reviewed elsewhere^41^.

### Phylogenetic analysis

Bayesian phylogenetic inference was performed using BEAST 2.6^50^. We modelled transitions between the two demes (*global* and *community*) using a discrete phylogeography (DPG) model^51^. Under this model, the geographic transition rate had a LogNormal(−0.738, 0.3) prior distribution, the relative transition rate from *global* to the *community* was sampled from LogNormal(4.29, 0.8), while the reverse rate was fixed at 1. This prior assumption means that imports into the community are expected to be significantly more frequent than exports back to the *global* deme, and is used to prevent back-and-forth transitions from appearing too often in the tree. The root of the phylogenetic tree is assumed to belong to the *global* deme. We used an efficient implementation of the Bayesian skyline tree prior implemented in the BICEPS package^26^, where the first effective population size is drawn from a Gamma(rate = *b*, shape = 2) distribution, where *b* ~ LogNormal(−2.43, 0.5). Nucleotide substitution was modelled using an HKY model^52^ with frequencies estimated from a Dirichlet(1,1,1,1) distribution, and a transition-transversion ratio drawn from a LogNormal(1, 1.25) prior. The molecular substitution rate was estimated from a LogNormal(−6.9, 0.05) prior. We used adaptive-weight operators from the ORC package^27^ and adaptive variance multivariate normal distribution operators^53^ to improve convergence during Bayesian Markov chain Monte Carlo (MCMC). Two independent MCMC chains were run under each lineage, and their convergences were diagnosed using Tracer^54^ Each analysis had over 200 effective samples for all relevant parameters. Our BEAST 2 XML file template is uploaded as Supplementary Data 1. Phylogenetic tree posterior distributions were summarised as the maximum clade credibility tree^55^ and visualised using UglyTrees^56^. Our sister clade analysis (Fig. 4) was performed by counting the proportion of each region in a clade next to a community outbreak, and then summing these proportions across all outbreaks in all 5 posterior distributions (one posterior for each variant) to attain the mean number of times each region occurred as a sister genome per tree. Detection lag (Fig. 5) was calculated as the time between the introduction and the first sample, where the introduction time is described above.

### Ethics statement

Nasopharyngeal samples that had positive results for SARS-CoV-2 by real-time reverse transcription PCR were obtained from medical diagnostic laboratories located throughout New Zealand. Under contract for the New Zealand Ministry of Health, the Institute of Environmental Science and Research has the approval to conduct genomic sequencing and phylogenetic analysis for surveillance of notifiable diseases.

### Reporting summary

Further information on research design is available in the Nature Research Reporting Summary linked to this article.

## Supplementary information

Supplementary Information

Description of Additional Supplementary Files

Supplementary Data 1

Supplementary Data 2

Supplementary Data 3

Supplementary Data 4

Supplementary Data 5

Supplementary Data 6

Reporting Summary

## Data Availability

All global SARS-CoV-2 genomic sequence data were available on GISAID (see Supplementary Data 2–6). Newly produced Omicron genomes from New Zealand are available on GenBank: accessions OP631676–OP641806, OP719781–OP720051, and OP729239. Case, death, hospitalisation and vaccination data used in the Introduction were taken from a New Zealand Ministry of Health GitHub repository (https://github.com/minhealthnz/nz-covid-data; accessed 30 June 2022). New Zealand passenger arrival data were taken from the Statistics New Zealand International travel provisional records (https://www.stats.govt.nz/indicators/international-travel-provisional; accessed 30 June 2022).

## Code availability

We used open-source software: BEAST v2.6.7, PANGO v4.0.6, NextAlign v.2.3.0 and the latest version of the ARTIC bioinformatics pipeline (https://github.com/artic-network/artic-ncov2019; accessed 30 June 2022). We used the BICEPS v1.0.1, ORC v.1.0.3 and BEASTLabs v1.9.7 packages for BEAST 2. Our BEAST 2 XML file template (with sequence data removed) is uploaded as Supplementary Data 1.
