# Supplementary material for: Tracing the international arrivals of SARS-CoV-2 Omicron variants after Aotearoa New Zealand reopened its border
Source: Nat Commun. 2022 Oct 29;13:6484. doi: 10.1038/s41467-022-34186-9 (PMC9617600; doi:10.1038/s41467-022-34186-9)
Supplement: Supplementary file 1 — Supplementary Information [file 41467_2022_34186_MOESM1_ESM.pdf]

Supporting Information: Tracing the international arrivals of SARS-CoV-2 Omicron variants after Aotearoa New Zealand reopened its border

Jordan Douglas[1,*], David Winter[2], Andrea McNeill[2], Sam Carr[2], Michael Bunce[2], Nigel French[3,4], James Hadfield[5], Joep de Ligt[2], David Welch[1], Jemma L Geoghegan[2,6]

[1]Centre for Computational Evolution,School of Computer Science, University of Auckland, Auckland, New Zealand.

[2]Institute of Environmental Science and Research, Wellington, New Zealand.

[3]Tāwharau Ora/School of Veterinary Science, Massey University, Palmerston North, New Zealand.

[4]Te Niwha, Infectious Diseases Research Platform, Institute of Environmental Science and Research.

[5]Fred Hutchinson Cancer Research Centre, Seattle, Washington, USA.

[6]Department of Microbiology and Immunology, University of Otago, Dunedin, New Zealand.

*To whom correspondence should be addressed: jordan.douglas@auckland.ac.nz

Figure S1 details the genomic sampling bias across the district health boards (DHB) of New Zealand. **Figures S2-S5** present the phylogenies of Omicron variants BA.1, BA.2, BA.2.12.1, and BA.4.

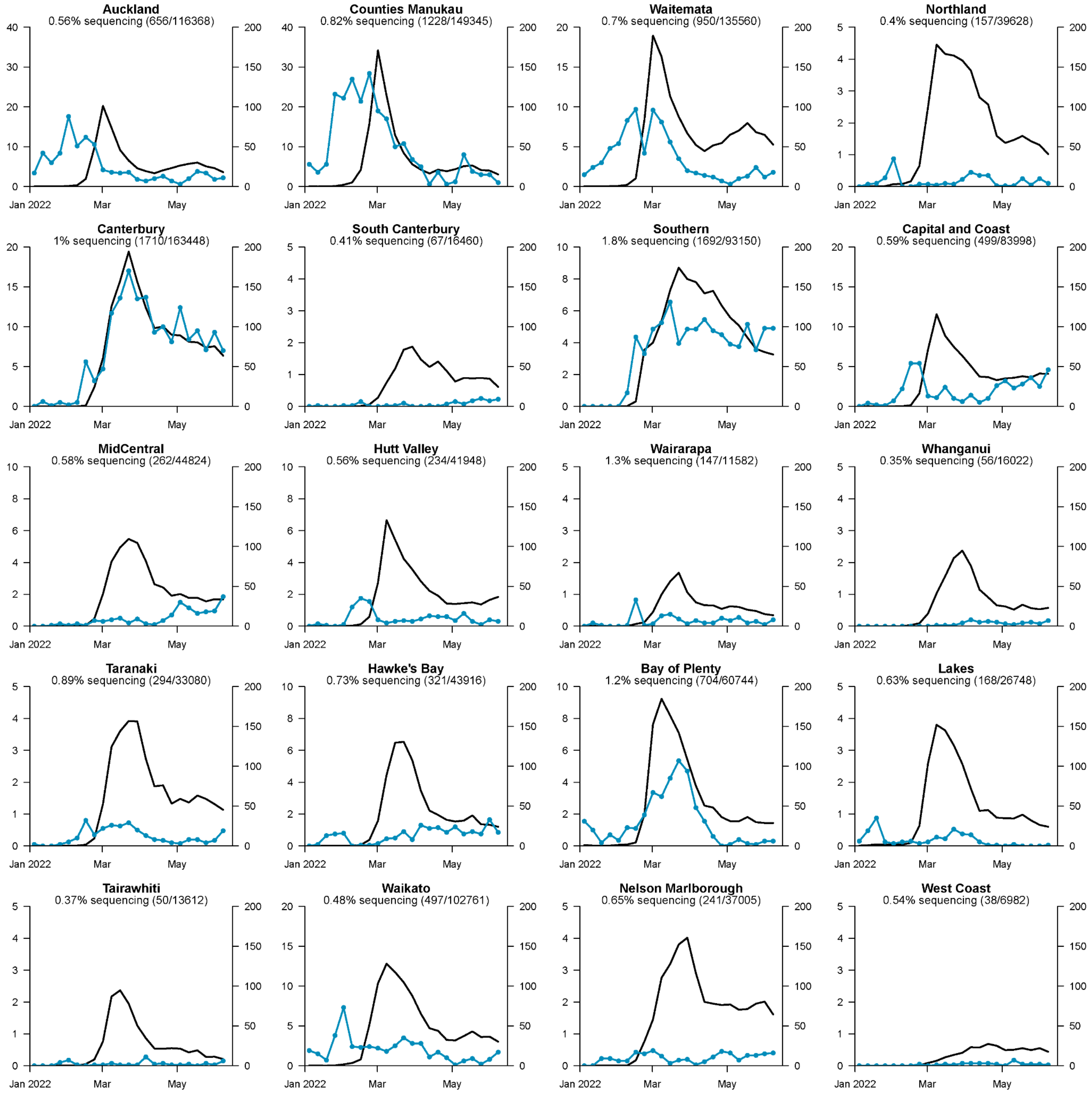

Figure S1. Thousands of cases (black curve; left axis) and number of genomes (dotted blue curve; right axis) by district health board. Genomic surveillance was especially proactive at Canterbury and the Auckland-based district health boards (Auckland, Counties Manukau, and Waitemata) due to their proximity to major international airports which saw a great amount of travel activity during the border reopening period.

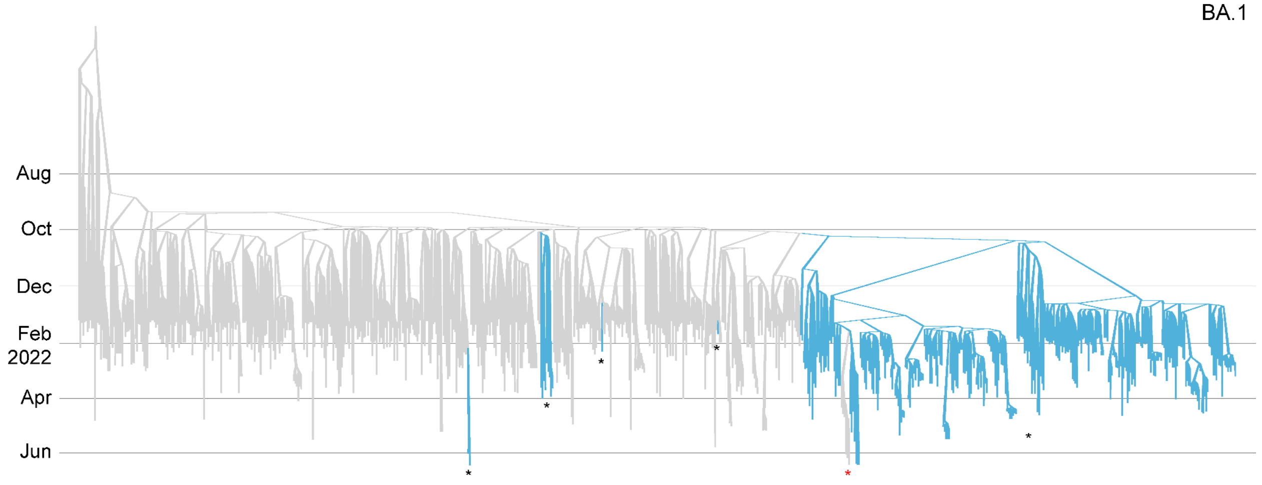

Figure S2. Summary tree of the BA.1 analysis. Lineages are coloured by world (grey) or community (blue). Introduction events from the world to the community are indicated by black stars *, while export events from the community to the border are indicated by red stars *. There are no export events from the community to the overseas pool in the summary tree.

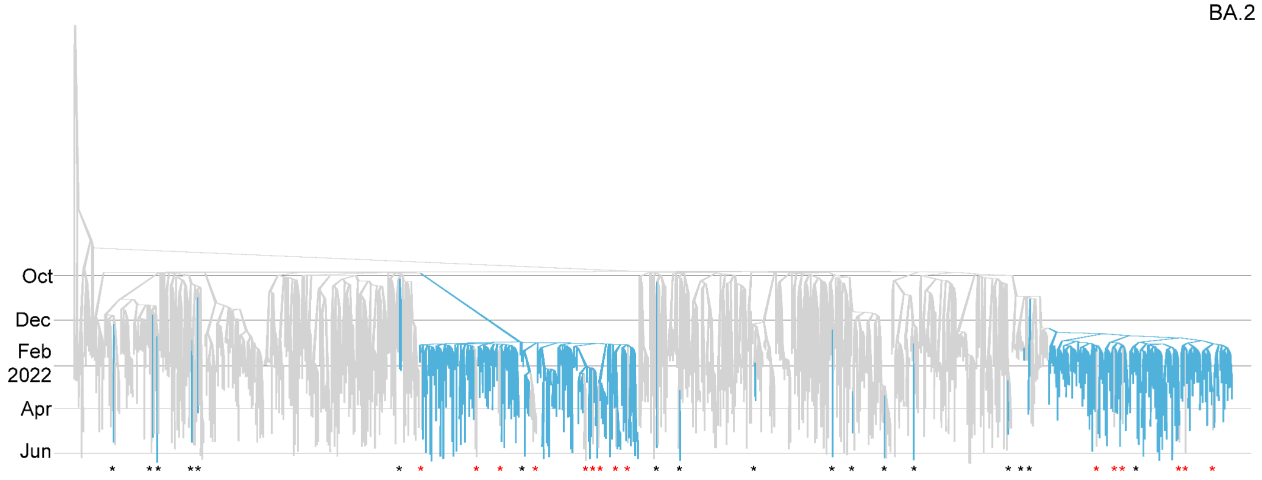

Figure S3. Summary tree of the BA.2 analysis. Lineages are coloured by world (grey) or community (blue). Introduction events from the world to the community are indicated by black stars *, while export events from the community to the border are indicated by red stars *. There are no export events from the community to the overseas pool in the summary tree.

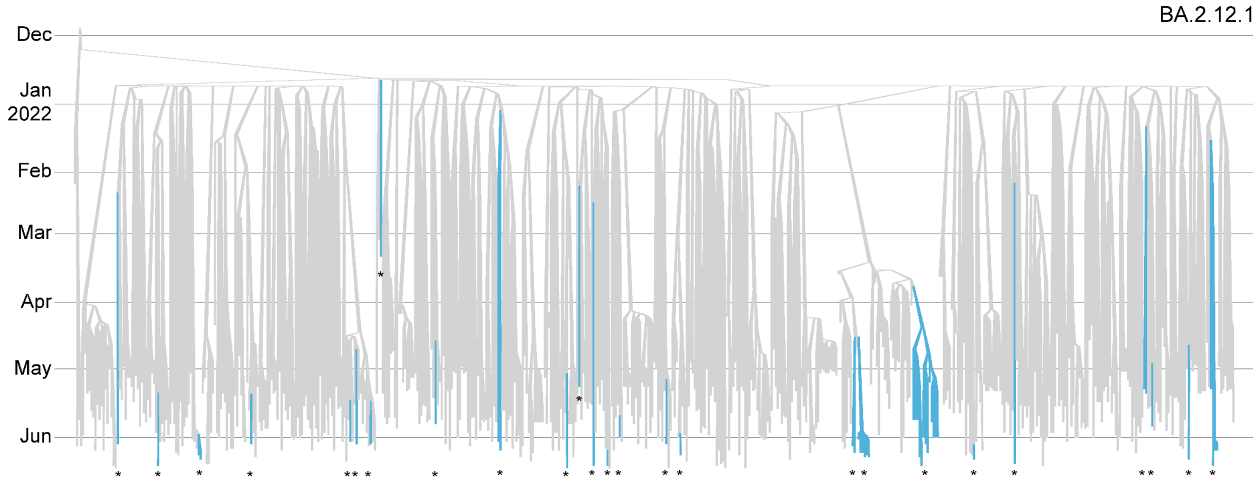

Figure S4. Summary tree of the BA.2.12.1 analysis. Lineages are coloured by world (grey) or community (blue). Introduction events from the world to the community are indicated by black stars *. There are no export events from the community to the global pool in the summary tree.

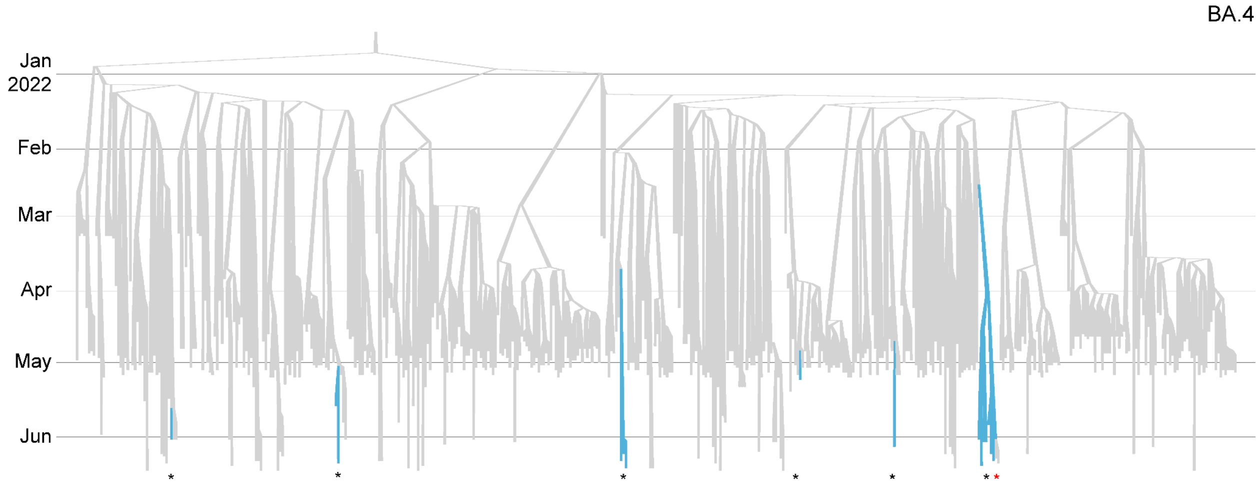

Figure S5. Summary tree of the BA.4 analysis. Lineages are coloured by world (grey) or community (blue). Tree heights are in units for years, where time 0 is 15 Jun 2022. Introduction events from the world to the community are indicated by black stars *, while export events from the community to the border are indicated by red stars *.
