# Supplementary material for: Tracing the international arrivals of SARS-CoV-2 Omicron variants after Aotearoa New Zealand reopened its border
Source: Nat Commun. 2022 Oct 29;13:6484. doi: 10.1038/s41467-022-34186-9 (PMC9617600; doi:10.1038/s41467-022-34186-9)
Supplement: Supplementary file 2 — Description of Additional Supplementary Files [file 41467_2022_34186_MOESM2_ESM.pdf]

**Description of Additional Supplementary Files**

File Name: Supplementary Data 1

Description: BEAST 2 XML file template (with sequence data removed)

File Name: Supplementary Data 2

Description: GISAID Acknowledgements for Omicron BA.1 genomes

File Name: Supplementary Data 3

Description: GISAID Acknowledgements for Omicron BA.2.12.1 genomes

File Name: Supplementary Data 4

Description: GISAID Acknowledgements for Omicron BA.2 genomes

File Name: Supplementary Data 5

Description: GISAID Acknowledgements for Omicron BA.4 genomes

File Name: Supplementary Data 6

Description: GISAID Acknowledgements for Omicron BA.5 genomes
