# Supplementary material for: Tracing the international arrivals of SARS-CoV-2 Omicron variants after Aotearoa New Zealand reopened its border
Source: Nat Commun. 2022 Oct 29;13:6484. doi: 10.1038/s41467-022-34186-9 (PMC9617600; doi:10.1038/s41467-022-34186-9)
Supplement: Supplementary file 4 — Supplementary Data 2 [file 41467_2022_34186_MOESM4_ESM.pdf]

We gratefully acknowledge the following Authors from the Originating laboratories responsible for obtaining the specimens, as well as the Submitting laboratories where the genome data were generated and shared via GISAID, on which this research is based.

All Submitters of data may be contacted directly via www.gisaid.org

Authors are sorted alphabetically.

Acknowledgement EPI_SET Identifier: EPI_SET_20220706be

Accession ID	Originating Laboratory	Submitting Laboratory	Authors
EPI_ISL_11686030	ACT Pathology	Schwessinger Lab, Research School of Biology, College of Science	Abigail Graetz; Ashley Jones; Austin Bird; Bayantes Dagvadorj; Benjamin Schwessinger; Carl McCombe; Catalina Barragán Quintero; Craig Kennedy; Evie Hodgson; Gabrielle Smith; Karina Kennedy; Makenna Short; Rachel Leonard; Rene Riedelbauch; Rita Tam; Robyn Hall; Scott Ferguson
EPI_ISL_10864979	AZDelta	AZ Delta Medical Laboratories in Roeselare, Belgium	Dieter De Smet; Frederik Van Hoecke; Geert Martens; Patrick Descheemaeker; on behalf of AZ Delta COVID-19 Genomics core (member of Genomic surveillance of SARS-CoV-2 in Belgium network)
EPI_ISL_8886635	Addu Equitorial Hospital	Addu Equitorial Hospital	D. Fathmath Nazla Rafeeq; Dr. Ibrahim Afzal; Mr. Ibrahim Nishan Ahmed; Ms. Aishath Shuhudha; Ms. Aminath Shazleena Abdul Rahman; Ms. Soafy Mohamed
EPI_ISL_9510038	BBMP- West	inStem, NCBS-INSACOG	Aswin SaiNarain; Dasaradhi Palakodeti; Uma Ramakrishnan
EPI_ISL_9648072, EPI_ISL_9896672, EPI_ISL_11992199	Bayerisches Landesamt fuer Gesundheit und Lebensmittelsicherheit (LGL)	Bayerisches Landesamt fuer Gesundheit und Lebensmittelsicherheit (LGL)	Alexandra Dangel; Andreas Sing; Annika Sprenger; Carola Berger; Laura Weise; Lisa Falk; Nikolaus Ackermann; Sabrina Hepner
EPI_ISL_13331313	Bheri Hospital	Center for Molecular Dynamics Nepal	Bheri Hospital; Birat Nepal Medical Trust; Center for Molecular Dynamics Nepal; Epidemic Intelligence Team; Koshi Hospital; Liverpool School of Tropical Medicine; Nepal Health Research Council; OUCRU Nepal; Sukraraj Tropical & Infectious Disease Hospital; University of Cambridge
EPI_ISL_10511959	Bio Molecular Laboratory	National Institute of Health, Department of Medical Sciences, Ministry of Public Health, Thailand	Archawin Rojanawiwat; Natchaya Khiadsang; Nuttida Thongpramul; Pakorn Piromtong; Pilailuk Okada; Sirikanda Wimol; Siripaporn Phuygun; Sunthareeya Waicharoen; Suratchana Mitrat; Thanutsapa Thanadachakul
EPI_ISL_12702789	BioneXt Lab	Laboratoire national de sante, Microbiology, Microbial Genomics Platform	Anke Wienecke-Baldacchino; Cynthia Oxacelay; Elodie Solarino; Eric Hugoson; Fatu Djabi; Jessica Tapp; Lise Pignon; Raoul Salmon; SibelCatherine Ragimbeau; Tamir Abdelrahman; Virginie Jover
EPI_ISL_11325766	Biotecnology and Genetics Laboratory - Instituto Nacional de Saúde	Biotecnology and Genetics Laboratory - Instituto Nacional de Saúde	Aventina Macuacua; Cacildo Magul; Mirela Pale; Nalia Ismael; Nuro Abilio; Samuel Chumane; Sofia Viegas
EPI_ISL_11905689	Bushehr University of Medical University	National Influenza Center	A Nejati; Adel Abedi; J Yavarian; K Sadeghi; Marzieh Faraji-Zonouz; NZ Shafiei Jandaghi; Nastaran Ghavami; Sevrin Zadheidar; V Salimi; and T Mokhtari Azad
EPI_ISL_9210610	CENTOGENE Frankfurt Laboratory: Niederlassung Industriepark Höchst	Robert Koch Institute	
EPI_ISL_9704077	CERBALLIANCE CENTRE VAL DE LOIRE	CERBA HealthCare	Bénédicte Roquebert; Laura Verdurme; Mathilde Roussel; Sabine Trombert; Stéphanie Haim-Boukobza
EPI_ISL_10314479	CERBALLIANCE COTE D'AZUR	CERBA HealthCare	Bénédicte Roquebert; Laura Verdurme; Mathilde Roussel; Sabine Trombert; Stéphanie Haim-Boukobza
EPI_ISL_9470063	CERBALLIANCE PARIS ET IDF EST	CERBA HealthCare	Bénédicte Roquebert; Laura Verdurme; Mathilde Roussel; Sabine Trombert; Stéphanie Haim-Boukobza
EPI_ISL_9300755	CHTMAD - Vila Real	Instituto Nacional de Saude (INSA)	Borges et al
EPI_ISL_9156927	Center for Laboratory Medicine	Center for Laboratory Medicine, ZLMSG	Yannick Gerth
EPI_ISL_10635619	Central Health Laboratory(Virology Dept) , Ministry of Health and Wellness	Central Health Laboratory(Virology Dept) , Ministry of Health and Wellness	Bahadoor BS; Janoo N; Mathur H; Ramuth M; Sonoo J; Sujeewon C; Ubheeram A
EPI_ISL_11895191, EPI_ISL_11895203	Central Public Health Laboratory - LACEN -Bahia, Salvador, Brazil	Central Public Health Laboratory - LACEN -Bahia, Salvador, Brazil	Arabela Leal; Felicidade Pereira; Gabriela Menezes; Jaqueline Gomes; Jessica Araujo; Luciana Oliveira; Luiz Alcantara; Marcela Gómez; Marta Giovanetti; Vagner Fonseca; Vanessa Nardy
EPI_ISL_11579838	Centre hospitalier de Châtellerault	Centre Hospitalier Universitaire (CHU) Poitiers	Agnes Beby-Defaux; Jean Philippe Da Mota; Luc Deroche; Magali Garcia; Manon Prat; Nicolas Leveque
EPI_ISL_9431841	Centrum Medyczne Medyk Sp. z o. o., Sp. k. Zakład Diagnostyki Medycznej	1. Academic Center for Pathomorphological and Genetic-Molecular Diagnostics ltd, Bialystok, Poland 2. National Institute of Public Health - National Institute of Hygiene, Warsaw, Poland	Anetta Sulewska; Jacek Nikliński; Janusz Dzięcioł; Joanna Kiśluk; Katarzyna Zacharczuk; Konrad Raczkowski; Magdalena Nowakowska; Małgorzata Sadkowska-Todys; Piotr Karabowicz; Piotr Majewski; Przemysław Biecek. Joanna Reszeć; Radosław Charkiewicz; Tomasz Wołkowicz
EPI_ISL_8931581, EPI_ISL_8931629	Cliniques universitaires Saint-Luc	UCLouvain/IREC/MBLG-CTMA	Benoit Kabamba Mukadi; Bertrand Bearzatto; Jean-Luc Gala; Nicolas Pinte; Paul Blanpain; Simon Ophélie; Valentin Coste
EPI_ISL_9227259	Department Medical Microbiology, Baerum Hospital, Vestre Viken Health Trust	Norwegian Institute of Public Health, Department of Virology	Atiya R Ali; Debech Nadia; Engebretsen Serina Beate; Garcia Llorente Ignacio; Hilde Elshaug; Hilde Nordby Falkenhaug; Hilde Vollan; Jon Bråte; Kamilla Heddeland Instefjord; Karoline Bragstad; Kathrine Stene-Johansen; Line Victoria Moen; Marie Paulsen Madsen; Olav Hungnes; Pedersen Benedikte Nevjen; Rasmus Riis Kopperud
EPI_ISL_8582273, EPI_ISL_8927075, EPI_ISL_9120233, EPI_ISL_9453635, EPI_ISL_9499975	Department of Bacteria, Parasites and Fungi, Statens Serum Institut, Copenhagen, Denmark	Statens Serum Institut Bioinformatics and Microbial Genomics	Danish Covid-19 Genome Consortium
EPI_ISL_10204396	Department of Clinical Microbiology and Center for Genomic Medicine, Rigshospitalet, Copenhagen, Denmark	Statens Serum Institut Bioinformatics and Microbial Genomics	Danish Covid-19 Genome Consortium
EPI_ISL_12418400	Department of Health Technology and Informatics, The Hong Kong Polytechnic University	Department of Health Technology and Informatics, The Hong Kong Polytechnic University	Alan Ka-Lun Wu; Alex Yat-Man Ho; Barry Kin-Chung Wong; Chloe Toi-Mei Chan; David Ho-Keung Shum; Gilman Kit-Hang Siu; Hiu-Yin Lao; Ivan Tak-Fai Wong; Jake Siu-Lun Leung; Kam-Tong Yip; Kenneth Siu-Sing Leung; Kingsley King-Gee Tam; Kitty Sau-Chun Fung; Kristine Luk; Lam-Kwong Lee; Miranda Chong-Yee Yau; Sandy Ka-Yee Chau; Shea Ping Yip; Tak-Lun Que; Timothy Ting-Leung Ng; Wing Cheong Yam; Wing-Hei Lo; Wing-Kin To; Yvette Wai-Man Lai
EPI_ISL_11539207, EPI_ISL_11539208, EPI_ISL_11725850, EPI_ISL_12005205	Department of Laboratory Medicine, Shinshu University Hospital	Department of Laboratory Medicine, Shinshu University Hospital	Akari Miyazaki; Saori Konno; Shohei Shigeto; Tatsuya Negishi; Wakaba Iha
EPI_ISL_12650021	Department of Medical Microbiology - section Molde, Molde Hospital	Norwegian Institute of Public Health, Department of Virology	Atiya R Ali; Debech Nadia; Engebretsen Serina Beate; Garcia Llorente Ignacio; Hilde Elshaug; Hilde Nordby Falkenhaug; Hilde Vollan; Jon Bråte; Kamilla Heddeland Instefjord; Karoline Bragstad; Kathrine Stene-Johansen; Line Victoria Moen; Marie Paulsen Madsen; Olav Hungnes; Pedersen Benedikte Nevjen; Rasmus Riis Kopperud
EPI_ISL_10844401, EPI_ISL_11055860, EPI_ISL_11260546, EPI_ISL_12009114	Department of Microbiology, Laboratoire Hospitalier Universitaire de Bruxelles, Université Libre de Bruxelles	Department of Microbiology, Laboratoire Hospitalier Universitaire de Bruxelles, Université Libre de Bruxelles	Charlotte Michel; Olivier Vandenberg; Sigi Van Den Wijngaert
EPI_ISL_11154406	Department of Public Health Bacau	National Institute of Infectious Diseases-Prof. Dr. Matei Bals Molecular Diagnostics Laboratory	Corina Casangiu; Dan Otelea; Leontina Banica; Marius Surleac; Ovidiu Vlaicu; Petre Milu; Robert Hohan; Simona Paraschiv
EPI_ISL_11154299	Department of Public Health Braila	National Institute of Infectious Diseases-Prof. Dr. Matei Bals Molecular Diagnostics Laboratory	Corina Casangiu; Dan Otelea; Leontina Banica; Marius Surleac; Ovidiu Vlaicu; Petre Milu; Robert Hohan; Simona Paraschiv
EPI_ISL_11154559	Department of Public Health Buzau	National Institute of Infectious Diseases-Prof. Dr. Matei Bals Molecular Diagnostics Laboratory	Corina Casangiu; Dan Otelea; Leontina Banica; Marius Surleac; Ovidiu Vlaicu; Petre Milu; Robert Hohan; Simona Paraschiv

EPI_ISL_11154317	Department of Public Health Constanta	National Institute of Infectious Diseases-Prof. Dr. Matei Bals Molecular Diagnostics Laboratory	Corina Casangiu; Dan Otelea; Leontina Banica; Marius Surleac; Ovidiu Vlaicu; Petre Milu; Robert Hohan; Simona Paraschiv
EPI_ISL_9967428, EPI_ISL_11154380, EPI_ISL_11154698, EPI_ISL_11154704	Department of Public Health Iasi	National Institute of Infectious Diseases-Prof. Dr. Matei Bals Molecular Diagnostics Laboratory	Corina Casangiu; Dan Otelea; Leontina Banica; Marius Surleac; Ovidiu Vlaicu; Petre Milu; Robert Hohan; Simona Paraschiv
EPI_ISL_9967316, EPI_ISL_9967343, EPI_ISL_9967468, EPI_ISL_9967469	Department of Public Health Mures	National Institute of Infectious Diseases-Prof. Dr. Matei Bals Molecular Diagnostics Laboratory	Corina Casangiu; Dan Otelea; Leontina Banica; Marius Surleac; Ovidiu Vlaicu; Petre Milu; Robert Hohan; Simona Paraschiv
EPI_ISL_9967533	Department of Public Health Timis	National Institute of Infectious Diseases-Prof. Dr. Matei Bals Molecular Diagnostics Laboratory	Corina Casangiu; Dan Otelea; Leontina Banica; Marius Surleac; Ovidiu Vlaicu; Petre Milu; Robert Hohan; Simona Paraschiv
EPI_ISL_8648923, EPI_ISL_9614456	Dept. of Microbiology and Infection Control, Akershus University Hospital HF	Dept. of Microbiology and Infection Control, Akershus University Hospital HF	Alexander Hesselberg Løvestad; Hanne Berggreen; Hege Vangstein Aamot
EPI_ISL_9658302, EPI_ISL_10230041	Diagnostyka. Laboratoria Medyczne.	1. ViroGenetics - BSL3 Laboratory of Virology, Małopolska Centre of Biotechnology, Jagiellonian University; 2. Diagtron Laboratoria Łukasz Rąbalski	Gromowski, T.; Kowalski, M.; Labaj; Maciej Kosinski; Mazur-Panasiuk, N.; Natalia Derewonko; P.P.; Pyrc, K.; Rabalski L.; Rogalska-Kupiec M.; Swadzba J.; Sylwia Januszczak; Szulc, P.; Wydmanski, W.
EPI_ISL_8998400	Division of Emerging Infectious Diseases, Bureau of Infectious Diseases Diagnosis Control, Korea Disease Control and Prevention Agency	Division of Emerging Infectious Diseases, Bureau of Infectious Diseases Diagnosis Control, Korea Disease Control and Prevention Agency	Ae Kyung Park; Chae Young Lee; Eun-Jin Kim; Hyuck Jin Lee; Il-Hwan Kim; Jeong-Ah Kim
EPI_ISL_8929209, EPI_ISL_10773177	Dutch COVID-19 response team	Medical Microbiology, Maastricht University Medical Centre	Brian van der Veer*; Carmen Reumkens; Christian Hoebe; Erik Beuken; Jozef Dingemans*; Lieke van Alphen; Paul Savelkoul
EPI_ISL_9085204, EPI_ISL_10036258, EPI_ISL_10036374, EPI_ISL_10036377, EPI_ISL_11659847, EPI_ISL_11840841	Dutch COVID-19 response team	National Institute for Public Health and the Environment (RIVM)	Adam Meijer; Afke Vogelzang; AnneMarie van den Brandt; Annelies Kroneman; Bas van der Veer; Chantal Reusken; Dennis Schmitz; Dirk Eggink; Florian Zwagemaker; Harry Vennema; Ivo van Walle; Jeroen Cremer; Jil Kocken; Jordy de Bakker; Karim Hajji; Kim Freriks; Linda van Someren; Lisa Wijsman; Lynn Aarts; Ryanne Jaarsma; Sanne Bos; Sharon van den Brink; on behalf of the national COVID-19 response team
EPI_ISL_9234082	GMERS Medical College, Valsad	Gujarat Biotechnology Research Centre	Akhilesh Modi; Apurvasinh Puvar; Bhadreshsinh Gohil; Chaitanya Joshi; Disha Vora; Janvi Raval; Jaykumar Rangani; Madhvi Joshi; Nimesh Patel; Nitin Savaliya; NitinShukla; Priyank Chavda; Ramesh Pandit; Roshani Mishra; Sonal Sharma; Tasnim Trivedi; Vicky Gandhi; Zarna Patel
EPI_ISL_8527573	Gandhi Medical College and Hospital (GMCH), Secunderabad	NIV Influenza	D.R.Manisha Rani; Dr.G.Sushma Rajya Lakshmi; Dr.K.Nagamani; Dr.Sunitha Pakalapaty
EPI_ISL_9191078, EPI_ISL_9709602, EPI_ISL_10807135, EPI_ISL_12695408, EPI_ISL_12695413, EPI_ISL_12739731, EPI_ISL_12739732, EPI_ISL_12740210, EPI_ISL_12982112			
see above	Genetica Molecular and Subdepartamento de Virologia ISP Chile	Instituto de Salud Publica de Chile	Andres Castillo; Barbara Parra; Constanza Campano; Ivan Ponce; Jorge Fernandez; Karen Orostica; Marcelo Rojas; Matias Pezoa; Patricia Bustos; Paulo covarrubias; Rodrigo Fasce
EPI_ISL_11855026, EPI_ISL_11855027, EPI_ISL_11855030, EPI_ISL_11855042, EPI_ISL_11859468, EPI_ISL_11859477, EPI_ISL_11859490, EPI_ISL_11859500			
see above	Gorgas Memorial Institute of Health Studies	Gorgas Memorial Institute of Health Studies	Abdiel Menacho; Alexander A Martinez; Ambar Moreno; Brechla Moreno; Claudia Gonzalez; Danilo Franco; Jessica Gondola; Leyda Abrego; Oris Chavarria
EPI_ISL_9850712	H Divino Espirito Santo - Ponta Delgada	Instituto Nacional de Saude (INSA)	Borges et al
EPI_ISL_13169662	HD-SANTA RITA	Laboratorio Central de Salud Pública	Analía Rojas; Andrea Gómez de la Fuente; Cynthia Vazquez; César Cantero; César Ojeda; Emmanuel Céspedes; Fátima Fleitas; Ivana Fernández; Juan Torales; Julio Barrios; Maria Liz Gamarra; María Jose Duarte; Sandra González; Shirley Villalba; Tania Alfonzo; Wilson Benítez.
EPI_ISL_12958919	HOSP. ALVAREZ BUYLLA	Laboratorio de Virología HUCA	; Alba L; Alvarez-Arguelles ME; Boga JA; Costales I; Coto E; González-Alba JM; Gómez de Oña J; Martín-Rodríguez G; Melón S; Perez-Martínez Z; Rojo S; Sandoval M
EPI_ISL_13169672	HOSPITAL DISTRITAL-HERNANDARIAS	Laboratorio Central de Salud Pública	Analía Rojas; Andrea Gómez de la Fuente; Cynthia Vazquez; César Cantero; César Ojeda; Emmanuel Céspedes; Fátima Fleitas; Ivana Fernández; Juan Torales; Julio Barrios; Maria Liz Gamarra; María Jose Duarte; Sandra González; Shirley Villalba; Tania Alfonzo; Wilson Benítez.
EPI_ISL_13169649, EPI_ISL_13169651, EPI_ISL_13169654, EPI_ISL_13169663, EPI_ISL_13169667	HR-CIUDAD DEL ESTE	Laboratorio Central de Salud Pública	Analía Rojas; Andrea Gómez de la Fuente; Cynthia Vazquez; César Cantero; César Ojeda; Emmanuel Céspedes; Fátima Fleitas; Ivana Fernández; Juan Torales; Julio Barrios; Maria Liz Gamarra; María Jose Duarte; Sandra González; Shirley Villalba; Tania Alfonzo; Wilson Benítez.
EPI_ISL_12422409	Hospital Alemán	Área de Secuenciación del Laboratorio de Virología del Hospital de Niños Dr. Ricardo Gutierrez on behalf of 'Proyecto Argentino Interinstitucional de genomica de SARS-CoV-2' (PAIS Consortium)	Acuña; D; Eugenia Ibañez; Goya; LE; Lusso; MI; MS; María Paula Della Latta; Nabaes Jodar; Natale; Natalia García allende; S; Valinotto; Viegas, M.
EPI_ISL_12476734	Hospital Borga Roma Verona	Laboratory of Medical Microbiology, University of Antwerp	Basil Britto Xavier; Evelina Tacconelli; Mathias Smet; Matilda Berkell; Surbhi Malhotra-Kumar
EPI_ISL_11579837, EPI_ISL_11579849	Hospital Center de Niort	Centre Hospitalier Universitaire (CHU) Poitiers	Agnes Beby-Defaux; Jean Philippe Da Mota; Luc Deroche; Magali Garcia; Manon Prat; Nicolas Leveque
EPI_ISL_11580270	Hospital Center de Saintonge	Centre Hospitalier Universitaire (CHU) Poitiers	Agnes Beby-Defaux; Jean Philippe Da Mota; Luc Deroche; Magali Garcia; Manon Prat; Nicolas Leveque
EPI_ISL_11788147, EPI_ISL_12184538	Hospital Clínico Universitario Virgen de la Arrixaca	Hospital Clínico Universitario Virgen de la Arrixaca	Laura Moreno Parrado and Luis Javier Gil-Gallardo.; Marina Simón Páez
EPI_ISL_11160306, EPI_ISL_11160321	Hospital General Universitario Gregorio Marañón	Hospital General Universitario Gregorio Marañón	Cristina Rodriguez-Grande; Darío García de Viedma; Jorge Rodríguez-Grande; Julia Suárez; Laura Pérez-Lago; Marta Herranz Martin; Patricia Muñoz; Pedro Sola Campoy; Pilar Catalán; Rosalía Palomino Cabrera; Sergio Buenestado Serrano
EPI_ISL_13156314	Hospital Nacional del Cáncer	Laboratorio Central de Salud Pública	Andrea Gómez de la Fuente; Cesar Cantero; Cynthia Vazquez; Juan Torales; Julio Barrios; Maria Liz Gamarra; Sandra Gonzalez; Shirley Villalba; Tania Alfonzo
EPI_ISL_12173793, EPI_ISL_12573062, EPI_ISL_12883432	Hospital Sao Rafael - H.S.R/BA	Plataforma de Vigilancia Molecular (PVM) - FIOCRUZ/BA	Ana Verena Almeida Mendes; Bruno Bezerril Andrade; Bruno Solano de Freitas Souza; Camila Araujo de Lorenzo Barcia; Camila I. de Oliveira; Carolina Kymie Vasques Nonaka; Clarissa Araujo Gurgel; Eduardo Oyama; Icaro Strobel; Izabela Jesus; Laise de Moraes; Leonardo Paiva Farias; Luisa Pedrosa; Marilia Miranda Franco; Marina Cucco; Ricardo Khouri on behalf of the Fiocruz COVID-19 Genomic Surveillance Network; Tiago Graf
EPI_ISL_10607983, EPI_ISL_10608192, EPI_ISL_10823591, EPI_ISL_10823791	Houston Methodist Hospital	Houston Methodist Hospital	Akanksha Batajoo; James J. Davis; James M. Musser; Jessica Cambric; Jimmy Gollihar; Jordan Pachuca; Kristina Reppond; Madison N. Shyer; Matthew Ojeda Saavedra; Nicole Kanellopoulos; Paul A. Christensen; Randall J. Olsen; Rashi M. Thakur; Regan Mangham; Richard Snehal; Robert Olson; Ryan Gadd; S. Wesley Long; Sindy Pena; Sinjini Gupta; Yuvanesh Vedaraju
EPI_ISL_9419339, EPI_ISL_9494928	ICMR-National Institute of Virology - INSACOG	NIV Influenza	Dr. Varsha Potdar and NIC Team
EPI_ISL_12220146	INSPI-Dirección_de_Idi	INSPI-Dirección_de_Idi	Andrés Carrazco-Montalvo; Ariana León; Clara Lucía Tello; David Guizado; Leandro Patiño
EPI_ISL_10826351	IZSM	TIGEM	Antonio Grimaldi Patrizia Annunziata Francesco Panariello Claudia Tiberio Teresa Giuliano Michela Daniele Valentina Bouche Ilaria Cimmino Chiara Colantuono Lucio Di Filippo Anna Manfredi Marcello Salvi Antonio Limone Luigi Atripaldi Andrea Ballabio Davide Cacchiarelli
EPI_ISL_9112451	Idaho Bureau of Laboratories	Idaho Bureau of Laboratories	Aimee Ceniseros; Christian Loera; Christopher Ball; Matthew Charles Burns; R. Beukelman; Robert L. Voermans
EPI_ISL_8886600	Indira Gandhi Memorial Hospital	Indira Gandhi Memorial Hospital	D. Fathmath Nazla Rafeeq; Dr. Ibrahim Afzal; Mr. Ibrahim Nishan Ahmed; Ms. Aishath Shuhudha; Ms. Aminath Shazleena Abdul Rahman; Ms. Soafy Mohamed
EPI_ISL_12359245	Innlandet Hospital Trust, Division Lillehammer, Department for Medical Microbiology	Norwegian Institute of Public Health, Department of Virology	Atiya R Ali; Debech Nadia; Engebretsen Serina Beate; Garcia Llorente Ignacio; Hilde Elshaug; Hilde Nordby Falkenhaug; Hilde Vollan; Jon Bråte; Kamilla Heddeland Instefjord; Karoline Bragstad; Kathrine Stene-Johansen; Line Victoria Moen; Marie Paulsen Madsen; Olav Hungnes; Pedersen Benedikte Nevjen; Rasmus Riis Kopperud
EPI_ISL_9087004, EPI_ISL_10204533, EPI_ISL_10204562, EPI_ISL_10552893, EPI_ISL_10985604, EPI_ISL_10985613, EPI_ISL_10985639, EPI_ISL_11073373			

see above	Institute for Water Quality and Resource Management, Technical University Vienna	Bergthaler laboratory, CeMM Research Center for Molecular Medicine of the Austrian Academy of Sciences	Andreas Bergthaler; Anna Schedl; Bekir Erguner; Benedikt Agerer; Christoph Bock; Fabian Amman; Jan Laine; Lukas Endler; Martin Senekowitsch; Matthew Thornton; Michael Schuster; Michelle Chan; Petr Triska; Thomas Penz
EPI_ISL_10204530, EPI_ISL_11073242, EPI_ISL_11073289	Institute of Legal Medicine, Medical University of Innsbruck	Bergthaler laboratory, CeMM Research Center for Molecular Medicine of the Austrian Academy of Sciences	Andreas Bergthaler; Anna Schedl; Bekir Erguner; Benedikt Agerer; Christoph Bock; Fabian Amman; Jan Laine; Lukas Endler; Martin Senekowitsch; Matthew Thornton; Michael Schuster; Michelle Chan; Petr Triska; Thomas Penz
EPI_ISL_9504693, EPI_ISL_9893350, EPI_ISL_9893629, EPI_ISL_9952817, EPI_ISL_9958034, EPI_ISL_11554269, EPI_ISL_11590727, EPI_ISL_11590865, EPI_ISL_11613412, EPI_ISL_11613419, EPI_ISL_12284836, EPI_ISL_12285171, EPI_ISL_12285177, EPI_ISL_13271851			
see above	Institute of Microbiology and Immunology, Faculty of Medicine, University of Ljubljana	Institute of Microbiology and Immunology, Faculty of Medicine, University of Ljubljana	Alen Suljič; Andraž Celar; Domen Lazar; Doroteja Vlaj; Mario Poljak; Miša Korva; Patricija Pozvek; Samo Zakotnik; Tatjana Avšič – Županc; Tina Gabrovšek; Tina Živič; Tomaž Mark Zorec; Špela Pleh
EPI_ISL_12424913	Instituto Rene Rachou / Fiocruz Minas	Instituto Rene Rachou / Fiocruz Minas	On behalf of the Fiocruz COVID-19 Genomic Surveillance Network
EPI_ISL_12754735	Irrua Specialist Teaching Hospital (ISTH)	Africa Centre for Excellence for Genomics of Infectious Diseases (ACEGID), Redeemer's University	A.T.; Abechi; Ajogbasile; Akano; C.A.; C.T.; Eromon; F.V.; Folarin, O.; Happi; I.B.; J.N.; J.U.; John; K.O.; Kayode; Nosamiefan, I.; O.G.; Oguzie; Olawoye; Olumade; Oluniyi; P.E.; P.S.; T.J.; Ugwu; Uwanibe
EPI_ISL_12771189	JKN PAHANG	iPROMISE, UiTM	Ariza Adnan; Fadzilah Mohd Nor; Lim Wai Feng; Mohd Asif Mohd Sukri; Mohd Nur Fakhruzzaman Noorizhab; Mohd Zaki Salleh; Sazzli Shahlan Kassim; Siti Farah Alwani Mohd Nawi; Siti Hamimah Sheikh Abdul Kadir; Teh Lay Kek; Wang Seok Mui
EPI_ISL_9306388	Jerusalem Central Laboratories	Israel Central Virology laboratory	Danit Sofer; Efrat Dahan Bucris; Ella Mendelson; Evan Nachum; Hagar Morad; Julia Vainer; Maya Davidovich; Michal Mandelboim; Michal Zak; Miranda Geva; Neta Zuckerman; Or Zilbertzan; Oran Erster; Orna Mor; Rona Grossman
EPI_ISL_8589447	Jessa	Jessa	Severine Berden et al. on behalf of the Jessa_cmdLab
EPI_ISL_13360911	KU Leuven, Rega Institute, Clinical and Epidemiological Virology	KU Leuven, Rega Institute, Clinical and Epidemiological Virology	Anne-Sophie Logist; Bert Vanmechelen; Jens Verlinden; Levi Ysebaert; Piet Maes; Robbe Sinnesael; Tony Wawina-Bokalanga
EPI_ISL_11327789	King Chulalongkorn Memorial Hospital	Thai Red Cross Emerging Infectious Diseases Clinical Center and Faculty of Medicine, Chulalongkorn University	Anthony R. Jones; Anutsara Jitsatja; Chanchanit Phanlop; Chonticha Klungthong; Gompol Suwanpimolkul; Khajohn Joonlasak; Leilani Paitoonpong; Nattakarn Thippamom; Opass Putcharoen; Pattama Torvorapanit; Piyawan Chinnawirotpisan; Sasiprapa Ninwattana; Stefan Fernandez; Supaporn Wacharapluesadee; Watsamon Jantarabenjakul; Wudtichai Manasatienkij
EPI_ISL_9143681, EPI_ISL_9143696, EPI_ISL_9143731	Klinika za infektivne bolesti "Dr. Fran Mihaljević"	Hrvatski zavod za javno zdravstvo	Anita Jurić; Dragan Jurić; Irena Tabain; Ivana Ferenčak; Josipa Kuzle
EPI_ISL_9446261	Klinikum Stuttgart	Robert Koch Institute	
EPI_ISL_8886623, EPI_ISL_8886624	Kudahuvadhoo Atoll Hospital	Kudahuvadhoo Atoll Hospital	D. Fathmath Nazla Rafeeq; Dr. Ibrahim Afzal; Mr. Ibrahim Nishan Ahmed; Ms. Aishath Shuhudha; Ms. Aminath Shazleena Abdul Rahman; Ms. Soafy Mohamed
EPI_ISL_8886610	Kulhudufushi Regional Hospital	Kulhudufushi Regional Hospital	D. Fathmath Nazla Rafeeq; Dr. Ibrahim Afzal; Mr. Ibrahim Nishan Ahmed; Ms. Aishath Shuhudha; Ms. Aminath Shazleena Abdul Rahman; Ms. Soafy Mohamed
EPI_ISL_11579864	LABOffice	Centre Hospitalier Universitaire (CHU) Poitiers	Agnes Beby-Defaux; Jean Philippe Da Mota; Luc Deroche; Magali Garcia; Manon Prat; Nicolas Leveque
EPI_ISL_11133791	LESP Baja California Sur	Instituto de Diagnostico y Referencia Epidemiologicos (INDRE)	Abril Rodriguez-Maldonado; Ana Cota-Magana; Ariadna Medina-Benitez; Claudia Wong-Arambula; Ernesto Ramirez-Gonzalez.; Fernando Gonzalez-Dominguez; Gisela Barrera-Badillo; Irma Lopez-Martinez; Joaquin Quiroz-Mercado; Lucia Hernandez-Rivas; Maribel Gonzalez-Villa; Natividad Cruz-Ortiz; Tatiana Nunez-Garcia; Vanessa Rivero-Arredondo
EPI_ISL_9749754	LESP Chihuahua	Instituto de Diagnostico y Referencia Epidemiologicos (INDRE)	Abril Rodriguez-Maldonado; Ariadna Medina-Benitez; Claudia Wong-Arambula; Ernesto Ramirez-Gonzalez.; Fernando Gonzalez-Dominguez; Gisela Barrera-Badillo; Irma Lopez-Martinez; Joaquin Quiroz-Mercado; Lucia Hernandez-Rivas; Maribel Gonzalez-Villa; Natividad Cruz-Ortiz; Sergio Rangel-Guerrero; Tatiana Nunez-Garcia; Vanessa Rivero-Arredondo
EPI_ISL_9749680	LESP Colima	Instituto de Diagnostico y Referencia Epidemiologicos (INDRE)	Abril Rodriguez-Maldonado; Ariadna Medina-Benitez; Claudia Wong-Arambula; Ernesto Ramirez-Gonzalez.; Fernando Gonzalez-Dominguez; Gisela Barrera-Badillo; Irma Lopez-Martinez; Joaquin Quiroz-Mercado; Lucia Hernandez-Rivas; Maribel Gonzalez-Villa; Natividad Cruz-Ortiz; Sergio Rangel-Guerrero; Tatiana Nunez-Garcia; Vanessa Rivero-Arredondo
EPI_ISL_9749779	LESP Guanajuato	Instituto de Diagnostico y Referencia Epidemiologicos (INDRE)	Abril Rodriguez-Maldonado; Ariadna Medina-Benitez; Claudia Wong-Arambula; Ernesto Ramirez-Gonzalez.; Fernando Gonzalez-Dominguez; Gisela Barrera-Badillo; Irma Lopez-Martinez; Joaquin Quiroz-Mercado; Lucia Hernandez-Rivas; Maribel Gonzalez-Villa; Natividad Cruz-Ortiz; Sergio Rangel-Guerrero; Tatiana Nunez-Garcia; Vanessa Rivero-Arredondo
EPI_ISL_9749556, EPI_ISL_9749557	LESP Jalisco	Instituto de Diagnostico y Referencia Epidemiologicos (INDRE)	Abril Rodriguez-Maldonado; Ariadna Medina-Benitez; Claudia Wong-Arambula; Ernesto Ramirez-Gonzalez.; Fernando Gonzalez-Dominguez; Gisela Barrera-Badillo; Irma Lopez-Martinez; Joaquin Quiroz-Mercado; Lucia Hernandez-Rivas; Maribel Gonzalez-Villa; Natividad Cruz-Ortiz; Sergio Rangel-Guerrero; Tatiana Nunez-Garcia; Vanessa Rivero-Arredondo
EPI_ISL_9749573	LESP Mexico City	Instituto de Diagnostico y Referencia Epidemiologicos (INDRE)	Abril Rodriguez-Maldonado; Ariadna Medina-Benitez; Claudia Wong-Arambula; Ernesto Ramirez-Gonzalez.; Fernando Gonzalez-Dominguez; Gisela Barrera-Badillo; Irma Lopez-Martinez; Joaquin Quiroz-Mercado; Lucia Hernandez-Rivas; Maribel Gonzalez-Villa; Natividad Cruz-Ortiz; Sergio Rangel-Guerrero; Tatiana Nunez-Garcia; Vanessa Rivero-Arredondo
EPI_ISL_9749656	LESP Sonora	Instituto de Diagnostico y Referencia Epidemiologicos (INDRE)	Abril Rodriguez-Maldonado; Ariadna Medina-Benitez; Claudia Wong-Arambula; Ernesto Ramirez-Gonzalez.; Fernando Gonzalez-Dominguez; Gisela Barrera-Badillo; Irma Lopez-Martinez; Joaquin Quiroz-Mercado; Lucia Hernandez-Rivas; Maribel Gonzalez-Villa; Natividad Cruz-Ortiz; Sergio Rangel-Guerrero; Tatiana Nunez-Garcia; Vanessa Rivero-Arredondo
EPI_ISL_9749697	LESP Tabasco	Instituto de Diagnostico y Referencia Epidemiologicos (INDRE)	Abril Rodriguez-Maldonado; Ariadna Medina-Benitez; Claudia Wong-Arambula; Ernesto Ramirez-Gonzalez.; Fernando Gonzalez-Dominguez; Gisela Barrera-Badillo; Irma Lopez-Martinez; Joaquin Quiroz-Mercado; Lucia Hernandez-Rivas; Maribel Gonzalez-Villa; Natividad Cruz-Ortiz; Sergio Rangel-Guerrero; Tatiana Nunez-Garcia; Vanessa Rivero-Arredondo
EPI_ISL_11133813, EPI_ISL_11133845	LESP Veracruz	Instituto de Diagnostico y Referencia Epidemiologicos (INDRE)	Abril Rodriguez-Maldonado; Ana Cota-Magana; Ariadna Medina-Benitez; Claudia Wong-Arambula; Ernesto Ramirez-Gonzalez.; Fernando Gonzalez-Dominguez; Gisela Barrera-Badillo; Irma Lopez-Martinez; Joaquin Quiroz-Mercado; Lucia Hernandez-Rivas; Maribel Gonzalez-Villa; Natividad Cruz-Ortiz; Tatiana Nunez-Garcia; Vanessa Rivero-Arredondo
EPI_ISL_11133876	LESP Yucatan	Instituto de Diagnostico y Referencia Epidemiologicos (INDRE)	Abril Rodriguez-Maldonado; Ana Cota-Magana; Ariadna Medina-Benitez; Claudia Wong-Arambula; Ernesto Ramirez-Gonzalez.; Fernando Gonzalez-Dominguez; Gisela Barrera-Badillo; Irma Lopez-Martinez; Joaquin Quiroz-Mercado; Lucia Hernandez-Rivas; Maribel Gonzalez-Villa; Natividad Cruz-Ortiz; Tatiana Nunez-Garcia; Vanessa Rivero-Arredondo
EPI_ISL_10296073	LKO	Jessa	Severine Berden et al. on behalf of the Jessa_cmdLab
EPI_ISL_9058428	Lab. Microbiologia e Virologia Cotugno A.O. dei Colli	TIGEM	Antonio Grimaldi Patrizia Annunziata Francesco Panariello Claudia Tiberio Teresa Giuliano Valentina Bouche Chiara Colantuono Michela Daniele Lucio Di Filippo Anna Manfredi Marcello Salvi Antonio Limone Luigi Atripaldi Andrea Ballabio Davide Cacchiarelli
EPI_ISL_12521761, EPI_ISL_13014643	Labor ZOTZ|KLIMAS; MVZ Düsseldorf-Centrum	Robert Koch Institute	
EPI_ISL_11293098	Laboratoire National de Santé Publique – LNSP	Genomics and Proteomics Departament, Gorgas Memorial Institute For Health Studies	Alexander A Martinez; Ambar Moreno; Claudia Gonzalez V; Ito Journel; Jaques Boncy; Jessica Gondola; Oris Chavarria; Patrick Delly
EPI_ISL_11110790	Laboratorio Central de Epidemiologia (LCE)	Unidad de Genomica Avanzada	Alejandro Sanchez-Flores; Alfredo Herrera-Estrella; Alicia Ocana-Mondragon; Angel Gustavo Salas-Lais; Bernardo Martinez-Miguel; Blanca Taboada; Brenda Irasema Maldonado-Meza; Carla Ivon Herrera-Najera; Carlos F. Arias; Celia Boukadida; Clara Esperanza Santacruz-Tinoco; Concepcion Grajales-Muniz; Consorcio Mexicano de Vigilancia Genomica (CoViGen-Mex). Authors (in alphabetical order): Julio Elias Alvarado-Yaah; Fernando Fontove-Herrera; Francisco Pulido; Gloria Elena Espinoza-Ayala; Gloria Maria Molina-Salinas; Gloria Vazquez; Hector Esteban Paz-Juarez; Hector Montoya-Fuentes; Helen Haydee Fernanda Ramirez-Plascencia; Jorge Ivan Salinal-Nevarez; Jose Antonio Enciso-Moreno; Jose Esteban Munoz-Medina; Jose de Jesus Nunez-Contreras; Juan Bautista Chale-Dzul; Luis Alberto Ochoa-Carrera; Margarita Matias-Florentino; Maria Guadalupe Santiago-Mauricio; Maria Guadalupe de Jesus Mireles-Rivera; Nelly Selem-Mojica; Pavel Isa; Ricardo Grande; Santiago Avila-Rios; Victor Hugo Borja-Aburto
EPI_ISL_9747755, EPI_ISL_10706152	Laboratorio Municipal de Biologia Molecular	Instituto Rene Rachou / Fiocruz Minas	Andre Menezes; Anna Salim; Caroline Rocha; Eneida Oliveira; Flavio Araujo; Gabriel Fernandes; On behalf of COVID-19 Fiocruz genomic network; Pedro Alves; Rubens do Monte Neto; Thais Silva
EPI_ISL_9801678	Laboratorio Nacional de Salud (LNS), Guatemala	Laboratorio Nacional de Salud (LNS), Guatemala	García G; Mendoza L
EPI_ISL_11629415, EPI_ISL_11629427	Laboratorio Nacional de Salud, Ministerio de Salud Publica y Asistencia Social	Genomics and Proteomics Departament, Gorgas Memorial Institute For Health Studies	Alexander A Martinez; Ambar Moreno; Claudia Estrada; Claudia Gonzalez V; César Roberto Conde Pereira; Jessica Gondola; Leyda Abrego; Marlene Castillo; Melissa Gaitan; Oris Chavarria
EPI_ISL_12422403	Laboratorio de Virologia del Hospital de Niños Dr Ricardo Gutierrez	Área de Secuenciación del Laboratorio de Virología del Hospital de Niños Dr. Ricardo Gutierrez on behalf of 'Proyecto Argentino Interinstitucional de genomica de SARS-CoV-2' (PAIS Consortium)	Acuña; Alejandra Musto; Alicia Mistchenko; Carla Medina; Cristian Díaz; Cristian Turchiaro; D; Erica Grandis; Erica Luczak; Estela Chascón; Goya; Guillermo Thomas; Isabel Desimone; Jorgelina Caruso; Julián Cipelli; Karina Zacarías; LE; Lorena Serrano; Lusso; MI; MS; Mariana Campal; Mariángeles Barreda Fink; María Cristina Alvarez López; María Elina Acevedo; María Emilia Villegas; Nabaes Jodar; Natale; Natalia Labarta; Omar Grossi; Oscar Jacquez; Oscar Luna; Raquel Barquez; Rubén Pelagamos; S; Sofía Alexay; Streitenberger Cintia; Valinotto; Viegas, M.
EPI_ISL_12422397	Laboratorio del Hospital Interzonal General de Agudos "Evita"	Área de Secuenciación del Laboratorio de Virología del Hospital de Niños Dr. Ricardo Gutierrez on behalf of 'Proyecto Argentino Interinstitucional de genomica de SARS-CoV-2' (PAIS Consortium)	Acuña; Alejandra Musto; D; Erica Luczak; Goya; Isabel Desimone; LE; Lorena Serrano; Lusso; MI; MS; Nabaes Jodar; Natale; Omar Grossi; Rubén Pelagamos; S; Valinotto; Viegas, M.
EPI_ISL_12422410	Laboratorio del Hospital Zonal Especializado Materno Infantil "Argentina Diego"	Área de Secuenciación del Laboratorio de Virología del Hospital de Niños Dr. Ricardo Gutierrez on behalf of 'Proyecto Argentino Interinstitucional de genomica de SARS-CoV-2' (PAIS	Acuña; D; Farinella Bagnozzi y Santiago Hernán Gauna; Goya; Guillermina; LE; Lusso; MI; MS; María Belén Arpaia; Nabaes Jodar; Natale; S; Valinotto; Viegas, M.

		Consortium)	
EPI_ISL_10775606	Laboratory of Genomics and Bioinformatics, Comenius University Science Park	Laboratory of Genomics and Bioinformatics, Comenius University Science Park	Anna Kaliňáková; Barbora Kotvasová; Diana Rusňáková; Jakub Styk; Jaroslav Budiš; Lucia Ševčíková; Michaela Jakubková Forgáčová; Miroslav Böhmer; Nikola Lipková; Pavol Mišenko; Silvia Bokorová; Tatiana Sedláčková; Terézia Vrabľová; Tomáš Szemes
EPI_ISL_13653682	Laboratory of Respiratory Viruses and Measles, Oswaldo Cruz Institute, FIOCRUZ	Laboratory of Respiratory Viruses and Measles, Oswaldo Cruz Institute, FIOCRUZ	Alice Sampaio Rocha; Bruna Mendonça da Silva; Elisa Cavalcante Pereira; Fernando Motta; Ighor Arantes; Jéssica Graça Macedo de Carvalho; Larissa Macedo Pinto; Luciana Appolinario; Marilda Siqueira on behalf of the Fiocruz COVID-19 Genomic Surveillance Network; Paola Resende; Victor Guimaraes
EPI_ISL_12708634, EPI_ISL_12708643	Laboratory of clinical bacteriology, Russian Children Clinical Hospital, Pirogov Medical University, Moscow, Russian Federation	Center for Precision Genome Editing and Genetic Technologies for Biomedicine, Pirogov Medical University, Moscow, Russian Federation	Denis Rebrikov; Dmitry Korostin; Fedorova Natalia; Iuliia Vasiliadis; Martynenkova Aliya; Natalia Ponikarovskaya; Vera Belova
EPI_ISL_11750291	Lembang General Hospital	West Java Health Laboratory; School of Life Sciences and Technology, Institut Teknologi Bandung	Azzania Fibriani; Cut Nur Cinthia Alamanda; Ema Rahmawati; Hadiana; Karimatu Khoirunnisa; Miftahul Faridl; Rifky Waluyajati Rachman; Rini Robiani; Ryan Bayusantika Ristandi
EPI_ISL_10693065, EPI_ISL_10693140, EPI_ISL_11325519, EPI_ISL_11561103	Lifebrain Covid Labor GmbH	Lifebrain Covid Labor GmbH	Abhishek Mitra; Alexandra Wagner; Anna Edermayr; Felix Valentin Spiegel; Filip Sima; Florian Scharhauser; Hannes Hagen; Kristina Bavrka Kolenc; Lucia Castello; So Jung Han
EPI_ISL_8699721, EPI_ISL_8704326, EPI_ISL_8743347, EPI_ISL_9901011, EPI_ISL_10201162, EPI_ISL_10202493, EPI_ISL_10428152, EPI_ISL_10525291, EPI_ISL_10531340, EPI_ISL_10856923, EPI_ISL_11044530, EPI_ISL_11088335, EPI_ISL_11652657			
see above	Lighthouse Lab in Glasgow	Wellcome Sanger Institute for the COVID-19 Genomics UK (COG-UK) Consortium	Anna Dominiczak and Alex Alderton; Carol Clugston; Cordelia Langford; David Gray; David K. Jackson; Dominic Kwiatkowski; Ewan Harrison; Harper VanSteenhouse; Ian Johnston; Jeffrey Barrett; John Sillitoe on behalf of the Wellcome Sanger Institute COVID-19 Surveillance Team; Roberto Amato; Sonia Goncalves; Yumi Kasai
EPI_ISL_9315229, EPI_ISL_10421352	Lighthouse Lab in Milton Keynes	Wellcome Sanger Institute for the COVID-19 Genomics UK (COG-UK) Consortium	Cordelia Langford; David K. Jackson; Dominic Kwiatkowski; Ewan Harrison; Ian Johnston; Jeffrey Barrett; John Sillitoe on behalf of the Wellcome Sanger Institute COVID-19 Surveillance Team; Roberto Amato; Sonia Goncalves; The Lighthouse Lab in Milton Keynes and Alex Alderton
EPI_ISL_10062804	Mae Sot General Hospital	Thailand MOPH - U.S. CDC Collaboration (TUC)	Beth Skaggs; Piroon Jenjaroenpun; Pongpun Sawatwong; Thidathip Wongsurawat
EPI_ISL_9506904, EPI_ISL_9957098, EPI_ISL_9957184	Medical Microbiology Unit, Department for Laboratory Medicine, Drammen Hospital, Vestre Viken Health Trust	Norwegian Institute of Public Health, Department of Virology	Atiya R Ali; Debech Nadia; Engebretsen Serina Beate; Garcia Llorente Ignacio; Hilde Elshaug; Hilde Nordby Falkenhaug; Hilde Vollan; Jon Bråte; Kamilla Heddeland Instefjord; Karoline Bragstad; Kathrine Stene-Johansen; Line Victoria Moen; Marie Paulsen Madsen; Olav Hungnes; Pedersen Benedikte Nevjen; Rasmus Riis Kopperud
EPI_ISL_10070736	Medical Virology and BSL3 Laboratory	Centre de Séquençage Génomique	Abbad Anas; Amalou Ghita; Anga Latifa; Barakat Abdelhamid; Bouzidi Aymane; Charoute Hicham; Chgouri Fatima; Dersi Noureddine; EL Hamouchi Adil; El Oualid Abdelmjid; Faouzi Abdellah; Harmak Houda; Maaroufi Abderrahmane; Nadifiyine Saloua; Nourlil Jalal; Omondi Francis Carey; Somda Soro Georgina Charlene; Zemmouri Faouzia; Zouheir Yassine
EPI_ISL_12027090	Medizinisch-Diagnostisches Labor Kempten allgäulab	Robert Koch Institute	
EPI_ISL_10079374	Michigan Department of Health and Human Services, Bureau of Laboratories	Michigan Department of Health and Human Services, Bureau of Laboratories	Blankenship HM; Riner D; Soehnlen MK
EPI_ISL_9848150, EPI_ISL_10844464	Microbiology Department, Laboratori Clínic Metropolitana Nord. Hospital Universitari Germans Trias i Pujol	Can Ruti SARS-CoV-2 Sequencing Hub (HUGTiP/IrsiCaixa/IGTP)	Alexia París; Ana Blanco; Andreu Coello; Antoni E Bordoy; Bonaventura Clotet; David Panisello; Francesc Catala-Moll; Gemma Clara; Ignacio Blanco; Laia Soler; Lauro Sumoy; Marc Noguera-Julian; Mercedes Guerrero; Montserrat Giménez; Pere-Joan Cardona; Pilar Armengol; Roger Paredes; Sara González; Verónica Saludes; and Elisa Martró on behalf of the Can Ruti SARS-CoV-2 Sequencing Hub
EPI_ISL_11248019	Microbiology Department. Complexo Hospitalario Universitario de Vigo	Microbiology Department. Complexo Hospitalario Universitario de Vigo	Alvarez M; Cabrera JJ; Carballo R; Cortizo S; Davina C; Martinez L; Mediero G; Pena I; Perez S; Potel C; Regueiro B; Rey S; Vasallo FJ; del-Campo V
EPI_ISL_8843407, EPI_ISL_8929501, EPI_ISL_8929502, EPI_ISL_10443635, EPI_ISL_10720896, EPI_ISL_10720897, EPI_ISL_10720905, EPI_ISL_10721105, EPI_ISL_10721173, EPI_ISL_10721212, EPI_ISL_10721232, EPI_ISL_10858408, EPI_ISL_10858415, EPI_ISL_10858472, EPI_ISL_10858530, EPI_ISL_11507575, EPI_ISL_13113844			
see above	Ministry of Health Turkey	Ministry of Health Turkey	Fatma Bayrakdar; Gulay Korukluoglu; Gülay Korukluoğlu; Gültekin Ünal; Suleyman Yalcin; Süleyman Yalcin; Yasemin Cosgun; Yasemin Coşgun
EPI_ISL_10008460, EPI_ISL_10008485	Ministry of Public Health / Hamad Medical Corporation	Biomedical Research Center (BRC), Qatar University / Qatar Genome Project (QGP)	Asmaa A. Al-Thani. MOPH and HMC: Abdullatif Al-Khal; BRC: Heba A. Al-Khatib; Chadi Saad; Dana Al-Batsh; Dina Elgakhlab. QGP: Fatima H. Al-Kuwari; Einas A. E. Al-Kuwari; Fatiha M. Benslimane; Fatma Hassan Ali; Hadeel T. Mohammed; Hadi M. Yassine; Hamad E. Al-Romaihi; Hamda Alromaihi; Maria K. Smatti; Mashael A. Al-Bader; Mohammed Al-Thani; Muna A. S. Al-Maslamani; Ola Al-Jamal; Peter V. Coyle; Reham A. El-Kahlout. QBB: Tasneem Al-Hamad; Roberto Bertollini; Salih Al-Marri; Swapna Thomas
EPI_ISL_12949403, EPI_ISL_12949519, EPI_ISL_12949521, EPI_ISL_12949546	Molecular Genetic Monitoring Group	Molecular Genetic Monitoring Group	Anna S. Gladkikh; Areg A.Totolian; Ekaterina O. Klyuchnikova; Valerya A. Sbarzaglia; Vladimir G. Dedkov
EPI_ISL_9457547, EPI_ISL_9860069	Nastavni zavod za javno zdravstvo Splitsko- Dalmatinske županije	Hrvatski zavod za javno zdravstvo	Anita Jurić; Dragan Jurić; Irena Tabain; Ivana Ferenčak; Josipa Kuzle
EPI_ISL_10854133, EPI_ISL_13180065	National Center of Infectious and Parasitic Diseases	National Center of Infectious and Parasitic Diseases	Alexiev et al
EPI_ISL_10083919, EPI_ISL_10083923, EPI_ISL_10083927, EPI_ISL_10083929, EPI_ISL_10083930, EPI_ISL_10083934	National Influenza Center	National Influenza Center	A Nejati; Adel Abedi; Adel Abedi and T Mokhtari Azad; J Yavarian; K Sadeghi; NZ Shafiei Jandaghi; Sevrin Zadheidar; Sevrin Zadheidar and T Mokhtari Azad; V Salimi
EPI_ISL_9417774, EPI_ISL_9823338	National Influenza Center, Virology Department	National Influenza Center	A Nejati; Adel Abedi and T Mokhtari Azad; J Yavarian; K Sadeghi; NZ Shafiei Jandaghi; Sevrin Zadheidar; V Salimi
EPI_ISL_9967402	National Institute of Infectious Diseases-Prof. Dr. Matei Bals Molecular Diagnostics Laboratory	National Institute of Infectious Diseases-Prof. Dr. Matei Bals Molecular Diagnostics Laboratory	Corina Casangiu; Dan Otelea; Leontina Banica; Marius Surleac; Ovidiu Vlaicu; Petre Milu; Robert Hohan; Simona Paraschiv
EPI_ISL_11982371, EPI_ISL_11982379, EPI_ISL_11982383, EPI_ISL_11982390, EPI_ISL_11982393, EPI_ISL_11982434, EPI_ISL_11982438, EPI_ISL_11982462, EPI_ISL_11982489, EPI_ISL_11982516, EPI_ISL_11982542, EPI_ISL_11982584			
see above	National Virus Reference Laboratory	National Virus Reference Laboratory	Charlene Bennett; Cillian F De Gascun; Gabriel Gonzalez; Jonathan Dean; Michael Carr; Zoe Yandle
EPI_ISL_12631494	NordLab Kokkola	Expert Microbiology, National Institute for Health and Welfare	Aino Palva; Carita Savolainen-Kopra; Emma Saarinen; Erika Lindh; Haider al-Hello; Jani Halkilahti; Kirsi Liitsola; Niina Ikonen; Olli Vapalahti; Phuoc Truong; Päivi Laurila; Ravi Kant; Sari Hannula; Soile Blomqvist; Suvi Kyttänen; Teemu Smura; Tiina Hannunen
EPI_ISL_9695869, EPI_ISL_9815626, EPI_ISL_10261068, EPI_ISL_10264569, EPI_ISL_10264638, EPI_ISL_10583127, EPI_ISL_10863341, EPI_ISL_11006863, EPI_ISL_11231939, EPI_ISL_11232756, EPI_ISL_11320379, EPI_ISL_11751242			
see above	Originating lab: Wales Specialist Virology Centre Sequencing lab: Pathogen Genomics Unit	Public Health Wales Microbiology Cardiff Wales Specialist Virology Centre	Alec Birchley; Alexander Adams; Amy Gaskin; Angela Marchbank; Bree Gatica-Wilcox; Catherine Moore; Jason Coombes; Joanne Watkins; Joel Southgate; Johnathan Evans; Laura Gifford; Lauren Gilbert; Lee Graham; Malorie Perry; Matthew Bull; Nicole Pacchiarini; Sally Corden; Sara Kumziene-Summerhayes; Sara Rey; Sarah Taylor; Simon Cottrell; Sophie Jones; Tom Connor
EPI_ISL_10190124, EPI_ISL_10826158, EPI_ISL_11023944, EPI_ISL_11055381, EPI_ISL_13030492	Ospedale Santa Maria delle Grazie	TIGEM	Antonio Grimaldi Patrizia Annunziata Francesco Panariello Claudia Tiberio Teresa Giuliano Michela Daniele Valentina Bouche Ilaria Cimmino Chiara Colantuono Lucio Di Filippo Anna Manfredi Marcello Salvi Antonio Limone Luigi Atripaldi Andrea Ballabio Davide Cacchiarelli; Antonio Grimaldi Patrizia Annunziata Francesco Panariello Claudia Tiberio Teresa Giuliano Tonya Fusco Valentina Bouche Ilaria Cimmino Chiara Colantuono Lucio Di Filippo Anna Manfredi Marcello Salvi Antonio Limone Luigi Atripaldi Andrea Ballabio Davide Cacchiarelli
EPI_ISL_11939211	PKD TIMUR LAUT	iPROMISE, UiTM	Ariza Adnan; Fadzilah Mohd Nor; Lim Wai Feng; Mohd Asif Mohd Sukri; Mohd Nur Fakhruzzaman Noorizhab; Mohd Zaki Salleh; Sazzli Shahlan Kassim; Siti Farah Alwani Mohd Nawi; Siti Hamimah Sheikh Abdul Kadir; Teh Lay Kek; Wang Seok Mui
EPI_ISL_12771264	PKDSPT	iPROMISE, UiTM	Ariza Adnan; Fadzilah Mohd Nor; Lim Wai Feng; Mohd Asif Mohd Sukri; Mohd Nur Fakhruzzaman Noorizhab; Mohd Zaki Salleh; Sazzli Shahlan Kassim; Siti Farah Alwani Mohd Nawi; Siti Hamimah Sheikh Abdul Kadir; Teh Lay Kek; Wang Seok Mui
EPI_ISL_9388271	PPHL Bheri	National Public Health Laboratory	National Public Health Laboratory Team
EPI_ISL_10101148	PRVKP FKUI	National Quality Control Laboratory of Drug and Food	Dillin N; Hana Apsari Pawestri; M. Erdiansyah; Sri Utaminingsih
EPI_ISL_9854383, EPI_ISL_10348671, EPI_ISL_10675280, EPI_ISL_10675284, EPI_ISL_10675322, EPI_ISL_10818821, EPI_ISL_10922766, EPI_ISL_11000733, EPI_ISL_12626318			
see above	PathWest Laboratory Medicine WA	PathWest Laboratory Medicine WA Microbial Surveillance Unit	PathWest Laboratory Medicine WA Microbial Surveillance Unit
EPI_ISL_11332334	Pathology Queensland and	Public Health Virology - Forensic	Chenwei Wang on behalf of Q-PHIRE Genomics

	Forensic Scientific Services	and Scientific Services	
EPI_ISL_10511960	Piyavate Hospital	National Institute of Health, Department of Medical Sciences, Ministry of Public Health, Thailand	Archawin Rojanawiwat; Natchaya Khiadsang; Nuttida Thongpramul; Pakorn Piromtong; Pilailuk Okada; Sirikanda Wimol; Siripaporn Phuygun; Sunthareeya Waicharoen; Suratchana Mitrat; Thanutsapa Thanadachakul
EPI_ISL_12422407	Plataforma de Servicios Biotecnólogicos: UTTIPP/PSB , Universidad Nacional de Quilmes.	Área de Secuenciación del Laboratorio de Virología del Hospital de Niños Dr. Ricardo Gutierrez on behalf of 'Proyecto Argentino Interinstitucional de genomica de SARS-CoV-2' (PAIS Consortium)	Acuña; Alejandra Zinni; Carla Capobianco; D; Georgina Cardama; Goya; Gustavo Bada; Hernán Farina; Humberto Lamdan; LE; Lusso; MI; MS; Marcelo Mandile; Nabaes Jodar; Natale; Norailys Lorenzo; S; Sandra Goñi; Valinotto; Viegas, M.
EPI_ISL_12573083	Plataforma de Vigilancia Molecular (PVM) - FIOCRUZ/BA	Plataforma de Vigilancia Molecular (PVM) - FIOCRUZ/BA	Bruno Bezerril Andrade; Camila I. de Oliveira; Clarissa Araujo Gurgel; Eduardo Oyama; Icaro Strobel; Izabela Jesus; Laise de Moraes; Leonardo Paiva Farias; Luisa Pedrosa; Marina Cucco; Ricardo Khouri on behalf of the Fiocruz COVID-19 Genomic Surveillance Network; Tiago Graf
EPI_ISL_8928596, EPI_ISL_8931071	Polski Bank Komórek Macierzystych	WSSE w Warszawie	Dorota Wagrocka - Roczniak
EPI_ISL_9353081	Public Health Authority of the Slovak Republic	Regional Authority of Public Health Banska Bystrica	Alžbeta Pristyáková; Diana Rusňáková; Lucia Maďarová; Michaela Mancoš; Miroslav Böhmer; Pavol Mišenko; Soňa Feiková; Terézia Tomajková; Tomáš Szemes
EPI_ISL_9561478	Quadram Institute Bioscience	COVID-19 Genomics UK (COG-UK) Consortium	Alexander J Trotter; Ana Martínez Gascueña; Andrew J. Page; Darcie Burgess; Dave J. Baker; Leonardo de Oliveira Martins; Lizzie Meadows; Martin Lott; Michaela Matthews; Nabil-Fareed Alikhan; Rebecca Sutcliffe; Rhiannon Evans; Thanh Le-Viet
EPI_ISL_11449558, EPI_ISL_11449559	Queen Sirikit National Institute of Child Health	National Institute of Health, Department of Medical Sciences, Ministry of Public Health, Thailand	Archawin Rojanawiwat; Natchaya Khiadsang; Nuttida Thongpramul; Pakorn Piromtong; Pilailuk Okada; Sirikanda Wimol; Siripaporn Phuygun; Sunthareeya Waicharoen; Suratchana Mitrat; Thanutsapa Thanadachakul
EPI_ISL_9371494, EPI_ISL_9371898	REUNILAB	UMR PIMIT	Dr Camille Lebarbenchon; Dr David A Wilkinson; Dr Jonathan Turpin; Dr Patrick Mavingui; Magali Turpin; Marie-Alice Simbi
EPI_ISL_9232416	Rainbow Hospital	CSIR-Centre for Cellular and Molecular Biology	Amareshwar Vodapalli; Ara Sreenivas; Archana Bharadwaj Siva; B Himasri; Divya Tej Sowpati; Jandhyala Sai Krishna; Kalyan Ram Uppaluri; Karthik Bharadwaj Tallapaka; Lamuk Zaveri; Malini Nemalikanti; Priya Nurkurthy; Rakesh K Mishra; Shreekant Verma; Smita Juvvadi; Sreelekshmi MS; Sricharan Devineni; Sumedha Avadhanula; Surabhi Srivastava; Tulasi Nagabandi; Valli Nagalakshmi Undamatla; Vidhyadhari Methuku
EPI_ISL_10987670	Regional Hospital Kladno	National Institute of Public Health	Alexander Nagy; Helena Jirincova; Jan Moskalyk; Jaromira Vecerova; Timotej Suri
EPI_ISL_9588925, EPI_ISL_9686940, EPI_ISL_11155050, EPI_ISL_11655400, EPI_ISL_11809578	Respiratory Virus Unit, Microbiology Services Colindale, Public Health England	COVID-19 Genomics UK (COG-UK) Consortium	PHE Covid Sequencing Team
EPI_ISL_11607503	Rosalind Franklin Laboratory	Wellcome Sanger Institute for the COVID-19 Genomics UK (COG-UK) Consortium	Cordelia Langford; David K. Jackson; Dominic Kwiatkowski; Donald Fraser; Ewan Harrison; Ian Johnston; Jeffrey Barrett; John Sillitoe on behalf of the Wellcome Sanger Institute COVID-19 Surveillance Team; Rob Howes; Roberto Amato; Sonia Goncalves; Suki Lee; The Rosalind Franklin Laboratory and Alex Alderton
EPI_ISL_10856693, EPI_ISL_10856789, EPI_ISL_10856799	SARS-CoV-2 Sequencing Castilla y Leon-Spain Consortium	SARS-CoV-2 Sequencing Castilla y Leon-Spain Consortium	Antonio Orduña-Domingo; Carlos Fuster Foz; Carmen Aldea-Mansilla; Carmen Gimeno Crespo; David Abad; Gabriel March Rosello; Gregoria Megías Lobón; Jose María Eiros Bouza; M. Isabel Fernandez-Natal; Marta Dominguez-Gil; Marta Hernandez; María Antonia García Castro; Mª Fe Brezmes-Valdivieso; Noelia Arenal Andrés; Silvia Rojo; Sonsoles Garcinuño Pérez
EPI_ISL_8920071, EPI_ISL_8920389, EPI_ISL_9303534, EPI_ISL_10495551	SARS-CoV-2 testing team, National Institute of Infectious Diseases	Pathogen Genomics Center, National Institute of Infectious Diseases	Hazuka Y Furihata; Kentaro Itokawa; Makoto Kuroda; Masanori Hashino; Masumichi Saito; Naomi Nojiri; Nozomu Hanaoka; Rina Tanaka; Tsuguto Fujimoto; Tsuyoshi Sekizuka
EPI_ISL_9850777	SEEBMO	Instituto Nacional de Saude (INSA)	Borges et al
EPI_ISL_11883434	SMS MEDICAL COLLEGE,JAIPUR	NIV Influenza	Bharti Malhotra; Dinesh parsoya; Farah Deeba; Himanshu sharma; Neha Bhomia; Nita pal; Nivedita Gupta; Pragya D Yadav; Pratibha Sharma; Sudhir Bhandari; Swati Gautam; Varsha Potdar
EPI_ISL_9301654	SOMDEJPRABOROMRACHINEENART NATAWEE HOSPITAL.	Prince of Songkhla University	Anusara Wongkotsila; Chanon Kongkamon; Pongsakorn Choochuen; Samonrapat Surasombatpattana; Sarunyou Chusri; Surakameth Mahasirimongkol; Surasak Sangkhathat; Thammasin Ingviya; Thanit Sila; Virasakdi Chongsuvivatwong; Wison Laochareonsuk
EPI_ISL_10271164	SPZOZ K-KOŻLE	Wojewódzka Stacja Sanitarno-Epidemiologiczna w Katowicach	Adrian Miara; Agata Chajda; Beata Rozwadowska; Elżbieta Bartkowiak; Marta Albertyńska
EPI_ISL_10918897, EPI_ISL_11656659	SYNLAB MVZ Weiden	Robert Koch Institute	
EPI_ISL_12631592	SYNLAB Suomi	Expert Microbiology, National Institute for Health and Welfare	Aino Palva; Carita Savolainen-Kopra; Emma Saarinen; Erika Lindh; Haider al-Hello; Jani Halkilahti; Kirsi Liitsola; Niina Ikonen; Olli Vapalahti; Phuoc Truong; Päivi Laurila; Ravi Kant; Sari Hannula; Soile Blomqvist; Suvi Kyttänen; Teemu Smura; Tiina Hannunen
EPI_ISL_10932297, EPI_ISL_10932307, EPI_ISL_11503037	Saratov Regional Clinical Hospital	WHO National Influenza Centre Russian Federation	Andrey Komissarov; Artem Fadeev; Daria Danilenko; Dmitry Lioznov; Elena Nabieva; Georgii Bazykin; Kirill Varchenko; Ksenia Safina; Kseniya Komissarova; Maria Pisareva; Mikhail Bakaev; Nikita Yolshin; Oula Mansour; Tamila Musaeva; Veronika Eder
EPI_ISL_11813240	SataDiag	Expert Microbiology, National Institute for Health and Welfare	Aino Palva; Carita Savolainen-Kopra; Emma Saarinen; Erika Lindh; Haider al-Hello; Jani Halkilahti; Kirsi Liitsola; Niina Ikonen; Olli Vapalahti; Phuoc Truong; Päivi Laurila; Ravi Kant; Sari Hannula; Soile Blomqvist; Suvi Kyttänen; Teemu Smura; Tiina Hannunen
EPI_ISL_12972907	Shahrekord University of Medical Sciences	National Influenza Center	A Nejati; Adel Abedi; J Yavarian; K Sadeghi; NZ Shafiei Jandaghi; Sevrin Zadheidar and T Mokhtari Azad; V Salimi
EPI_ISL_13234656	Shizuoka Institute of Environment and Hygiene	Japan COVID-19 Open Data Consortium	Atsushi Toyoda; Hiromi Nagaoka; Hiroshi Mori; Ituro Inoue; Katsuyuki Ishigami; Ken Kurokawa; Masanori Arita; Satomi Asano; Shigeki Mitsunaga; Takatomo Fujisawa; Yasuhiro Tanizawa; Yono Arita
EPI_ISL_9301611, EPI_ISL_9301613, EPI_ISL_9301618	Songkhla Hospital	Prince of Songkhla University	Anusara Wongkotsila; Chanon Kongkamon; Pongsakorn Choochuen; Samonrapat Surasombatpattana; Sarunyou Chusri; Surakameth Mahasirimongkol; Surasak Sangkhathat; Thammasin Ingviya; Thanit Sila; Virasakdi Chongsuvivatwong; Wison Laochareonsuk
EPI_ISL_9301688, EPI_ISL_9301689	Songklanagarind hospital	Prince of Songkhla University	Anusara Wongkotsila; Chanon Kongkamon; Pongsakorn Choochuen; Samonrapat Surasombatpattana; Sarunyou Chusri; Surakameth Mahasirimongkol; Surasak Sangkhathat; Thammasin Ingviya; Thanit Sila; Virasakdi Chongsuvivatwong; Wison Laochareonsuk
EPI_ISL_12291218, EPI_ISL_12291238	State Public Health Laboratory - INSACOG	NIV Influenza	Avudaiselvi Rathinasamy; Darez Ahamed; Devi Monika Ayyagari Venkata; Gurunathan Subramanian; Hemashree Kannan; Kalpana Raghu; Rajesh Kumar Manivannan; Raju Sivadoss; Sampath Palani; Selvavinayagam Sivaprakasam
EPI_ISL_10612213, EPI_ISL_11849183	Swedish national genomic surveillance program of SARS-CoV-2	The Public Health Agency of Sweden	Alma Brolund; Emmi Andersson; Maria Lind Karlberg; Maximilian Riess; Swedish national genomic surveillance program of SARS-CoV-2
EPI_ISL_9698203, EPI_ISL_9698255, EPI_ISL_9963318, EPI_ISL_10444775, EPI_ISL_10444785, EPI_ISL_10444866, EPI_ISL_10444869, EPI_ISL_10444891, EPI_ISL_10444916			
see above	Synlab Eesti OÜ	1. Laboratory of Communicable Diseases (Estonia); 2. Eurofins Genomics Europe Sequencing GmbH	Abroi A.; Avi R.; Dotsenko L.; Epštein J.; Huik K.; Härma M-A.; Jaaniso E.; Kaarna K.; Kallas E.; Koppel I.; Kuzmin I.; Lahesaare A.; Lutsar I.; Metspalu M.; Milani L.; Naaber P.; Niglas H.; Oopkaup O.E.; Pauskar M.; Peterson H.; Päll T.; Ratnik K.; Raudvere U.; Reisberg T.; Sadikova O.; Sepp H.; Shablinskaja A.; Suija H.; Talas U.G.; Truusalu K.
EPI_ISL_10745657	UAB "Baltic Medics"	National Public Health Surveillance Laboratory	Ana Steponkiene; Danas Baksa; Jelena Razmuk; Lukas Vasionis; Lukas Zemaitis; Migle Gabrielaite; Svajune Muralyte
EPI_ISL_9606453, EPI_ISL_9850775	ULSNE - Braganca	Instituto Nacional de Saude (INSA)	Borges et al
EPI_ISL_10036476	UMC Groningen, Clinical Virology, Department of Medical Microbiology and Infection Prevention	UMC Groningen, Clinical Virology, Department of Medical Microbiology and Infection Prevention	Alexander Friedrich; Coretta Van Leer-Buter; Erley Lizarazo-Forero; Hubert Niesters; Lilli Gard; Marjolein Knoester; Monika Fliss; Sigrid Rosema; Xuewei Zhou
EPI_ISL_9760530	UW Virology Lab	UW Virology Lab	Alexander Greninger; Hong Xie; Isabel Arnould; Keith R Jerome; Meei-Li Huang; Pavitra Roychoudhury; Pooneh Hajian; Ricardo Perez; Saraswathi Sathees; Sean Ellis; Seffir T. Wendm; Shah Mohamed Bakhash
EPI_ISL_10953139	Unidad de Investigación Biomédica de Zacatecas (UIBZ)	Microbial Genomics Laboratory	; Alejandra García-Gasca; Alejandra Hernández-Terán; Alejandro Sánchez-Flores; Alfredo Herrera-Estrella; Alicia Ocaña-Mondragón; Andreu Comas-García; Angel Gustavo Salas-Lais; Antonio Loza Román; Bernardo Martínez-Miguel; Blanca Taboada; Brenda Irasema Maldonado-Meza; Bruno Gómez-Gil; Carla Ivón Herrera-Najera; Carlos F. Arias; Celia Boukadida; Clara Esperanza Santacruz-Tinoco; Concepción Grajales-Muñiz; Consorcio Mexicano de Vigilancia Genómica (CoViGen-Mex). Authors (in alphabetical order): Julio Elias Alvarado-Yaah; Cristóbal Cháidez-Quiróz; Célida Duque Molina; Célida Martínez-Rodríguez; Daniel Fregoso-Rueda; Daniel Lira Morales; Eduardo Becerril-Vargas; Fernando Fontove-Herrera; Fidencio Mejía-Nepomuceno; Francisco Pulido; Gloria Elena Espinosa-Ayala; Gloria María Molina-Salinas; Gloria Vazquez; Hector Esteban Paz-Juárez; Hector Montoya-Fuentes; Helen Haydee Fernanda Ramírez-Plascencia; Irvin González-López; Jean Pierre González; Jesús Hernández; Joel Armando Vázquez-Pérez.; Jorge Salas-Hernández; José Antonio Enciso-Moreno; José Arturo Martínez-Orozco; José Esteban Muñoz-Medina; José de Jesús Nuñez-Contreras; Juan Bautista Chale-Dzul; Julissa Enciso-Ibarra; Luis Alberto Ochoa-Carrera; Margarita Matías-Florentino; Mario Mújica-Sánchez; Marissa Perez-Garcia; María Guadalupe Santiago-Mauricio; María Guadalupe de Jesús Mireles-Rivera; Nelly Sélem-Mojica; Pavel Isa; Ricardo Ciria Merce; Ricardo Grande; Rosa María Gutiérrez Rios; Santiago Ávila-Ríos; Selene Zárate; Susana Lopez; Verónica Mata-Haro; Victor Eduardo García-Arias; Víctor Hugo Borja-Aburto
EPI_ISL_12650022	Unilabs Laboratory Medicine	Norwegian Institute of Public Health, Department of Virology	Atiya R Ali; Debech Nadia; Engebretsen Serina Beate; Garcia Llorente Ignacio; Hilde Elshaug; Hilde Nordby Falkenhaug; Hilde Vollan; Jon Bråte; Kamilla Heddeland Instefjord; Karoline Bragstad; Kathrine Stene-Johansen; Line Victoria Moen; Marie Paulsen Madsen; Olav Hungnes; Pedersen Benedikte Nevjen; Rasmus Riis Kopperud
EPI_ISL_9075007,	University Clinical Hospital of	University of Sarajevo, Veterinary	Goletic S; Goletic T; Herceg I; Hodzic A; Jazic A; Nicevic M; Ostojic M; Sabic E; Skocibusic S; Softic A; Terzic I

EPI_ISL_9075015, EPI_ISL_9075021	Mostar, Department of Microbiology and Molecular Diagnostics	Faculty, Laboratory for Molecular Diagnostic and Research	
EPI_ISL_8791223, EPI_ISL_9025461, EPI_ISL_9325219, EPI_ISL_10265762, EPI_ISL_10265780	Universität Zürich	Institute of Medical Virology, University of Zurich	Alexandra Trkola; Annette Audigé; Catharine Aquino; Cyril Shah; Daniel Ehrsam; Gabriela Ziltener; Guido Bloemberg; Hubert Rehrauer; Isabel Stürmer; Joel Wirz; Jon Huder; Jürg Böni; Kevin Steiner; Maria Grünberg; Maryam Zaheri; Michael Huber; Riccarda Capaul; Stefan Schmutz; Verena Kufner; Weihong Qi
EPI_ISL_9025918, EPI_ISL_9325281, EPI_ISL_9622656, EPI_ISL_9622690, EPI_ISL_9622710, EPI_ISL_9881460	UniversitätsSpital Zürich	Institute of Medical Virology, University of Zurich	Alexandra Trkola; Annette Audigé; Catharine Aquino; Cyril Shah; Daniel Ehrsam; Gabriela Ziltener; Guido Bloemberg; Hubert Rehrauer; Isabel Stürmer; Joel Wirz; Jon Huder; Jürg Böni; Kevin Steiner; Maria Grünberg; Maryam Zaheri; Michael Huber; Riccarda Capaul; Stefan Schmutz; Verena Kufner; Weihong Qi
EPI_ISL_10314277	Universitätsklinikum Köln; Institut für Virologie	Robert Koch Institute	
EPI_ISL_11905699	Urmia Reference Laboratory	National Influenza Center	A Nejati; Adel Abedi; J Yavarian; K Sadeghi; Marzieh Faraji-Zonouz and T Mokhtari Azad; NZ Shafiei Jandaghi; Nastaran Ghavami Sevrin Zadheidar; V Salimi
EPI_ISL_9402090, EPI_ISL_9402097	VERMAAK	National Institute for Communicable Diseases of the National Health Laboratory Service	Amoako DG; Bhiman JN; Everatt J; Ismail A; Kekana D; Mahlangu B; Mnguni A; Mohale T; Ntuli N; Scheepers C; Wolter N
EPI_ISL_10851409	Viesoji istaiga Klaipedos universitetine ligonine	National Public Health Surveillance Laboratory	Ana Steponkiene; Danas Baksa; Jelena Razmuk; Lukas Vasionis; Lukas Zemaitis; Migle Gabrielaite; Svajune Muralyte
EPI_ISL_11299840	Viollier AG	Department of Biosystems Science and Engineering, ETH Zürich	Andrea Patrizia Salzmann; Chaoran Chen; Christian Beisel; Christiane Beckmann; Christoph Noppen; David Dreifuss; Elodie Burcklen; Franziska Singer; Henriette Kurth; Ina Nissen; Ivan Topolsky; Kim Philipp Jablonski; Lara Fuhrmann; Louis du Plessis; Matteo Carrara; Maurice Redondo; Mirjam Feldkamp; Natascha Santacroce; Niko Beerenwinkel; Olivier Kobel; Pelin Icer; Rebecca Denes; Sarah Nadeau; Shuqing Yu; Tanja Stadler; Tobias Schär
EPI_ISL_9353266, EPI_ISL_9353326, EPI_ISL_12172042, EPI_ISL_12172048, EPI_ISL_12172064	Virology Laboratory, Scientific Department, Army Medical Center	Virology Laboratory, Scientific Department, Army Medical Center	Anella Monte; Anna Anselmo; Antonella Fortunato; Filippo Molinari; Florigio Lista; Francesco Giordani; Giancarlo Petralito; Giandomenico Cerreto; Giulia Campoli; Lucia Nicosia; Marzia Cavalli; Pietro Marco D'Angelo; Riccardo De Sanctis; Rossella Brandi; Silvia Fillo; Vanessa Vera Fain
EPI_ISL_9154644, EPI_ISL_9978176, EPI_ISL_9984839	Vitomed Sp. z o.o.	1. Academic Center for Pathomorphological and Genetic-Molecular Diagnostics ltd, Bialystok, Poland 2. National Institute of Public Health - National Institute of Hygiene, Warsaw, Poland	Anetta Sulewska; Jacek Nikliński; Janusz Dzięcioł; Joanna Kiśluk; Katarzyna Zacharczuk; Konrad Raczkowski; Magdalena Nowakowska; Małgorzata Sadkowska-Todys; Piotr Karabowicz; Piotr Majewski; Przemysław Biecek. Joanna Reszeć; Radosław Charkiewicz; Tomasz Wołkowicz
EPI_ISL_11924903	West of Scotland Specialist Virology Centre, NHSGGC / MRC-University of Glasgow Centre for Virus Research	COVID-19 Genomics UK (COG-UK) Consortium	Alasdair MacLean; Ana da Silva Filipe; Andy Young; Antonia Ho; Daniel Mair; David L Robertson; Emily Goldstein; Emma Thomson; Gonzalo Yebra; Guy Mollett; Ioulia Tsatsani; James Shepherd; Jenna Nichols; Jessica Benkaroun; Jon Perkins; Jordan Ashworth; Joseph Hughes; Kathy Smollett; Kirsty Mangin; Kyriaki Nomikou; Lily Tong; Matthew Holden; Nicolas Suarez; Rachael Tomb; Rachel Blacow; Richard Orton; Rory Gunson; Sarah McDonald; Sharif Shaaban; Sreenu Vattipally
EPI_ISL_9414680	White Buffalo, Inc	PSU Animal DiagnosticLaboratory	Meera Surendran Nair; Suresh Kuchipudi; Vivek Kapur
EPI_ISL_11934993	Yourgene Health	COVID-19 Genomics UK (COG-UK) Consortium	UKHSA; Yourgene Health
EPI_ISL_9202992	Zakład Patologii Nowotworów i Patomorfologii Centrum Onkologii im. prof. F. Łukaszczyka w Bydgoszczy	1. Academic Center for Pathomorphological and Genetic-Molecular Diagnostics ltd, Bialystok, Poland 2. National Institute of Public Health - National Institute of Hygiene, Warsaw, Poland	Anetta Sulewska; Jacek Nikliński; Janusz Dzięcioł; Joanna Kiśluk; Katarzyna Zacharczuk; Konrad Raczkowski; Magdalena Nowakowska; Małgorzata Sadkowska-Todys; Piotr Karabowicz; Piotr Majewski; Przemysław Biecek. Joanna Reszeć; Radosław Charkiewicz; Tomasz Wołkowicz
EPI_ISL_9860127	Zavod za javno zdravstvo Ličko-senjske županije	Hrvatski zavod za javno zdravstvo	Anita Jurić; Dragan Jurić; Irena Tabain; Ivana Ferenčak; Josipa Kuzle
EPI_ISL_9975863, EPI_ISL_9975913, EPI_ISL_9976348	Zavod za javno zdravstvo Varaždinske županije	Hrvatski zavod za javno zdravstvo	Anita Jurić; Dragan Jurić; Irena Tabain; Ivana Ferenčak; Josipa Kuzle
