# Supplementary material for: Tracing the international arrivals of SARS-CoV-2 Omicron variants after Aotearoa New Zealand reopened its border
Source: Nat Commun. 2022 Oct 29;13:6484. doi: 10.1038/s41467-022-34186-9 (PMC9617600; doi:10.1038/s41467-022-34186-9)
Supplement: Supplementary file 5 — Supplementary Data 3 [file 41467_2022_34186_MOESM5_ESM.pdf]

We gratefully acknowledge the following Authors from the Originating laboratories responsible for obtaining the specimens, as well as the Submitting laboratories where the genome data were generated and shared via GISAID, on which this research is based.

All Submitters of data may be contacted directly via www.gisaid.org

Authors are sorted alphabetically.

Acknowledgement EPI_SET Identifier: EPI_SET_20220706us

Accession ID	Originating Laboratory	Submitting Laboratory	Authors
EPI_ISL_12572794	AREA DE SALUD FORTUNA	Inciensa, Instituto Costarricense de Investigación y Enseñanza en Nutrición y Salud	Adriana Godínez; Claudio Soto-Garita; Estela Cordero; Francisco Duarte; Gabriel Morales & Karolina Hall Loria; Hebleen Porras; José Luis Vargas; Mariela Gutiérrez; Melany Calderón; Sofia Herrera
EPI_ISL_13109457	AREA DE SALUD GARABITO	Inciensa, Instituto Costarricense de Investigación y Enseñanza en Nutrición y Salud	Adriana Godínez; Claudio Soto-Garita; Estela Cordero; Francisco Duarte; Gabriel Morales; Hebleen Porras; José Luis Vargas; Mariela Gutiérrez; Melany Calderón; Natalia Bonilla & Andrea Moreno Carvajal; Sofia Herrera
EPI_ISL_13109453	AREA DE SALUD PAVAS (COOPESALUD)	Inciensa, Instituto Costarricense de Investigación y Enseñanza en Nutrición y Salud	Adriana Godínez; Claudio Soto-Garita; Estela Cordero; Francisco Duarte; Gabriel Morales & Natalia Bonilla; Hebleen Porras; José Luis Vargas; Mariela Gutiérrez; Melany Calderón; Sofia Herrera
EPI_ISL_12935273	AREA DE SALUD SANTA CRUZ	Inciensa, Instituto Costarricense de Investigación y Enseñanza en Nutrición y Salud	Adriana Godínez; Claudio Soto-Garita; Estela Cordero; Francisco Duarte; Gabriel Morales; Hebleen Porras; José Luis Vargas; Mariela Gutiérrez; Melany Calderón; Natalia Bonilla & Mónica Montoya; Sofia Herrera
EPI_ISL_13285186	ASST GOM NIGUARDA	ASST Grande ospedale Metropolitano Niguarda	Alice Nava
EPI_ISL_12918611, EPI_ISL_13087666, EPI_ISL_13227223, EPI_ISL_13227233	ASST MONZA	ASST MONZA	Sergio Maria Ivano Malandrin
EPI_ISL_12968865	American Samoa DOH Clinical Laboratory Tafuna Family Health Center	State Laboratories Division, Hawaii State Department of Health	Ayana Garnet; Briana Ofilas; Cheryl-lynn Daquip; Cheyenne Barela; Daniel Strange; Drew Kuwazaki; Edward Desmond; Jeffrey Au; Mark Nagata; Pamela O'Brien; Remedios Gose; Samantha Cotter; Samantha Sruba
EPI_ISL_13297838	Arcispedale Santa Maria Nuova Autoimmunità Allergologia e Biotecnologie Innovative	Istituto Zooprofilattico Sperimentale della Lombardia e dell'Emilia Romagna (IZSLER), Risk Analysis and Genomic Epidemiology Unit	Alessandro Zerbini; Erika Scaltriti; Ilaria Menozzi; Lucia Belloni; Marina Morganti; Stefania Croci; Stefano Pongolini
EPI_ISL_13199457	Area of Virology, Serology and Virology Division (SAViD), New South Wales Health Pathology Randwick	Virology Research Laboratory; Area of Virology, Serology and Virology Division (SAViD), New South Wales Health Pathology Randwick	Foster, C.; Jean, T.; Rawlinson, W.; Van Hal, S.; Wong, M.; Yeang, M.
EPI_ISL_13276915, EPI_ISL_13276921, EPI_ISL_13276942	Area of Virology, Serology and Virology Division (SAViD), New South Wales Health Pathology Randwick, Prince of Wales Hospital	Virology Research Laboratory, Area of Virology, Serology and Virology Division (SAViD), New South Wales Health Pathology Randwick, Prince Of Wales Hospital	Foster, C.; Jean, T.; Rawlinson, W.; Van Hal, S.; Wong, M.; Yeang, M.
EPI_ISL_12870016, EPI_ISL_12870018	Area of Virology, Serology and Virology Division (SAViD), New South Wales Health Pathology Randwick, Prince of Wales Hospital	Virology Research Laboratory; Area of Virology, Serology and Virology Division (SAViD), New South Wales Health Pathology Randwick, Prince Of Wales Hospital	Foster, C.; Jean, T.; Rawlinson, W.; Van Hal, S.; Wong, M.; Yeang, M.
EPI_ISL_13059280, EPI_ISL_13059307	Area of Virology, Serology and Virology Division (SAViD), New South Wales Health Pathology Randwick, Prince of Wales Hospital	Virology Research Laboratory; Area of Virology, Serology and Virology Division (SAViD), New South Wales Health Pathology Randwick, Prince of Wales Hospital	Foster, C.; Jean, T.; Rawlinson, W.; Van Hal, S.; Wong, M.; Yeang, M.
EPI_ISL_12808702	Australian Clinical Labs (formerly Healthscope Pathology)	NSW Health Pathology - Institute of Clinical Pathology and Medical Research; Westmead Hospital; University of Sydney	Arnott A.; Draper J.; Gall M.; Martinez E.; Rockett R.; Sintchenko V.; on behalf of ICPMR
EPI_ISL_12606027	Azienda Ospedaliero - Universitaria di Modena Policlinico - Virologia e Microbiologia Molecolare	Istituto Zooprofilattico Sperimentale della Lombardia e dell'Emilia Romagna (IZSLER), Risk Analysis and Genomic Epidemiology Unit	Erika Scaltriti; Giulia Fregni Serpini; Ilaria Menozzi; Marina Morganti; Monica Pecorari; Stefano Pongolini; William Gennari
EPI_ISL_12918854, EPI_ISL_13284343	Azienda Sanitaria dell'Alto Adige - Laboratorio Aziendale di Microbiologia e Virologia	Azienda Sanitaria dell'Alto Adige	Irene Bianconi
EPI_ISL_13031916, EPI_ISL_13282591, EPI_ISL_13282682, EPI_ISL_13282705, EPI_ISL_13291939, EPI_ISL_13292088, EPI_ISL_13292168, EPI_ISL_13292473, EPI_ISL_13292511			
see above	BioneXt Lab	Laboratoire national de sante, Microbiology, Microbial Genomics Platform	Anke Wienecke-Baldacchino; Catherine Ragimbeau; Elodie Solarino; Eric Hugoson; Fatu Djabi; Jessica Tapp; Lise Pignon; Raoul Salmon; Sibel Berger; Tamir Abdelrahman; Thibault Ferrandon; Virginie Jover
EPI_ISL_12966874, EPI_ISL_12966927, EPI_ISL_12967054, EPI_ISL_12967573, EPI_ISL_12967615, EPI_ISL_12968039	British Columbia Centre For Disease Control	B.C. Centre for Disease Control Public Health Laboratory	Ana Pacagnella; Corrinne Ng; Dan Fornika; James Zlosnik; John Tyson; Kim Macdonald; Kimia Kamelian; Linda Hoang; Loretta Janz; Mel Krajden; Prystajecky Natalie; Robert Azana; Shannon Russell
EPI_ISL_12381897, EPI_ISL_12382570, EPI_ISL_12384337, EPI_ISL_12661665, EPI_ISL_12663137, EPI_ISL_12663617, EPI_ISL_12664541, EPI_ISL_12664673			
see above	British Columbia Centre For Disease Control	BCCDC Public Health Laboratory	Ana Pacagnella; Corrinne Ng; Dan Fornika; James Zlosnik; John Tyson; Kim Macdonald; Kimia Kamelian; Linda Hoang; Loretta Janz; Mel Krajden; Prystajecky Natalie; Robert Azana; Shannon Russell
EPI_ISL_13068765, EPI_ISL_13068789, EPI_ISL_13068803, EPI_ISL_13068805, EPI_ISL_13068807, EPI_ISL_13068820, EPI_ISL_13068832, EPI_ISL_13223816, EPI_ISL_13223841			
see above	CHUV	Laboratory of genomics and metagenomics, Institute of Microbiology, University Hospital Centre and University of Lausanne	Claire Bertelli; Damien Jacot; Gilbert Greub; Sébastien Aeby; Trestan Pillonel
EPI_ISL_13177319	Centre Hospitalier Universitaire (CHU) Nimes	Centre Hospitalier Universitaire (CHU) Nîmes	Agathe Boudet; Milene Sasso; Sophie Bravo; Stephan Robin
EPI_ISL_13018096, EPI_ISL_13018097	Centro de Investigación Biomédica de Occidente (CIBO)	Microbial Genomics Laboratory	; Alejandra García-Gasca; Alejandra Hernández-Terán; Alejandro Sánchez-Flores; Alfredo Herrera-Estrella; Alicia Ocaña-Mondragón; Andreu Comas-García; Angel Gustavo Salas-Lais; Antonio Loza Román; Bernardo Martínez-Miguel; Blanca Taboada; Brenda Irasema Maldonado-Meza; Bruno Gómez-Gil; Carla Ivón Herrera-Najera; Carlos F. Arias; Celia Boukadida; Clara Esperanza Santacruz-Tinoco; Concepción Grajales-Muñiz; Consorcio Mexicano de Vigilancia Genómica (CoViGen-Mex). Authors (in alphabetical order): Julio Elias Alvarado-Yaah; Cristóbal Cháidez-Quiróz; Célida Duque Molina; Célida Martínez- Rodríguez; Daniel Fregoso-Rueda; Daniel Lira Morales; Eduardo Becerril-Vargas; Fernando Fontove-Herrera; Fidencio Mejía-Nepomuceno; Francisco Pulido; Gloria Elena Espinosa-Ayala; Gloria María Molina-Salinas; Gloria Vazquez; Hector Esteban Paz-Juárez; Hector Montoya-Fuentes; Helen Haydee Fernanda Ramírez-Plascencia; Irvin González-López; Jean Pierre González; Jesús Hernández; Joel Armando Vázquez-Pérez.; Jorge Salas-Hernández; José Antonio Enciso-Moreno; José Arturo Martínez-Orozco; José Esteban Muñoz-Medina; José de Jesús Nuñez-Contreras; Juan Bautista Chale-Dzul; Julissa Enciso-Ibarra; Luis Alberto Ochoa-Carrera; Margarita Matías-Florentino; Mario Mújica-Sánchez; Marissa Perez-Garcia; María Guadalupe Santiago-Mauricio; María Guadalupe de Jesús Mireles-Rivera; Nelly Sélem-Mojica; Pavel Isa; Ricardo Ciria Merce; Ricardo Grande; Rosa María Gutiérrez Rios; Santiago Ávila-Ríos; Selene Zárate; Susana Lopez; Verónica Mata-Haro; Victor Eduardo García-Arias; Víctor Hugo Borja-Aburto

EPI_ISL_13018124	Centro de Investigación Biomédica del Noreste (CIBIN)	Microbial Genomics Laboratory	; Alejandra García-Gasca; Alejandra Hernández-Terán; Alejandro Sánchez-Flores; Alfredo Herrera-Estrella; Alicia Ocaña-Mondragón; Andreu Comas-García; Angel Gustavo Salas-Lais; Antonio Loza Román; Bernardo Martínez-Miguel; Blanca Taboada; Brenda Irasema Maldonado-Meza; Bruno Gómez-Gil; Carla Ivón Herrera-Najera; Carlos F. Arias; Celia Boukadida; Clara Esperanza Santacruz-Tinoco; Concepción Grajales-Muñiz; Consorcio Mexicano de Vigilancia Genómica (CoViGen-Mex). Authors (in alphabetical order): Julio Elias Alvarado-Yaah; Cristóbal Cháidez-Quiróz; Célida Duque Molina; Célida Martínez- Rodríguez; Daniel Fregoso-Rueda; Daniel Lira Morales; Eduardo Becerril-Vargas; Fernando Fontove-Herrera; Fidencio Mejía-Nepomuceno; Francisco Pulido; Gloria Elena Espinosa-Ayala; Gloria María Molina-Salinas; Gloria Vazquez; Hector Esteban Paz-Juárez; Hector Montoya-Fuentes; Helen Haydee Fernanda Ramírez-Plascencia; Irvin González-López; Jean Pierre González; Jesús Hernández; Joel Armando Vázquez-Pérez.; Jorge Salas-Hernández; José Antonio Enciso-Moreno; José Arturo Martínez-Orozco; José Esteban Muñoz-Medina; José de Jesús Nuñez-Contreras; Juan Bautista Chale-Dzul; Julissa Enciso-Ibarra; Luis Alberto Ochoa-Carrera; Margarita Matías-Florentino; Mario Mújica-Sánchez; Marissa Perez-Garcia; María Guadalupe Santiago-Mauricio; María Guadalupe de Jesús Mireles-Rivera; Nelly Sélem-Mojica; Pavel Isa; Ricardo Ciria Merce; Ricardo Grande; Rosa María Gutiérrez Rios; Santiago Ávila-Ríos; Selene Zárate; Susana Lopez; Verónica Mata-Haro; Victor Eduardo García-Arias; Víctor Hugo Borja-Aburto
EPI_ISL_12916151, EPI_ISL_13074889, EPI_ISL_13075023, EPI_ISL_13075041, EPI_ISL_13075182	Clinical Microbiology Laboratory, Tel Aviv Sourasky Medical Center	Clinical Microbiology Laboratory, Tel Aviv Sourasky Medical Center	Alon Ziv; Amos Adler; Goel Morad; Katya Levytskyi; Lior Handler; Matan Slutzkin; Ora Halutz; Orly Eshel
EPI_ISL_13295814	Colorado Department of Public Health and Environment	Colorado Department of Public Health and Environment	Alexandria Rossheim; Arianna Smith; Diana Ir; Emily A. Travanty; Laura Bankers; Mandy Waters; Michael Martin; Molly C. Hetherington-Rauth; Shannon R. Matzinger
EPI_ISL_13035997	Department für Labormedizin Abteilung III Bereich Molekulare Diagnostik Universitätsklinikum Halle(Saale)	Robert Koch Institute	
EPI_ISL_12632417, EPI_ISL_12895353, EPI_ISL_12895738, EPI_ISL_12993951, EPI_ISL_12994838, EPI_ISL_13030124, EPI_ISL_13049829, EPI_ISL_13067091, EPI_ISL_13090399, EPI_ISL_13127770, EPI_ISL_13127884, EPI_ISL_13177784, EPI_ISL_13177852, EPI_ISL_13178841, EPI_ISL_13178943, EPI_ISL_13241580, EPI_ISL_13280703, EPI_ISL_13299539, EPI_ISL_13299658, EPI_ISL_13299782			
see above	Department of Bacteria, Parasites and Fungi, Statens Serum Institut, Copenhagen, Denmark	Statens Serum Institut Bioinformatics and Microbial Genomics	Danish Covid-19 Genome Consortium
EPI_ISL_13090220, EPI_ISL_13216471, EPI_ISL_13251688, EPI_ISL_13251692	Department of Clinical Microbiology	GIGA Medical Genomics	Claire Gourzonès; Cécile Meex; Keith Durkin; Laurent Gillet; Maria Artesi; Marie-Pierre Hayette; Nadine Cambisano; Nathalie Renotte; Olivier Ek; Sébastien Bontems; Vincent Bours
EPI_ISL_12812530, EPI_ISL_12812534, EPI_ISL_12812562, EPI_ISL_13140325, EPI_ISL_13140329, EPI_ISL_13140331, EPI_ISL_13140342, EPI_ISL_13140466, EPI_ISL_13140497, EPI_ISL_13140514, EPI_ISL_13140531, EPI_ISL_13140542, EPI_ISL_13140543, EPI_ISL_13140547, EPI_ISL_13140557			
see above	Department of Health Technology and Informatics, The Hong Kong Polytechnic University	Department of Health Technology and Informatics, The Hong Kong Polytechnic University	Alan Ka-Lun Wu; Alex Yat-Man Ho; Barry Kin-Chung Wong; Chloe Toi-Mei Chan; David Ho-Keung Shum; Gilman Kit-Hang Siu; Hiu-Yin Lao; Ivan Tak-Fai Wong; Jake Siu-Lun Leung; Kam-Tong Yip; Kenneth Siu-Sing Leung; Kingsley King-Gee Tam; Kitty Sau-Chun Fung; Kristine Luk; Lam-Kwong Lee; Miranda Chong-Yee Yau; Sandy Ka-Yee Chau; Shea Ping Yip; Tak-Lun Que; Timothy Ting-Leung Ng; Wing Cheong Yam; Wing-Hei Lo; Wing-Kin To; Yvette Wai-Man Lai
EPI_ISL_12471280, EPI_ISL_12869517, EPI_ISL_13132094	Department of Medical Microbiology & Infection prevention, Amsterdam University Medical Centers location AMC	Department of Medical Microbiology & Infection prevention, Amsterdam University Medical Centers location AMC	Akke Cornelissen; Fokla Zorgdrager; Janke Schinkel; Jelle Koopsen; Judith den Uil; Marcel Jonges; Matthijs Welkers; Menno de Jong; Menno de Jong and Mariken van der Lubben on behalf of the Amsterdam Regional Genomic epidemiology and Outbreak Surveillance (ARGOS) consortium; Robin van Houdt; Sebastien Matamoros; Sjoerd Rebers; Sylvia Bruisten; Tjalling Leenstra and Mariken van der Lubben on behalf of the Amsterdam Regional Genomic epidemiology and Outbreak Surveillance (ARGOS) consortium
EPI_ISL_13181259	Department of Medical Microbiology - section Molde, Molde Hospital	Norwegian Institute of Public Health, Department of Virology	Atiya R Ali; Debech Nadia; Engebretsen Serina Beate; Garcia Llorente Ignacio; Hilde Elshaug; Hilde Nordby Falkenhaug; Hilde Vollan; Jon Bråte; Kamilla Heddeland Instefjord; Karoline Bragstad; Kathrine Stene-Johansen; Line Victoria Moen; Marie Paulsen Madsen; Olav Hungnes; Pedersen Benedikte Nevjen; Rasmus Riis Kopperud
EPI_ISL_13182855	Department of Medical Microbiology, Baerum Hospital, Vestre Viken Health Trust	Norwegian Institute of Public Health, Department of Virology	Atiya R Ali; Debech Nadia; Engebretsen Serina Beate; Garcia Llorente Ignacio; Hilde Elshaug; Hilde Nordby Falkenhaug; Hilde Vollan; Jon Bråte; Kamilla Heddeland Instefjord; Karoline Bragstad; Kathrine Stene-Johansen; Line Victoria Moen; Marie Paulsen Madsen; Olav Hungnes; Pedersen Benedikte Nevjen; Rasmus Riis Kopperud
EPI_ISL_12981760, EPI_ISL_12982778, EPI_ISL_13181514	Department of Medical Microbiology, St. Olavs hospital	Norwegian Institute of Public Health, Department of Virology	Atiya R Ali; Debech Nadia; Engebretsen Serina Beate; Garcia Llorente Ignacio; Hilde Elshaug; Hilde Nordby Falkenhaug; Hilde Vollan; Jon Bråte; Kamilla Heddeland Instefjord; Karoline Bragstad; Kathrine Stene-Johansen; Line Victoria Moen; Marie Paulsen Madsen; Olav Hungnes; Pedersen Benedikte Nevjen; Rasmus Riis Kopperud
EPI_ISL_13182953	Dept. of Medical Microbiology, Stavanger University Hospital, Helse Stavanger HF	Norwegian Institute of Public Health, Department of Virology	Atiya R Ali; Debech Nadia; Engebretsen Serina Beate; Garcia Llorente Ignacio; Hilde Elshaug; Hilde Nordby Falkenhaug; Hilde Vollan; Jon Bråte; Kamilla Heddeland Instefjord; Karoline Bragstad; Kathrine Stene-Johansen; Line Victoria Moen; Marie Paulsen Madsen; Olav Hungnes; Pedersen Benedikte Nevjen; Rasmus Riis Kopperud
EPI_ISL_13090981	Dept. of Microbiology and Infection Control, Akershus University Hospital HF	Dept. of Microbiology and Infection Control, Akershus University Hospital HF	Alexander Hesselberg Løvestad; Divya Murugananthan; Hanne Berggreen; Hege Vangstein Aamot
EPI_ISL_13198059	Diagnostyka Sp. z o. o. Lodz	1. Academic Center for Pathomorphological and Genetic-Molecular Diagnostics ltd, Bialystok, Poland 2. National Institute of Public Health - National Institute of Hygiene, Warsaw, Poland	Anetta Sulewska; Jacek Niklinski; Janusz DzięcioL; Joanna Kiśluk; Katarzyna Zacharczuk; Konrad Raczkowski; MaLgorzata Sadkowska-Todys; Magdalena Nowakowska; Piotr Karabowicz; Piotr Majewski; PrzemysLaw Biecek. Joanna Reszeć; RadosLaw Charkiewicz; Tomasz WoLkowicz
EPI_ISL_12635082, EPI_ISL_13086514, EPI_ISL_13137582, EPI_ISL_13137913, EPI_ISL_13200241	Division of Emerging Infectious Diseases, Bureau of Infectious Diseases Diagnosis Control, Korea Disease Control and Prevention Agency	Division of Emerging Infectious Diseases, Bureau of Infectious Diseases Diagnosis Control, Korea Disease Control and Prevention Agency	Ae Kyung Park; Chae Young Lee; Eun-Jin Kim; Hyuck Jin Lee; Il-Hwan Kim; Jeong-Ah Kim
EPI_ISL_13084086, EPI_ISL_13084087	Division of Infectious Disease Diagnosis Control, Capital Regional Center for Disease Control and Prevention, Korea Disease Control and Prevention Agency, KDCA	Division of Emerging Infectious Diseases, Bureau of Infectious Diseases Diagnosis Control, Korea Disease Control and Prevention Agency	EunJung Lee; Jeong-Gu Nam; SaHyun Hong
EPI_ISL_13281606, EPI_ISL_13281618	Division of Infectious Disease Diagnosis Control, Gyeongnam Regional Center for Disease Control and Prevention, Korea Disease Control and Prevention Agency, KDCA	Division of Emerging Infectious Diseases, Bureau of Infectious Diseases Diagnosis Control, Korea Disease Control and Prevention Agency	Byung Hak Kang; Dongchul Park; Seon Do Hwang
EPI_ISL_12932997	Douglass Hanly Moir Pathology	NSW Health Pathology - Institute of Clinical Pathology and Medical Research; Westmead Hospital; University of Sydney	Arnott A.; Draper J.; Gall M.; Martinez E.; Rockett R.; Sintchenko V.; on behalf of ICPMR
EPI_ISL_13215555, EPI_ISL_13215597	Edmonton Provincial Lab	Alberta Precision Labs (APL)	Buss E; Croxen M; Deo A; Dieu P; Ferrato C; Gill K; Koleva P; Li V; Lloyd C; Lynch T; Ma R; Murphy S; Pabbaraju K; Shideler S; Shokopoles S; Skitsko T; Thayer J; Tipples G; Wong A; Yu C; Zelyas N.
EPI_ISL_13034611	Eurofins MVZ Labor Gelsenkirchen	Robert Koch Institute	
EPI_ISL_13134161, EPI_ISL_13294894	Eurofins-NMDL	Eurofins-NMDL	Anco Molijn; Anne Vogel; Lisa Dreesens; Marvin Ruiter; Maurine Leversteijn-van Hall; Roy Masius; Simon Lansu
EPI_ISL_13104272, EPI_ISL_13247784, EPI_ISL_13247811	Fondazione IRCCS Ca' Granda Ospedale Maggiore Policlinico	Fondazione IRCCS Ca' Granda Ospedale Maggiore Policlinico	Ferruccio Ceriotti; Sara Uceda Renteria
EPI_ISL_12223766, EPI_ISL_12613086, EPI_ISL_12613126, EPI_ISL_12953155, EPI_ISL_12953241, EPI_ISL_12953244, EPI_ISL_12953266, EPI_ISL_12953284, EPI_ISL_12953286, EPI_ISL_13305203, EPI_ISL_13305219, EPI_ISL_13305228, EPI_ISL_13305276, EPI_ISL_13307956, EPI_ISL_13310979, EPI_ISL_13311025, EPI_ISL_13311032, EPI_ISL_13311033, EPI_ISL_13311047			
see above	Gandhi Medical College and Hospital (GMCH), Secunderabad	NIV Influenza	Abdul Majeed; Amrithesh Kumar Arun; D.R.Manisha Rani; Devendhar; Dr.G.Sushma Rajya Lakshmi; Dr.K.Nagamani; Dr.Sunitha Pakalapaty; Hajeera Osmani; Sahithya
EPI_ISL_12739971, EPI_ISL_12740011	Genetica Molecular and Subdepartamento de Virologia ISP Chile	Instituto de Salud Publica de Chile	Andres Castillo; Barbara Parra; Constanza Campano; Ivan Ponce; Jorge Fernandez; Karen Orostica; Marcelo Rojas; Matias Pezoa; Patricia Bustos; Rodrigo Fasce
EPI_ISL_13040429	Genomics for Life	Public Health Virology - Forensic and Scientific Services (PHV-FSS)	Chenwei Wang on behalf of Q-PHIRE Genomics
EPI_ISL_12832916	HOSPITAL DE CIUDAD NEILY	Inciensa, Instituto Costarricense de	Adriana Godínez; Claudio Soto-Garita; Estela Cordero; Francisco Duarte; Gabriel Morales; Hebleen Porras; José Luis Vargas; Mariela Gutiérrez; Melany Calderón; Natalia Bonilla & Gabriela Marchena Jimenez; Sofia Herrera

		Investigación y Enseñanza en Nutrición y Salud	
EPI_ISL_12935315, EPI_ISL_12935316	HOSPITAL DR. RAFAEL A. CALDERON GUARDIA	Inciensa, Instituto Costarricense de Investigación y Enseñanza en Nutrición y Salud	Adriana Godínez; Claudio Soto-Garita; Estela Cordero; Francisco Duarte; Gabriel Morales; Hebleen Porras; José Luis Vargas; Mariela Gutiérrez; Melany Calderón; Natalia Bonilla & Ericka Madrigal Arias; Sofia Herrera
EPI_ISL_12832953	HOSPITAL DR. TOMAS CASAS CASAJUS	Inciensa, Instituto Costarricense de Investigación y Enseñanza en Nutrición y Salud	Adriana Godínez; Claudio Soto-Garita; Estela Cordero; Francisco Duarte; Gabriel Morales; Hebleen Porras; José Luis Vargas; Mariela Gutiérrez; Melany Calderón; Natalia Bonilla & Antony Orozco Barquero; Sofia Herrera
EPI_ISL_12935330	HOSPITAL GUAPILES	Inciensa, Instituto Costarricense de Investigación y Enseñanza en Nutrición y Salud	Adriana Godínez; Claudio Soto-Garita; Estela Cordero; Francisco Duarte; Gabriel Morales; Hebleen Porras; José Luis Vargas; Mariela Gutiérrez; Melany Calderón; Natalia Bonilla & Cesar Cerdas Quesada; Sofia Herrera
EPI_ISL_13209262, EPI_ISL_13209264, EPI_ISL_13209268, EPI_ISL_13209270, EPI_ISL_13209271, EPI_ISL_13209282, EPI_ISL_13209283			
see above	HOSPITAL UNIVERSITARIO CENTRAL DE ASTURIAS	Laboratorio de Virología HUCA	; Alba L; Alvarez-Arguelles ME; Boga JA; Costales I; Coto E; González-Alba JM; Gómez de Oña J; Martín-Rodríguez G; Melón S; Perez-Martínez Z; Rojo S; Sandoval M
EPI_ISL_13300448	Hia Brest	Centre Hospitalier Universitaire (CHU) Brest	Adissa Tran Minoui; Christopher Payan; Léa Pilorgé; Simon Rambaud; Sophie Vallet
EPI_ISL_13018129	Hospital Margarita Maza de Juárez	Microbial Genomics Laboratory	; Alejandra García-Gasca; Alejandra Hernández-Terán; Alejandro Sánchez-Flores; Alfredo Herrera-Estrella; Alicia Ocaña-Mondragón; Andreu Comas-García; Angel Gustavo Salas-Lais; Antonio Loza Román; Bernardo Martínez-Miguel; Blanca Taboada; Brenda Irasema Maldonado-Meza; Bruno Gómez-Gil; Carla Ivón Herrera-Najera; Carlos F. Arias; Celia Boukadida; Clara Esperanza Santacruz-Tinoco; Concepción Grajales-Muñiz; Consorcio Mexicano de Vigilancia Genómica (CoViGen-Mex). Authors (in alphabetical order): Julio Elias Alvarado-Yaah; Cristóbal Cháidez-Quiróz; Célida Duque Molina; Célida Martínez- Rodríguez; Daniel Fregoso-Rueda; Daniel Lira Morales; Eduardo Becerril-Vargas; Fernando Fontove-Herrera; Fidencio Mejía-Nepomuceno; Francisco Pulido; Gloria Elena Espinosa-Ayala; Gloria María Molina-Salinas; Gloria Vazquez; Hector Esteban Paz-Juárez; Hector Montoya-Fuentes; Helen Haydee Fernanda Ramírez-Plascencia; Irvin González-López; Jean Pierre González; Jesús Hernández; Joel Armando Vázquez-Pérez.; Jorge Salas-Hernández; José Antonio Enciso-Moreno; José Arturo Martínez-Orozco; José Esteban Muñoz-Medina; José de Jesús Nuñez-Contreras; Juan Bautista Chale-Dzul; Julissa Enciso-Ibarra; Luis Alberto Ochoa-Carrera; Margarita Matías-Florentino; Mario Mújica-Sánchez; Marissa Perez-Garcia; María Guadalupe Santiago-Mauricio; María Guadalupe de Jesús Mireles-Rivera; Nelly Sélem-Mojica; Pavel Isa; Ricardo Ciria Merce; Ricardo Grande; Rosa María Gutiérrez Rios; Santiago Ávila-Ríos; Selene Zárate; Susana Lopez; Verónica Mata-Haro; Victor Eduardo García-Arias; Víctor Hugo Borja-Aburto
EPI_ISL_13105923	Hospital Universitario 12 de Octubre	Hospital Universitario 12 de Octubre	Carmen Martín-Higuera; Esther Viedma; Irene Múñoz-Gallego; M.ª Dolores Folgueira; Mar Aguilera; Noelia Moral; Rafael Delgado; Sagrario Zurita
EPI_ISL_13304467, EPI_ISL_13304493, EPI_ISL_13304546, EPI_ISL_13304567, EPI_ISL_13304579	ICMR-National Institute of Virology - INSACOG	NIV Influenza	Dr. Varsha Potdar and NIC Team
EPI_ISL_12982710	Innlandet Hospital Trust, Division Lillehammer, Department for Medical Microbiology	Norwegian Institute of Public Health, Department of Virology	Atiya R Ali; Debech Nadia; Engebretsen Serina Beate; Garcia Llorente Ignacio; Hilde Elshaug; Hilde Nordby Falkenhaug; Hilde Vollan; Jon Bråte; Kamilla Heddeland Instefjord; Karoline Bragstad; Kathrine Stene-Johansen; Line Victoria Moen; Marie Paulsen Madsen; Olav Hungnes; Pedersen Benedikte Nevjen; Rasmus Riis Kopperud
EPI_ISL_13002093, EPI_ISL_13015511	Institut für Immunologie und Genetik Kaiserslautern: Medizinisches Labor Dr. med. B. Thiele	Robert Koch Institute	
EPI_ISL_13183482, EPI_ISL_13183510, EPI_ISL_13183517, EPI_ISL_13183534	Jessa	Jessa	Laura Vanstraelen et al. on behalf of the Jessa_cmdLab
EPI_ISL_13307011	Kasturba Hospital Molecular Lab	NIV Influenza	Jayanthi Shastri; Vidushi Chitalia
EPI_ISL_12905607, EPI_ISL_12905610	Kijabe Hospital	KEMRI-Wellcome Trust Research Programme,Kilifi	Agoti C.; D.J.Nokes; Githinji G.; Lambisia A.; Makori T.; Mburu M.W.; Mohamed K.S.; Morobe J.; Ndwiga L.; Ochola I; Ongera E.; de Laurent Z.
EPI_ISL_12854414, EPI_ISL_12854421, EPI_ISL_12854429, EPI_ISL_13032314, EPI_ISL_13032363	Klinika za infektivne bolesti "Dr. Fran Mihaljević"	Hrvatski zavod za javno zdravstvo	Anita Jurić; Dragan Jurić; Irena Tabain; Ivana Ferenčak; Josipa Kuzle
EPI_ISL_13141704	Klinikum Ernst von Bergmann gemeinnützige GmbH - stationärer Bereich	Robert Koch Institute	
EPI_ISL_12983339	Klinisch Laboratorium ZNA	Klinisch Laboratorium ZNA	Verstrepen et al.
EPI_ISL_13192071	LESP Sinaloa	Instituto de Diagnostico y Referencia Epidemiologicos (INDRE)	Abril Rodriguez-Maldonado; Ariadna Medina-Benitez; Claudia Wong-Arambula; Ernesto Ramirez-Gonzalez.; Fernando Gonzalez-Dominguez; Gisela Barrera-Badillo; Irma Lopez-Martinez; Joaquin Quiroz-Mercado; Lucia Hernandez-Rivas; Maribel Gonzalez-Villa; Natividad Cruz-Ortiz; Ruth Madera-Sandoval; Tatiana Nunez-Garcia; Vanessa Rivero-Arredondo
EPI_ISL_13192069	LESP Tabasco	Instituto de Diagnostico y Referencia Epidemiologicos (INDRE)	Abril Rodriguez-Maldonado; Ariadna Medina-Benitez; Claudia Wong-Arambula; Ernesto Ramirez-Gonzalez.; Fernando Gonzalez-Dominguez; Gisela Barrera-Badillo; Irma Lopez-Martinez; Joaquin Quiroz-Mercado; Lucia Hernandez-Rivas; Maribel Gonzalez-Villa; Natividad Cruz-Ortiz; Ruth Madera-Sandoval; Tatiana Nunez-Garcia; Vanessa Rivero-Arredondo
EPI_ISL_12953088, EPI_ISL_13133552	LKO	Jessa	Laura Vanstraelen et al. on behalf of the Jessa_cmdLab
EPI_ISL_13238512	Labor Blackholm MVZ	Robert Koch Institute	
EPI_ISL_12678403, EPI_ISL_12678503	Labor Mönchengladbach MVZ Dr. Stein + Kollegen GbR	Robert Koch Institute	
EPI_ISL_13244831	Labor Prof. Dr. G. Enders MVZ GbR	Robert Koch Institute	
EPI_ISL_12702619, EPI_ISL_13292379	Laboratoire national de sante, Microbiology, Virology	Laboratoire national de sante, Microbiology, Microbial Genomics Platform	Anke Wienecke-Baldacchino; Catherine Ragimbeau; Elodie Solarino; Eric Hugoson; Fatu Djabi; Jessica Tapp; Lise Pignon; Raoul Salmon; Sibel Berger; Tamir Abdelrahman; Thibault Ferrandon; Trung Nguyen Nguyen; Virginie Jover
EPI_ISL_13292055	Laboratoires Reunis	Laboratoire national de sante, Microbiology, Microbial Genomics Platform	Anke Wienecke-Baldacchino; Bernard Weber; Catherine Ragimbeau; Elodie Solarino; Eric Hugoson; Fatu Djabi; Jessica Tapp; Lise Pignon; Raoul Salmon; Sibel Berger; Tamir Abdelrahman; Virginie Jover
EPI_ISL_12452939, EPI_ISL_13282603, EPI_ISL_13292283, EPI_ISL_13292284, EPI_ISL_13292341, EPI_ISL_13292426	Laboratoires d'analyses medicales - KETTERTHILL	Laboratoire national de sante, Microbiology, Microbial Genomics Platform	Anke Wienecke-Baldacchino; Caroline Scheiber; Catherine Ragimbeau; Elodie Solarino; Eric Hugoson; Fatu Djabi; Jessica Tapp; Lise Pignon; Raoul Salmon; Serge Vedy; Sibel Berger; Tamir Abdelrahman; Virginie Jover
EPI_ISL_12837673, EPI_ISL_12837770	Laboratoires d'analyses medicales - KETTERTHILL	Microbiology, Microbial Genomics Platform, LNS Laboratoire National De Santé	Anke Wienecke-Baldacchino; Caroline Scheiber; Catherine Ragimbeau; Elodie Solarino; Eric Hugoson; Fatu Djabi; Jessica Tapp; Lise Pignon; Raoul Salmon; Serge Vedy; Sibel Berger; Tamir Abdelrahman; Virginie Jover
EPI_ISL_13002244	Laboratori de Referencia de Catalunya	Laboratori de Referencia de Catalunya	Bellosillo B.; Canal M.; Hernandez JJ.; Padilla E.; Ramirez A.; Vilas A.
EPI_ISL_13018078, EPI_ISL_13018086, EPI_ISL_13018100	Laboratorio Central de Epidemiología (LCE)	Microbial Genomics Laboratory	; Alejandra García-Gasca; Alejandra Hernández-Terán; Alejandro Sánchez-Flores; Alfredo Herrera-Estrella; Alicia Ocaña-Mondragón; Andreu Comas-García; Angel Gustavo Salas-Lais; Antonio Loza Román; Bernardo Martínez-Miguel; Blanca Taboada; Brenda Irasema Maldonado-Meza; Bruno Gómez-Gil; Carla Ivón Herrera-Najera; Carlos F. Arias; Celia Boukadida; Clara Esperanza Santacruz-Tinoco; Concepción Grajales-Muñiz; Consorcio Mexicano de Vigilancia Genómica (CoViGen-Mex). Authors (in alphabetical order): Julio Elias Alvarado-Yaah; Cristóbal Cháidez-Quiróz; Célida Duque Molina; Célida Martínez- Rodríguez; Daniel Fregoso-Rueda; Daniel Lira Morales; Eduardo Becerril-Vargas; Fernando Fontove-Herrera; Fidencio Mejía-Nepomuceno; Francisco Pulido; Gloria Elena Espinosa-Ayala; Gloria María Molina-Salinas; Gloria Vazquez; Hector Esteban Paz-Juárez; Hector Montoya-Fuentes; Helen Haydee Fernanda Ramírez-Plascencia; Irvin González-López; Jean Pierre González; Jesús Hernández; Joel Armando Vázquez-Pérez.; Jorge Salas-Hernández; José Antonio Enciso-Moreno; José Arturo Martínez-Orozco; José Esteban Muñoz-Medina; José de Jesús Nuñez-Contreras; Juan Bautista Chale-Dzul; Julissa Enciso-Ibarra; Luis Alberto Ochoa-Carrera; Margarita Matías-Florentino; Mario Mújica-Sánchez; Marissa Perez-Garcia; María Guadalupe Santiago-Mauricio; María Guadalupe de Jesús Mireles-Rivera; Nelly Sélem-Mojica; Pavel Isa; Ricardo Ciria Merce; Ricardo Grande; Rosa María Gutiérrez Rios; Santiago Ávila-Ríos; Selene Zárate; Susana Lopez; Verónica Mata-Haro; Victor Eduardo García-Arias; Víctor Hugo Borja-Aburto
EPI_ISL_13282998	Laboratorio di Patologia Clinica, Ospedale San Paolo in Valloria, ASL 2 Liguria	U.O. Igiene, Ospedale Policlinico San Martino	Bruzzone Bianca; De Pace Vanessa; Domnich Alexander; Icardi Giancarlo on behalf of SARS-CoV-2 ITALIAN RESEARCH ENTERPRISE-(SCIRE) Collaborative Group; Lillo Flavia; Orsi Andrea; Randazzo Nadia; Ricucci Valentina; Stefanelli Federica
EPI_ISL_12594388, EPI_ISL_12620925, EPI_ISL_12758416, EPI_ISL_12889656, EPI_ISL_12889672, EPI_ISL_12974019, EPI_ISL_13080443, EPI_ISL_13118718, EPI_ISL_13151881, EPI_ISL_13161853, EPI_ISL_13219717, EPI_ISL_13309702, EPI_ISL_13314752, EPI_ISL_13315013, EPI_ISL_13315577			
see above	Laboratory Corporation of	Centers for Disease Control and	Amanda Douglas; Amanda Suchanek; Andrea Throop; Ayla Burns; Benjamin Rambo-Martin; Bobbi Croy; Brian Krueger; Brian Norvell; Christopher Gulvick; Christos Petropoulos; Clinton Paden; Craig Lukasik; Dakota Howard; Debbie Boles; Dhwani Batra; Duncan MacCannell; Eyad Almasri; Goran Stevovic; Howard

	America	Prevention Division of Viral Diseases, Pathogen Discovery	Engler; Hrushikesh Deshmukh; Jake Humphrey; Jana Schroth; Jason Caravas; Joe Voshell; John Pruitt; Jonathan Meltzer; Jonathan Williams; Kimberly Wagner; Kristine Lacek; Lax Iyer; Lisa Pfefferle; Lyndon Tilson; Manoj Jain; Marcia Eisenberg; Mary Cristobal; Mary Williamson; Matthew Robinson; Matthew Schmerer; Michael Levandoski; Mike Sapeta; Mindy Nye; Minoo Agarwal; Mohan Kolli; Nuthawin Charoensri; Oren Cohen; Peter Cook; Prashant Gupta; Qian Zeng; Rama Ghatti; Scott Parker; Scott Ryan; Scott Sammons; Shatavia Morrison; Stanley Letovsky; Steven Ragan; Suresh Selvaraju; Susan Countryman; Susan Hicks; Suzanne Dale; Thomas Urban; Tim Kuphal; Tricia Zwiefelhofer; Tymeckia Kendall; Victoria Caban Figueroa; Vincent Drouillon; Yvette Unoarumhi
EPI_ISL_13019185, EPI_ISL_13256194	Laboratory of Clinical Microbiology, Virology and Bioemergencies, ASST Fatebenefratelli Sacco - Sacco Hospital	Laboratory of Clinical Microbiology, Virology and Bioemergencies, ASST Fatebenefratelli Sacco - Sacco Hospital	Valeria Micheli
EPI_ISL_11984839, EPI_ISL_11984847, EPI_ISL_12471927, EPI_ISL_12610653, EPI_ISL_12875109, EPI_ISL_12875111, EPI_ISL_12875118, EPI_ISL_12875129, EPI_ISL_12875167, EPI_ISL_12875174, EPI_ISL_12875175, EPI_ISL_12875180, EPI_ISL_12875181, EPI_ISL_13228732, EPI_ISL_13228746, EPI_ISL_13228752, EPI_ISL_13228756, EPI_ISL_13228762, EPI_ISL_13238781, EPI_ISL_13238814, EPI_ISL_13238820, EPI_ISL_13250096, EPI_ISL_13250136, EPI_ISL_13281195			
see above	Lifebrain Covid Labor GmbH	Lifebrain Covid Labor GmbH	Abhishek Mitra; Alexandra Wagner; Anna Edermayr; Felix Valentin Spiegel; Filip Sima; Florian Scharhauser; Hannes Hagen; Kristina Bavrka Kolenc; Lucia Castello; So Jung Han
EPI_ISL_12828082	Limbach - MVZ Humangenetik Ulm	Robert Koch Institute	
EPI_ISL_12675667, EPI_ISL_13247268	Limbach - MVZ Labor Dr. Volkmann & Kollegen	Robert Koch Institute	
EPI_ISL_12675694	Limbach - MVZ Labor Westmecklenburg Schmudlach-Oswald-Kettermann & Kollegen	Robert Koch Institute	
EPI_ISL_13169074	MARIANO MARCOS MEMORIAL HOSPITAL AND MEDICAL CENTER	Philippine Genome Center	Alethea R. de Guzman; Alyssa Joyce E. Telles; Anna Ong-Lim; Arianne A. Zamora; Benedict A. Maralit; Carlo M. Lapid; Celia Carlos; Cynthia P. Saloma; Devon Ray Pacial; Diomedes A. Cariño; Edsel Maurice Salvana; El King D. Morado; Elcid Aaron R. Pangilinan; Eva Maria Cutiongco-de la Paz; Francis A. Tablizo; Henrietta Marie Rodriguez; Jaime C. Montoya; Jan Michael C. Yap; Jarvin E. Nipales; Jo-Hannah S. Llames; John Michael Egana; John Q. Wong; Joshua Gregor A. Dizon; Joshua Jose Endozo; Juan Antonio R. Magalang; Karol Sophia Agape R. Padilla; Kris P. Punayan; Kristina Patriz Dela Cruz; Lindsay Clare D.L. Carandang; Ma. Exanil Plantig; Marc Edsel C. Ayes; Maria Rosario Singh-Vergeire; Maria Sofia L. Yangzon; Marielle M. Gamboa; Marissa Alejandria; Niña Francesca Bustamante; Razel Nikka M. Hao; Renato Jacinto Q. Mantaring; Rianna Patricia S. Cruz; Shiela Mae M. Araiza; Yvonne Valerie Austria; Zipporah Mariebelle R. Enriquez; Zyrel V. Mollejon
EPI_ISL_13086361	MEPHI, Aix Marseille University	MEPHI, Aix Marseille University	Anthony LEVASSEUR
EPI_ISL_13001778, EPI_ISL_13241339	MVZ Dr. Eberhard & Partner Dortmund	Robert Koch Institute	
EPI_ISL_13265071	MVZ Labor Dr. Fenner und Kollegen (Standort Hamburg)	Robert Koch Institute	
EPI_ISL_12845818	Maine Health and Environmental Testing Laboratory	Tewhey Lab, The Jackson Laboratory	Barter, M.; Dewey, H.; H. and Tewhey, R.; Iosue, F.; Lynch, R.; Matluk, N.; Munger
EPI_ISL_13235637, EPI_ISL_13235674	Med. Labor Prof. Schenk Dr. Ansorge & Kollegen	Robert Koch Institute	
EPI_ISL_13202446	Medical Microbiology Unit, Department for Laboratory Medicine, Drammen Hospital, Vestre Viken Health Trust	Norwegian Institute of Public Health, Department of Virology	Atiya R Ali; Debech Nadia; Engebretsen Serina Beate; Garcia Llorente Ignacio; Hilde Elshaug; Hilde Nordby Falkenhaug; Hilde Vollan; Jon Bråte; Kamilla Heddeland Instefjord; Karoline Bragstad; Kathrine Stene-Johansen; Line Victoria Moen; Marie Paulsen Madsen; Olav Hungnes; Pedersen Benedikte Nevjen; Rasmus Riis Kopperud
EPI_ISL_13028110, EPI_ISL_13312801, EPI_ISL_13312812, EPI_ISL_13312813	Microvida	Microvida	Jaco J. Verweij; Joep J. J. M. Stohr; Suzan D. Pas
EPI_ISL_13253591	NZOZ Białostockie Centrum Analiz Medycznych Sp. z o o	1. Academic Center for Pathomorphological and Genetic-Molecular Diagnostics ltd, Bialystok, Poland 2. National Institute of Public Health - National Institute of Hygiene, Warsaw, Poland	Anetta Sulewska; Jacek Nikliński; Janusz Dzięcioł; Joanna Kiśluk; Katarzyna Zacharczuk; Konrad Raczkowski; Magdalena Nowakowska; Małgorzata Sadkowska-Todys; Piotr Karabowicz; Piotr Majewski; Przemysław Biecek. Joanna Reszeć; Radosław Charkiewicz; Tomasz Wołkowicz
EPI_ISL_12208048	National Platform bis COVID ULB-IBC	National Platform bis COVID ULB-IBC	Arnaud Marchant; Benoit Haerlingen; Coralie Henin; Marie-Luce Delforge; Ricardo De Mendonça
EPI_ISL_12646114, EPI_ISL_12647046, EPI_ISL_12647174, EPI_ISL_12647189, EPI_ISL_13094097, EPI_ISL_13273959, EPI_ISL_13273968, EPI_ISL_13273971			
see above	National Public Health Laboratory, National Centre for Infectious Diseases	National Public Health Laboratory, National Centre for Infectious Diseases	BeiBei Chen; Benny Yeo; Chen Shi Ling; Grace Ngan; Jesslin Tan; Lin Cui; Raymond Tzer Pin Lin; Royce Ang; Samuel Loo; Yichen Ding; Zhenyang Zhou
EPI_ISL_13186686, EPI_ISL_13252570, EPI_ISL_13252571, EPI_ISL_13252573, EPI_ISL_13252574, EPI_ISL_13252586, EPI_ISL_13252589, EPI_ISL_13252619, EPI_ISL_13252661, EPI_ISL_13252669, EPI_ISL_13252677, EPI_ISL_13252704, EPI_ISL_13252726, EPI_ISL_13252740, EPI_ISL_13252747, EPI_ISL_13252754, EPI_ISL_13252765, EPI_ISL_13252891, EPI_ISL_13298765, EPI_ISL_13298766, EPI_ISL_13298792, EPI_ISL_13298805, EPI_ISL_13298868, EPI_ISL_13298906			
see above	National Virus Reference Laboratory	National Virus Reference Laboratory	Charlene Bennett; Cillian F De Gascun; Gabriel Gonzalez; Jonathan Dean; Michael Carr; Zoe Yandle
EPI_ISL_13251086	NutriMedlab	National Institute of Public Health	Alexander Nagy; Helena Jirincova; Jan Moskalyk; Jaromira Vecerova; Timotej Suri
EPI_ISL_12983026, EPI_ISL_13180979, EPI_ISL_13181084	Oslo University Hospital, Department of Microbiology	Norwegian Institute of Public Health, Department of Virology	Arvind Yegambaram Meenakshi Sundaram; Cathrine Fladeby; Garcia Llorente Ignacio; Gregor D. Gilfillan; Hilde Elshaug; Hilde Vollan; Jon Bråte; Kamilla Heddeland Instefjord; Karoline Bragstad; Kathrine Stene-Johansen; Line Victoria Moen; Lise Andresen; Mariann Nilsen; Mona Holberg-Petersen; Olav Hungnes; Pedersen Benedikte Nevjen; Pål Marius Bjørnstad; Rasmus Riis Kopperud; Teodora Plamenova Ribarska
EPI_ISL_13291713	PR Public Health Lab	Centers for Disease Control and Prevention Division of Viral Diseases, Pathogen Discovery	Alex Burgin; Ben Rambo-Martin; Clinton Paden; Dakota Howard; Dave Wentworth; Dhwani Batra; Jasmine Padilla; Joseph Madden; Justin Lee; Kristen Knipe; Kristine Lacek; Mark Burroughs; Matthew Schmerer; Meghan Bentz; Mili Sheth; Peter Cook; Sam Shepard; Sarah Nobles; Vivien Dugan; Yvette Unoarumhi
EPI_ISL_12859909, EPI_ISL_13042826, EPI_ISL_13043400, EPI_ISL_13121528, EPI_ISL_13256963	Pandemic Response Lab - NYC	Pandemic Response Lab, R&D	Alex Carpio; Cybill del Castillo; Haiping Hao; Isabel Fernandez Escapa; Jon Laurent; Melissa Hopkins; Michael Hammerling; Simran Chhabria; Simran Gupta; Sol Rey; Steven Chase; Tiara Rivera; William Ward
EPI_ISL_13040764	Pathology Queensland and Forensic Scientific Services	Public Health Virology - Forensic and Scientific Services (PHV-FSS)	Chenwei Wang on behalf of Q-PHIRE Genomics
EPI_ISL_13103406, EPI_ISL_13104100	Provincial Laboratory for Public Health (ProvLab) - North	Alberta Precision Labs (APL)	Buss E; Croxen M; Deo A; Dieu P; Ferrato C; Gill K; Koleva P; Li V; Lloyd C; Lynch T; Ma R; Murphy S; Pabbaraju K; Shideler S; Shokopoles S; Skitsko T; Thayer J; Tipples G; Wong A; Yu C; Zelyas N.
EPI_ISL_12869449, EPI_ISL_12869477, EPI_ISL_12869500, EPI_ISL_13044485, EPI_ISL_13132054, EPI_ISL_13132085, EPI_ISL_13271194, EPI_ISL_13271216, EPI_ISL_13271246			
see above	Public Health Laboratory, Public Health Service Amsterdam, The Netherlands	Department of Medical Microbiology & Infection prevention, Amsterdam University Medical Centers location AMC	Akke Cornelissen; Fokla Zorgdrager; Gini van Rijckevorsel; Janke Schinkel; Jelle Koopsen; Judith den Uil; Marcel Jonges; Matthijs Welkers; Menno de Jong; Menno de Jong and Mariken van der Lubben on behalf of the Amsterdam Regional Genomic epidemiology and Outbreak Surveillance (ARGOS) consortium; Patrick Habermehl; Robin van Houdt; Sebastien Matamoros; Sjoerd Rebers; Sylvia Bruisten; Tjalling Leenstra and Mariken van der Lubben on behalf of the Amsterdam Regional Genomic epidemiology and Outbreak Surveillance (ARGOS) consortium
EPI_ISL_13111253	Public health laboratories Jerusalem	Israel Central Virology laboratory	Danit Sofer; Efrat Dahan Bucris; Ella Mendelson; Evan Nachum; Hagar Morad; Julia Vainer; Maya Davidovich; Michal Mandelboim; Michal Zak; Miranda Geva; Neta Zuckerman; Or Zilbertzan; Oran Erster; Orna Mor; Rona Grossman
EPI_ISL_12582173, EPI_ISL_12583321, EPI_ISL_13001342, EPI_ISL_13028243, EPI_ISL_13048796, EPI_ISL_13066298, EPI_ISL_13066330, EPI_ISL_13066345, EPI_ISL_13107947, EPI_ISL_13108332, EPI_ISL_13131454, EPI_ISL_13201660, EPI_ISL_13201824, EPI_ISL_13244003, EPI_ISL_13244065, EPI_ISL_13244115, EPI_ISL_13244149, EPI_ISL_13244180, EPI_ISL_13244230			
see above	Respiratory Virus Unit, Microbiology Services Colindale, Public Health England	COVID-19 Genomics UK (COG-UK) Consortium	PHE Covid Sequencing Team
EPI_ISL_13242611, EPI_ISL_13242616	Rosalind Franklin Laboratory	Wellcome Sanger Institute for the COVID-19 Genomics UK (COG-UK) Consortium	Cordelia Langford; David K. Jackson; Dominic Kwiatkowski; Donald Fraser; Ewan Harrison; Ian Johnston; Jeffrey Barrett; John Sillitoe on behalf of the Wellcome Sanger Institute COVID-19 Surveillance Team; Rob Howes; Roberto Amato; Sonia Goncalves; Suki Lee; The Rosalind Franklin Laboratory and Alex Alderton
EPI_ISL_13282978	S.C. Laboratorio Analisi, Ospedale di Lavagna, ASL 4 Liguria	U.O. Igiene, Ospedale Policlinico San Martino	Bandettini Roberto; Bruzzone Bianca; De Pace Vanessa; Domnich Alexander; Icardi Giancarlo on behalf of SARS-CoV-2 ITALIAN RESEARCH ENTERPRISE-(SCIRE) Collaborative Group; Orsi Andrea; Randazzo Nadia; Ricucci Valentina; Stefanelli Federica
EPI_ISL_13292599, EPI_ISL_13293034, EPI_ISL_13300919, EPI_ISL_13300921	SARS-CoV-2 Sequencing Castilla y Leon-Spain Consortium	SARS-CoV-2 Sequencing Castilla y Leon-Spain Consortium	Antonio Orduña-Domingo; Carlos Fuster Foz; Carmen Aldea-Mansilla; Carmen Gimeno Crespo; David Abad; Gabriel March Rosello; Gregoria Megías Lobón; Jose María Eiros Bouza; M. Isabel Fernandez-Natal; Marta Dominguez-Gil; Marta Hernandez; María Antonia García Castro; Mª Fe Brezmes-Valdivieso; Noelia Arenal Andrés; Silvia Rojo; Sonsoles Garcinuño Pérez

EPI_ISL_12806962, EPI_ISL_12807012, EPI_ISL_12807046, EPI_ISL_12807091, EPI_ISL_12807162, EPI_ISL_13027401, EPI_ISL_13027571, EPI_ISL_13027577, EPI_ISL_13027612, EPI_ISL_13217440, EPI_ISL_13217505, EPI_ISL_13217673, EPI_ISL_13217703			
see above	SARS-CoV-2 testing team, National Institute of Infectious Diseases	Pathogen Genomics Center, National Institute of Infectious Diseases	Hazuka Y Furihata; Kentaro Itokawa; Makoto Kuroda; Masanori Hashino; Masumichi Saito; Naomi Nojiri; Nozomu Hanaoka; Rina Tanaka; Tsuguto Fujimoto; Tsuyoshi Sekizuka
EPI_ISL_10943953	SMS MEDICAL COLLEGE,JAIPUR	NIV Influenza	Bharti Malhotra; Dinesh parsoya; Farah Deeba; Himanshu sharma; Neha Bhomia; Nita pal; Nivedita Gupta; Pragya D Yadav; Pratibha Sharma; Sudhir Bhandari; Swati Gautam; Varsha Potdar
EPI_ISL_12903141	SYNLAB	University Hospital Brno, CMBG	Bezdicek Matej; Dolejska Monika; Kristyna Dufkova; Lengerova Martina; Svaton Jan
EPI_ISL_11312517, EPI_ISL_12902035, EPI_ISL_13112098	Shamir Medical Center (Asaf Harofe)	Shamir Medical Center (Asaf Harofe)	Abu Hamad Ramzia; Adina Bar Chaim; Alona Frenkel; Anna Vishnevsky; Chen Weiner; Nir Rainy; Patricia Benveniste-Lekovitz; Reut Sorek Abramovich; Yevgeni Yegorov
EPI_ISL_13040526	Sullivan Nicolaides Pathology	Public Health Virology - Forensic and Scientific Services (PHV-FSS)	Chenwei Wang on behalf of Q-PHIRE Genomics
EPI_ISL_13066241, EPI_ISL_13133873	Synlab Eesti OÜ	1. Laboratory of Communicable Diseases (Estonia); 2. Eurofins Genomics Europe Sequencing GmbH	Abroi A.; Avi R.; Dotsenko L.; Epštein J.; Hoidmets D.; Huik K.; Härma M-A.; Jaaniso E.; Kaarna K.; Kallas E.; Koppel I.; Kuzmin I.; Lahesaare A.; Lutsar I.; Metspalu M.; Milani L.; Naaber P.; Niglas H.; Oopkaup O.E.; Pauskar M.; Peterson H.; Päll T.; Ratnik K.; Raudvere U.; Reisberg T.; Sadikova O.; Sepp H.; Shablinskaja A.; Suija H.; Talas U.G.; Truusalu K.
EPI_ISL_13198051	Szpital Wojewodzki im. KardynaLa Stefana Wyszynskiego w Lomży	1. Academic Center for Pathomorphological and Genetic-Molecular Diagnostics ltd, Bialystok, Poland 2. National Institute of Public Health - National Institute of Hygiene, Warsaw, Poland	Anetta Sulewska; Jacek Niklinski; Janusz DzięcioL; Joanna Kiśluk; Katarzyna Zacharczuk; Konrad Raczkowski; MaLgorzata Sadkowska-Todys; Magdalena Nowakowska; Piotr Karabowicz; Piotr Majewski; PrzemysLaw Biecek. Joanna Reszeć; RadosLaw Charkiewicz; Tomasz WoLkowicz
EPI_ISL_12980664, EPI_ISL_13273628	Tokyo Metropolitan Institute of Public Health	Tokyo Metropolitan Institute of Public Health	Ai Suzuki; Akane Negishi; Arisa Amano; Fumi Kasuya; Hirofumi Miyake; Kenji Sadamasu; Kenshirou Kuroki; Mami Nagashima; Maya Isogai; Ryota Kumagai; Sachiko Harada; Takushi Fujiwara
EPI_ISL_12870115, EPI_ISL_13223359, EPI_ISL_13223378	U.O. Microbiologia Laboratorio Unico Centro Servizi - AUSL della Romagna	U.O. Microbiologia, Laboratorio Unico Centro Servizi - AUSL della Romagna	Giorgio Dirani
EPI_ISL_12825301, EPI_ISL_12825326	UMC Groningen, Clinical Virology, Department of Medical Microbiology and Infection Prevention	UMC Groningen, Clinical Virology, Department of Medical Microbiology and Infection Prevention	Alexander Friedrich; Coretta Van Leer-Buter; Erley Lizarazo-Forero; Hubert Niesters; Lilli Gard; Marjolein Knoester; Monika Fliss; Sigrid Rosema; Xuewei Zhou
EPI_ISL_13046845, EPI_ISL_13103105	UW Virology Lab	UW Virology Lab	Alexander Greninger; Hong Xie; Isabel Arnould; Keith R Jerome; Pavitra Roychoudhury; Pooneh Hajian; Sarai Luna; Saraswathi Sathees; Sean Ellis; Seffir T. Wendm; Shah Mohamed Bakhash
EPI_ISL_13018126, EPI_ISL_13018127, EPI_ISL_13018142	Unidad de Investigación Médica de Yucatán (UIMY)	Microbial Genomics Laboratory	; Alejandra García-Gasca; Alejandra Hernández-Terán; Alejandro Sánchez-Flores; Alfredo Herrera-Estrella; Alicia Ocaña-Mondragón; Andreu Comas-García; Angel Gustavo Salas-Lais; Antonio Loza Román; Bernardo Martínez-Miguel; Blanca Taboada; Brenda Irasema Maldonado-Meza; Bruno Gómez-Gil; Carla Ivón Herrera-Najera; Carlos F. Arias; Celia Boukadida; Clara Esperanza Santacruz-Tinoco; Concepción Grajales-Muñiz; Consorcio Mexicano de Vigilancia Genómica (CoViGen-Mex). Authors (in alphabetical order): Julio Elias Alvarado-Yaah; Cristóbal Cháidez-Quiróz; Célida Duque Molina; Célida Martínez- Rodríguez; Daniel Fregoso-Rueda; Daniel Lira Morales; Eduardo Becerril-Vargas; Fernando Fontove-Herrera; Fidencio Mejía-Nepomuceno; Francisco Pulido; Gloria Elena Espinosa-Ayala; Gloria María Molina-Salinas; Gloria Vazquez; Hector Esteban Paz-Juárez; Hector Montoya-Fuentes; Helen Haydee Fernanda Ramírez-Plascencia; Irvin González-López; Jean Pierre González; Jesús Hernández; Joel Armando Vázquez-Pérez.; Jorge Salas-Hernández; José Antonio Enciso-Moreno; José Arturo Martínez-Orozco; José Esteban Muñoz-Medina; José de Jesús Nuñez-Contreras; Juan Bautista Chale-Dzul; Julissa Enciso-Ibarra; Luis Alberto Ochoa-Carrera; Margarita Matías-Florentino; Mario Mújica-Sánchez; Marissa Perez-Garcia; María Guadalupe Santiago-Mauricio; María Guadalupe de Jesús Mireles-Rivera; Nelly Sélem-Mojica; Pavel Isa; Ricardo Ciria Merce; Ricardo Grande; Rosa María Gutiérrez Rios; Santiago Ávila-Ríos; Selene Zárate; Susana Lopez; Verónica Mata-Haro; Victor Eduardo García-Arias; Víctor Hugo Borja-Aburto
EPI_ISL_12983143	University Hospital of Northern Norway, Department for Microbiology and Infectious Disease Control	Norwegian Institute of Public Health, Department of Virology	Atiya R Ali; Debech Nadia; Engebretsen Serina Beate; Garcia Llorente Ignacio; Hilde Elshaug; Hilde Nordby Falkenhaug; Hilde Vollan; Jon Bråte; Kamilla Heddeland Instefjord; Karoline Bragstad; Kathrine Stene-Johansen; Line Victoria Moen; Marie Paulsen Madsen; Olav Hungnes; Pedersen Benedikte Nevjen; Rasmus Riis Kopperud
EPI_ISL_13182714	Vestfold Hospital, Toensberg, Department of Microbiology	Norwegian Institute of Public Health, Department of Virology	Atiya R Ali; Debech Nadia; Engebretsen Serina Beate; Garcia Llorente Ignacio; Hilde Elshaug; Hilde Nordby Falkenhaug; Hilde Vollan; Jon Bråte; Kamilla Heddeland Instefjord; Karoline Bragstad; Kathrine Stene-Johansen; Line Victoria Moen; Marie Paulsen Madsen; Olav Hungnes; Pedersen Benedikte Nevjen; Rasmus Riis Kopperud
EPI_ISL_12737315, EPI_ISL_13133561	ZOL	Jessa	Laura Vanstraelen et al. on behalf of the Jessa_cmdLab; Rita Smets et al. on behalf of the Jessa_cmdLab
EPI_ISL_12879758	ZOTZ KLIMAS MVZ Düsseldorf-Centrum GbR ÜBAG für Labormedizin, Genetik, Zytologie, Pathologie	Center of Medical Microbiology, Virology, and Hospital Hygiene, University of Duesseldorf	Alexander Dilthey; Andreas Walker; Daniel Strelow; Jessica Nicolai; Jörg Timm; Katrin Hoffmann; Klaus Pfeffer; Lisanna Hülse; Malte Kohns Vasconcelos; Maximilian Damagnez; Nadine Lübke; Patrick Finzer; Rainer Zotz; Tobias Wienemann; Torsten Houwaart
EPI_ISL_13238014, EPI_ISL_13268353	labopart - Medizinische Laboratorien Dresden	Robert Koch Institute	
