# Supplementary material for: Tracing the international arrivals of SARS-CoV-2 Omicron variants after Aotearoa New Zealand reopened its border
Source: Nat Commun. 2022 Oct 29;13:6484. doi: 10.1038/s41467-022-34186-9 (PMC9617600; doi:10.1038/s41467-022-34186-9)
Supplement: Supplementary file 6 — Supplementary Data 4 [file 41467_2022_34186_MOESM6_ESM.pdf]

We gratefully acknowledge the following Authors from the Originating laboratories responsible for obtaining the specimens, as well as the Submitting laboratories where the genome data were generated and shared via GISAID, on which this research is based.

All Submitters of data may be contacted directly via www.gisaid.org

Authors are sorted alphabetically.

Accession ID	Originating Laboratory	Submitting Laboratory	Authors
EPI_ISL_12177518, EPI_ISL_12570911	A.O. PERUGIA	A.O. PERUGIA	Bicchieraro G; Bondi P; Camilloni B; Cappelletti E; Ciurnelli R; Lepri E; Lucheroni F; Mencacci A; Spaccapelo R
EPI_ISL_12838823	ALIFE HEALTH	Instituto Nacional de Salud-Dirección de Investigación en Salud Pública	Beatriz de Arco; Carlos Franco-Muñoz; Diego A. Álvarez-Díaz; Diego Andrés Prada; Dioselina Peláez-Carvajal; Gerardo Santamaría; Héctor Alejandro Ruiz-Moreno; Jhonnatan Reales-González; Jorge Rivera; Julián Naizaque; Katherine Laiton-Donato; Marcela Mercado-Reyes.; Martha Lucia Ospina Martínez; María T. Herrera-Sepúlveda; Paola Rojas-Estevez; Sheryll Corchuelo; Tatiana Cobos
EPI_ISL_13059818, EPI_ISL_13059823, EPI_ISL_13059835	ALIFE HEALTH	Instituto de Investigación de Recursos Biológicos Alexander von Humboldt	Daniel Martínez Vargas; Mailyn Gonzalez; Nicolás D. Franco-Sierra; Vanessa Otero-Jiménez
EPI_ISL_12832931	AREA DE SALUD BARVA	Inciensa, Instituto Costarricense de Investigación y Enseñanza en Nutrición y Salud	Adriana Godínez; Claudio Soto-Garita; Estela Cordero; Francisco Duarte; Gabriel Morales; Hebleen Porras; José Luis Vargas; Mariela Gutiérrez; Melany Calderón; Natalia Bonilla & Sharon Peñaranda Chanto; Sofia Herrera
EPI_ISL_13109480	AREA DE SALUD COTO BRUS	Inciensa, Instituto Costarricense de Investigación y Enseñanza en Nutrición y Salud	Adriana Godínez; Claudio Soto-Garita; Estela Cordero; Francisco Duarte; Gabriel Morales & Natalia Bonilla; Hebleen Porras; José Luis Vargas; Mariela Gutiérrez; Melany Calderón; Sofia Herrera
EPI_ISL_12935269	AREA DE SALUD SANTA CRUZ	Inciensa, Instituto Costarricense de Investigación y Enseñanza en Nutrición y Salud	Adriana Godínez; Claudio Soto-Garita; Estela Cordero; Francisco Duarte; Gabriel Morales; Hebleen Porras; José Luis Vargas; Mariela Gutiérrez; Melany Calderón; Natalia Bonilla & María José Villegas Bermúdez; Sofia Herrera
EPI_ISL_12784048, EPI_ISL_12784049	AZ St.Jan Brugge-Oostende	AZ St.Jan Brugge-Oostende	Jorn Hellemans; Marie Madeleine Chabert-Consen; Marijke Reynders; Melissa Provoost; Merijn Vanhee; Sofie Mahboob
EPI_ISL_9829939, EPI_ISL_9829944, EPI_ISL_9829952, EPI_ISL_9829955, EPI_ISL_9829964, EPI_ISL_10104461, EPI_ISL_10407059, EPI_ISL_10407068, EPI_ISL_10993984, EPI_ISL_10993999, EPI_ISL_10994012, EPI_ISL_10994016, EPI_ISL_10994033, EPI_ISL_10994034, EPI_ISL_10994039			
see above	Allergy, Immunology and Cell Biology Unit (AICBU)	Allergy, Immunology and Cell Biology Unit (AICBU)	Ayesha Wijesinghe; Chandima Jeewandara; Deshni Jayathilaka; Dinuka Ariyaratne; Diyanath Ranasinghe; Dumni Guasinghe; Farha Bary; Gathsaurie Neelika Malavige; Heshan Kuruppu; Tibutius Thanesh
EPI_ISL_13337990	Area De Salud Colorado	Inciensa, Instituto Costarricense de Investigación y Enseñanza en Nutrición y Salud	Adriana Godínez; Claudio Soto-Garita; Estela Cordero; Francisco Duarte; Gabriel Morales; Hebleen Porras; José Luis Vargas; Mariela Gutiérrez; Melany Calderón; Natalia Bonilla & Shirley Rojas Ramírez; Sofia Herrera
EPI_ISL_13337847	Area de salud corredores	Inciensa, Instituto Costarricense de Investigación y Enseñanza en Nutrición y Salud	Adriana Godínez; Claudio Soto-Garita; Estela Cordero; Francisco Duarte; Gabriel Morales; Hebleen Porras; José Luis Vargas; Mariela Gutiérrez; Melany Calderón; Natalia Bonilla & Gabriela Marchena Jimenez; Sofia Herrera
EPI_ISL_13337834	Area de salud la cruz	Inciensa, Instituto Costarricense de Investigación y Enseñanza en Nutrición y Salud	Adriana Godínez; Claudio Soto-Garita; Estela Cordero; Francisco Duarte; Gabriel Morales & Natalia Bonilla; Hebleen Porras; José Luis Vargas; Mariela Gutiérrez; Melany Calderón; Sofia Herrera
EPI_ISL_11852963	Associazione Donatori di Sangue Teramo	Istituto Zooprofilattico Sperimentale dell'Abruzzo e Molise "G. Caporale"	Ancora M; Calistri P; Cammà C; Caporale M; Curini V; Delli Compagni E; Di Domenico M; Di Lollo Valeria; Di Pasquale A; Lorusso A; Mangone I; Marcacci M; Puglia I; Rinaldi A; Savini G; Scialabba S
EPI_ISL_11073123, EPI_ISL_12024284, EPI_ISL_12313884, EPI_ISL_12650435, EPI_ISL_12727797	Austrian Agency for Health and Food Safety (AGES)	Bergthaler laboratory, CeMM Research Center for Molecular Medicine of the Austrian Academy of Sciences	Alberto Alises; Andreas Bergthaler; Anna Schedl; Bekir Erguner; Benedikt Agerer; Christoph Bock; Fabian Amman; Jan Laine; Lukas Endler; Martin Senekowitsch; Matthew Thornton; Michael Schuster; Michelle Chan; Petr Triska; Thomas Penz
EPI_ISL_11269099	Aversi Clinic	Department for Virology, Molecular Biology and Genome Research, R. G. Lugar Center for Public Health Research, National Center for Disease Control and Public Health (NCDC) of Georgia.	Adam Kotorashvili; Amiran Gamkrelidze.; Ana Papkiauri; Ann Machablishvili; Anna Kasradze; Davit Tsaguria; Ekaterine Khmaladze; Ekaterine Zangaladze; Ekaterine Zhghenti; Giorgi Gogoladze; Giorgi Tomashvili; Gvantsa Brachveli; Gvantsa Chanturia; Irma Burjanadze; Ketevan Sidamonidze; Khatuna Zakhashvili; Lela Sabadze; Lela Urushadze; Magda Dgebuadze; Maia Alkhazashvili; Mari Gavashelidze; Mariam Zakalashvili; Marine Murtskhvaladze; Meri Pantsulaia; Nato Kotaria; Nino Berishvili; Paata Imnadze; Roena Sukhiashvili; Salome Javashvili; Tamar Jashiashvili; Tata Imnadze; Tea Tevdoradze
EPI_ISL_12946141, EPI_ISL_13100329	Basurto University Hospital: Clinical Microbiology Laboratory	Basurto University Hospital: Clinical Microbiology Laboratory	Estibaliz Ugalde Zarraga; José Luis Díaz de Tuesta del Arco; Mikel Urrutikoetxea-Gutiérrez; Mª Carmen Nieto Toboso
EPI_ISL_10969316	Biomedical Centre Martin, Jessenius Faculty of Medicine in Martin, Comenius University	Laboratory of Genomics and Bioinformatics, Comenius University Science Park	Andrea Hornáková; Anna Kaliňáková; Barbora Kotvasová; Diana Rusňáková; Dušan Loderer; Ivana Kašubová; Jaroslav Budiš; Katarína Janíková; Lucia Ševčíková; Marián Grendár; Miroslav Böhmer; Mária Škereňová; Pavol Mišenko; Terézia Vrabľová; Tomáš Szemes
EPI_ISL_10442995	Botswana Harvard HIV Reference Laboratory	Botswana Harvard HIV Reference Laboratory	Boitumelo Zuze; Dorcas Maruapula; Joseph Makhema; Kgomotso Moruisi; Legodile Kooepile; Mosepele Mosepele; Mphaphi B. Mbulawa; Ontlametse T. Bareng; Pamela Smith-Lawrence; Roger Shapiro; Sefetogi Ramaologa; Shahin Lockman; Shirley Johane; Sikhulile Moyo; Simani Gaseitsiwe; Thongbotho Mphoyakgosi; Wonderful T. Choga
EPI_ISL_13168976, EPI_ISL_13168981	CENTRO DE ESPECIALIDADES DERMATOLÓGICAS	Laboratorio Central de Salud Pública	Analía Rojas; Andrea Gómez de la Fuente; Cynthia Vazquez; César Cantero; César Ojeda; Emmanuel Céspedes; Fátima Fleitas; Ivana Fernández; Juan Torales; Julio Barrios; Maria Liz Gamarra; María Jose Duarte; Sandra González; Shirley Villalba; Tania Alfonzo; Wilson Benítez.
EPI_ISL_12285404	CERBALLIANCE ARTOIS	CERBA HealthCare	Bénédicte Roquebert; Laura Verdurme; Mathilde Roussel; Sabine Trombert; Stéphanie Haim-Boukobza
EPI_ISL_9550429	COVID-19 Detection Lab, Chattogram Veterinary and Animal Sciences University	COVID-19 Detection Lab, Chattogram Veterinary and Animal Sciences University	56. Tanvir Ahmad Nizami; Barna Goswami; Eaftekhar Ahmed Rana; Goutam Buddha Das; Iffat Jahan; Md. Ahasan Habib; Md. Murshed Hasan Sarkar; Md. Salim Khan; Md. Sirazul Islam; Mohammad Mohi Uddin; Paritosh Kumar Biswas; Pronesh Dutta; Sanjana Fatema Chowdhury; Shahina Akter; Sharmin Chowdhury; Showti Raheel Nase; Tanjina Akhter Banu; Tridip Das
EPI_ISL_12279304, EPI_ISL_12428361, EPI_ISL_12693978	COVID-19 Diagnostic Lab, BADAS	Genomic Research Laboratory, BSMMU	Laila Anjuman Banu; Mahmud Hossain; Md.Sharfuddin Ahmed; Nahid Azmin; Zahid Hassan
EPI_ISL_12607716, EPI_ISL_12607741, EPI_ISL_12607756, EPI_ISL_12607779, EPI_ISL_12607813	CPHL,MOH,EGYPT	CPHL	Abd Monaem Adel; Amel nagiub; Amer Sayed; Amr Kandil.; Dalia Ramadan; Galal Mahmoud; Marwa Abd EL fatah; Marwa Saleh; Mohamed Hassany; Mohamed Kamal; Nancy el guindy; Ramy Galal; Rasha Zaater; Shymaa s. Ahmed; Wael H. Roshdy; salma sayed
EPI_ISL_13134340	Cantacuzino National Military-Medical Institute, Viral Respiratory Infections Laboratory	Cantacuzino National Military-Medical Institute, Viral Respiratory Infections Laboratory	Catalina Pascu; Luiza Ustea; Mihaela Lazar; Mihaela Oprea; Sorin Dinu; Vitencu Oana
EPI_ISL_12024394, EPI_ISL_12144619, EPI_ISL_12896424	Center for Virology, Medical University of Vienna	Bergthaler laboratory, CeMM Research Center for Molecular Medicine of the Austrian Academy of Sciences	Alberto Alises; Andreas Bergthaler; Anna Schedl; Christoph Bock; Fabian Amman; Lukas Endler; Matthew Thornton; Michael Schuster; Michelle Chan; Petr Triska
EPI_ISL_11698098, EPI_ISL_11698102	Center of Excellence in Clinical Virology, Faculty of Medicine, Chulalongkorn University	Center of Excellence in Clinical Virology, Faculty of Medicine, Chulalongkorn University	Amornmas Kongkliang; Jira Chansaenroj; Jiratchaya Puenpa; Kamolthip Atsawawaranunt; Nutsada Saengdao; Oraphan Mayuramart; Pakkaporn Panwijitkul; Pattaraporn Nimsamer; Patthaya Rattanakomol; Sunchai Payungporn; Suwanna Petto; Vichan Pawun; Vorthon Sawaswong; Yong Poovorawan
EPI_ISL_13332960, EPI_ISL_13333002, EPI_ISL_13333011, EPI_ISL_13333023, EPI_ISL_13333027, EPI_ISL_13333037	Centers for Disease Control and Prevention, Dengue Branch	Centers for Disease Control and Prevention, Dengue Branch	Betzabel Flores; Gabriela Paz-Bailey; Gilberto A. Santiago; Glenda Gonzalez; Jorge L. Munoz-Jordan; Keyla Charriez
EPI_ISL_13003004	Centre Hospitalier Universitaire Clermont-Ferrand	CHU Clermont-Ferrand, service de virologie	Bisseux Maxime; Combes Patricia; Henquell Cecile; Mirand Audrey
EPI_ISL_13350769	Centro Medico La Costa	Laboratorio Central de Salud Pública	Analía Rojas; Andrea Gómez de la Fuente; Cynthia Vazquez; César Cantero; César Ojeda; Emmanuel Céspedes; Fátima Fleitas; Ivana Fernández; Juan Torales; Julio Barrios; Maria Liz Gamarra; María Jose Duarte; Sandra González; Shirley Villalba; Tania Alfonzo; Wilson Benítez.

EPI_ISL_12934866	Centro de Investigación Biomédica del Noreste (CIBIN)	Instituto de Biotecnología de la UNAM	; Alejandra García-Gasca; Alejandra Hernández-Terán; Alejandro Sánchez-Flores; Alfredo Herrera-Estrella; Alicia Ocaña-Mondragón; Andreu Comas-García; Angel Gustavo Salas-Lais; Antonio Loza Román; Bernardo Martínez-Miguel; Blanca Taboada; Brenda Irasema Maldonado-Meza; Bruno Gómez-Gil; Carla Ivón Herrera-Najera; Carlos F. Arias; Celia Boukadida; Clara Esperanza Santacruz-Tinoco; Concepción Grajales-Muñiz; Consorcio Mexicano de Vigilancia Genómica (CoViGen-Mex). Authors (in alphabetical order): Julio Elias Alvarado-Yaah; Cristóbal Cháidez-Quiróz; Célida Duque Molina; Célida Martínez- Rodríguez; Daniel Fregoso-Rueda; Daniel Lira Morales; Eduardo Becerril-Vargas; Fernando Fontove-Herrera; Fidencio Mejía-Nepomuceno; Francisco Pulido; Gloria Elena Espinosa-Ayala; Gloria María Molina-Salinas; Gloria Vazquez; Hector Esteban Paz-Juárez; Hector Montoya-Fuentes; Helen Haydee Fernanda Ramírez-Plascencia; Irvin González-López; Jean Pierre González; Jesús Hernández; Joel Armando Vázquez-Pérez).; Jorge Salas-Hernández; José Antonio Enciso-Moreno; José Arturo Martínez-Orozco; José Esteban Muñoz-Medina; José de Jesús Nuñez-Contreras; Juan Bautista Chale-Dzul; Julissa Enciso-Ibarra; Kathia Elizabeth Tapia-Diaz; Luis Alberto Ochoa-Carrera; Margarita Matías-Florentino; Mario Mújica-Sánchez; Marissa Perez-Garcia; María Guadalupe Santiago-Mauricio; María Guadalupe de Jesús Mireles-Rivera; Nelly Sélem-Mojica; Pavel Isa; Ricardo Ciria Merce; Ricardo Grande; Rosa María Gutiérrez Rios; Santiago Ávila-Ríos; Selene Zárate; Susana Lopez; Verónica Mata-Haro; Victor Eduardo García-Arias; Víctor Hugo Borja-Aburto
EPI_ISL_10993343	Cianjur Public Health Laboratory	West Java Health Laboratory; School of Life Sciences and Technology, Institut Teknologi Bandung	Azzania Fibriani; Cut Nur Cinthia Alamanda; Ema Rahmawati; Karimatu Khoirunnisa; Miftahul Faridl; Rifky Waluyajati Rachman; Rini Robiani; Ryan Bayusantika Ristandi
EPI_ISL_11832515, EPI_ISL_13135431, EPI_ISL_13135599	Clinical Emergency County Hospital, Craiova	Cantacuzino National Military-Medical Institute, Viral Respiratory Infections Laboratory	Carmen Cherciu; Catalina Pascu; Luiza Ustea; Mihaela Lazar; Mihaela Oprea; Pascu Catalina; Sorin Dinu; Vitencu Oana
EPI_ISL_12415946	Clinical Hospital of Infectious Diseases, Cluj-Napoca	Cantacuzino National Military-Medical Institute, Viral Respiratory Infections Laboratory	Catalina Pascu; Luiza Ustea; Mihaela Lazar; Mihaela Oprea; Sorin Dinu; Vitencu Oana
EPI_ISL_13017266	Cliniques universitaires Saint-Luc	UCLouvain/IREC/MBLG-CTMA	Benoit Kabamba Mukadi; Bertrand Bearzatto; Jean-Luc Gala; Valentin Coste
EPI_ISL_12666259	Colsanitas Keralty	Instituto de Investigación de Recursos Biológicos Alexander von Humboldt	Daniel Martínez Vargas; Mailyn Gonzalez; Nicolás D. Franco-Sierra
EPI_ISL_11149531, EPI_ISL_11149533, EPI_ISL_11149552, EPI_ISL_11149643	DSMRC	Defence Services Medical Research Centre (DSMRC)	Aung Myo Nyi; Aung Sitt Hmue; Khine Zaw Oo; Ko Ko Lwin; Kyaw Moe Htike; Kyaw Zawl Lin; Kyee Myint; Phyo Kyaw Aung; Thet Wai Oo; Zaw Win Htun
EPI_ISL_11027040	Department for Virology, Molecular Biology and Genome Research, R. G. Lugar Center for Public Health Research, National Center for Disease Control and Public Health (NCDC) of Georgia.	Department for Virology, Molecular Biology and Genome Research, R. G. Lugar Center for Public Health Research, National Center for Disease Control and Public Health (NCDC) of Georgia.	Adam Kotorashvili; Amiran Gamkrelidze.; Ana Papkiauri; Ann Machablishvili; Anna Kasradze; Davit Tsaguria; Ekaterine Khmaladze; Ekaterine Zangaladze; Ekaterine Zhghenti; Giorgi Gogoladze; Giorgi Tomashvili; Gvantsa Brachveli; Gvantsa Chanturia; Irma Burjanadze; Ketevan Sidamonidze; Khatuna Zakhashvili; Lela Sabadze; Lela Urushadze; Magda Dgebuadze; Maia Alkhazashvili; Mari Gavashelidze; Mariam Zakalashvili; Marine Murtskhvaladze; Meri Pantsulaia; Nato Kotaria; Nino Berishvili; Paata Imnadze; Roena Sukhiashvili; Salome Javashvili; Tamar Jashiashvili; Tata Imnadze; Tea Tevdoradze
EPI_ISL_13352745, EPI_ISL_13352747, EPI_ISL_13352772, EPI_ISL_13352778	Department of Clinical Laboratory	Department of Clinical Laboratory	Jin Xu; Mei Zeng; Yuanyun Ao
EPI_ISL_12417480, EPI_ISL_12417518, EPI_ISL_12417533, EPI_ISL_12417571, EPI_ISL_12417581, EPI_ISL_12417591, EPI_ISL_12417780, EPI_ISL_12417944			
see above	Department of Health Technology and Informatics, The Hong Kong Polytechnic University	Department of Health Technology and Informatics, The Hong Kong Polytechnic University	Alan Ka-Lun Wu; Alex Yat-Man Ho; Barry Kin-Chung Wong; Chloe Toi-Mei Chan; David Ho-Keung Shum; Gilman Kit-Hang Siu; Hiu-Yin Lao; Ivan Tak-Fai Wong; Jake Siu-Lun Leung; Kam-Tong Yip; Kenneth Siu-Sing Leung; Kingsley King-Gee Tam; Kitty Sau-Chun Fung; Kristine Luk; Lam-Kwong Lee; Miranda Chong-Yee Yau; Sandy Ka-Yee Chau; Shea Ping Yip; Tak-Lun Que; Timothy Ting-Leung Ng; Wing Cheong Yam; Wing-Hei Lo; Wing-Kin To; Yvette Wai-Man Lai
EPI_ISL_13088333	Department of Laboratory Services, National Virology Reference Laboratory	Clinical Molecular Diagnostic Laboratory For Infectious Disease, Department of Laboratory Services, Microbial Genomic Services	Amal Nabihah Ahmad; Faezah Fariha Abd Latif; Haziq Momin; Izzati Azhar; Nor Azian Hafneh; Nur Amirah Ibarahim; Zainun Zaini
EPI_ISL_11804454, EPI_ISL_11804489, EPI_ISL_11804580, EPI_ISL_11804613, EPI_ISL_12702064	Department of Microbiology, National Institute of Public Health of Kosovo	Department of Microbiology, National Institute of Public Health of Kosovo	Aferdita Kuqi-Hyseni; Blendi Jerliu; Donjeta Hajdari; Nazmi Mehmeti; Pranvera Abazi; Robert Ramadani; Xhevat Jakupi; Zana Deva
EPI_ISL_9856176, EPI_ISL_10774006, EPI_ISL_10774010	Department of Pathology and Laboratory Medicine, AKUH Laboratories, Karachi, Pakistan	Department of Pathology and Laboratory Medicine, AKUH Laboratories, Karachi, Pakistan	A. Kanji; A. Nasir; A. Samreen; A.R. Bukhari; AKU; Health Security Partners; Islamabad; J. Ashraf; P.Thielen; Pakistan; R. Hasan; U.B. Aamir; USA; USA); University Research Council Grant; WHO; Z. AzizUllah; Z. Hasan (Funding support NIH Fogarty Training Grant
EPI_ISL_10337127, EPI_ISL_10337406	Department of Virology, National Veterinary Research Institute	Department of Omics Analysis, National Veterinary Research Institute	Bomba Arkadiusz; Domanska-Blicharz Katarzyna; Iwan Ewelina; Katarzyna Tluscik; Lisowska Anna; Niemczuk Krzysztof; Opolska Justyna; Orlowska Anna; Rola Jerzy; Smreczak Marcin; Trebas Pawel
EPI_ISL_8799568, EPI_ISL_8799569, EPI_ISL_8932859, EPI_ISL_8933054	Dhulikhel Hospital, Kathmandu University Hospital	Molecular and Genomics Research Lab, Dhulikhel Hospital, Kathmandu University Hospital	Dipesh Tamrakar; Nishan Katuwal; Rajeev Shrestha; Surendra Kumar Madhup
EPI_ISL_11750210	Diagnostyka Sp. z o.o. Gdańsk	Wojewódzka Stacja Sanitarno-Epidemiologiczna w Gdańsku	Barbara Skórczewska; Barbara Zawadzka; Gabriela Rutkowska; Marta Piszczyk
EPI_ISL_12750355, EPI_ISL_12899516	Dr. Yoshitaka Tamura Department of Clinical Laboratory, Osaka Habikino Medical Center	Osaka Women's and Children's Hospital Department of Developmental Medicine, Research Institute, Osaka Women's and Children's Hospital	Itaru Yanagihara; Yukiko Nakura
EPI_ISL_10875757, EPI_ISL_10875764, EPI_ISL_10875767, EPI_ISL_10875777, EPI_ISL_10875781, EPI_ISL_10875786	EWARN's Lab	Ministry of Health Turkey	Yasir Elferruh
EPI_ISL_12871556	Fasa University of Medical Sciences	National Influenza Center	A Nejati; Adel Abedi; J Yavarian; K Sadeghi; Mrzieh Faraji-Zonouzand T Mokhtari Azad; NZ Shafiei Jandaghi; Nastaran Ghavami; Sevrin Zadheidar; V Salimi
EPI_ISL_12832821	Fusco Lab	Maness Lab	Arnaud Drouin; Dahlene Fusco; Joshua Katz; Lori Rowe; Matthew Moreida; Nicholas Maness
EPI_ISL_8605841	GENOME CENTER, Jashore University of Science and Technology.	GENOME CENTER, Jashore University of Science and Technology.	Hassan M. Al-Emran; Iqbal Kabir Jahid; Md. Ali Ahasan Setu; Md. Anwar Hossain; Md. Rasel Parvez; Md. Shaminur Rahman Prosanto Kumar Das; Md. Shazid Hasan; Shovon Lal Sarkar; Toukir Ahammed
EPI_ISL_11018136, EPI_ISL_11050899	Genome Analysis Center, Yamanashi Central Hospital	Genome Analysis Center, Yamanashi Central Hospital	Yosuke Hirotsu; Yuki Nagakubo; Yuki Nagakubo and Yosuke Hirotsu
EPI_ISL_13169680	HE-HOSPITAL DEL CANCER	Laboratorio Central de Salud Pública	Analía Rojas; Andrea Gómez de la Fuente; Cynthia Vazquez; César Cantero; César Ojeda; Emmanuel Céspedes; Fátima Fleitas; Ivana Fernández; Juan Torales; Julio Barrios; Maria Liz Gamarra; María Jose Duarte; Sandra González; Shirley Villalba; Tania Alfonzo; Wilson Benítez.
EPI_ISL_12172435	HOSPITAL DR. TOMAS CASAS CASAJUS	Inciensa, Instituto Costarricense de Investigación y Enseñanza en Nutrición y Salud	Adriana Godínez; Claudio Soto-Garita; Estela Cordero; Francisco Duarte; Gabriel Morales & Antony Orozco Barquero; Hebleen Porras; José Luis Vargas; Mariela Gutiérrez; Melany Calderón; Sofia Herrera
EPI_ISL_12838819	HOSPITAL UNIVERSITARIO FUNDACION SANTA FE DE BOGOTA	Instituto Nacional de Salud-Dirección de Investigación en Salud Pública	Beatriz de Arco; Carlos Franco-Muñoz; Diego A. Álvarez-Díaz; Diego Andrés Prada; Dioselina Peláez-Carvajal; Gerardo Santamaría; Héctor Alejandro Ruiz-Moreno; Jhonnatan Reales-González; Jorge Rivera; Julián Naizaque; Katherine Laiton-Donato; Marcela Mercado-Reyes.; Martha Lucia Ospina Martínez; María T. Herrera-Sepúlveda; Paola Rojas-Estevez; Sheryll Corchuelo; Tatiana Cobos
EPI_ISL_11006098	Ha Nam	Bachmai Hospital	Doanh Khuong; Dung Le; Lan Pham; Linh Le; Nga Pham; Ngan Le; Phuong Truong; Van Vu; Vuong Bui
EPI_ISL_13025766	Hoa Binh General Hospital	Bachmai Hospital	Doanh Khuong; Dung Le; Lan Pham; Linh Le; Nga Pham; Ngan Le; Phuong Truong; Van Vu; Vuong Bui
EPI_ISL_12157760, EPI_ISL_12157762	Hopital	National Reference Center for Viruses of Respiratory Infections, Institut Pasteur, Paris	Angela Brisebarre; Camille Capel; Christophe Malabat; Corinne Maufrais; Etienne Simon-Lorière; Frédéric Lemoine; Julien Fumey; Laurence FAGOUR; Louise Lefrançois; Marion Barbet; Maud Vanpeene; Méline Bizard; Slim El Khiari; Sylvie Behillil; Sylvie Van der Werf; Vincent Enouf
EPI_ISL_12717510	Hospital Universitario Marqués de Valdecilla	Servicio de Microbiologia. Hospital Universitario Marqués de Valdecilla	Elena Bolado Conde; Javier Freire Salinas; Jesús Agüero Balbín; Jesús Rodríguez Lozano; Jorge Calvo Montes; Mónica Gozalo Margüello; Sergio Garcia Fernandez
EPI_ISL_13337845	Hospital de ciudad neily	Inciensa, Instituto Costarricense de Investigación y Enseñanza en	Adriana Godínez; Claudio Soto-Garita; Estela Cordero; Francisco Duarte; Gabriel Morales; Hebleen Porras; José Luis Vargas; Mariela Gutiérrez; Melany Calderón; Natalia Bonilla & Gabriela Marchena Jimenez; Sofia Herrera

		Nutrición y Salud	
EPI_ISL_13350774	Hospital del Cancer-INCAN	Laboratorio Central de Salud Pública	Analía Rojas; Andrea Gómez de la Fuente; Cynthia Vazquez; César Cantero; César Ojeda; Emmanuel Céspedes; Fátima Fleitas; Ivana Fernández; Juan Torales; Julio Barrios; Maria Liz Gamarra; María Jose Duarte; Sandra González; Shirley Villalba; Tania Alfonzo; Wilson Benítez.
EPI_ISL_12637345	ICMT APARTADO	Laboratorio Departamental de Salud Publica de Antioquia	Ana Victoria Valencia Duarte; Cristian Arbey Velarde Hoyos; Gloria Isabel Escobar; Idabely Betancur Ortiz; Juan Pablo Isaza Agudelo
EPI_ISL_13107536	INCMNSZ	Instituto Nacional de Medicina Genomica	Cedro-Tanda A; Cruz-Islas Jazmin; Escobar-Arrazola MA; Garnica-Lopez Dora; Herrera-Montalvo LA.; Hidalgo-Miranda A; Mendoza-Vargas A; Ramirez-Vega O; Rangel-DeLeon D; Reyes-Grajeda JP; Rubio-Alvarado PV; Yair Alfaro-Mora
EPI_ISL_9738913, EPI_ISL_10994857, EPI_ISL_10994862	Indira Gandhi Memorial Hospital	Indira Gandhi Memorial Hospital	D. Fathmath Nazla Rafeeq; Dr. Ibrahim Afzal; Mr. Ibrahim Nishan Ahmed; Ms. Aishath Shuhudha; Ms. Aminath Shazleena Abdul Rahman; Ms. Soafy Mohamed
EPI_ISL_11732770	Institut für Lebensmittelsicherheit, Veterinärmedizin, Umwelt	Institut für Lebensmittelsicherheit, Veterinärmedizin, Umwelt	Gunther Vogl
EPI_ISL_11878815, EPI_ISL_12650139, EPI_ISL_12896521	Institute for Water Quality and Resource Management, Technical University Vienna	Bergthaler laboratory, CeMM Research Center for Molecular Medicine of the Austrian Academy of Sciences	Alberto Alises; Andreas Bergthaler; Anna Schedl; Bekir Erguner; Benedikt Agerer; Christoph Bock; Fabian Amman; Jan Laine; Lukas Endler; Martin Senekowitsch; Matthew Thornton; Michael Schuster; Michelle Chan; Petr Triska; Thomas Penz
EPI_ISL_12024316, EPI_ISL_12024321, EPI_ISL_12727976	Institute of Legal Medicine, Medical University of Innsbruck	Bergthaler laboratory, CeMM Research Center for Molecular Medicine of the Austrian Academy of Sciences	Alberto Alises; Andreas Bergthaler; Anna Schedl; Christoph Bock; Fabian Amman; Lukas Endler; Matthew Thornton; Michael Schuster; Michelle Chan; Petr Triska
EPI_ISL_12153603, EPI_ISL_12153605, EPI_ISL_12153606, EPI_ISL_12153607, EPI_ISL_12153613, EPI_ISL_12153617, EPI_ISL_12153618, EPI_ISL_12153620, EPI_ISL_12153625			
see above	Institute of Medical Microbiology and Hospital Hygiene	Institute of Medical Microbiology and Hospital Hygiene	Aljoscha Tersteegen; Prof. Dr. Achim Kaasch
EPI_ISL_12156899, EPI_ISL_12400912	Institute of Molecular and Translational Medicine / Laboratory of Experimental Medicine, Faculty of Medicine and Dentistry, Palacky University	Institute of Molecular and Translational Medicine / Laboratory of Experimental Medicine, Faculty of Medicine and Dentistry, Palacky University	Barbora Blumová; Hana Jaworek; Kateřina Kubáňová; Marián Hajdúch; Rastislav Slavkovský; Vladimíra Koudeláková
EPI_ISL_13107322	Instituto Nacional de Medicina Genomica	Instituto Nacional de Medicina Genomica	Cedro-Tanda A; Escobar-Arrazola MA; Garnica-Lopez Dora; Herrera-Montalvo LA.; Hidalgo-Miranda A; Mendoza-Vargas A; Ramirez-Vega O; Rangel-DeLeon D; Reyes-Grajeda JP; Yair Alfaro-Mora
EPI_ISL_11994764, EPI_ISL_11994767, EPI_ISL_11994771, EPI_ISL_11994776, EPI_ISL_11994781	Iressef Genomics lab	IRESSEF	Abdou PADANE; Ambroise AHOUIDI; Aminata DIA; Aminata MBOUP; Astou Gaye GAYE; Barada CISSE; Birahim Piere NDIAYE; Cyrille Diedhiou; Diabou Diagne; Gora LO; Khadim GUEYE; Moustapha MBOW; Nafisatou LEYE; Ndeye Coumba Toure KANE; Papa Alassane DIAW; Samba Ndiour; Seni Ndiaye; Souleymane MBOUP; Yacine DIA
EPI_ISL_12657276	Jurisdiccion Sanitaria No.1	LESP Aguascalientes	Anastacio Palacios-Marmolejo; Antonio Hernandez-Mejia; Brian Muñoz-Gomez; David Hernandez-Wancho; Magally Tristan-Leon; Mirella Paredes-Salas; Miriam Lozano-Gamboa; Monica Ortiz-Palos; Sabel Hernandez-Zavala; Susana Morales-Moran. Brenda Ortiz-Jasso
EPI_ISL_11323578	Kasturba Hospital Molecular Lab	Kasturba hospital WGS	Jayanthi Shastri; Vidushi Chitalia
EPI_ISL_11664731	Kasturba Hospital Molecular Lab	NIV Influenza	Jayanthi Shastri; Vidushi Chitalia
EPI_ISL_9512091	Kasturba Hospital Molecular Lab - INSACOG	Kasturba hospital WGS	Jayanthi Shastri; Vidushi Chitalia
EPI_ISL_12813250, EPI_ISL_12813251	Klinisch Laboratorium ZNA	Klinisch Laboratorium ZNA	Verstrepen et al.
EPI_ISL_13037531	Krajská nemocnice T.Bati, a.s.	Institute of Molecular and Translational Medicine / Laboratory of Experimental Medicine, Faculty of Medicine and Dentistry, Palacky University	Barbora Blumová; Hana Jaworek; Kateřina Kubáňová; Marián Hajdúch; Rastislav Slavkovský; Vladimíra Koudeláková
EPI_ISL_12061332, EPI_ISL_12175820, EPI_ISL_12175862, EPI_ISL_12299416, EPI_ISL_13064574, EPI_ISL_13064624	Kuala Lumpur International Airport	Institute for Medical Research, Infectious Disease Research Centre, National Institutes of Health, Ministry of Health Malaysia	Anasir MI; G.Adypatti NM; Jamaluddin MS; Kalyanasundram J; Kamel K; MatRahim N; Nawi MH; Suib FA; Suppiah J; Thayan R
EPI_ISL_13168949	LAB PRIV CYRLAB	Laboratorio Central de Salud Pública	Analía Rojas; Andrea Gómez de la Fuente; Cynthia Vazquez; César Cantero; César Ojeda; Emmanuel Céspedes; Fátima Fleitas; Ivana Fernández; Juan Torales; Julio Barrios; Maria Liz Gamarra; María Jose Duarte; Sandra González; Shirley Villalba; Tania Alfonzo; Wilson Benítez.
EPI_ISL_11586749	LABORATORIUM BADAŃ KLINICZNYCH WSSE w OPOLU	Wojewódzka Stacja Sanitarno-Epidemiologiczna w Katowicach	Adrian Miara; Agata Chajda; Beata Rozwadowska; Dorota Liberska; Elzbieta Bartkowiak; Hubert Okla; Marta Albertynska; Natalia Kuczera
EPI_ISL_11892333	LBA - SITE CASTELJALOUX	CERBA HealthCare	Bénédicte Roquebert; Laura Verdurme; Mathilde Roussel; Sabine Trombert; Stéphanie Haim-Boukobza
EPI_ISL_12140289	LBBMS BIO POLE ANTILLES	CERBA HealthCare	Bénédicte Roquebert; Laura Verdurme; Mathilde Roussel; Sabine Trombert; Stéphanie Haim-Boukobza
EPI_ISL_9749785	LESP Jalisco	Instituto de Diagnostico y Referencia Epidemiologicos (INDRE)	Abril Rodriguez-Maldonado; Ariadna Medina-Benitez; Claudia Wong-Arambula; Ernesto Ramirez-Gonzalez.; Fernando Gonzalez-Dominguez; Gisela Barrera-Badillo; Irma Lopez-Martinez; Joaquin Quiroz-Mercado; Lucia Hernandez-Rivas; Maribel Gonzalez-Villa; Natividad Cruz-Ortiz; Sergio Rangel-Guerrero; Tatiana Nunez-Garcia; Vanessa Rivero-Arredondo
EPI_ISL_13094338	LOMWRU	LOMWRU/Microbiology Laboratory, Mahosot Hospital	Audrey Dubot-Pérès; Elizabeth Ashley; Elizabeth Batty; Manila Souksavanh; Manivanh Vongsouvath; Matthew T. Robinson; Siribun Panapruksachat
EPI_ISL_9618959	Lab Klinik Utama Karunia	National Institute of Health Research and Development	Arie Ardiansyah Nugraha; Fajar Nur Sulistiyohadi; Hana Apsari Pawestri; Hartanti Dian Ikawati; Kartika Dewi Puspa; Nelly Puspandari; Nur Ika Hariastuti; Putri Widia; Subangkit; Vivi Setiawaty
EPI_ISL_11021578, EPI_ISL_12157642, EPI_ISL_12637726, EPI_ISL_13283347	Labo Analyses Med	National Reference Center for Viruses of Respiratory Infections, Institut Pasteur, Paris	Angela Brisebarre; Camille Capel; Christophe Malabat; Corinne Maufrais; Etienne Simon-Lorière; Frédéric Lemoine; Isabelle SOURON; Julien Fumey; Louise Lefrançois; Marc BIRON; Marie-Hélène GLAUDON LOUVEAU DE LA GUIGNERAYE; Marion Barbet; Maud Vanpeene; Méline Bizard; Nicolas GUIGUE; Slim El Khiari; Sylvie Behillil; Sylvie Van der Werf; Vincent Enouf
EPI_ISL_11450489, EPI_ISL_12349816, EPI_ISL_12349820, EPI_ISL_12349827	Laboratori d'anàlisis clíniques, Hospital Nostra Senyora de Meritxell	LBM de CHU de Toulouse, Hôpitaux de Toulouse	Garcia M et al.; Lobaco C; Rendon M
EPI_ISL_12906839, EPI_ISL_12906840	Laboratorio Central de Salud Publica	Laboratorio de Biología Molecular, Instituto de Medicina Regional on behalf of 'Proyecto Argentino Interinstitucional de genomica de SARS-CoV-2' (PAIS Consortium)	Andrea Ayala; Bettina Brusés; Erica Struss; Esteban Paredes; Griselda Oria; Horacio Lucero.; Javier Mussin; Laura Formichelli; Melina Lorenzini Campos; Raúl Maximiliano Acevedo; Verónica Gómez; Victoria Femenías
EPI_ISL_13350750	Laboratorio Central de Salud Pública	Laboratorio Central de Salud Pública	Analía Rojas; Andrea Gómez de la Fuente; Cynthia Vazquez; César Cantero; César Ojeda; Emmanuel Céspedes; Fátima Fleitas; Ivana Fernández; Juan Torales; Julio Barrios; Maria Liz Gamarra; María Jose Duarte; Sandra González; Shirley Villalba; Tania Alfonzo; Wilson Benítez.
EPI_ISL_13020757, EPI_ISL_13228829, EPI_ISL_13228831	Laboratorio Central de Saude Publica do Estado de Santa Catarina (LACEN/SC)	Laboratory of Respiratory Viruses and Measles, Oswaldo Cruz Institute, FIOCRUZ	Alice Sampaio Rocha; Bruna Mendonça da Silva; Darcita Buerger Rovaris; Elisa Cavalcante Pereira; Fernando Motta; Ighor Arantes; Jéssica Graça Macedo de Carvalho; Larissa Macedo Pinto; Luciana Appolinario; Marilda Siqueira on behalf of the Fiocruz COVID-19 Genomic Surveillance Network; Paola Resende; Sandra Bianchini Fernandes; Victor Guimaraes
EPI_ISL_12680632	Laboratorio Central de Saude Publica do Estado do Parana (LACEN/PR)	Laboratory of Respiratory Viruses and Measles, Oswaldo Cruz Institute, FIOCRUZ	Alice Sampaio Rocha; Bruna Mendonça da Silva; Elisa Cavalcante Pereira; Fernando Motta; Ighor Arantes; Irina Riediger; Jéssica Graça Macedo de Carvalho; Larissa Macedo Pinto; Luciana Appolinario; Marilda Siqueira on behalf of the Fiocruz COVID-19 Genomic Surveillance Network; Paola Resende; Victor Guimaraes
EPI_ISL_13350777	Laboratorio Curie SRL	Laboratorio Central de Salud Pública	Analía Rojas; Andrea Gómez de la Fuente; Cynthia Vazquez; César Cantero; César Ojeda; Emmanuel Céspedes; Fátima Fleitas; Ivana Fernández; Juan Torales; Julio Barrios; Maria Liz Gamarra; María Jose Duarte; Sandra González; Shirley Villalba; Tania Alfonzo; Wilson Benítez.
EPI_ISL_12543211	Laboratorio Nacional de Salud (LNS), Guatemala	Inciensa, Instituto Costarricense de Investigación y Enseñanza en Nutrición y Salud	Claudia Estrada; César Conde; Laboratorio de Genómica INCIENSA; Selene González
EPI_ISL_13081535, EPI_ISL_13081537, EPI_ISL_13081539,	Laboratorio Nacional de Salud (LNS), Guatemala	Laboratorio Nacional de Salud (LNS), Guatemala	Calan K; García G; M; Mendoza L; Perez

EPI_ISL_13096537, EPI_ISL_13096541, EPI_ISL_13096550			
EPI_ISL_12694830, EPI_ISL_12694862, EPI_ISL_12694865, EPI_ISL_13221697, EPI_ISL_13221706, EPI_ISL_13221709, EPI_ISL_13221730, EPI_ISL_13221731			
see above	Laboratorio de Infectologia y Virologia Molecular	Laboratory of Molecular Virology, School of Medicine, Pontificia Universidad Catolica de Chile	Ana Maria Contreras; Andres E. Munoz-Marcos; Carlos Palma; Catalina Pardo-Roa; Constanza Maldonado; Eileen Serrano; Erick Salinas; Estefany Poblete; Eugenia L. Fuentes Luppichini; Francisco Melo; Jennifer Angulo; Jorge Levican; Leonardo I. Almonacid; M. Belen Leyton; Marcela Ferres; Maria Jose Avendano; María José Avendaño; Rafael A. Medina.; Tamara Garcia-Salum
EPI_ISL_9791182	Laboratorium Diagnostyki Medycznej	Wojewódzka Stacja Sanitarno-Epidemiologiczna w Gdańsku	Barbara Skórczewska; Gabriela Rutkowska; Karolina Smarzyńska
EPI_ISL_11701733	Laboratorium Medyczne GynCentrum – Oddział Sosnowiec	Wojewódzka Stacja Sanitarno-Epidemiologiczna w Katowicach	Adrian Miara; Agata Chajda; Beata Rozwadowska; Dorota Liberska; Elzbieta Bartkowiak; Hubert Okla; Marta Albertynska; Natalia Kuczera
EPI_ISL_11269129	Laboratory Med Diagnostics	Department for Virology, Molecular Biology and Genome Research, R. G. Lugar Center for Public Health Research, National Center for Disease Control and Public Health (NCDC) of Georgia.	Adam Kotorashvili; Amiran Gamkrelidze.; Ana Papkiauri; Ann Machablishvili; Anna Kasradze; Davit Tsaguria; Ekaterine Khmaladze; Ekaterine Zangaladze; Ekaterine Zhghenti; Giorgi Gogoladze; Giorgi Tomashvili; Gvantsa Brachveli; Gvantsa Chanturia; Irma Burjanadze; Ketevan Sidamonidze; Khatuna Zakhashvili; Lela Sabadze; Lela Urushadze; Magda Dgebuadze; Maia Alkhazashvili; Mari Gavashelidze; Mariam Zakalashvili; Marine Murtskhvaladze; Meri Pantsulaia; Nato Kotaria; Nino Berishvili; Paata Imnadze; Roena Sukhiashvili; Salome Javashvili; Tamar Jashiashvili; Tata Imnadze; Tea Tevdoradze
EPI_ISL_11269132	Laboratory Pyramid	Department for Virology, Molecular Biology and Genome Research, R. G. Lugar Center for Public Health Research, National Center for Disease Control and Public Health (NCDC) of Georgia.	Adam Kotorashvili; Amiran Gamkrelidze.; Ana Papkiauri; Ann Machablishvili; Anna Kasradze; Davit Tsaguria; Ekaterine Khmaladze; Ekaterine Zangaladze; Ekaterine Zhghenti; Giorgi Gogoladze; Giorgi Tomashvili; Gvantsa Brachveli; Gvantsa Chanturia; Irma Burjanadze; Ketevan Sidamonidze; Khatuna Zakhashvili; Lela Sabadze; Lela Urushadze; Magda Dgebuadze; Maia Alkhazashvili; Mari Gavashelidze; Mariam Zakalashvili; Marine Murtskhvaladze; Meri Pantsulaia; Nato Kotaria; Nino Berishvili; Paata Imnadze; Roena Sukhiashvili; Salome Javashvili; Tamar Jashiashvili; Tata Imnadze; Tea Tevdoradze
EPI_ISL_10645403, EPI_ISL_11704808	Laboratory for COVID19 diagnostics, Clinical Centre of Serbia	Institute of microbiology and Immunology, Faculty of Medicine, University of Belgrade	Jankovic, M.; Jovanovic, T.; Knezevic, A.; Milicevic, O.; Sekler, M.; Tesovic, B.; Vidanovic, D.
EPI_ISL_12236180, EPI_ISL_12337045, EPI_ISL_12345902	Laboratory for Molecular Diagnostics,IPHMN	Eurofins Genomics Europe Sequencing GmbH	Danijela Vujošević; Marija Govedarica; Rejhan Hot
EPI_ISL_9441481, EPI_ISL_11166339, EPI_ISL_11166342, EPI_ISL_11166343	Laboratory of Genetics and Personalized Medicine, Zan Mitrev Clinic	Laboratory of Genetics and Personalized Medicine, Zan Mitrev Clinic	Gjorgjievska M; Kungulovski G; Kungulovski G. et al.; et al.
EPI_ISL_11798846	Laboratory of Genomics and Bioinformatics, Comenius University Science Park	Laboratory of Genomics and Bioinformatics, Comenius University Science Park	Anna Kaliňáková; Barbora Kotvasová; Diana Rusňáková; Jakub Styk; Jaroslav Budiš; Lucia Ševčíková; Michaela Jakubková Forgáčová; Miroslav Böhmer; Nikola Lipková; Pavol Mišenko; Silvia Bokorová; Tatiana Sedláčková; Terézia Vrabľová; Tomáš Szemes
EPI_ISL_13653682	Laboratory of Respiratory Viruses and Measles, Oswaldo Cruz Institute, FIOCRUZ	Laboratory of Respiratory Viruses and Measles, Oswaldo Cruz Institute, FIOCRUZ	Alice Sampaio Rocha; Bruna Mendonça da Silva; Elisa Cavalcante Pereira; Fernando Motta; Ighor Arantes; Jéssica Graça Macedo de Carvalho; Larissa Macedo Pinto; Luciana Appolinario; Marilda Siqueira on behalf of the Fiocruz COVID-19 Genomic Surveillance Network; Paola Resende; Victor Guimaraes
EPI_ISL_12978303	Laboratório de Microbiologia Molecular - Universidade FEEVALE	Laboratório de Microbiologia Molecular - Universidade FEEVALE	Alana Witt Hansen; Fernando Rosado Spilki; Fágner Henrique Heldt; Juliana Schons Gularte; Juliane Deise Fleck; Mariana Soares da Silva; Matheus Nunes Weber; Meriane Demoliner; Micheli Filippi; Paula Rodrigues de Almeida; Viviane Girardi; Vyctoria Malayhka
EPI_ISL_13169087	MANILA DOCTORS HOSPITAL	Philippine Genome Center	Alethea R. de Guzman; Alyssa Joyce E. Telles; Anna Ong-Lim; Arianne A. Zamora; Benedict A. Maralit; Carlo M. Lapid; Celia Carlos; Cynthia P. Saloma; Devon Ray Pacial; Diomedes A. Cariño; Edsel Maurice Salvana; El King D. Morado; Elcid Aaron R. Pangilinan; Eva Maria Cutiongco-de la Paz; Francis A. Tablizo; Henrietta Marie Rodriguez; Jaime C. Montoya; Jan Michael C. Yap; Jarvin E. Nipales; Jo-Hannah S. Llames; John Michael Egana; John Q. Wong; Joshua Gregor A. Dizon; Joshua Jose Endozo; Juan Antonio R. Magalang; Karol Sophia Agape R. Padilla; Kris P. Punayan; Kristina Patriz Dela Cruz; Lindsay Clare D.L. Carandang; Ma. Exanil Plantig; Marc Edsel C. Ayes; Maria Rosario Singh-Vergeire; Maria Sofia L. Yangzon; Marielle M. Gamboa; Marissa Alejandria; Niña Francesca Bustamante; Razel Nikka M. Hao; Renato Jacinto Q. Mantaring; Rianna Patricia S. Cruz; Shiela Mae M. Araiza; Yvonne Valerie Austria; Zipporah Mariebelle R. Enriquez; Zyrel V. Mollejon
EPI_ISL_11523594	Medical Genetics Laboratory, Regional Centre of Medical Genetics, Emergency County Hospital Craiova	Medical Genetics Laboratory, Regional Centre of Medical Genetics, Emergency County Hospital Craiova	Adina Dragos; Ana-Maria Buga; Anca-Lelia (Riza) Costache; Andrei Pirvu; Elena Plesea; Ioana Streata; Mihai Cucu; Mihai Ioana; Monica Cara; Razvan Plesea; Stefania Dorobantu
EPI_ISL_12006316, EPI_ISL_12006363, EPI_ISL_12006364	Medical Genetics, Pamukkale University	Medical Genetics, Pamukkale University	Sari, T.; Tokgun, O.
EPI_ISL_12559611	Medyczne Laboratorium Diagnostyczne, Szpital Miejski nr 4 w Gliwicach Sp. z.o.o	Wojewódzka Stacja Sanitarno-Epidemiologiczna w Katowicach	Adrian Miara; Agata Chajda; Beata Rozwadowska; Dorota Liberska; ElZbieta Bartkowiak; Hubert Okla; Marta AlbertyNska; Natalia Kuczera
EPI_ISL_12315253	Microbiology Department, University Hospital Donostia	Microbiology Department, University Hospital Donostia	Cilla G.; Marimon JM; Martin-Peñaranda T; Montes M; Piñeiro L; Sorarrain A; Vallejo P
EPI_ISL_12130670, EPI_ISL_12484402, EPI_ISL_12484670, EPI_ISL_12484857	Microbiology Department. Complexo Hospitalario Universitario de Vigo	Microbiology Department. Complexo Hospitalario Universitario de Vigo	Microbiology Department. Complexo Hospitalario Universitario de Vigo
EPI_ISL_12420377, EPI_ISL_13043517, EPI_ISL_13043700	Molecular Biology Laboratory, Vidant Medical Center	Brody Integrative Genomics Core, East Carolina University	Changhong Yin; Heather Duncan; James Woodward; John T. Fallon; Kimberly P. Briley; Weihua Huang
EPI_ISL_12875335, EPI_ISL_12875336, EPI_ISL_12875340, EPI_ISL_12875371, EPI_ISL_12875386, EPI_ISL_13032234, EPI_ISL_13032235, EPI_ISL_13032269, EPI_ISL_13032270, EPI_ISL_13032282			
see above	Molecular Genetic Monitoring Group	Molecular Genetic Monitoring Group	Anna S. Gladkikh; Areg A.Totolian; Ekaterina O. Klyuchnikova; Valerya A. Sbarzaglia; Vladimir G. Dedkov
EPI_ISL_13259123, EPI_ISL_13259130	NIH	National Institute of Hygiene	Abderrahman Bimouhen; Fatima El Falaki; Hassan Ihazmade; Hicham Oumzil; Zakia Regragui
EPI_ISL_13289770, EPI_ISL_13289786, EPI_ISL_13289787, EPI_ISL_13289790, EPI_ISL_13289793	NIH-Rabat	NIC at National Institute of Hygiene	Abderrahman Bimouhen; Fatima El Falaki; Hassan Ihazmade; Hicham Oumzil; Mohammed Rajaoui; Zakia Regragui
EPI_ISL_12643819, EPI_ISL_12643823, EPI_ISL_12791912, EPI_ISL_12791923, EPI_ISL_13078896, EPI_ISL_13345968	NL-Dr. Leonard A. Miller Centre for Health Services	Newfoundland and Labrador - Eastern Health	Ana Duggan; Anna Majer; Anneliese Landgraff; CanCOGeN's metadata curation team; Christopher Corkum; Darian Hole; Elsie Grudeski; Gary Van Domselaar; Geoffrey Woodland; George Zahariadis; Grace Seo; Jennifer Tanner; Kirsten Biggar; Madison Chapel; Morag Graham; Natalie Knox; Nathalie Bastien; Phil Andrews; Philip Mabon; Public Health Agency of Canada CanCOGeN team; Rhiannon Huzarewich; Robert Needle; Russell Mandes; Shari Tyson; Timothy Booth; Yan Li; Yang Yu
EPI_ISL_13138232, EPI_ISL_13138351, EPI_ISL_13138352, EPI_ISL_13138609, EPI_ISL_13138625, EPI_ISL_13138640, EPI_ISL_13138695, EPI_ISL_13179231, EPI_ISL_13180592, EPI_ISL_13180788			
see above	National Center of Infectious and Parasitic Diseases	National Center of Infectious and Parasitic Diseases	Alexiev et al
EPI_ISL_9342570, EPI_ISL_10893985, EPI_ISL_11526215	National Health Laboratory	Botswana Harvard HIV Reference Laboratory	Boitumelo Zuze; Botshelo Radibe; Dorcas Maruapula; Joseph Makhema; Keoratile Ntshambiwa; Kgomotso Moruisi; Legodile Kooepile; Mosepele Mosepele; Mphaphi B. Mbulawa; Ontlametse T. Bareng; Pamela Smith-Lawrence; Roger Shapiro; Sefetogi Ramaologa; Shahin Lockman; Shirley Johane; Sikhulile Moyo; Simani Gaseitsiwe; Thongbotho Mphoyakgosi; Wonderful T. Choga
EPI_ISL_11674429, EPI_ISL_12587875, EPI_ISL_12872672	National Health Laboratory Services, Virology	National Health Laboratory Services, Virology	Ashlyn S. C. Davis; Florette K. Treurnicht; Kathleen Subramoney; Nkhensani Mtileni; Selebogo F. Maoko
EPI_ISL_11209670, EPI_ISL_11209674, EPI_ISL_11209679, EPI_ISL_11209697, EPI_ISL_11209699	National Influenza Center - Rafic Hariri University Hospital	National Influenza Center Lebanon Rafik Hariri University Hospital	Alissar Zalghout; Amirtharaj Francis; Hanan Abbass; Lina Mroueh; Mona Albuaini; Nada Ghosn; Nisrine Jammal
EPI_ISL_12603513	National Institute of Infectious Diseases-Prof. Dr. Matei Bals Molecular Diagnostics Laboratory	National Institute of Infectious Diseases-Prof. Dr. Matei Bals Molecular Diagnostics Laboratory	Corina Casangiu; Dan Otelea; Leontina Banica; Marius Surleac; Ovidiu Vlaicu; Simona Paraschiv

EPI_ISL_9696521, EPI_ISL_10067998	National Institute of Public Health	Virology Unit, Institut Pasteur du Cambodge	Cecile Troupin; Chau Darapheak; Chin Savuth; Erik A Karlsson; Jurre Y Siegers; Kraing Sidonn; Leakhena Pum; Ly Sovann; Sophoannadedh Rath; Veasna Duong; Yi Sengdoeurn
EPI_ISL_12252832	National Public Health Laboratory	National Public Health Laboratory	Kamal Hisham Bin Kamarul Zaman; Muhamad Syamim Bin Roslan; Noriah Binti Mohd Yusof; Nur Hazliza Binti Salleh; Rehan Shuhada Binti Abu Bakar; Selvanesan A/L Sengol; Yu Kie A/P Chem
EPI_ISL_10796087, EPI_ISL_10804816, EPI_ISL_10805960	National Virology Reference Laboratory	Microbial Genomic Services	Haziq Momin; Nor Azian Hafneh; Zainun Zaini
EPI_ISL_13020806	Nghe An General Hospital	Bachmai Hospital	Doanh Khuong; Dung Le; Lan Pham; Linh Le; Nga Pham; Ngan Le; Phuong Truong; Van Vu; Vuong Bui
EPI_ISL_11936110, EPI_ISL_13091544	OSPEDALE CIVILE TERAMO - CENTRO TRASFUSIONALE	Istituto Zooprofilattico Sperimentale dell'Abruzzo e Molise "G. Caporale"	Ancora M; Calistri P; Cammà C; Caporale M; Curini V; Delli Compagni E; Di Domenico M; Di Lollo Valeria; Di Pasquale A; Lorusso A; Mangone I; Marcacci M; Puglia I; Rinaldi A; Savini G; Scialabba S
EPI_ISL_13372020	Oblastni nemocnice Trutnov a.s.	University Hospital Hradec Kralove	Helena Parova; Lenka Rysava; Marketa Gancarcikova; Monika Berankova
EPI_ISL_12806352	Orion Laboratories	Orion Laboratories	Andrew Campagna; Keisha Simoneaux
EPI_ISL_12635835	Ospedale Civile Teramo - Centro Trasfusionale	Istituto Zooprofilattico Sperimentale dell'Abruzzo e Molise "G. Caporale"	Ancora M; Calistri P; Cammà C; Caporale M; Curini V; Delli Compagni E; Di Domenico M; Di Lollo Valeria; Di Pasquale A; Lorusso A; Mangone I; Marcacci M; Puglia I; Rinaldi A; Savini G; Scialabba S
EPI_ISL_12851716, EPI_ISL_13331828, EPI_ISL_13331838, EPI_ISL_13331857	Outre Mer	Institut Pasteur	Angela Brisebarre; Camille Capel; Christophe Malabat; Corinne Maufrais; Etienne Simon-Lorière; Frédéric Lemoine; Julien Fumey; Louise Lefrançois; Marc BIRON; Marie-Hélène GLAUDON LOUVEAU DE LA GUIGNERAYE; Marion Barbet; Maud Vanpeene; Méline Bizard; Slim El Khiari; Sylvaine BASTIAN; Sylvie Behillil; Sylvie Van der Werf; Vincent Enouf
EPI_ISL_11759616, EPI_ISL_11894151, EPI_ISL_11997193, EPI_ISL_12179886, EPI_ISL_12465580, EPI_ISL_12499883, EPI_ISL_13283356, EPI_ISL_13324157			
see above	Outre Mer	National Reference Center for Viruses of Respiratory Infections, Institut Pasteur, Paris	Angela Brisebarre; Camille Capel; Christophe Malabat; Corinne Maufrais; Etienne Simon-Lorière; François NESTOUR; Frédéric Lemoine; Julien Fumey; Laurence FAGOUR; Louise Lefrançois; Marc BIRON; Marie-Hélène GLAUDON LOUVEAU DE LA GUIGNERAYE; Marion Barbet; Maud Vanpeene; Méline Bizard; Slim El Khiari; Stéphanie GUYOMARD-RABENIRINA; Sylvie Behillil; Sylvie Van der Werf; Vincent Enouf
EPI_ISL_13169212	PHILIPPINE GENERAL HOSPITAL (PGH)	Philippine Genome Center	Alethea R. de Guzman; Alyssa Joyce E. Telles; Anna Ong-Lim; Arianne A. Zamora; Benedict A. Maralit; Carlo M. Lapid; Celia Carlos; Cynthia P. Saloma; Devon Ray Pacial; Diomedes A. Cariño; Edsel Maurice Salvana; El King D. Morado; Elcid Aaron R. Pangilinan; Eva Maria Cutiongco-de la Paz; Francis A. Tablizo; Henrietta Marie Rodriguez; Jaime C. Montoya; Jan Michael C. Yap; Jarvin E. Nipales; Jo-Hannah S. Llames; John Michael Egana; John Q. Wong; Joshua Gregor A. Dizon; Joshua Jose Endozo; Juan Antonio R. Magalang; Karol Sophia Agape R. Padilla; Kris P. Punayan; Kristina Patriz Dela Cruz; Lindsay Clare D.L. Carandang; Ma. Exanil Plantig; Marc Edsel C. Ayes; Maria Rosario Singh-Vergeire; Maria Sofia L. Yangzon; Marielle M. Gamboa; Marissa Alejandria; Niña Francesca Bustamante; Razel Nikka M. Hao; Renato Jacinto Q. Mantaring; Rianna Patricia S. Cruz; Shiela Mae M. Araiza; Yvonne Valerie Austria; Zipporah Mariebelle R. Enriquez; Zyrel V. Mollejon
EPI_ISL_13312858	PO AVEZZANO - U.O.C. Laboratorio Analisi	Istituto Zooprofilattico Sperimentale dell'Abruzzo e Molise "G. Caporale"	Ancora M; Calistri P; Cammà C; Caporale M; Curini V; Delli Compagni E; Di Domenico M; Di Lollo Valeria; Di Pasquale A; Lorusso A; Mangone I; Marcacci M; Puglia I; Rinaldi A; Savini G; Scialabba S
EPI_ISL_13345346	Palapye Primary Hospital Laboratory	Botswana Harvard HIV Reference Laboratory	Boitumelo Zuze; Botshelo Radibe; Dorcas Maruapula; Joseph Makhema; Keoratile Ntshambiwa; Kgomotso Moruisi; Legodile Kooepile; Lynnette N. Bhebhe; Mosepele Mosepele; Mphaphi B. Mbulawa; Ontlametse T. Bareng; Pamela Smith-Lawrence; Patrick T. Mokgethi; Roger Shapiro; Sefetogi Ramaologa; Shahin Lockman; Sikhulile Moyo; Simani Gaseitsiwe; Thongbotho Mphoyakgosi; Wonderful T. Choga
EPI_ISL_12139787	Pardubicka nemocnice	University Hospital Hradec Kralove	Helena Kovarikova; Lenka Rysava; Marketa Gancarcikova; Monika Berankova
EPI_ISL_11742048	Pathocare Pathology Laboratory, Vadodara	Gujarat Biotechnology Research Centre	Akhilesh Modi; Apurvasinh Puvar; Bhadreshsinh Gohil; Chaitanya Joshi; Disha Vora; Janvi Raval; Jaykumar Rangani; Madhvi Joshi; Nimesh Patel; Nitin Savaliya; Nitin Shukla; Priyank Chavda; Ramesh Pandit; Roshani Mishra; Sonal Sharma; Tasnim Trivedi; Viral Patel; Zarna Patel
EPI_ISL_12206505	Province Public Health Laboratory	Province Public Health laboratory	Amrendra Mishra; Jitendra Sah; Manmohan Mishra; Shravan Kumar Mishra
EPI_ISL_11370184, EPI_ISL_11370186, EPI_ISL_11370187, EPI_ISL_11402758	Provincial Public Health Reference Lab, Punjab AIDS Control Program, Lahore	Provincial Public Health Reference Lab, Punjab AIDS Control Program, Lahore	Abida Bano; Fuzail Ahmad; Hasnain Javed; Laiba; Warda Fatima
EPI_ISL_13069004	R SOLVAY 27	Institut de Pathologie et Genetique (IPG)	Jérémie Gras; Pascale Hilbert
EPI_ISL_10816832, EPI_ISL_10816834, EPI_ISL_10816835, EPI_ISL_10816836, EPI_ISL_10816837, EPI_ISL_10816843	Recherche	National Reference Center for Viruses of Respiratory Infections, Institut Pasteur, Paris	Angela Brisebarre; Camille Capel; Christophe Malabat; Corinne Maufrais; Etienne Simon-Lorière; Frédéric Lemoine; Julien Fumey; Louise Lefrançois; Marion Barbet; Maud Vanpeene; Méline Bizard; Olivier DEJOUX; Slim El Khiari; Sylvie Behillil; Sylvie Van der Werf; Vincent Enouf
EPI_ISL_12727219	Regional Medical Sciences Center 1 Chiang Mai	National Institute of Health, Department of Medical Sciences, Ministry of Public Health, Thailand	Archawin Rojanawiwat; Natchaya Khiadsang; Nuttida Thongpramul; Pakorn Piromtong; Pilailuk Okada; Sirikanda Wimol; Siripaporn Phuygun; Sunthareeya Waicharoen; Suratchana Mitrat; Thanutsapa Thanadachakul
EPI_ISL_12141415	Regional Medical Sciences Center 12/1 Trang	National Institute of Health, Department of Medical Sciences, Ministry of Public Health, Thailand	Archawin Rojanawiwat; Natchaya Khiadsang; Nuttida Thongpramul; Pakorn Piromtong; Pilailuk Okada; Sirikanda Wimol; Siripaporn Phuygun; Sunthareeya Waicharoen; Suratchana Mitrat; Thanutsapa Thanadachakul
EPI_ISL_11179283, EPI_ISL_11179518	Regional Medical Sciences Center 6 Chonburi	Medical Genomic Centre,Medical Life Sciences Institute,Department of Medical Sciences, Ministry of Public Health, Thailand	Archawin Rojanawiwat; Jirapha Pakdee; Natthakul Bunneang; Nuanjun Wichukchinda; Penpitcha Thawong; Pilailuk Akkapaiboon Okada; Pundharika Piboonsiri; Surakameth Mahasirimongkol; Waritta Sawaengdee
EPI_ISL_11986011	S.C. Laboratorio Analisi, ASL 3 Liguria	U.O. Igiene, Ospedale Policlinico San Martino	Bruzzone Bianca; De Pace Vanessa; Domnich Alexander; Icardi Giancarlo on behalf of SARS-CoV-2 ITALIAN RESEARCH ENTERPRISE-(SCIRE) Collaborative Group; Orsi Andrea; Randazzo Nadia; Ricucci Valentina; Spitaleri Antonino; Stefanelli Federica
EPI_ISL_11864810	SALUD DIGNA	Instituto Nacional de Medicina Genomica	Abraham Campos-Romero; Cedro-Tanda A; Escobar-Arrazola MA; Garcia-Garcia FE; Garnica-Lopez Dora; Herrera-Montalvo LA.; Hidalgo-Miranda A; Luna-Ruiz Marco; Mendoza-Vargas A; Moreno-Camacho José Luis; Ramirez-Vega O; Rangel-DeLeon D; Reyes-Grajeda JP; Rodriguez-Gallegos Jorge; Sanchez-Xochipa S; Yair Alfaro-Mora
EPI_ISL_12685438, EPI_ISL_12685498, EPI_ISL_12727363	SC (UCO) Igiene e Sanità Pubblica, ASUGI, Trieste	SC (UCO) Igiene e Sanità Pubblica, ASUGI, Trieste	Basaglia G; Busetti M; D'Agaro P; Fontana F; Forciniti G; Koncan R; Pipan C; Piscianz E; Segat L
EPI_ISL_9859616, EPI_ISL_11438687, EPI_ISL_11438717, EPI_ISL_11438720	SI «Public Health Center of MHU»	The Institute of Molecular Biology and Genetics of NASU	M.Tukalo et al.
EPI_ISL_13169039	SOUTHERN ISABELA MEDICAL CENTER MOLECULAR DIAGNOSTIC & RESEARCH LABORATORY (SIMC - MDRL)	Philippine Genome Center	Alethea R. de Guzman; Alyssa Joyce E. Telles; Anna Ong-Lim; Arianne A. Zamora; Benedict A. Maralit; Carlo M. Lapid; Celia Carlos; Cynthia P. Saloma; Devon Ray Pacial; Diomedes A. Cariño; Edsel Maurice Salvana; El King D. Morado; Elcid Aaron R. Pangilinan; Eva Maria Cutiongco-de la Paz; Francis A. Tablizo; Henrietta Marie Rodriguez; Jaime C. Montoya; Jan Michael C. Yap; Jarvin E. Nipales; Jo-Hannah S. Llames; John Michael Egana; John Q. Wong; Joshua Gregor A. Dizon; Joshua Jose Endozo; Juan Antonio R. Magalang; Karol Sophia Agape R. Padilla; Kris P. Punayan; Kristina Patriz Dela Cruz; Lindsay Clare D.L. Carandang; Ma. Exanil Plantig; Marc Edsel C. Ayes; Maria Rosario Singh-Vergeire; Maria Sofia L. Yangzon; Marielle M. Gamboa; Marissa Alejandria; Niña Francesca Bustamante; Razel Nikka M. Hao; Renato Jacinto Q. Mantaring; Rianna Patricia S. Cruz; Shiela Mae M. Araiza; Yvonne Valerie Austria; Zipporah Mariebelle R. Enriquez; Zyrel V. Mollejon
EPI_ISL_13169032	SUPERCARE MEDICAL SERVICES, INC.	Philippine Genome Center	Alethea R. de Guzman; Alyssa Joyce E. Telles; Anna Ong-Lim; Arianne A. Zamora; Benedict A. Maralit; Carlo M. Lapid; Celia Carlos; Cynthia P. Saloma; Devon Ray Pacial; Diomedes A. Cariño; Edsel Maurice Salvana; El King D. Morado; Elcid Aaron R. Pangilinan; Eva Maria Cutiongco-de la Paz; Francis A. Tablizo; Henrietta Marie Rodriguez; Jaime C. Montoya; Jan Michael C. Yap; Jarvin E. Nipales; Jo-Hannah S. Llames; John Michael Egana; John Q. Wong; Joshua Gregor A. Dizon; Joshua Jose Endozo; Juan Antonio R. Magalang; Karol Sophia Agape R. Padilla; Kris P. Punayan; Kristina Patriz Dela Cruz; Lindsay Clare D.L. Carandang; Ma. Exanil Plantig; Marc Edsel C. Ayes; Maria Rosario Singh-Vergeire; Maria Sofia L. Yangzon; Marielle M. Gamboa; Marissa Alejandria; Niña Francesca Bustamante; Razel Nikka M. Hao; Renato Jacinto Q. Mantaring; Rianna Patricia S. Cruz; Shiela Mae M. Araiza; Yvonne Valerie Austria; Zipporah Mariebelle R. Enriquez; Zyrel V. Mollejon
EPI_ISL_13134834	SURA	Laboratorio Departamental de Salud Publica de Antioquia	Ana Victoria Valencia Duarte; Cristian Arbey Velarde Hoyos; Gloria Isabel Escobar; Idabely Betancur Ortiz; Juan Pablo Isaza Agudelo
EPI_ISL_13300419	SYNLAB ANGEL DIAGNOSTICA	Universidad del Valle	Andres Castillo; Beatriz Parra & Programa Nacional de Caracterización Genómica de SARS-CoV-2; David Valencia; Diana López-Alvarez; Erica M. Aristizabal; Flor Saa; Melissa Solarte; Nelson Rivera Franco
EPI_ISL_12871530	Sari University of Medical Sciences	National Influenza Center	A Nejati; Adel Abedi and T Mokhtari Azad; J Yavarian; K Sadeghi; Marzieh Fraji-Zonouz; NZ Shafiei Jandaghi; Nastaran Ghavami; Sevrin Zadheidar; V Salimi
EPI_ISL_11750302	Sayang Cianjur General Hospital Laboratory	West Java Health Laboratory; School of Life Sciences and Technology, Institut Teknologi Bandung	Azzania Fibriani; Cut Nur Cinthia Alamanda; Ema Rahmawati; Hadiana; Karimatu Khoirunnisa; Miftahul Faridl; Rifky Waluyajati Rachman; Rini Robiani; Ryan Bayusantika Ristandi
EPI_ISL_12970541	Semnan University of Medical Sciences	National Influenza Center	A Nejati; Adel Abedi; J Yavarian; K Sadeghi; Mrzieh Faraji-Zonouzand T Mokhtari Azad; NZ Shafiei Jandaghi; Nastaran Ghavami; Sevrin Zadheidar; V Salimi
EPI_ISL_12139809, EPI_ISL_12139871, EPI_ISL_12144237, EPI_ISL_12184894, EPI_ISL_12185039	Servicio Microbiología H.U. Dr. Negrín	Servicio Microbiología H.U. Dr. Negrín	Ana Bordes Benítez; Francisco Javier Chamizo López; Francisco Javier Chamizo López Ana Bordes Benítez
EPI_ISL_12871526, EPI_ISL_12970531	Shahrekord University of Medical Sciences	National Influenza Center	A Nejati; Adel Abedi; Adel Abedi and T Mokhtari Azad; J Yavarian; K Sadeghi; Marzieh Faraji-Zonouz; NZ Shafiei Jandaghi; Nastaran Ghavami; Sevrin Zadheidar; V Salimi; and T Mokhtari Azad

EPI_ISL_9698125, EPI_ISL_9963286, EPI_ISL_10444842, EPI_ISL_10798736, EPI_ISL_11287432, EPI_ISL_11737003, EPI_ISL_11739553, EPI_ISL_11739584, EPI_ISL_13029278, EPI_ISL_13066207			
see above	Synlab Eesti OÜ	1. Laboratory of Communicable Diseases (Estonia); 2. Eurofins Genomics Europe Sequencing GmbH	Abroi A.; Avi R.; Dotsenko L.; Epštein J.; Hoidmets D.; Huik K.; Härma M-A.; Jaaniso E.; Kaarna K.; Kallas E.; Koppel I.; Kuzmin I.; Lahesaare A.; Lutsar I.; Metspalu M.; Milani L.; Naaber P.; Niglas H.; Oopkaup O.E.; Pauskar M.; Peterson H.; Päll T.; Ratnik K.; Raudvere U.; Reisberg T.; Sadikova O.; Sepp H.; Shablinskaja A.; Suija H.; Talas U.G.; Truusalu K.
EPI_ISL_11586788	Szpital Uniwersytecki w Krakowie	Wojewódzka Stacja Sanitarno-Epidemiologiczna w Katowicach	Adrian Miara; Agata Chajda; Beata Rozwadowska; Dorota Liberska; Elzbieta Bartkowiak; Hubert Okla; Marta Albertynska; Natalia Kuczera
EPI_ISL_11147430	TSGH-CP molecular lab	TSGH-CP molecular lab	Cherng-Lih Perng; Chien-Wen Chen; Chih-Kai Chang; Feng-Yee Chang; Hsing-Yi Chung; Hung-Sheng Shang; Jung-Chung Lin; Kuo-Ming Yeh; Kuo-Sheng Hung; Ming-Jr JIAN; Sheng-Kang Chiu; Shih-Hung Tsai; Tien-Yao Chang
EPI_ISL_13322005	Thai Binh General Hospital	Bachmai Hospital	Doanh Khuong; Dung Le; Lan Pham; Linh Le; Nga Pham; Ngan Le; Phuong Truong; Van Vu; Vuong Bui
EPI_ISL_11583310	Thai Binh hospital	Bachmai Hospital	Doanh Khuong; Dung Le; Lan Pham; Linh Le; Nga Pham; Ngan Le; Phuong Truong; Van Vu; Vuong Bui
EPI_ISL_12248962, EPI_ISL_12870044	Tokyo Medical and Dental University	Tokyo Medical and Dental University	Akinori Kimura; Hiroaki Takeuchi; Kousuke Tanimoto; Yoko Nukui; Yoshiaki Gu; Yukie Tanaka
EPI_ISL_11221479	Turkish Society of Internal Medicine	Gen Era Diagnostics Corporation Life Science Research and Molecular Diagnostics	Serhat Ünal
EPI_ISL_12195111, EPI_ISL_12212702	USL1	A.O. PERUGIA	Bicchieraro G; Bondi P; Cappelletti E; Ciurnelli R; Lepri E; Lucheroni F; Malagigi V; Spaccapelo R; Tacconi P
EPI_ISL_12771335	Unit Forensik, Hospital Kepala Batas	iPROMISE, UiTM	Ariza Adnan; Fadzilah Mohd Nor; Lim Wai Feng; Mohd Asif Mohd Sukri; Mohd Nur Fakhruzzaman Noorizhab; Mohd Zaki Salleh; Sazzli Shahlan Kassim; Siti Farah Alwani Mohd Nawi; Siti Hamimah Sheikh Abdul Kadir; Teh Lay Kek; Wang Seok Mui
EPI_ISL_10440805, EPI_ISL_11159932, EPI_ISL_12862020	University Hospital Hradec Kralove	University Hospital Hradec Kralove	Helena Kovarikova; Lenka Rysava; Marketa Gancarcikova; Monika Berankova
EPI_ISL_11010632	Università degli Studi di Perugia	Istituto Zooprofilattico Sperimentale dell'Abruzzo e Molise "G. Caporale"	Ancora M; Biagetti M; Calistri P; Camilloni B; Cammà C; Curini V; Delli Compagni E; Di Domenico M; Di Pasquale A; Giammarioli M; Lorusso A; Mangone I; Marcacci M; Mencacci A; Puglia I; Rinaldi A; Savini G; Scialabba S
EPI_ISL_13172633, EPI_ISL_13173728, EPI_ISL_13173897	VICENTE SOTTO MEMORIAL MEDICAL CENTER (VSMMC)	Philippine Genome Center	Alethea R. de Guzman; Alyssa Joyce E. Telles; Anna Ong-Lim; Arianne A. Zamora; Benedict A. Maralit; Carlo M. Lapid; Celia Carlos; Cynthia P. Saloma; Devon Ray Pacial; Diomedes A. Cariño; Edsel Maurice Salvana; El King D. Morado; Elcid Aaron R. Pangilinan; Eva Maria Cutiongco-de la Paz; Francis A. Tablizo; Henrietta Marie Rodriguez; Jaime C. Montoya; Jan Michael C. Yap; Jarvin E. Nipales; Jo-Hannah S. Llames; John Michael Egana; John Q. Wong; Joshua Gregor A. Dizon; Joshua Jose Endozo; Juan Antonio R. Magalang; Karol Sophia Agape R. Padilla; Kris P. Punayan; Kristina Patriz Dela Cruz; Lindsay Clare D.L. Carandang; Ma. Exanil Plantig; Marc Edsel C. Ayes; Maria Rosario Singh-Vergeire; Maria Sofia L. Yangzon; Marielle M. Gamboa; Marissa Alejandria; Niña Francesca Bustamante; Razel Nikka M. Hao; Renato Jacinto Q. Mantaring; Rianna Patricia S. Cruz; Shiela Mae M. Araiza; Yvonne Valerie Austria; Zipporah Mariebelle R. Enriquez; Zyrel V. Mollejon
EPI_ISL_10283481, EPI_ISL_10284065, EPI_ISL_10730607, EPI_ISL_10730610, EPI_ISL_10910333, EPI_ISL_11106023, EPI_ISL_11106084, EPI_ISL_11106669, EPI_ISL_11106789, EPI_ISL_11412329, EPI_ISL_11412331			
see above	Virology Laboratory, NAPH, Moldova	Virology Laboratory, NAPH, Moldova	Apostol Mariana; Cataraga Alina; Colac Svetlana; Olga Burduniuc
EPI_ISL_10314780, EPI_ISL_10635697, EPI_ISL_10635740, EPI_ISL_11023851, EPI_ISL_12089737, EPI_ISL_12089792, EPI_ISL_12089851			
see above	Virology Unit, Institut Pasteur du Cambodge	Virology Unit, Institut Pasteur du Cambodge	Cecile Troupin; Chau Darapheak; Chin Savuth; Erik A Karlsson; Jurre Y Siegers; Kraing Sidonn; Leakhena Pum; Ly Sovann; Sophoannadedh Rath; Veasna Duong; Yi Sengdoeurn
EPI_ISL_10847043	Vitomed Sp. z o.o.	Wojewódzka Stacja Sanitarno-Epidemiologiczna w Katowicach	Adrian Miara; Agata Chajda; Beata Rozwadowska; Elżbieta Bartkowiak; Hubert Okla; Marta Albertyńska; Natalia Kuczera
EPI_ISL_11789460	Đà Nẵng, Vietnam	Department of Microbiology and Immunology, Pasteur Institute in Nha Trang	Do Thai Hung; Hoang Tien Thanh; Huynh Kim Mai; Nguyen Bao Trieu; Nguyen Dinh Luong; Nguyen Duc Duy; Trinh Hoang Long; Vu Thi Ngoc
