# Supplementary material for: Tracing the international arrivals of SARS-CoV-2 Omicron variants after Aotearoa New Zealand reopened its border
Source: Nat Commun. 2022 Oct 29;13:6484. doi: 10.1038/s41467-022-34186-9 (PMC9617600; doi:10.1038/s41467-022-34186-9)
Supplement: Supplementary file 7 — Supplementary Data 5 [file 41467_2022_34186_MOESM7_ESM.pdf]

We gratefully acknowledge the following Authors from the Originating laboratories responsible for obtaining the specimens, as well as the Submitting laboratories where the genome data were generated and shared via GISAID, on which this research is based.

All Submitters of data may be contacted directly via www.gisaid.org

Authors are sorted alphabetically.

Acknowledgement EPI_SET Identifier: EPI_SET_20220706bz

Accession ID	Originating Laboratory	Submitting Laboratory	Authors
EPI_ISL_12273990, EPI_ISL_12274060, EPI_ISL_12307643, EPI_ISL_12476998, EPI_ISL_12520048, EPI_ISL_12763752, EPI_ISL_12763807, EPI_ISL_12765602, EPI_ISL_12903392, EPI_ISL_12903602			
see above	AMPATH	National Institute for Communicable Diseases of the National Health Laboratory Service	Amoako DG; Bhiman JN; Everatt J; Ismail A; Kekana D; Mahlangu B; Mnguni A; Mohale T; Ntuli N; Scheepers C; Wolter N
EPI_ISL_12790124	AOUC Azienda Ospedaliero-Universitaria Careggi	Microbiology and Virology Unit, Florence Careggi University Hospital; Department of Experimental and Clinical Medicine, University of Florence, Florence, Italy	Gianmaria Rossolini
EPI_ISL_12722930	AREA DE SALUD HEREDIA-CUBUJUQUI - CLINICA DR. FRANCISCO BOLAÑOS	Inciensa, Instituto Costarricense de Investigación y Enseñanza en Nutrición y Salud	Adriana Godínez; Claudio Soto-Garita; Estela Cordero; Francisco Duarte; Gabriel Morales & Natalia Bonilla; Hebleen Porras; José Luis Vargas; Mariela Gutiérrez; Melany Calderón; Sofia Herrera
EPI_ISL_12935378	AREA DE SALUD MATA REDONDA-HOSPITAL - CLINICA DR. MORENO CAÑAS	Inciensa, Instituto Costarricense de Investigación y Enseñanza en Nutrición y Salud	Adriana Godínez; Claudio Soto-Garita; Estela Cordero; Francisco Duarte; Gabriel Morales & Natalia Bonilla; Hebleen Porras; José Luis Vargas; Mariela Gutiérrez; Melany Calderón; Sofia Herrera
EPI_ISL_12278927	ASP Reggio Calabria Polo Sanitario Nord - Dr.ssa Fiorillo	SOC Microbiologia e Virologia - AO Pugliese-Ciaccio	Pasquale Minchella
EPI_ISL_12780020	ASST GOM NIGUARDA	ASST Grande ospedale Metropolitano Niguarda	Alice Nava
EPI_ISL_12401707, EPI_ISL_12401714, EPI_ISL_12401721, EPI_ISL_12496098, EPI_ISL_12496100, EPI_ISL_12496101, EPI_ISL_12496103, EPI_ISL_12496106, EPI_ISL_12496108, EPI_ISL_12496111, EPI_ISL_12496114, EPI_ISL_12496123, EPI_ISL_12496124, EPI_ISL_12563697			
see above	ASST MONZA	ASST MONZA	Sergio Maria Ivano Malandrin
EPI_ISL_12758092	AZ Klina	AZ Klina	Carl Vael; Lynsey Berckmans
EPI_ISL_13124152	Ampath	National Institute for Communicable Diseases of the National Health Laboratory Service	Amoako DG; Bhiman JN; Everatt J; Ismail A; Kekana D; Mahlangu B; Mnguni A; Mohale T; Ntuli N; Scheepers C; Wolter N
EPI_ISL_12206542	Area of Virology, Serology and Virology Division (SAViD), New South Wales Health Pathology Randwick	Virology Research Laboratory; Area of Virology, Serology and Virology Division (SAViD), New South Wales Health Pathology Randwick	Foster, C.; Jean, T.; Rawlinson, W.; Van Hal, S.; Wong, M.; Yeang, M.
EPI_ISL_12578776, EPI_ISL_12578777	Area of Virology, Serology and Virology Division (SAViD), New South Wales Health Pathology Randwick, Prince Of Wales Hospital	Area of Virology, Serology and Virology Division (SAViD), New South Wales Health Pathology Randwick, Prince Of Wales Hospital	Foster, C.; Jean, T.; Rawlinson, W.; Van Hal, S.; Wong, M.; Yeang, M.
EPI_ISL_12679229	Australian Clinical Labs (formerly Healthscope Pathology)	NSW Health Pathology - Institute of Clinical Pathology and Medical Research; Westmead Hospital; University of Sydney	Arnott A.; Draper J.; Gall M.; Martinez E.; Rockett R.; Sintchenko V.; on behalf of ICPMR
EPI_ISL_12727829, EPI_ISL_12896303, EPI_ISL_12896305, EPI_ISL_12896306, EPI_ISL_12896308, EPI_ISL_12896310, EPI_ISL_12896313			
see above	Austrian Agency for Health and Food Safety (AGES)	Bergthaler laboratory, CeMM Research Center for Molecular Medicine of the Austrian Academy of Sciences	Alberto Alises; Andreas Bergthaler; Anna Schedl; Christoph Bock; Fabian Amman; Lukas Endler; Matthew Thornton; Michael Schuster; Michelle Chan; Petr Triska
EPI_ISL_12421048	BIO VSM LAB	Department of Virology, Henri Mondor University Hospital, Assistance Publique Hôpitaux de Paris, Université Paris-Est Créteil, INSERM U955	Alexandre Soulier; Christophe Rodriguez; Elisabeth Trawinski; Guillaume Gricourt; Jean-Michel Pawlotsky; Melissa N'Debi; Slim Fourati; Vanessa Demontant
EPI_ISL_12837202, EPI_ISL_12837262	BioneXt LAB - Laboratoire d'analyses médicales	Microbiology, Microbial Genomics Platform, LNS Laboratoire National De Santé	Anke Wienecke-Baldacchino; Catherine Ragimbeau; Elodie Solarino; Eric Hugoson; Fatu Djabi; Jessica Tapp; Lise Pignon; Raoul Salmon; Sibel Berger; Tamir Abdelrahman; Thibault Ferrandon; Virginie Jover
EPI_ISL_12527983	Bioscientia Labor Wermsdorf	Robert Koch Institute	
EPI_ISL_12119232	Botswana Harvard HIV Reference Laboratory	Botswana Harvard HIV Reference Laboratory	Boitumelo Zuze; Dorcas Maruapula; Joseph Makhema; Kgomotso Moruisi; Legodile Kooepile; Mosepele Mosepele; Mphaphi B. Mbulawa; Ontlametse T. Bareng; Pamela Smith-Lawrence; Roger Shapiro; Sefetogi Ramaologa; Shahin Lockman; Shirley Johane; Sikhulile Moyo; Simani Gaseitsiwe; Thongbotho Mphoyakgosi; Wonderful T. Choga
EPI_ISL_12968476, EPI_ISL_13318092	British Columbia Centre For Disease Control	B.C. Centre for Disease Control Public Health Laboratory	Ana Pacagnella; Corrinne Ng; Dan Fornika; James Zlosnik; John Tyson; Kim Macdonald; Kimia Kamelian; Linda Hoang; Loretta Janz; Mel Krajden; Prystajecky Natalie; Robert Azana; Shannon Russell
EPI_ISL_12665547, EPI_ISL_12665652	British Columbia Centre For Disease Control	BCCDC Public Health Laboratory	Ana Pacagnella; Corrinne Ng; Dan Fornika; James Zlosnik; John Tyson; Kim Macdonald; Kimia Kamelian; Linda Hoang; Loretta Janz; Mel Krajden; Prystajecky Natalie; Robert Azana; Shannon Russell
EPI_ISL_12682132, EPI_ISL_12791446	Broad Institute Clinical Research Sequencing Platform	Infectious Disease Program, Broad Institute of Harvard and MIT	Adams, G.; B.L.; B.W.; Bauer, M.; Birren; Blumenstiel, B.; Brown, C.; Carter, A.; Chaluvadi, S.; D.J.; DeFelice, M.; DeRuff, K.; Dodge, S.; Gabriel, S.; Gallagher, G.; Gladden-Young, A.; Granger, B.; J.E.; K.J.; Lagerborg, K.; Larkin, K.; Lee, M.; Lemieux; Lennon, N.; Loreth, C.; Madoff, L.; McGovern, S.; Meldrim, J.; Normandin, E.; P.C.; Park; Pearlman, L.; Reilly, S.; Rudy, M.; Sabeti; Siddle; Smole, S.; Tomkins-Tinch, C.; Vicente, G.; and MacInnis
EPI_ISL_13276608, EPI_ISL_13276610, EPI_ISL_13276613, EPI_ISL_13276614, EPI_ISL_13276621, EPI_ISL_13276622, EPI_ISL_13276623, EPI_ISL_13276624, EPI_ISL_13276625, EPI_ISL_13276633, EPI_ISL_13276634, EPI_ISL_13276636			
see above	Bumrungrad International Hospital	National Institute of Health, Department of Medical Sciences, Ministry of Public Health, Thailand	Archawin Rojanawiwat; Natchaya Khiadsang; Nuttida Thongpramul; Pakorn Piromtong; Pilailuk Okada; Sirikanda Wimol; Siripaporn Phuygun; Sunthareeya Waicharoen; Suratchana Mitrat; Thanutsapa Thanadachakul
EPI_ISL_12954867, EPI_ISL_12954868, EPI_ISL_12954870, EPI_ISL_12954873, EPI_ISL_12954877, EPI_ISL_12954878, EPI_ISL_12954879, EPI_ISL_12954880, EPI_ISL_12954881, EPI_ISL_12954882, EPI_ISL_12954883, EPI_ISL_12954884, EPI_ISL_12954887, EPI_ISL_12954888, EPI_ISL_12954889, EPI_ISL_12954890, EPI_ISL_12954891, EPI_ISL_12954892, EPI_ISL_12954895, EPI_ISL_12954896, EPI_ISL_12954897, EPI_ISL_12954900, EPI_ISL_12954903			
see above	CENTRAL HEALTH LABORATORY	National Institute for Communicable Diseases of the National Health Laboratory Service	Amoako DG; Bhiman JN; Everatt J; Ismail A; Kekana D; Mahlangu B; Mnguni A; Mohale T; Ntuli N; Scheepers C; Wolter N
EPI_ISL_12688770, EPI_ISL_12688820, EPI_ISL_12688839	CH. E. MULLER	Department of Virology, Henri Mondor University Hospital, Assistance Publique Hôpitaux de Paris, Université Paris-Est Créteil, INSERM U955	Alexandre Soulier; Christophe Rodriguez; Elisabeth Trawinski; Guillaume Gricourt; Jean-Michel Pawlotsky; Melissa N'Debi; Slim Fourati; Vanessa Demontant
EPI_ISL_12763787	CHRIS HANI BARAGWANATH LABORATORY	National Institute for Communicable Diseases of the National Health Laboratory Service	Amoako DG; Bhiman JN; Everatt J; Ismail A; Kekana D; Mahlangu B; Mnguni A; Mohale T; Ntuli N; Scheepers C; Wolter N
EPI_ISL_13068754	CHUV	Laboratory of genomics and metagenomics, Institute of Microbiology, University Hospital Centre and University of Lausanne	Claire Bertelli; Damien Jacot; Gilbert Greub; Sébastien Aeby; Trestan Pillonel
EPI_ISL_13331190, EPI_ISL_13331210	Central Health Laboratory, Ministry of Health and Wellness, Mauritius	Central Health Laboratory, Ministry of Health and Wellness, Mauritius	Bahadoor BS; Mathur H; Ramuth M Janoo N; Sonoo J; Sujeewon C; Ubheeram J
EPI_ISL_12456544, EPI_ISL_12633207, EPI_ISL_12636562, EPI_ISL_12637066, EPI_ISL_12637330	Clinical Microbiology Laboratory, Tel Aviv Sourasky Medical Center	Clinical Microbiology Laboratory, Tel Aviv Sourasky Medical Center	Alon Ziv; Amos Adler; Goel Morad; Katya Levytskyi; Lior Handler; Matan Slutzkin; Ora Halutz; Orly Eshel

EPI_ISL_12701860	Clinical Microbiology, Infection Prevention and Control	Section for Molecular Diagnostics	Björn Hallström; Jonas Björkman
EPI_ISL_12660480, EPI_ISL_13017314	Clinique Saint-Pierre Ottignies	UCLouvain/IREC/MBLG-CTMA	Benoit Kabamba Mukadi; Bertrand Bearzatto; Jean-Luc Gala; Valentin Coste
EPI_ISL_12226685, EPI_ISL_12226709, EPI_ISL_12660422, EPI_ISL_12660425	Cliniques universitaires Saint-Luc	UCLouvain/IREC/MBLG-CTMA	Benoit Kabamba Mukadi; Bertrand Bearzatto; Jean-Luc Gala; Valentin Coste
EPI_ISL_13020144	Colorado Department of Public Health and Environment	Colorado Department of Public Health and Environment	Alexandria Rossheim; Arianna Smith; Diana Ir; Emily A. Travanty; Laura Bankers; Mandy Waters; Michael Martin; Molly C. Hetherington-Rauth; Shannon R. Matzinger
EPI_ISL_13363318	DASA	DASA	Adriano Bonaldi; Annelise Lopes; Bianca Cota; Camila Romano; Cristina Oliveira; Jose Levi; Keila Orneles; Laryssa Sassi; Luciane Sussuchi; Paulo Pierry; Rodrigo Guarischi; Rodrigo Salazar
EPI_ISL_12248508, EPI_ISL_12286266, EPI_ISL_12317495, EPI_ISL_12401348, EPI_ISL_12435606, EPI_ISL_12472206, EPI_ISL_12473024, EPI_ISL_12533737, EPI_ISL_12558347, EPI_ISL_12558354, EPI_ISL_12558608, EPI_ISL_12609354, EPI_ISL_12610106, EPI_ISL_12632184, EPI_ISL_12632471, EPI_ISL_12632539, EPI_ISL_12648815, EPI_ISL_12648960, EPI_ISL_12648962, EPI_ISL_12649098, EPI_ISL_12727163, EPI_ISL_12811576			
see above	Department of Bacteria, Parasites and Fungi, Statens Serum Institut, Copenhagen, Denmark	Statens Serum Institut Bioinformatics and Microbial Genomics	Danish Covid-19 Genome Consortium
EPI_ISL_12812558, EPI_ISL_12812568	Department of Health Technology and Informatics, The Hong Kong Polytechnic University	Department of Health Technology and Informatics, The Hong Kong Polytechnic University	Alan Ka-Lun Wu; Alex Yat-Man Ho; Barry Kin-Chung Wong; Chloe Toi-Mei Chan; David Ho-Keung Shum; Gilman Kit-Hang Siu; Hiu-Yin Lao; Ivan Tak-Fai Wong; Jake Siu-Lun Leung; Kam-Tong Yip; Kenneth Siu-Sing Leung; Kingsley King-Gee Tam; Kitty Sau-Chun Fung; Kristine Luk; Lam-Kwong Lee; Miranda Chong-Yee Yau; Sandy Ka-Yee Chau; Shea Ping Yip; Tak-Lun Que; Timothy Ting-Leung Ng; Wing Cheong Yam; Wing-Hei Lo; Wing-Kin To; Yvette Wai-Man Lai
EPI_ISL_13088328	Department of Laboratory Services, National Virology Reference Laboratory	Clinical Molecular Diagnostic Laboratory For Infectious Disease, Department of Laboratory Services, Microbial Genomic Services	Amal Nabihah Ahmad; Faezah Fariha Abd Latif; Haziq Momin; Izzati Azhar; Nor Azian Hafneh; Nur Amirah Ibarahim; Zainun Zaini
EPI_ISL_12471284, EPI_ISL_12715948	Department of Medical Microbiology & Infection prevention, Amsterdam University Medical Centers location AMC	Department of Medical Microbiology & Infection prevention, Amsterdam University Medical Centers location AMC	Akke Cornelissen; Fokla Zorgdrager; Janke Schinkel; Jelle Koopsen; Judith den Uil; Marcel Jonges; Matthijs Welkers; Menno de Jong; Robin van Houdt; Sebastien Matamoros; Sjoerd Rebers; Sylvia Bruisten; Tjalling Leenstra and Mariken van der Lubben on behalf of the Amsterdam Regional Genomic epidemiology and Outbreak Surveillance (ARGOS) consortium
EPI_ISL_13278440	Department of Veterinary Science and Department of Virology I, National Institute of Infectious Diseases	Research Center for Influenza and Respiratory Viruses, National Institute of Infectious Disease	Hideka Miura; Hideki Ebihara; Hideki Hasegawa; Ikuyo Takayama; Kaya Miyazaki; Ken Maeda; Mutsuyo Takayama-Ito; Seiichiro Fujisaki; Shiho Nagata; Shinji Watanabe; Shuetsu Fukushi; Takahiro Maeki; Tsukasa Yamamoto; Yudai Kuroda
EPI_ISL_13024747, EPI_ISL_13086515	Division of Emerging Infectious Diseases, Bureau of Infectious Diseases Diagnosis Control, Korea Disease Control and Prevention Agency	Division of Emerging Infectious Diseases, Bureau of Infectious Diseases Diagnosis Control, Korea Disease Control and Prevention Agency	Ae Kyung Park; Chae Young Lee; Eun-Jin Kim; Hyuck Jin Lee; Il-Hwan Kim; Jeong-Ah Kim
EPI_ISL_12751210	Dr. Mustafa, Dr. Richter Labor für medizinisch-chemische und mikrobiologische Diagnostik GmbH, Abteilung Molekularbiologie	Dr. Mustafa, Dr. Richter Labor für medizinisch-chemische und mikrobiologische Diagnostik GmbH, Abteilung Molekularbiologie	Alexander Gamisch; Maria Elisabeth Mustafa
EPI_ISL_12782902, EPI_ISL_12783249, EPI_ISL_12953365, EPI_ISL_12953676, EPI_ISL_12953692, EPI_ISL_12953764, EPI_ISL_12953785, EPI_ISL_12953795, EPI_ISL_12953796			
see above	Dutch COVID-19 response team	National Institute for Public Health and the Environment (RIVM)	Adam Meijer; Afke Vogelzang; AnneMarie van den Brandt; Annelies Kroneman; Bas van der Veer; Chantal Reusken; Dennis Schmitz; Dirk Eggink; Florian Zwagemaker; Harry Vennema; Ivo van Walle; Jeroen Cremer; Jil Kocken; Jordy de Bakker; Karim Hajji; Kim Freriks; Linda van Someren; Lisa Wijsman; Lynn Aarts; Ryanne Jaarsma; Sanne Bos; Sharon van den Brink; on behalf of the national COVID-19 response team
EPI_ISL_12832192, EPI_ISL_12832194	Edmonton Provincial Lab	Alberta Precision Labs (APL)	Buss E; Croxen M; Deo A; Dieu P; Ferrato C; Gill K; Granger D; Koleva P; Li V; Lloyd C; Lynch T; Ma R; Murphy S; Pabbaraju K; Rotich S; Shideler S; Shokopoles S; Skitsko T; Thayer J; Tipples G; Wong A; Yu C; Zelyas N.
EPI_ISL_12624687, EPI_ISL_12624817, EPI_ISL_12714036, EPI_ISL_12714066	Enfer	Enfer	Elaine M. Kenny; Suzie Coughlan
EPI_ISL_12589338, EPI_ISL_12706344, EPI_ISL_12706389, EPI_ISL_12706401	Eurofins-NMDL	Eurofins-NMDL	Anco Molijn; Anne Vogel; Lisa Dreesens; Marvin Ruiter; Maurine Leversteijn-van Hall; Roy Masius; Simon Lansu
EPI_ISL_13344356	Fundación Cardiovascular de Colombia Zona Franca	Molecular Genetics and Antimicrobial Resistance - UGRA, Universidad El Bosque	Alexandra Parada; Jinnethe Reyes; Lorena Diaz; Marcela Mercado; Mauricio Pacheco; Nicolas Forero; Sandra Rincon; Yordy Rodriguez
EPI_ISL_12688305	Genetica Molecular and Subdepartamento de Virologia ISP Chile	Instituto de Salud Publica de Chile	Andres Castillo; Barbara Parra; Constanza Campano; Ivan Ponce; Jorge Fernandez; Karen Orostica; Marcelo Rojas; Matias Pezoa; Patricia Bustos; Rodrigo Fasce
EPI_ISL_12954183	Good Sherperd Hospital	National Institute for Communicable Diseases of the National Health Laboratory Service	Amoako DG; Bhiman JN; Everatt J; Ismail A; Kekana D; Mahlangu B; Maphalala G; Mnguni A; Mohale T; Ntuli N; Scheepers C; Wolter N
EPI_ISL_12475182, EPI_ISL_12475185	HOSPITAL UNIVERSITARIO SON ESPASES	HOSPITAL UNIVERSITARIO SON ESPASES	Dr. Antonio Oliver; Dr. Carla López-Causapé; Dr. Gabriel Cabot; Hospital Universitario Son Espases; on behalf of Servicio de Microbiología
EPI_ISL_12339502	Haaglanden Medisch Centrum	Leiden University Medical Center	Stefan Boers
EPI_ISL_12638056, EPI_ISL_12740321, EPI_ISL_12786084, EPI_ISL_12786412	Helix	Centers for Disease Control and Prevention Division of Viral Diseases, Pathogen Discovery	Benjamin Rambo-Martin; Christopher Gulvick; Clinton Paden; Dakota Howard; Dhwani Batra; Duncan MacCannell; Erisa Sula; Helix CA; Jason Caravas; Kristine Lacek; Matthew Schmerer; Peter Cook; Scott Sammons; Shatavia Morrison; Tymeckia Kendall; Victoria Caban Figueroa; Yvette Unoarumhi
EPI_ISL_12605801	Histopath	NSW Health Pathology - Institute of Clinical Pathology and Medical Research; Westmead Hospital; University of Sydney	Arnott A.; Draper J.; Gall M.; Martinez E.; Rockett R.; Sintchenko V.; on behalf of ICPMR
EPI_ISL_12582288, EPI_ISL_13372671	Hopital	National Reference Center for Viruses of Respiratory Infections, Institut Pasteur, Paris	Angela Brisebarre; Aurélie GUIGON; Camille Capel; Christophe Malabat; Corinne Maufrais; Etienne Simon-Lorière; Frédéric Lemoine; Julien Fumey; Louise Lefrançois; Marion Barbet; Maud Vanpeene; Méline Bizard; Slim El Khiari; Sylvie Behillil; Sylvie Van der Werf; Vincent Enouf
EPI_ISL_12703161	Hospital Center Luxembourg	Laboratoire national de sante, Microbiology, Microbial Genomics Platform	Anke Wienecke-Baldacchino; Catherine Ragimbeau; Elodie Solarino; Eric Hugoson; Fatu Djabi; Jessica Tapp; Lise Pignon; Raoul Salmon; Sibel Berger; Tamir Abdelrahman; Trung Nguyen Nguyen; Virginie Jover
EPI_ISL_12687970	Hospital General Universitario Gregorio Marañón	Hospital General Universitario Gregorio Marañón	Cristina Rodriguez-Grande; Daniel Peñas Utrilla; Darío García de Viedma; Jorge Rodríguez-Grande; Julia Suárez; Laura Pérez-Lago; Marta Herranz Martin; Patricia Muñoz; Pedro Sola Campoy; Pilar Catalán; Rosalía Palomino Cabrera; Sergio Buenestado Serrano
EPI_ISL_12717878, EPI_ISL_13014083	Institute for Infectious Diseases	Institute for Infectious Diseases, University of Bern	Alban Ramette; Christian Baumann; Cora Sägesser; Franziska Suter-Riniker; Lea Stauber; Loïc Borcard; Miguel A Terrazos Miani; Pascal Bittel; Peter Keller; Sonja Gempeler; Stefan Neuenschwander; Stephen L Leib
EPI_ISL_12483994	Jessa	Jessa	Rita Smets et al. on behalf of the Jessa_cmdLab
EPI_ISL_12963450, EPI_ISL_12965004	Kaiser Permanente Southern California	Helix	Helix; Kaiser Permanente Southern California
EPI_ISL_12730831, EPI_ISL_12749785	LABM SAINT BENOIT	Laboratoire de virologie, CNR arbovirus Associé, Chu de la Réunion	Anne-Julie Gourdé; Etienne Frumence; Marie-Christine Jaffar Bandjee; Nicolas M'nemosyme; Nicolas Traversier; Rubens Lhonneur
EPI_ISL_11994300	LABM VIALATTE ESPACE SANTE	CERBA HealthCare	Bénédicte Roquebert; Laura Verdurme; Mathilde Roussel; Sabine Trombert; Stéphanie Haim-Boukobza
EPI_ISL_12755936	LABORATOIRE BIO-VAL	CNR Virus des Infections Respiratoires - France SUD	Antonin Bal; Bruno Lina; Bruno Simon; Gregory Destras; Gwendolyne Burfin; Hadrien Regue; Laurence Josset; Martine Valette; Quentin Semanas; Theophile Boyer
EPI_ISL_12699259, EPI_ISL_12755669	LABORATOIRE NOVELAB	CNR Virus des Infections Respiratoires - France SUD	Antonin Bal; Bruno Lina; Bruno Simon; Gregory Destras; Gwendolyne Burfin; Hadrien Regue; Laurence Josset; Martine Valette; Quentin Semanas; Theophile Boyer
EPI_ISL_13321776	LPA BESANCON	Department of Virology, Henri Mondor University Hospital, Assistance Publique Hôpitaux de Paris, Université Paris-Est Créteil, INSERM U955	Alexandre Soulier; Christophe Rodriguez; Elisabeth Trawinski; Guillaume Gricourt; Jean-Michel Pawlotsky; Melissa N'Debi; Slim Fourati; Vanessa Demontant

EPI_ISL_13017778	Labo Analyses Med	Institut Pasteur	Angela Brisebarre; Camille Capel; Christophe Malabat; Corinne Maufrais; Domitille LEMAN; Etienne Simon-Lorière; Frédéric Lemoine; Julien Fumey; Louise Lefrançois; Marion Barbet; Maud Vanpeene; Méline Bizard; Slim El Khiari; Sylvie Behillil; Sylvie Van der Werf; Vincent Enouf
EPI_ISL_12714440	Labo Analyses Med - Ocealab - Le Tenerio	National Reference Center for Viruses of Respiratory Infections, Institut Pasteur, Paris	Angela Brisebarre; Camille Capel; Christophe Malabat; Corinne Maufrais; Etienne Simon-Lorière; Frédéric Lemoine; Julien Fumey; Karine MICHEZ; Louise Lefrançois; Marion Barbet; Maud Vanpeene; Méline Bizard; Slim El Khiari; Sylvie Van der Werf; Vincent Enouf
EPI_ISL_13002482	Labor Dr. Spranger	Robert Koch Institute	
EPI_ISL_13014545	Labor ZOTZ|KLIMAS; MVZ Düsseldorf-Centrum	Robert Koch Institute	
EPI_ISL_12907221	Laboratoire MAYMAT	Department of Virology, Henri Mondor University Hospital, Assistance Publique Hôpitaux de Paris, Université Paris-Est Créteil, INSERM U955	Alexandre Soulier; Christophe Rodriguez; Elisabeth Trawinski; Guillaume Gricourt; Jean-Michel Pawlotsky; Melissa N'Debi; Slim Fourati; Vanessa Demontant
EPI_ISL_12295715, EPI_ISL_12914362, EPI_ISL_12914364, EPI_ISL_13055526	Laboratoire de santé publique du Québec	Laboratoire de santé publique du Québec	Guillaume Bourque; Ioannis Ragoussis; Jesse Shapiro; Mark Lathrop and Judith Fafard on behalf of the CoVSeQ research group; Sandrine Moreira
EPI_ISL_12837461, EPI_ISL_12837532	Laboratoire national de sante, Microbiology, Virology	Microbiology, Microbial Genomics Platform, LNS Laboratoire National De Santé	Anke Wienecke-Baldacchino; Catherine Ragimbeau; Elodie Solarino; Eric Hugoson; Fatu Djabi; Jessica Tapp; Lise Pignon; Raoul Salmon; Sibel Berger; Tamir Abdelrahman; Trung Nguyen Nguyen; Virginie Jover
EPI_ISL_13031783	Laboratoires Reunis	Laboratoire national de sante, Microbiology, Microbial Genomics Platform	Anke Wienecke-Baldacchino; Bernard Weber; Catherine Ragimbeau; Elodie Solarino; Eric Hugoson; Fatu Djabi; Jessica Tapp; Lise Pignon; Raoul Salmon; Sibel Berger; Tamir Abdelrahman; Virginie Jover
EPI_ISL_12837868, EPI_ISL_12837880	Laboratoires Reunis	Microbiology, Microbial Genomics Platform, LNS Laboratoire National De Santé	Anke Wienecke-Baldacchino; Bernard Weber; Catherine Ragimbeau; Elodie Solarino; Eric Hugoson; Fatu Djabi; Jessica Tapp; Lise Pignon; Raoul Salmon; Sibel Berger; Tamir Abdelrahman; Virginie Jover
EPI_ISL_12512307, EPI_ISL_12512309	Laboratorio CQRC	CQRC_QUALITY CONTROL CHEMICAL BIOLOGICAL RISK_AOOR Villa Sofia Cervello Palermo	Broccolo F.; Brunacci G.; Contino F.; Di Gaudio F.
EPI_ISL_12169006, EPI_ISL_12169353, EPI_ISL_12756256, EPI_ISL_12757576	Laboratory Corporation of America	Centers for Disease Control and Prevention Division of Viral Diseases, Pathogen Discovery	Amanda Douglas; Amanda Suchanek; Andrea Throop; Ayla Burns; Benjamin Rambo-Martin; Bobbi Croy; Brian Krueger; Brian Norvell; Christopher Gulvick; Christos Petropoulos; Clinton Paden; Craig Lukasik; Dakota Howard; Debbie Boles; Dhwani Batra; Duncan MacCannell; Eyad Almasri; Goran Stevovic; Howard Engler; Hrushikesh Deshmukh; Jake Humphrey; Jana Schroth; Jason Caravas; Joe Voshell; John Pruitt; Jonathan Meltzer; Jonathan Williams; Kimberly Wagner; Kristine Lacek; Lax Iyer; Lisa Pfefferle; Lyndon Tilson; Manoj Jain; Marcia Eisenberg; Mary Cristobal; Mary Williamson; Matthew Robinson; Matthew Schmerer; Michael Levandoski; Mike Sapeta; Mindy Nye; Minoo Agarwal; Mohan Kolli; Nuthawin Charoensri; Oren Cohen; Peter Cook; Prashant Gupta; Qian Zeng; Rama Ghatti; Scott Parker; Scott Ryan; Scott Sammons; Shatavia Morrison; Stanley Letovsky; Steven Ragan; Suresh Selvaraju; Susan Countryman; Susan Hicks; Suzanne Dale; Thomas Urban; Tim Kuphal; Tricia Zwiefelhofer; Tymeckia Kendall; Victoria Caban Figueroa; Vincent Drouillon; Yvette Unoarumhi
EPI_ISL_11984862, EPI_ISL_12292992, EPI_ISL_12293148, EPI_ISL_12293149, EPI_ISL_12293160, EPI_ISL_12293161, EPI_ISL_12293281, EPI_ISL_12293283, EPI_ISL_12293292, EPI_ISL_12472109, EPI_ISL_12472111, EPI_ISL_12472129, EPI_ISL_12472136, EPI_ISL_12610583, EPI_ISL_12610598, EPI_ISL_12610609			
see above	Lifebrain Covid Labor GmbH	Lifebrain Covid Labor GmbH	Abhishek Mitra; Alexandra Wagner; Anna Edermayr; Felix Valentin Spiegel; Filip Sima; Florian Scharhauser; Hannes Hagen; Kristina Bavrka Kolenc; Lucia Castello; So Jung Han
EPI_ISL_11604519, EPI_ISL_12478907, EPI_ISL_12482009, EPI_ISL_12515844, EPI_ISL_12515952, EPI_ISL_12548001, EPI_ISL_12550282, EPI_ISL_12553282, EPI_ISL_12555769, EPI_ISL_12570336, EPI_ISL_12630820, EPI_ISL_12630919, EPI_ISL_12630959, EPI_ISL_12647695, EPI_ISL_13130112			
see above	Lighthouse Lab in Glasgow	Wellcome Sanger Institute for the COVID-19 Genomics UK (COG-UK) Consortium	Anna Dominiczak and Alex Alderton; Carol Clugston; Cordelia Langford; David Gray; David K. Jackson; Dominic Kwiatkowski; Ewan Harrison; Harper VanSteenhouse; Ian Johnston; Jeffrey Barrett; John Sillitoe on behalf of the Wellcome Sanger Institute COVID-19 Surveillance Team; Roberto Amato; Sonia Goncalves; Yumi Kasai
EPI_ISL_12675976	Limbach - MVZ Clotten Labor Freiburg Labor Dr. Haas Dr. Raif & Kollegen GbR	Robert Koch Institute	
EPI_ISL_12671280	Limbach - MVZ Labor Dr. Volkmann & Kollegen	Robert Koch Institute	
EPI_ISL_13003774	Limbach - MVZ Labor Ravensburg Labor Dr. Gärtner	Robert Koch Institute	
EPI_ISL_12821530	Limbach - MVZ Labor Westmecklenburg Schmudlach-Oswald-Kettermann & Kollegen	Robert Koch Institute	
EPI_ISL_12755245, EPI_ISL_12755263, EPI_ISL_12892789	MIRIALIS CLUSES BECHET	CNR Virus des Infections Respiratoires - France SUD	Antonin Bal; Bruno Lina; Bruno Simon; Gregory Destras; Gwendolyne Burfin; Hadrien Regue; Laurence Josset; Martine Valette; Quentin Semanas; Theophile Boyer
EPI_ISL_12733100	MVZ Labor Dr. Limbach & Kollegen GbR	Robert Koch Institute	
EPI_ISL_12844498	Max von Pettenkofer Institute, Virology, National Reference Center for Retroviruses, LMU Munich	Laboratory for Functional Genome Analysis (LAFUGA), Gene Center of the LMU Munich	Alexander Graf; Helmut Blum; Max Muenchhoff; Oliver Keppler; Stefan Krebs
EPI_ISL_12954184	Mbabane Government Hospital	National Institute for Communicable Diseases of the National Health Laboratory Service	Amoako DG; Bhiman JN; Everatt J; Ismail A; Kekana D; Mahlangu B; Maphalala G; Mnguni A; Mohale T; Ntuli N; Scheepers C; Wolter N
EPI_ISL_12954185, EPI_ISL_12954186	Mbabane Public Health Unit	National Institute for Communicable Diseases of the National Health Laboratory Service	Amoako DG; Bhiman JN; Everatt J; Ismail A; Kekana D; Mahlangu B; Maphalala G; Mnguni A; Mohale T; Ntuli N; Scheepers C; Wolter N
EPI_ISL_12767686	Michigan Department of Health and Human Services, Bureau of Laboratories	Michigan Department of Health and Human Services, Bureau of Laboratories	Blankenship HM; Riner D; Soehnlen MK
EPI_ISL_12628241	Microbiologia CATLAB	Can Ruti SARS-CoV-2 Sequencing Hub (HUGTiP/IrsiCaixa/IGTP)	Alexia París; Ana Blanco; Andreu Coello; Antoni E Bordoy; Bonaventura Clotet; David Panisello; Francesc Catala-Moll; Gemma Clara; Ignacio Blanco; Laia Soler; Marc Noguera-Julian; Montserrat Giménez; Pere-Joan Cardona; Pilar Armengol; Roger Paredes; Sara González; Verónica Saludes; and Elisa Martró on behalf of the Can Ruti SARS-CoV-2 Sequencing Hub
EPI_ISL_12979827, EPI_ISL_12979841	Microbiological Diagnostic Unit - Public Health Laboratory (MDU-PHL)	Microbiological Diagnostic Unit - Public Health Laboratory (MDU-PHL)	Horan, K.; N.L.; Seemann, T.; Sherry
EPI_ISL_12849528	Microbiological Diagnostic Unit - Public Health Laboratory (MDU-PHL), The Peter Doherty Institute for Infection and Immunity	Microbiological Diagnostic Unit - Public Health Laboratory (MDU-PHL), The Peter Doherty Institute for Infection and Immunity	Horan, K.; N.L.; Seemann, T.; Sherry
EPI_ISL_12850230	Microbiological Diagnostic Unit - Public Health Laboratory (MDU-PHL), The Peter Dororthy Institute for Infection and Immunity	Microbiological Diagnostic Unit - Public Health Laboratory (MDU-PHL), The Peter Dororthy Institute for Infection and Immunity	Horan, K.; N.L.; Seemann, T.; Sherry
EPI_ISL_12628235, EPI_ISL_12628250, EPI_ISL_12628267, EPI_ISL_12628303	Microbiology Department, Laboratori Clínic Metropolitana Nord. Hospital Universitari Germans Trias i Pujol	Can Ruti SARS-CoV-2 Sequencing Hub (HUGTiP/IrsiCaixa/IGTP)	Alexia París; Ana Blanco; Andreu Coello; Antoni E Bordoy; Bonaventura Clotet; David Panisello; Francesc Catala-Moll; Gemma Clara; Ignacio Blanco; Laia Soler; Marc Noguera-Julian; Montserrat Giménez; Pere-Joan Cardona; Pilar Armengol; Roger Paredes; Sara González; Verónica Saludes; and Elisa Martró on behalf of the Can Ruti SARS-CoV-2 Sequencing Hub
EPI_ISL_12642823	Microbiology Department. Complexo Hospitalario Universitario de Vigo	Microbiology Department. Complexo Hospitalario Universitario de Vigo	Microbiology Department. Complexo Hospitalario Universitario de Vigo
EPI_ISL_12845550	National Health Laboratory Services	CERI, Centre for Epidemic Response and Innovation, Stellenbosch University and KRISP, KZN Research Innovation and Sequencing Platform, UKZN.	Anyaneji UJ; Giandhari J; Maharaj A; Moir M; Naidoo Y; Nokukhanya Mdlalose; Pillay S; San JE; Sanko TJ; Tegally H; Tshiabuila D; Van Wyk S; Wilkinson E; de Oliveira T
EPI_ISL_12043264, EPI_ISL_12474407	National Health Laboratory Services, Virology	National Health Laboratory Services, Virology	Ashlyn S. C. Davis; Florette K. Treurnicht; Kathleen Subramoney; Nkhensani Mtileni
EPI_ISL_12252953	National Platform bis UMONS / Jolimont	National Platform bis UMONS / Jolimont	Caroline Debecker; Clothilde Claus; Eric Tarantino; Florian Juszczak; Gautier Detry; Laetitia Gheysen; Ruddy Wattiez
EPI_ISL_12533200, EPI_ISL_12835584	National Platform bis UMONS/Jolimont	National Platform bis UMONS/Jolimont	Caroline Debecker; Clothilde Claus; Eric Tarantino; Florian Juszczak; Gautier Detry; Laetitia Gheysen; Ruddy Wattiez
EPI_ISL_12647216, EPI_ISL_12647217, EPI_ISL_12689375	National Public Health Laboratory, National Centre for Infectious Diseases	National Public Health Laboratory, National Centre for Infectious Diseases	BeiBei Chen; Benny Yeo; Chen Shi Ling; Grace Ngan; Jesslin Tan; Lin Cui; Raymond Tzer Pin Lin; Royce Ang; Samuel Loo; Yichen Ding; Zhenyang Zhou

EPI_ISL_13186295, EPI_ISL_13186587, EPI_ISL_13280019, EPI_ISL_13280073, EPI_ISL_13280158, EPI_ISL_13280186, EPI_ISL_13280229, EPI_ISL_13280310, EPI_ISL_13280312			
see above	National Virus Reference Laboratory	National Virus Reference Laboratory	Charlene Bennett; Cillian F De Gascun; Gabriel Gonzalez; Jonathan Dean; Michael Carr; Zoe Yandle
EPI_ISL_12623503	New Brunswick - Vitalite Health Network	New Brunswick - Vitalite Health Network	Allain E.; Chacko S.; Crapoulet N.; Desnoyers G.; Garceau R.; Lacroix J.; Lyons P.; Shaw W.
EPI_ISL_12841626	New Brunswick - Vitalite Health Network, Dr. Georges-L.-Dumont University Hospital Centre	New Brunswick - Vitalite Health Network, Dr. Georges-L.-Dumont University Hospital Centre	Allain E.; Chacko S.; Crapoulet N.; Desnoyers G.; Garceau R.; Lacroix J.; Lyons P.; Shaw W.
EPI_ISL_13079038	Noble Hospital Samples	INSACOG-IISER Pune	; Aurnab Ghose; Joy Merwin Monteiro; Krishanpal Karmodiya
EPI_ISL_12853572, EPI_ISL_12853587, EPI_ISL_12895004, EPI_ISL_12895028	Originating lab: Wales Specialist Virology Centre Sequencing lab: Pathogen Genomics Unit	Public Health Wales Microbiology Cardiff Wales Specialist Virology Centre	Alec Birchley; Alexander Adams; Amy Gaskin; Angela Marchbank; Bree Gatica-Wilcox; Catherine Moore; Jason Coombes; Joanne Watkins; Joel Southgate; Johnathan Evans; Laura Gifford; Lauren Gilbert; Lee Graham; Malorie Perry; Matthew Bull; Nicole Pacchiarini; Sally Corden; Sara Kumziene-Summerhayes; Sara Rey; Sarah Taylor; Simon Cottrell; Sophie Jones; Tom Connor
EPI_ISL_13010930, EPI_ISL_13010931	Outre Mer	National Reference Center for Viruses of Respiratory Infections, Institut Pasteur, Paris	Angela Brisebarre; Camille Capel; Christophe Malabat; Corinne Maufrais; Didier MATTERA; Etienne Simon-Lorière; Frédéric Lemoine; Julien Fumey; Louise Lefrançois; Marion Barbet; Maud Vanpeene; Méline Bizard; Slim El Khiari; Sylvie Van der Werf; Vincent Enouf
EPI_ISL_13048417, EPI_ISL_13048521	PORT ELIZABETH LABORATORY	National Institute for Communicable Diseases of the National Health Laboratory Service	Amoako DG; Bhiman JN; Everatt J; Ismail A; Kekana D; Mahlangu B; Mnguni A; Mohale T; Ntuli N; Scheepers C; Wolter N
EPI_ISL_12012887	PathCare, Cape Town	Division of Medical Virology, National Health Laboratory Service (NHLS), Tygerberg Hospital / Stellenbosch University	Gert van Zyl; Jean Maritz; Nadine Cronje; Petra Raimond; Shannon Wilson; Tongai Maponga; Wolfgang Preiser
EPI_ISL_12626263, EPI_ISL_12626309, EPI_ISL_12770929, EPI_ISL_12911598	PathWest Laboratory Medicine WA	PathWest Laboratory Medicine WA Microbial Surveillance Unit	PathWest Laboratory Medicine WA Microbial Surveillance Unit
EPI_ISL_12871857	Pathcare	CERI, Centre for Epidemic Response and Innovation, Stellenbosch University and KRISP, KZN Research Innovation and Sequencing Platform, UKZN.	Anyaneji UJ; Claassen M; Giandhari J; Maharaj A; Maponga T; Moir M; Naidoo Y; Pillay S; Preiser W; San JE; Sanko TJ; Stander T; Tegally H; Tshiabuila D; Van Wyk S; Wilkinson E; Wilson S; de Oliveira T; van Zyl G
EPI_ISL_12905595	Pathologist Lancet Kenya	KEMRI-Wellcome Trust Research Programme,Kilifi	Agoti C.; D.J.Nokes; Githinji G.; Lambisia A.; Makori T.; Mburu M.W.; Mohamed K.S.; Morobe J.; Mukadam R; Munoko A.; Ndwiga L.; Ngari C.; Ochola I; Ongera E.; de Laurent Z.
EPI_ISL_12605042, EPI_ISL_12605043	Pathology North - Royal North Shore Hospital - NSW Health Pathology	NSW Health Pathology - Institute of Clinical Pathology and Medical Research; Westmead Hospital; University of Sydney	Arnott A.; Draper J.; Gall M.; Martinez E.; Rockett R.; Sintchenko V.; on behalf of ICPMR
EPI_ISL_12613687, EPI_ISL_12648066	Plateforme de testing Namuroise	Plateforme de testing Namuroise	Bleret Leonore; Degosserie Jonathan; Denis Olivier; Gilliard Nicolas; Lesly Nyinkeu Kemamen; Louise Janssens; Maschietto Céline; Mullier François; Otto Gaetan; Pellet Nathan; Renguet Edith
EPI_ISL_12683708	Platform BIS UZA/UAntwerpen	Labo Klinische Biologie, UZA	Basil Britto Xavier; Christine Lammens; Herman Goossens; Ines Verbesselt; Jasmine Coppens; Kathleen Holemans; Marie Le Mercier; Silke Liers; Veerle Matheeussen
EPI_ISL_12571627, EPI_ISL_12715870, EPI_ISL_12715916, EPI_ISL_12715923	Public Health Laboratory, Public Health Service Amsterdam, The Netherlands	Department of Medical Microbiology & Infection prevention, Amsterdam University Medical Centers location AMC	Akke Cornelissen; Fokla Zorgdrager; Janke Schinkel; Jelle Koopsen; Judith den Uil; Marcel Jonges; Matthijs Welkers; Menno de Jong; Robin van Houdt; Sebastien Matamoros; Sjoerd Rebers; Sylvia Bruisten; Tjalling Leenstra and Mariken van der Lubben on behalf of the Amsterdam Regional Genomic epidemiology and Outbreak Surveillance (ARGOS) consortium
EPI_ISL_13149254, EPI_ISL_13149259, EPI_ISL_13149261, EPI_ISL_13149273, EPI_ISL_13149279, EPI_ISL_13149280, EPI_ISL_13149284, EPI_ISL_13149291, EPI_ISL_13149293, EPI_ISL_13149297, EPI_ISL_13149303, EPI_ISL_13149305, EPI_ISL_13149318			
see above	Public Health Laboratory: COVID-19 Lab	International Livestock Research Institute	Collins Muli; Daniel Ouso; Edward Kiritu; Edward O. Abworo; Gilbert Kibet; Gugu Maphalala; Mncedisi Hlophe; Nomcebo Phungwayo; Patrick Amoth; Paul Dobi; Samuel O. Oyola; Shebbar Osiany; Siphesihle Langwenya; Sonal P. Henson; Susan Kamalizeni; Vishvanath Nene
EPI_ISL_12266791, EPI_ISL_12466376, EPI_ISL_12627205	Public Health Ontario Laboratory	Public Health Ontario Laboratory	Aimin Li; Alex Marchand-Austin; Andre Villegas; Anna Puzinovici; Ashleigh Sullivan; Brandon Ye; Candice Schreiber; Carla Duncan; Christina Rampertab; Christine Seah; Claudia Chu; Dean Maxwell; Dhiraj Gaglani; Doonia Bajovic; Esther Nagai; Fatemeh Shaeri; Fatima Merza; Grace Jeong; Hadia Hussain; Himeshi Samarsinghe; Jacob Afelskie; Jason Iraheta; Jesse Wang; John Palmer; Karthikeyan Sivaraman; Kirby Cronin; Lisa Kim; Lisa McTaggart; Maria Mariscal; Mark Horsman; Narisha Shakuralli; Nataliya Potapova; Natasha Sing; Nobish Varghese; Philip Banh; Rachelle DiTullio; Rebecca Azzaro; Rima Palencia; Samir N Patel; Sarah Teatero; Semra Tibebu; Sophie Yu; Surendra Kumar; Sushma Kavikondala; Vincent Su Bin Cha; Zarah Rajaei
EPI_ISL_12932497	Queensland Medical Laboratories	PHV-FSS	Chenwei Wang on behalf of Q-PHIRE Genomics
EPI_ISL_12660069, EPI_ISL_12874244, EPI_ISL_12874769, EPI_ISL_13056807	Quest Diagnostics Incorporated	Centers for Disease Control and Prevention Division of Viral Diseases, Pathogen Discovery	A. Gerasimova; A. Perez; B. Anderson; Benjamin Rambo-Martin; Christopher Gulvick; Clinton Paden; Dakota Howard; Dhwani Batra; Duncan MacCannell; Erisa Sula; F. Lacbawan; I. Shlyakhter; Jason Caravas; K. Livingston; Kristine Lacek; L. Bernstein; M. Hua; Matthew Schmerer; P. Tanpaiboon; Peter Cook; R. Kagan; R. Owen; R. Rolando; S. Rosenthal; Scott Sammons; Shatavia Morrison; Tymeckia Kendall; Victoria Caban Figueroa; Y. Liu; Yvette Unoarumhi
EPI_ISL_13089278	ROMILLY DYNALAB	Department of Virology, Henri Mondor University Hospital, Assistance Publique Hôpitaux de Paris, Université Paris-Est Créteil, INSERM U955	Alexandre Soulier; Christophe Rodriguez; Elisabeth Trawinski; Guillaume Gricourt; Jean-Michel Pawlotsky; Melissa N'Debi; Slim Fourati; Vanessa Demontant
EPI_ISL_12704014	Regional Virus Laboratory, Belfast Health and Social Care Trust; and: Genomics Core Technology Unit, Queen's University Belfast.	COVID-19 Genomics UK (COG-UK) Consortium	Alan; Alison Watt; Arun Mahesh; BHSCT]: Conall McCaughey; Ciara Cox; Clara Radulescu; David Simpson; Deborah Lavin; Derek Fairley; Evan Troendle; Fiona Rogan; James McKenna; Jana Gazdova; Julia Miskelly; Mairead Connor; Miao Tang; QUB]: Marc Fuchs; Rice; Sarah Sonner; Stephen Bridgett; Susan Feeney; Syed Umbreen; Tanya Curran; Timofey Skvortsov; Zoltan Molnar; [Genomics Core Technology Unit; [Regional Virus Laboratory
EPI_ISL_12152675, EPI_ISL_12241466, EPI_ISL_12469666, EPI_ISL_12469747, EPI_ISL_12469892, EPI_ISL_12470002, EPI_ISL_12514770, EPI_ISL_12515007, EPI_ISL_12515119, EPI_ISL_12606310, EPI_ISL_12606668, EPI_ISL_12606838, EPI_ISL_12606881, EPI_ISL_12606913, EPI_ISL_12667094, EPI_ISL_12694146, EPI_ISL_12698346			
see above	Rosalind Franklin Laboratory	Wellcome Sanger Institute for the COVID-19 Genomics UK (COG-UK) Consortium	Cordelia Langford; David K. Jackson; Dominic Kwiatkowski; Donald Fraser; Ewan Harrison; Ian Johnston; Jeffrey Barrett; John Sillitoe on behalf of the Wellcome Sanger Institute COVID-19 Surveillance Team; Rob Howes; Roberto Amato; Sonia Goncalves; Suki Lee; The Rosalind Franklin Laboratory and Alex Alderton
EPI_ISL_12442764, EPI_ISL_13045384, EPI_ISL_13045388, EPI_ISL_13045498, EPI_ISL_13045602, EPI_ISL_13045673, EPI_ISL_13045755			
see above	SA Pathology	SA Pathology	Caitlin Selway; Chuan Kok Lim; Ivan Bastian; Lex Leong; Mark Turra
EPI_ISL_12724716, EPI_ISL_12807071, EPI_ISL_13026924	SARS-CoV-2 testing team, National Institute of Infectious Diseases	Pathogen Genomics Center, National Institute of Infectious Diseases	Hazuka Y Furihata; Kentaro Itokawa; Makoto Kuroda; Masanori Hashino; Masumichi Saito; Naomi Nojiri; Nozomu Hanaoka; Rina Tanaka; Tsuguto Fujimoto; Tsuyoshi Sekizuka
EPI_ISL_12959393	SD Public Health Laboratory	Centers for Disease Control and Prevention Division of Viral Diseases, Pathogen Discovery	Alex Burgin; Ben Rambo-Martin; Clinton Paden; Dakota Howard; Dave Wentworth; Dhwani Batra; Jasmine Padilla; Joseph Madden; Justin Lee; Kristen Knipe; Kristine Lacek; Mark Burroughs; Matthew Schmerer; Meghan Bentz; Mili Sheth; Peter Cook; Sam Shepard; Sarah Nobles; Vivien Dugan; Yvette Unoarumhi
EPI_ISL_12856291	SELAS BC-LAB	Department of Virology, Henri Mondor University Hospital, Assistance Publique Hôpitaux de Paris, Université Paris-Est Créteil, INSERM U955	Alexandre Soulier; Christophe Rodriguez; Elisabeth Trawinski; Guillaume Gricourt; Jean-Michel Pawlotsky; Melissa N'Debi; Slim Fourati; Vanessa Demontant
EPI_ISL_12335014	SYNLAB MVZ Leverkusen	Robert Koch Institute	
EPI_ISL_12520814, EPI_ISL_12674190, EPI_ISL_12733204	SYNLAB MVZ Weiden	Robert Koch Institute	
EPI_ISL_12871407, EPI_ISL_12877262, EPI_ISL_12877455, EPI_ISL_12902154	Shamir Medical Center (Asaf Harofe)	Shamir Medical Center (Asaf Harofe)	Abu Hamad Ramzia; Adina Bar Chaim; Anna Vishnevsky; Chen Weiner; Nir Rainy; Patricia Benveniste-Lekovitz; Reut Sorek Abramovich; Yevgeni Yegorov
EPI_ISL_13248991	Sonic - Labor Staber Dresden (Klipphausen)	Robert Koch Institute	
EPI_ISL_12605102	St Vincent's Pathology (SydPath)	NSW Health Pathology - Institute of Clinical Pathology and Medical Research; Westmead Hospital; University of Sydney	Arnott A.; Draper J.; Gall M.; Martinez E.; Rockett R.; Sintchenko V.; on behalf of ICPMR
EPI_ISL_12587465	Stadtspital Triemli	Institute of Medical Virology, University of Zurich	Alexandra Trkola; Annette Audigé; Catharine Aquino; Cyril Shah; Daniel Ehrsam; Gabriela Ziltener; Guido Bloemberg; Hubert Rehrauer; Isabel Stürmer; Joel Wirz; Jon Huder; Jürg Böni; Kevin Steiner; Maria Grünberg; Maryam Zaheri; Michael Huber; Riccarda Capaul; Stefan Schmutz; Verena Kufner; Weihong Qi
EPI_ISL_12785355	State Public Health Laboratory O/o DPH&PM, Chennai	CSIR-NEERI, Nagpur Covid-19 Testing Lab	Krishna Khairnar et al.
EPI_ISL_13184869,	Swedish national genomic	The Public Health Agency of Sweden	Alma Brolund; Emmi Andersson; Maria Lind Karlberg; Swedish national genomic surveillance program of SARS-CoV-2

EPI_ISL_13185323, EPI_ISL_13185598	surveillance program of SARS-CoV-2		
EPI_ISL_12898417	Switch Health	National Microbiology Laboratory (NML)	Adrian Zetner; Anna Majer; Anneliese Landgraff; CanCOGeN's metadata curation team; Carmen Lia Murall; Chanchal Yadav; Connor Chato; Darian Hole; Elsie Grudeski; Emily Haidl; Gary Van Domselaar; Gordon Jolly; Grace Seo; Jeff Tuff; Jennifer Tanner; Katherine Eaton; Kirsten Biggar; Kristyn Burak; Madison Chapel; Morag Graham; Natalie Knox; Nathalie Bastien; Philip Mabon; Public Health Agency of Canada's CCGP and Scientific Informatics Services team; Rhiannon Huzarewich; Russell Mandes; Shari Tyson; Timothy Booth; Yan Li
EPI_ISL_13029523	Synlab Eesti OÜ	1. Laboratory of Communicable Diseases (Estonia); 2. Eurofins Genomics Europe Sequencing GmbH	Abroi A.; Avi R.; Dotsenko L.; Epštein J.; Hoidmets D.; Huik K.; Härma M-A.; Jaaniso E.; Kaarna K.; Kallas E.; Koppel I.; Kuzmin I.; Lahesaare A.; Lutsar I.; Metspalu M.; Milani L.; Naaber P.; Niglas H.; Oopkaup O.E.; Pauskar M.; Peterson H.; Päll T.; Ratnik K.; Raudvere U.; Reisberg T.; Sadikova O.; Sepp H.; Shablinskaja A.; Suija H.; Talas U.G.; Truusalu K.
EPI_ISL_13207909	Taksin hospital	Medical Genomic Centre,Medical Life Sciences Institute,Department of Medical Sciences, Ministry of Public Health, Thailand	Archawin Rojanawiwat; Jirapha Pakdee; Naphatcha Thawong; Natthakul Bunneang; Nuanjun Wichukchinda; Pilailuk Akkapaiboon Okada; Pundharika Piboonsiri; Surakameth Mahasirimongkol; Waritta Sawaengdee
EPI_ISL_12771764, EPI_ISL_12771765	Temporary Specimen Collection Centre at the AsiaWorld-Expo	Hong Kong Department of Health	Alan K.L. Tsang; Edman T.K. Lam; Ken H.L. Ng; Patricia K. L. Leung; Peter C.W. Yip; Rickjason C.W. Chan
EPI_ISL_12607382, EPI_ISL_12607392, EPI_ISL_12607398, EPI_ISL_12607412, EPI_ISL_12749708	U.O. Microbiologia Laboratorio Unico Centro Servizi - AUSL della Romagna	U.O. Microbiologia, Laboratorio Unico Centro Servizi - AUSL della Romagna	Giorgio Dirani
EPI_ISL_12252797	UMC Utrecht	UMC Utrecht	Anne Wensing; Joris Schoonderwoerd; Rob Schuurman
EPI_ISL_13330459	UNILIANS BIOGROUP Décines	Department of Virology, Henri Mondor University Hospital, Assistance Publique Hôpitaux de Paris, Université Paris-Est Créteil, INSERM U955	Alexandre Soulier; Christophe Rodriguez; Elisabeth Trawinski; Guillaume Gricourt; Jean-Michel Pawlotsky; Melissa N'Debi; Slim Fourati; Vanessa Demontant
EPI_ISL_12529661	UO Microbiologia, IRCCS Az.Ospedaliero-Universitaria di Bologna, Policlinico di S.Orsola	Unità Operativa di Microbiologia, IRCSS Policlinico di Sant'Orsola, Azienda Ospedaliero Universitaria di Bologna	Giada Rossini
EPI_ISL_12696584	USC Clinical Lab	Los Angeles County Public Health Laboratories	J. Garrigues et al.
EPI_ISL_12956393	University Hospitals of Geneva, Laboratory of Virology	HUG, Laboratory of Virology and the Health2030 Genome Center	Aline Mamin; Ana Rita Goncalves; Cedric Howald; Deborah Penet; Francisco Perez; Henri Pegeot; Ioannis Xenarios; Keith Harshman; Laurent Kaiser; Lorenzo Cerutti; Melyssa Elies; Samuel Cordey
EPI_ISL_13050826	Usansolo-Galdakao University Hospital	Usansolo-Galdakao University Hospital	Ana Gual-de-Torrella; Izaskun Alejo-Cancho; Mikel Urrutikoetxea-Gutierrez
EPI_ISL_12130073, EPI_ISL_12340151, EPI_ISL_12846026	Viollier AG	Department of Biosystems Science and Engineering, ETH Zürich	Andrea Patrizia Salzmann; Chaoran Chen; Christian Beisel; Christiane Beckmann; Christoph Noppen; David Dreifuss; Elodie Burcklen; Franziska Singer; Henriette Kurth; Ina Nissen; Ivan Topolsky; Kim Philipp Jablonski; Lara Fuhrmann; Louis du Plessis; Matteo Carrara; Maurice Redondo; Mirjam Feldkamp; Natascha Santacroce; Niko Beerenwinkel; Olivier Kobel; Pelin Icer; Rebecca Denes; Sarah Nadeau; Sebastian Kurscheid; Shuqing Yu; Tanja Stadler; Tobias Schär
EPI_ISL_12651663, EPI_ISL_12709399, EPI_ISL_12780237, EPI_ISL_12895107, EPI_ISL_12895207	West of Scotland Specialist Virology Centre, NHSGGC / MRC-University of Glasgow Centre for Virus Research	COVID-19 Genomics UK (COG-UK) Consortium	Alasdair MacLean; Ana da Silva Filipe; Andy Young; Antonia Ho; Daniel Mair; David L Robertson; Emily Goldstein; Emma Thomson; Gonzalo Yebra; Guy Mollett; Ioulia Tsatsani; James Shepherd; Jenna Nichols; Jessica Benkaroun; Jon Perkins; Jordan Ashworth; Joseph Hughes; Kathy Smollett; Kirsty Mangin; Kyriaki Nomikou; Lily Tong; Matthew Holden; Nicolas Suarez; Rachael Tomb; Rachel Blacow; Richard Orton; Rory Gunson; Sarah McDonald; Sharif Shaaban; Sreenu Vattipally
EPI_ISL_12713156	Worcester Hospital wc WOC	NHLS/UCT	Arash Iranzadeh; Carolyn Williamson; Diana Hardie; Gert Marais; Innocent Mudau; Luicer Olubayo; Marvin Hsiao; Nokuzola Mbhele; Rageema Joseph; Stephen Korsman
EPI_ISL_12739598	Yale Clinical Virology Lab	Grubaugh Lab - Yale School of Public Health	Anne Hahn; Bony De Kumar; Chaney Kalinich; Chantal Vogels; Christopher Castaldi; David Ferguson; David Peaper; Irina Tikhonova; Kendall Billig; Kien Pham; Mallery Breban; Marie L. Landry; Nathan Grubaugh; Nicholas Chen; Nicholas Kerantzas; Rebecca Earnest; Tobias Koch; Wade Schulz
EPI_ISL_12580061	ZOTZ KLIMAS MVZ Düsseldorf-Centrum GbR ÜBAG für Labormedizin, Genetik, Zytologie, Pathologie	Center of Medical Microbiology, Virology, and Hospital Hygiene, University of Duesseldorf	Alexander Dilthey; Andreas Walker; Daniel Strelow; Jessica Nicolai; Jörg Timm; Katrin Hoffmann; Klaus Pfeffer; Lisanna Hülse; Malte Kohns Vasconcelos; Maximilian Damagnez; Nadine Lübke; Patrick Finzer; Rainer Zotz; Tobias Wienemann; Torsten Houwaart
