# Supplementary material for: Tracing the international arrivals of SARS-CoV-2 Omicron variants after Aotearoa New Zealand reopened its border
Source: Nat Commun. 2022 Oct 29;13:6484. doi: 10.1038/s41467-022-34186-9 (PMC9617600; doi:10.1038/s41467-022-34186-9)
Supplement: Supplementary file 8 — Supplementary Data 6 [file 41467_2022_34186_MOESM8_ESM.pdf]

We gratefully acknowledge the following Authors from the Originating laboratories responsible for obtaining the specimens, as well as the Submitting laboratories where the genome data were generated and shared via GISAID, on which this research is based.

All Submitters of data may be contacted directly via www.gisaid.org

Authors are sorted alphabetically.

Accession ID	Originating Laboratory	Submitting Laboratory	Authors
EPI_ISL_13227147	A.O. PERUGIA	A.O. PERUGIA	Bicchieraro G; Bondi P; Camilloni B; Cappelletti E; Ciurnelli R; Lepri E; Lucheroni F; Mencacci A; Spaccapelo R
EPI_ISL_13323342	A.S.L. TO3	Fondazione del Piemonte per l'Oncologia IRCCS	Antonino Sottile; Giorgio Giardina; Paola Marino; Silvia Brossa
EPI_ISL_13108779	ABC Algarve	Instituto Nacional de Saude Doutor Ricardo Jorge (INSA)	Borges et al
EPI_ISL_12763815	AMPATH	National Institute for Communicable Diseases of the National Health Laboratory Service	Amoako DG; Bhiman JN; Everatt J; Ismail A; Kekana D; Mahlangu B; Mnguni A; Mohale T; Ntuli N; Scheepers C; Wolter N
EPI_ISL_13322873	AOPD	Istituto Zooprofilattico Sperimentale delle Venezie	Adelaide Milani; Alessia Schivo; Alice Fusaro; Ambra Pastori; Angela Salomoni; Annalisa Salviato; Antonia Ricci; Calogero Terregino; Edoardo Giussani; Elisa Palumbo; Erika Giorgia Quaranta; Isabella Monne
EPI_ISL_13176072	AOUIVR	Unità Operativa Complessa di Microbiologia e Virologia Azienda Ospedaliera Universitaria Integrata di Verona; Istituto Zooprofilattico Sperimentale delle Venezie	Adelaide Milani; Alessia Schivo; Alice Fusaro; Angela Salomoni; Annalisa Salviato; Antonia Ricci; Calogero Terregino; Davide Gibellini; Edoardo Giussani; Elisa Palumbo; Erika Giorgia Quaranta; Giona Turri; Isabella Monne; Monica Castellucci; Nicoletta Medaina
EPI_ISL_13371594, EPI_ISL_13371618	ARION GENETICA	Instituto Nacional de Medicina Genomica	Cedro-Tanda A; Escobar-Arrazola MA; Garnica-Lopez Dora; Herrera-Montalvo LA.; Hidalgo-Miranda A; Mendoza-Vargas A; Ramirez-Vega O; Rangel-DeLeon D; Reyes-Grajeda JP; Roldan-Castillo Magaly; Uribe-Figueroa Laura; Verea Jazmin; Yair Alfaro-Mora
EPI_ISL_13019093, EPI_ISL_13019106	ARS Algarve - Laboratorio Laura Ayres	Instituto Nacional de Saude Doutor Ricardo Jorge (INSA)	Borges et al
EPI_ISL_12981999, EPI_ISL_13181342, EPI_ISL_13181797	Akershus University Hospital, Department for Microbiology and Infectious Disease Control	Norwegian Institute of Public Health, Department of Virology	Atiya R Ali; Debech Nadia; Engebretsen Serina Beate; Garcia Llorente Ignacio; Hilde Elshaug; Hilde Nordby Falkenhaug; Hilde Vollan; Jon Bråte; Kamilla Heddeland Instefjord; Karoline Bragstad; Kathrine Stene-Johansen; Line Victoria Moen; Marie Paulsen Madsen; Olav Hungnes; Pedersen Benedikte Nevjen; Rasmus Riis Kopperud
EPI_ISL_13202249	Algemeen Klinisch Labo	Labo Klinische Biologie, UZA	Basil Britto Xavier; Christine Lammens; Herman Goossens; Ines Verbesselt; Jasmine Coppens; Kathleen Holemans; Marie Le Mercier; Silke Liers; Veerle Matheeussen
EPI_ISL_12917780	Ampath	National Institute for Communicable Diseases of the National Health Laboratory Service	Amoako DG; Bhiman JN; Everatt J; Ismail A; Kekana D; Mahlangu B; Mnguni A; Mohale T; Ntuli N; Scheepers C; Wolter N
EPI_ISL_13337865	Area de salud barva (coopesiba)	Inciensa, Instituto Costarricense de Investigación y Enseñanza en Nutrición y Salud	Adriana Godínez; Claudio Soto-Garita; Estela Cordero; Francisco Duarte; Gabriel Morales; Hebleen Porras; José Luis Vargas; Mariela Gutiérrez; Melany Calderón; Natalia Bonilla & Sharon Peñaranda Chanto; Sofia Herrera
EPI_ISL_13337850	Area de salud pavas (coopesalud)	Inciensa, Instituto Costarricense de Investigación y Enseñanza en Nutrición y Salud	Adriana Godínez; Claudio Soto-Garita; Estela Cordero; Francisco Duarte; Gabriel Morales & Natalia Bonilla; Hebleen Porras; José Luis Vargas; Mariela Gutiérrez; Melany Calderón; Sofia Herrera
EPI_ISL_13340450	Austrian Agency for Health and Food Safety (AGES)	Bergthaler laboratory, CeMM Research Center for Molecular Medicine of the Austrian Academy of Sciences	Alberto Alises; Andreas Bergthaler; Anna Schedl; Christoph Bock; Fabian Amman; Lukas Endler; Matthew Thornton; Michael Schuster; Michelle Chan; Petr Triska
EPI_ISL_13134758	BP Healthcare Group	Institute for Medical Research, Infectious Disease Research Centre, National Institutes of Health, Ministry of Health Malaysia	Anasir MI; G.Adypatti NM; Jamaluddin MS; Kalyanasundram J; Kamel K; MatRahim N; Nawi MH; Suib FA; Suppiah J; Thayan R
EPI_ISL_13106453, EPI_ISL_13177148, EPI_ISL_13340742	Basurto University Hospital: Clinical Microbiology Laboratory	Basurto University Hospital: Clinical Microbiology Laboratory	Estibaliz Ugalde Zarraga; José Luis Díaz de Tuesta del Arco; Mikel Urrutikoetxea-Gutiérrez; Mª Carmen Nieto Toboso
EPI_ISL_13031192, EPI_ISL_13032045, EPI_ISL_13282427	BioneXt Lab	Laboratoire national de sante, Microbiology, Microbial Genomics Platform	Anke Wienecke-Baldacchino; Catherine Ragimbeau; Elodie Solarino; Eric Hugoson; Fatu Djabi; Jessica Tapp; Lise Pignon; Raoul Salmon; Sibel Berger; Tamir Abdelrahman; Thibault Ferrandon; Virginie Jover
EPI_ISL_13344815, EPI_ISL_13344818, EPI_ISL_13344826, EPI_ISL_13344838, EPI_ISL_13344839	Botswana Harvard AIDS Institute Partnership	Botswana Harvard HIV Reference Laboratory	Boitumelo Zuze; Botshelo Radibe; Dorcas Maruapula; Joseph Makhema; Keoratile Ntshambiwa; Kgomotso Moruisi; Legodile Kooepile; Mosepele Mosepele; Mphaphi B. Mbulawa; Ontlametse T. Bareng; Pamela Smith-Lawrence; Patrick T. Mokgethi; Roger Shapiro; Sefetogi Ramaologa; Shahin Lockman; Sikhulile Moyo; Simani Gaseitsiwe; Thongbotho Mphoyakgosi; Wonderful T. Choga
EPI_ISL_13158767, EPI_ISL_13159538, EPI_ISL_13159551, EPI_ISL_13276609, EPI_ISL_13276611, EPI_ISL_13276629, EPI_ISL_13276630			
see above	Bumrungrad International Hospital	National Institute of Health, Department of Medical Sciences, Ministry of Public Health, Thailand	Archawin Rojanawiwat; Natchaya Khiadsang; Nuttida Thongpramul; Pakorn Piromtong; Pilailuk Okada; Sirikanda Wimol; Siripaporn Phuygun; Sunthareeya Waicharoen; Suratchana Mitrat; Thanutsapa Thanadachakul
EPI_ISL_12954894, EPI_ISL_12954905	CENTRAL HEALTH LABORATORY	National Institute for Communicable Diseases of the National Health Laboratory Service	Amoako DG; Bhiman JN; Everatt J; Ismail A; Kekana D; Mahlangu B; Mnguni A; Mohale T; Ntuli N; Scheepers C; Wolter N
EPI_ISL_13253710	CERBALLIANCE OISE	CERBA HealthCare	Bénédicte Roquebert; Laura Verdurme; Mathilde Roussel; Sabine Trombert; Stéphanie Haim-Boukobza
EPI_ISL_12731318	CERBALLIANCE PORT	Laboratoire de virologie, CNR arbovirus Associé, Chu de la Réunion	Anne-Julie Gourdé; Etienne Frumence; Marie-Christine Jaffar Bandjee; Nicolas M'nemosyme; Nicolas Traversier; Rubens Lhonneur
EPI_ISL_13253757	CERBALLIANCE PYRENEES	CERBA HealthCare	Bénédicte Roquebert; Laura Verdurme; Mathilde Roussel; Sabine Trombert; Stéphanie Haim-Boukobza
EPI_ISL_13229725, EPI_ISL_13229728	CH Porto - H Sto Antonio	Instituto Nacional de Saude Doutor Ricardo Jorge (INSA)	Borges et al
EPI_ISL_12721258, EPI_ISL_13229798	CH Setubal	Instituto Nacional de Saude Doutor Ricardo Jorge (INSA)	Borges et al
EPI_ISL_12863086	CH Tamega e Sousa	Instituto Nacional de Saude Doutor Ricardo Jorge (INSA)	Borges et al
EPI_ISL_13229615	CH Tondela Viseu	Instituto Nacional de Saude Doutor Ricardo Jorge (INSA)	Borges et al
EPI_ISL_13053240	CHAZ Laboratory	Churches Health Association of Zambia (CHAZ) Laboratory	CHAZ Lab Staff; Chipango. C; Muyombo. A; Sandala. D; Shempela. D; Sikalima. J
EPI_ISL_13229591, EPI_ISL_13229600	CHTMAD	Instituto Nacional de Saude Doutor Ricardo Jorge (INSA)	Borges et al
EPI_ISL_13257781	Center for laboratory medicine, Clinical Center of Vojvodina	Institute of Molecular Genetics and Genetic Engineering University of Belgrade	Andriana Lazic; Anita Skakic; Bojan Ristivojevic; Ivana Moric; Jelena Stojčević Maletić; Katarina Novovic; Maja Tolinacki; Marija Cumbo; Milka Malesevic; Mina Peric; Mirjana Novkovic; Natasa Radakovic; Natasa Stevanovic; Sandra Vojnovic; Sofija Nesic; Stefan Stanovcic; Valentina Djordjevic
EPI_ISL_13341550, EPI_ISL_13360209, EPI_ISL_13360226	Central Health Laboratory , Victoria Hospital, Ministry of Health and Wellness, Mauritius	CERI, Centre for Epidemic Response and Innovation, Stellenbosch University and KRISP, KZN Research Innovation and Sequencing Platform, UKZN.	Anyaneji UJ; Bahadoor BS; Claassen M; Giandhari J; Issack M; Jannoo N; Maharaj A; Maponga T; Mathur H; Moir M; Naidoo Y; Pillay S; Preiser W; Ramuth M; San JE; Sanko TJ; Sankon TJ; Sonoo J; Stander T; Tegally H; Tshiabuila D; Ubheeram A; Van Wyk S; Wilkinson E; Wilson S; de Oliveira T; van Zyl G
EPI_ISL_13243355	Centrum Medyczne MEDYK Zaklad Diagnostyki Medycznej	Wojewodzka Stacja Sanitarno-Epidemiologiczna w Rzeszowie, Laboratorium Diagnostyki Medycznej	Anna Nowakowska; Karolina Ostrowska; Katarzyna Wilk; Marzena Baranowska
EPI_ISL_13337902	Clinica biblica	Inciensa, Instituto Costarricense de Investigación y Enseñanza en Nutrición y Salud	Adriana Godínez; Claudio Soto-Garita; Estela Cordero; Francisco Duarte; Gabriel Morales & Natalia Bonilla; Hebleen Porras; José Luis Vargas; Mariela Gutiérrez; Melany Calderón; Sofia Herrera
EPI_ISL_13323849, EPI_ISL_13323984	Clinical Microbiology Laboratory, Tel Aviv Sourasky Medical Center	Clinical Microbiology Laboratory, Tel Aviv Sourasky Medical Center	Alon Ziv; Amos Adler; Goel Morad; Katya Levytskyi; Lior Handler; Matan Slutzkin; Ora Halutz; Orly Eshel
EPI_ISL_13129404, EPI_ISL_13242132	Clinical Microbiology, Infection Prevention and Control	Section for Molecular Diagnostics	Björn Hallström; Jonas Björkman
EPI_ISL_12954164	Coronavirus Homecare	National Institute for Communicable Diseases of the National Health Laboratory Service	Amoako DG; Bhiman JN; Everatt J; Ismail A; Kekana D; Mahlangu B; Maphalala G; Mnguni A; Mohale T; Ntuli N; Scheepers C; Wolter N

EPI_ISL_12918515	D'Almeida Clinic wc DAL	NHLS/UCT	Arash Iranzadeh; Carolyn Williamson; Diana Hardie; Gert Marais; Innocent Mudau; Luicer Olubayo; Marvin Hsiao; Nokuzola Mbhele; Rageema Joseph; Stephen Korsman
EPI_ISL_12291987, EPI_ISL_12728612, EPI_ISL_12895296, EPI_ISL_13049308, EPI_ISL_13049625, EPI_ISL_13157845, EPI_ISL_13177782, EPI_ISL_13178663, EPI_ISL_13201568, EPI_ISL_13241448, EPI_ISL_13299517			
see above	Department of Bacteria, Parasites and Fungi, Statens Serum Institut, Copenhagen, Denmark	Statens Serum Institut Bioinformatics and Microbial Genomics	Danish Covid-19 Genome Consortium
EPI_ISL_13140517	Department of Health Technology and Informatics, The Hong Kong Polytechnic University	Department of Health Technology and Informatics, The Hong Kong Polytechnic University	Alan Ka-Lun Wu; Alex Yat-Man Ho; Barry Kin-Chung Wong; Chloe Toi-Mei Chan; David Ho-Keung Shum; Gilman Kit-Hang Siu; Hiu-Yin Lao; Ivan Tak-Fai Wong; Jake Siu-Lun Leung; Kam-Tong Yip; Kenneth Siu-Sing Leung; Kingsley King-Gee Tam; Kitty Sau-Chun Fung; Kristine Luk; Lam-Kwong Lee; Miranda Chong-Yee Yau; Sandy Ka-Yee Chau; Shea Ping Yip; Tak-Lun Que; Timothy Ting-Leung Ng; Wing Cheong Yam; Wing-Hei Lo; Wing-Kin To; Yvette Wai-Man Lai
EPI_ISL_13182857	Department of Medical Microbiology, Baerum Hospital, Vestre Viken Health Trust	Norwegian Institute of Public Health, Department of Virology	Atiya R Ali; Debech Nadia; Engebretsen Serina Beate; Garcia Llorente Ignacio; Hilde Elshaug; Hilde Nordby Falkenhaug; Hilde Vollan; Jon Bråte; Kamilla Heddeland Instefjord; Karoline Bragstad; Kathrine Stene-Johansen; Line Victoria Moen; Marie Paulsen Madsen; Olav Hungnes; Pedersen Benedikte Nevjen; Rasmus Riis Kopperud
EPI_ISL_13328006	Department of Medical Microbiology, St. Olavs hospital	Norwegian Institute of Public Health, Department of Virology	Atiya R Ali; Debech Nadia; Engebretsen Serina Beate; Garcia Llorente Ignacio; Hilde Elshaug; Hilde Nordby Falkenhaug; Hilde Vollan; Jon Bråte; Kamilla Heddeland Instefjord; Karoline Bragstad; Kathrine Stene-Johansen; Line Victoria Moen; Marie Paulsen Madsen; Olav Hungnes; Pedersen Benedikte Nevjen; Rasmus Riis Kopperud
EPI_ISL_13351801	Department of Microbiology and Infection Control, Akershus University Hospital HF	Department of Microbiology and Infection Control, Akershus University Hospital HF	Alexander Hesselberg Løvestad; Divya Murugananthan; Hanne Berggreen; Hege Vangstein Aamot
EPI_ISL_13109539, EPI_ISL_13109540, EPI_ISL_13302865	Department of Virology, National Institute of Health, Islamabad, Pakistan	Department of Virology, National Institute of Health, Islamabad, Pakistan	Aamer Ikram; Massab Umair; Muhammad Ammar; Muhammad Salman; Nazish Badar; Qasim Malik; Syed Adnan Haider; Zaira Rehman
EPI_ISL_12972952	Dianalabs SA	Genesupport	Geraldine Jost; Katia Jaton; Nadia Liassine; Tanguy ARAUD
EPI_ISL_13102657, EPI_ISL_13102658, EPI_ISL_13102683, EPI_ISL_13254037, EPI_ISL_13254040, EPI_ISL_13254058, EPI_ISL_13342160			
see above	Directorate of Public Health and Preventive Medicine	CDFD-INSACOG	Arunkumar Karunanidhi; Ashwin Dalal; Asmita Gupta; Avudaiselvi Rathinasamy; Darez Ahamed; Devi Monika Ayyagari Venkata; Divya Vashisht; Gurunathan Subramanian; Hemashree Kannan; Kalpana Raghu; Murali Bashyam; Rajesh Kumar Manivannan; Raju Sivadoss; Rupin Shelake; Sampath Palani; Selvavinayagam Sivaprakasam; Vinay Donipadi
EPI_ISL_13025341, EPI_ISL_13086516, EPI_ISL_13353282, EPI_ISL_13353404, EPI_ISL_13353627	Division of Emerging Infectious Diseases, Bureau of Infectious Diseases Diagnosis Control, Korea Disease Control and Prevention Agency	Division of Emerging Infectious Diseases, Bureau of Infectious Diseases Diagnosis Control, Korea Disease Control and Prevention Agency	Ae Kyung Park; Chae Young Lee; Eun-Jin Kim; Hyuck Jin Lee; Il-Hwan Kim; Jeong-Ah Kim
EPI_ISL_12703375, EPI_ISL_12903896, EPI_ISL_13209520	Dr. Mustafa, Dr. Richter Labor für medizinisch-chemische und mikrobiologische Diagnostik GmbH, Abteilung Molekularbiologie	Dr. Mustafa, Dr. Richter Labor für medizinisch-chemische und mikrobiologische Diagnostik GmbH, Abteilung Molekularbiologie	Alexander Gamisch; Maria Elisabeth Mustafa
EPI_ISL_13051401, EPI_ISL_13051430, EPI_ISL_13243915, EPI_ISL_13243973, EPI_ISL_13243977, EPI_ISL_13243990, EPI_ISL_13363222			
see above	Dr. Risch Ostschweiz AG	Dr Risch Laboratory	Dominique Fabien Hilti; Faina Wehrli; Lorenz Risch; Martin Risch; Nadia Wohlwend; Sinem Kas; Thomas Bodmer
EPI_ISL_12783845, EPI_ISL_13144618, EPI_ISL_13214669, EPI_ISL_13332292, EPI_ISL_13332293, EPI_ISL_13332294, EPI_ISL_13332295, EPI_ISL_13332296, EPI_ISL_13332297, EPI_ISL_13332299, EPI_ISL_13332300, EPI_ISL_13332355, EPI_ISL_13332375			
see above	Dutch COVID-19 response team	National Institute for Public Health and the Environment (RIVM)	Adam Meijer; Afke Vogelzang; AnneMarie van den Brandt; Annelies Kroneman; Bas van der Veer; Chantal Reusken; Dennis Schmitz; Dirk Eggink; Florian Zwagemaker; Harry Vennema; Ivo van Walle; Jeroen Cremer; Jil Kocken; Jordy de Bakker; Karim Hajji; Kim Freriks; Linda van Someren; Lisa Wijsman; Lynn Aarts; Ryanne Jaarsma; Sanne Bos; Sharon van den Brink; on behalf of the national COVID-19 response team
EPI_ISL_13160184	EORLA	Kingston Health Sciences Centre	Calvin Sjaarda; Drew Roberts; Henry Wong; Jacob Whalen; Nick Buchner; Phung Ta; Prameet Sheth; Sheri Levesque
EPI_ISL_13353762	Elizabeth Glaser Pediatric AIDS Foundation	KEMRI-Wellcome Trust Research Programme,Kilifi	Agoti C.; D.J.Nokes; Githinji G.; Lambisia A.; Makori T.; Mburu M.W.; Mohamed K.S.; Morobe J.; Ndwiga L.; Ochola I; Ongera E.; de Laurent Z.
EPI_ISL_13223992	Enfer	Enfer	Elaine M. Kenny; Suzie Coughlan
EPI_ISL_13294882	Eurofins-NMDL	Eurofins-NMDL	Anco Molijn; Anne Vogel; Lisa Dreesens; Marvin Ruiter; Maurine Leversteijn-van Hall; Roy Masius; Simon Lansu
EPI_ISL_13228364	Faculty Hospital Bulovka, Department of Clinical Microbiology	Charles University, Faculty of Science, BIOCEV, OMICS Genomics	Alžběta Bučková; Blanka Hamplová; Ingrid Poláková; Jiří Novák; Nela Václavíková; Ruth Tachezy; Sebastian Cristian Treitli; Vladimír Hampl; Zoltán Füssy; Štěpánka Hrdá
EPI_ISL_13163536	Fondation Congolaise pour la recherche medicale (FCRM), Francine Ntoumi	Fondation Congolaise pour la Recherche Médicale	Dr Batchi-Bouyou Armel Landry; Dr. Jean Claude Djontu; Mfoutou Mapanguy Claujens Chastel; Prof. Francine Ntoumi
EPI_ISL_13311036, EPI_ISL_13346730, EPI_ISL_13346731, EPI_ISL_13346748	Gandhi Medical College and Hospital (GMCH), Secunderabad	NIV Influenza	A.Rajender goud; Abdul Majeed; Amrithesh Kumar Arun; D.R.Manisha Rani; Devendhar; Dr.G.Sushma Rajya Lakshmi; Dr.K.Nagamani; Dr.Sunitha Pakalapaty; Hajeera Osmani; Sahithya
EPI_ISL_12982341, EPI_ISL_12982342, EPI_ISL_12982343, EPI_ISL_13154708, EPI_ISL_13154710, EPI_ISL_13154971, EPI_ISL_13155270, EPI_ISL_13155302, EPI_ISL_13155321, EPI_ISL_13164925, EPI_ISL_13164932, EPI_ISL_13164941, EPI_ISL_13164983, EPI_ISL_13164991, EPI_ISL_13165708, EPI_ISL_13165719			
see above	Genetica Molecular and Subdepartamento de Virologia ISP Chile	Instituto de Salud Publica de Chile	Andres Castillo; Barbara Parra; Constanza Campano; Ivan Ponce; Jorge Fernandez; Karen Orostica; Marcelo Rojas; Matias Pezoa; Patricia Bustos; Paulo covarrubias; Rodrigo Fasce
EPI_ISL_13229452, EPI_ISL_13368867	H Fernando Fonseca	Instituto Nacional de Saude Doutor Ricardo Jorge (INSA)	Borges et al
EPI_ISL_13142445, EPI_ISL_13142450, EPI_ISL_13206776	HB TERREO EMERG CONV QUARTOS	Instituto Butantan	; Alex Ranieri Lima; Antonio Jorge Martins; Claudia Renata dos Santos Barros; David Schlesinger; Debora Botequio Moretti; Dimas Tadeu Covas; Elaine Cristina Marqueze; Elaine Vieira Santos; Evandra Strazza Rodrigues; Gabriela Ribeiro; Heidge Fukumasu; Jayme Augusto de Souza-Neto; Luiz Alcantara; Luiz Lehmann Coutinho; Maria Carolina Elias; Maurício Lacerda Nogueira; Raul Machado Neto; Rejane Maria Tommasini Grotto; Ricardo Haddad; Sandra Coccuzzo Sampaio Vessoni; Simone Kashima; Svetoslav Nanev Slavov; Vincent Louis Viala
EPI_ISL_13150091	HOPITAL DE MONTLUCON	CHU Clermont-Ferrand, service de virologie	Bisseux Maxime; Combes Patricia; Henquell Cecile; Mirand Audrey
EPI_ISL_13069827	HOSPITAL DR. WILLIAM ALLEN	Inciensa, Instituto Costarricense de Investigación y Enseñanza en Nutrición y Salud	Adriana Godínez; Claudio Soto-Garita; Estela Cordero; Francisco Duarte; Gabriel Morales & Natalia Bonilla; Hebleen Porras; José Luis Vargas; Mariela Gutiérrez; Melany Calderón; Sofia Herrera
EPI_ISL_13114125	HOSPITAL UNIVERSITARIO CENTRAL DE ASTURIAS	Laboratorio de Virología HUCA	; Alba L; Alvarez-Arguelles ME; Boga JA; Costales I; Coto E; González-Alba JM; Gómez de Oña J; Martín-Rodríguez G; Melón S; Perez-Martínez Z; Rojo S; Sandoval M
EPI_ISL_12933029, EPI_ISL_13198799, EPI_ISL_13337436	Histopath	NSW Health Pathology - Institute of Clinical Pathology and Medical Research; Westmead Hospital; University of Sydney	Arnott A.; Draper J.; Gall M.; Martinez E.; Rockett R.; Sintchenko V.; on behalf of ICPMR
EPI_ISL_13369822, EPI_ISL_13369826, EPI_ISL_13369827, EPI_ISL_13369828, EPI_ISL_13369861	Home Quarantine Taskforce	Hong Kong Department of Health	Alan K.L. Tsang; Edman T.K. Lam; Ken H.L. Ng; Patricia K. L. Leung; Rickjason C.W. Chan
EPI_ISL_13283230, EPI_ISL_13283380, EPI_ISL_13283861, EPI_ISL_13372852	Hopital	National Reference Center for Viruses of Respiratory Infections, Institut Pasteur, Paris	Angela Brisebarre; Camille Capel; Christophe Malabat; Corinne Maufrais; Etienne Simon-Lorière; Frédéric Lemoine; Julien Fumey; L COURDAVAULT; Laura DJAMDJIAN; Laurence FAGOUR; Louise Lefrançois; Marion Barbet; Maud Vanpeene; Méline Bizard; Slim El Khiari; Sylvie Van der Werf; Vincent Enouf
EPI_ISL_13002450	Hospital Universitari Dr. Josep Trueta	Institut d'Investigació Biomèdica de Girona | Hospital Universitari Dr. Josep Trueta	Bernat del Olmo; Mel·lina Pinsach; Meritxell Deulofeu; Nuria Esther Neto; Paula Costa
EPI_ISL_13340685	Institute for Water Quality and Resource Management, Technical University Vienna	Bergthaler laboratory, CeMM Research Center for Molecular Medicine of the Austrian Academy of Sciences	Alberto Alises; Andreas Bergthaler; Anna Schedl; Christoph Bock; Fabian Amman; Lukas Endler; Matthew Thornton; Michael Schuster; Michelle Chan; Petr Triska
EPI_ISL_13368512, EPI_ISL_13368516, EPI_ISL_13368536	Institute of Microbiology and Immunology, Faculty of Medicine, University of Ljubljana	Institute of Microbiology and Immunology, Faculty of Medicine, University of Ljubljana	Alen Suljič; Andraž Celar; Doroteja Vlaj; Mario Poljak; Miša Korva; Patricija Pozvek; Samo Zakotnik; Tatjana Avšič – Županc; Tina Gabrovšek; Tina Živič; Tomaž Mark Zorec; Špela Pleh
EPI_ISL_13118911	Islab, Pohjois-Karjalan aluelaboratorio	Expert Microbiology, National Institute for Health and Welfare	Carita Savolainen-Kopra; Erika Lindh; Haider al-Hello; Jani Halkilahti; Kirsi Liitsola; Niina Ikonen; Niko Tervo; Olli Vapalahti; Pekka Ellonen; Phuoc Truong; Päivi Laurila; Ravi Kant; Sari Hannula; Soile Blomqvist; Teemu Smura
EPI_ISL_13203753, EPI_ISL_13203755, EPI_ISL_13203845, EPI_ISL_13203852, EPI_ISL_13203877, EPI_ISL_13203887	Islab, Pohjois-Savon aluelaboratorio	Expert Microbiology, National Institute for Health and Welfare	Carita Savolainen-Kopra; Erika Lindh; Haider al-Hello; Jani Halkilahti; Kirsi Liitsola; Niina Ikonen; Niko Tervo; Olli Vapalahti; Pekka Ellonen; Phuoc Truong; Päivi Laurila; Ravi Kant; Sari Hannula; Soile Blomqvist; Teemu Smura

EPI_ISL_13353748	KEMRI-Wellcome Trust Research Programme,Kilifi	KEMRI-Wellcome Trust Research Programme,Kilifi	Agoti C.; D.J.Nokes; Githinji G.; Lambisia A.; Makori T.; Mburu M.W.; Mohamed K.S.; Morobe J.; Ndwiga L.; Ochola I; Ongera E.; de Laurent Z.
EPI_ISL_13334887	Kaiser Permanente Southern California	Helix	Helix; Kaiser Permanente Southern California
EPI_ISL_13371724, EPI_ISL_13371772, EPI_ISL_13371783	Karolinska University Hospital Huddinge	Karolinska University Hospital	Annika Tiveljung Lindell; Henning Onsbring; Jan Albert; Karina Hentrich; Lynda Eneh; Maria Ropat; Martin Ekman; Natalija Gerasimcik; Robert Dyrdak; Sandra Broddesson; Shambhu Ganeshappa Aralaguppe; Tanja Normark; Tobias Allander; Valtteri Wirta; Zhibing Yun
EPI_ISL_12864060, EPI_ISL_13131104, EPI_ISL_13313980, EPI_ISL_13314006	Karolinska University Hospital Solna	Karolinska University Hospital	Annika Tiveljung Lindell; Henning Onsbring; Jan Albert; Karina Hentrich; Lynda Eneh; Maria Ropat; Martin Ekman; Natalija Gerasimcik; Robert Dyrdak; Sandra Broddesson; Shambhu Ganeshappa Aralaguppe; Tanja Normark; Tobias Allander; Valtteri Wirta; Zhibing Yun
EPI_ISL_13064563	Kuala Lumpur International Airport	Institute for Medical Research, Infectious Disease Research Centre, National Institutes of Health, Ministry of Health Malaysia	Anasir MI; G.Adypatti NM; Jamaluddin MS; Kalyanasundram J; Kamel K; MatRahim N; Nawi MH; Suib FA; Suppiah J; Thayan R
EPI_ISL_13342510	LABORATOIRE CREAVALLEE	CNR Virus des Infections Respiratoires - France SUD	Antonin Bal; Bruno Lina; Bruno Simon; Gregory Destras; Gwendolyne Burfin; Hadrien Regue; Laurence Josset; Martine Valette; Quentin Semanas; Theophile Boyer
EPI_ISL_13176333	LABORATOIRE d'ANALYSES de BIOLOGIE MEDICALES	CNR Virus des Infections Respiratoires - France SUD	Antonin Bal; Bruno Lina; Bruno Simon; Gregory Destras; Gwendolyne Burfin; Hadrien Regue; Laurence Josset; Martine Valette; Quentin Semanas; Theophile Boyer
EPI_ISL_12767816	LATE - Laboratório de Técnicas Especiais - Hospital Israelita Albert Einstein	LATE - Laboratório de Técnicas Especiais - Hospital Israelita Albert Einstein	Alexandre Hideaki Takara; Ana Paula Moreira Salles; Anelisie da Silva Santos; Deyvid Amgarten; Erick Gustavo Dorlass; Fernanda de Mello Malta; João Renato Rebello Pinho; Luiz Vicente Rizzo; Marcio Anunciacao Menezes; Pedro Henrique Sebe Rodrigues; Raquel Riyuzo
EPI_ISL_13183074	Lab voor klinische biologie	Lab voor klinische biologie	Bruno Verhasselt; Hannelore Hamerlinck; May-Linh Truong
EPI_ISL_13363991, EPI_ISL_13364015	Labkesda DKI	National Quality Control Laboratory of Drug and Food	Hana Apsari Pawestri; M. Erdiansyah; Nurul Azizah; Sri Utaminingsih
EPI_ISL_13351624	Labkesda Kabupaten Batang	National Institute of Health Research and Development	Arie Ardiansyah Nugraha; Fajar Nur Sulistiyohadi; Hana Apsari Pawestri; Hartanti Dian Ikawati; IGM Wirabrata; Kartika Dewi Puspa; Nelis Imaningsih; Nur Ika Hariastuti; Putri Widia Astuti; Subangkit
EPI_ISL_13091520, EPI_ISL_13283371, EPI_ISL_13362924, EPI_ISL_13372706, EPI_ISL_13372838	Labo Analyses Med	National Reference Center for Viruses of Respiratory Infections, Institut Pasteur, Paris	Angela Brisebarre; Camille Capel; Christophe Malabat; Corinne Maufrais; Etienne Simon-Lorière; Frédéric Lemoine; J.M CORCY; Julien Fumey; Karine MICHEZ; Louise Lefrançois; Marie-Hélène GLAUDON LOUVEAU DE LA GUIGNERAYE; Marion Barbet; Maud Vanpeene; Méline Bizard; Slim El Khiari; Sylvie Van der Werf; Vincent Enouf
EPI_ISL_13241273	Labor Lübeck bzw. Laborärztliche Gemeinschaftspraxis Lübeck	Robert Koch Institute	
EPI_ISL_13343334	Labor team w AG	Department of Biosystems Science and Engineering, ETH Zürich	Andreas Grutsch; Andreas Lindauer; Chaoran Chen; Christian Beisel; David Dreifuss; Elodie Burcklen; Franziska Singer; Ina Nissen; Ivan Topolsky; Kim Philipp Jablonski; Lara Fuhrmann; Louis du Plessis; Matteo Carrara; Mirjam Feldkamp; Monika Bucher; Natascha Santacroce; Niko Beerenwinkel; Pelin Burcak Icer; Rebecca Denes; Rebekka Pohl; Sarah Nadeau; Shuqing Yu; Tanja Stadler; Tobias Schär
EPI_ISL_13140449, EPI_ISL_13140452	Laboratoire Rodolphe Mérieux (INRB Goma)	Pathogen Genomics Lab, National Institute for Biomedical Research (INRB)	Allison Black; Amuri Aziza; Andrew Rambaut; Catherine Pratt; Daniel Mukadi; Eddy Kinganda-Lusamaki; Edith Nkwembe; Emmanuel Lokilo Lofiko; Francisca Muyembe Mawete; Gradi Luakanda; Hervé Viala; Ian Goodfellow; James Hadfield; Jean Claude Makangara; Jean-Jacques Muyembe Tamfum; Josh Quick; Kristian Andersen; Matthias Pauthner; Michael Wiley; Michel Mbimbi; Nick Loman; Placide Mbala-Kingebeni; Steve Ahuka-Mundeke; Trevor Bedford
EPI_ISL_13303541, EPI_ISL_13303746, EPI_ISL_13304033	Laboratoire de santé publique du Québec	Laboratoire de santé publique du Québec	Guillaume Bourque; Ioannis Ragoussis; Jesse Shapiro; Mark Lathrop and Judith Fafard on behalf of the CoVSeQ research group; Sandrine Moreira
EPI_ISL_13282483	Laboratoire national de sante, Microbiology, Virology	Laboratoire national de sante, Microbiology, Microbial Genomics Platform	Anke Wienecke-Baldacchino; Catherine Ragimbeau; Elodie Solarino; Eric Hugoson; Fatu Djabi; Jessica Tapp; Lise Pignon; Raoul Salmon; Sibel Berger; Tamir Abdelrahman; Trung Nguyen Nguyen; Virginie Jover
EPI_ISL_13292140	Laboratoires Reunis	Laboratoire national de sante, Microbiology, Microbial Genomics Platform	Anke Wienecke-Baldacchino; Bernard Weber; Catherine Ragimbeau; Elodie Solarino; Eric Hugoson; Fatu Djabi; Jessica Tapp; Lise Pignon; Raoul Salmon; Sibel Berger; Tamir Abdelrahman; Virginie Jover
EPI_ISL_13292035, EPI_ISL_13292343, EPI_ISL_13292366	Laboratoires d'analyses medicales - KETTERTHILL	Laboratoire national de sante, Microbiology, Microbial Genomics Platform	Anke Wienecke-Baldacchino; Caroline Scheiber; Catherine Ragimbeau; Elodie Solarino; Eric Hugoson; Fatu Djabi; Jessica Tapp; Lise Pignon; Raoul Salmon; Serge Vedy; Sibel Berger; Tamir Abdelrahman; Virginie Jover
EPI_ISL_12838778	Laboratorio Central de Saude Publica do Estado do Rio de Janeiro (LACEN/RJ)	Laboratory of Respiratory Viruses and Measles, Oswaldo Cruz Institute, FIOCRUZ	Alice Sampaio Rocha; Andrea Cony Cavalcanti; Bruna Mendonça da Silva; Elisa Cavalcante Pereira; Fernando Motta; Ighor Arantes; Jéssica Graça Macedo de Carvalho; Larissa Macedo Pinto; Luciana Appolinario; Marilda Siqueira on behalf of the Fiocruz COVID-19 Genomic Surveillance Network; Paola Resende; Victor Guimaraes
EPI_ISL_13142047, EPI_ISL_13314030, EPI_ISL_13314041, EPI_ISL_13314213	Laboratorio de Referencia Nacional de Virus Respiratorios. Centro Nacional de Salud Publica. Instituto Nacional de Salud Peru.	Laboratorio de Referencia Nacional de Virus Respiratorios. Centro Nacional de Salud Publica. Instituto Nacional de Salud Peru.	Alicia Nuñez Llanos; Carlos Padilla Rojas; Estela Huaman Angeles; Francisco Ascue Orosco; Gloria Arotinco Garayar.; Henri Bailon Calderon; Iris Silva Molina; Jorge Giraldo Chavez; Joseph Huayra Niquen; Karla Vasquez Cajachahua; Kelly Izarra Rojas; Lely Solari Zerpa; Lilian Huarca Balbin; Lisbet Roxana Inga Angulo; Luis Barcena Flores; Luren Sevilla Castañeda; Marco Galarza Perez; Maria Villar Saavedra; Nancy Rojas Serrano; Omar Caceres Rey; Orson Mestanza Millones; Princesa Medrano Alhuay; Priscila Lope Pari; Steve Acedo Lazo; Veronica Hurtado Vela; Victor Jimenez Vasquez; Wendy Lizarraga Olivares
EPI_ISL_12889805, EPI_ISL_13152605	Laboratory Corporation of America	Centers for Disease Control and Prevention Division of Viral Diseases, Pathogen Discovery	Amanda Douglas; Amanda Suchanek; Andrea Throop; Ayla Burns; Benjamin Rambo-Martin; Bobbi Croy; Brian Krueger; Brian Norvell; Christopher Gulvick; Christos Petropoulos; Clinton Paden; Craig Lukasik; Dakota Howard; Debbie Boles; Dhwani Batra; Duncan MacCannell; Eyad Almasri; Goran Stevovic; Howard Engler; Hrushikesh Deshmukh; Jake Humphrey; Jana Schroth; Jason Caravas; Joe Voshell; John Pruitt; Jonathan Meltzer; Jonathan Williams; Kimberly Wagner; Kristine Lacek; Lax Iyer; Lisa Pfefferle; Lyndon Tilson; Manoj Jain; Marcia Eisenberg; Mary Cristobal; Mary Williamson; Matthew Robinson; Matthew Schmerer; Michael Levandoski; Mike Sapeta; Mindy Nye; Minoo Agarwal; Mohan Kolli; Nuthawin Charoensri; Oren Cohen; Peter Cook; Prashant Gupta; Qian Zeng; Rama Ghatti; Scott Parker; Scott Ryan; Scott Sammons; Shatavia Morrison; Stanley Letovsky; Steven Ragan; Suresh Selvaraju; Susan Countryman; Susan Hicks; Suzanne Dale; Thomas Urban; Tim Kuphal; Tricia Zwiefelhofer; Tymeckia Kendall; Victoria Caban Figueroa; Vincent Drouillon; Yvette Unoarumhi
EPI_ISL_13013557, EPI_ISL_13229149, EPI_ISL_13229156, EPI_ISL_13229163, EPI_ISL_13229172	Laboratory of Genomics and Bioinformatics, Comenius University Science Park	Laboratory of Genomics and Bioinformatics, Comenius University Science Park	Anna Kaliňáková; Barbora Kotvasová; Diana Rusňáková; Jakub Styk; Jaroslav Budiš; Lucia Ševčíková; Michaela Jakubková Forgáčová; Miroslav Böhmer; Nikola Lipková; Pavol Mišenko; Silvia Bokorová; Tatiana Sedláčková; Terézia Vrabľová; Tomáš Szemes
EPI_ISL_12561210, EPI_ISL_12561211, EPI_ISL_12561212, EPI_ISL_12561213	Landspítali, Department of Clinical Microbiology	Landspítali, Department of Clinical Microbiology	Arsalan Amirfallah; Freyja Valsdóttir; Zarko Urosevic
EPI_ISL_12875191, EPI_ISL_12875272, EPI_ISL_13249983, EPI_ISL_13250051	Lifebrain Covid Labor GmbH	Lifebrain Covid Labor GmbH	Alexandra Wagner; Anna Edermayr; Filip Sima; Kristina Bavrka Kolenc; Lucia Castello
EPI_ISL_13157142, EPI_ISL_13185833, EPI_ISL_13231687, EPI_ISL_13311807	Lighthouse Lab in Glasgow	Wellcome Sanger Institute for the COVID-19 Genomics UK (COG-UK) Consortium	Anna Dominiczak and Alex Alderton; Carol Clugston; Cordelia Langford; David Gray; David K. Jackson; Dominic Kwiatkowski; Ewan Harrison; Harper VanSteenhouse; Ian Johnston; Jeffrey Barrett; John Sillitoe on behalf of the Wellcome Sanger Institute COVID-19 Surveillance Team; Roberto Amato; Sonia Goncalves; Yumi Kasai
EPI_ISL_13033928	Limbach - MVZ Labor Ravensburg Labor Dr. Gärtner	Robert Koch Institute	
EPI_ISL_13137139, EPI_ISL_13355257	MANILA DOCTORS HOSPITAL	Research Institute for Tropical Medicine	Alexander Sadiasa; Amalea Dulcene D. Nicolasora; Angela Kae T. Chang; Anne Pauline A. Alpino; Ardiane Ysabelle M. Dolor; Catalino S. Demetria; Charalyn Babida; Claudette Lee S. Navarro; Criselda T. Bautista; Dodge R. Lim; Francisco Gerardo M. Polotan; Gerald Ivan S. Sotelo; Jefferson Earl J. Halog; Joseph Hughes; Joy Mariette L. Parayray; Katie Hampson; Kirstyn Brunker; Lei Lanna M. Dancel; Ma. Angelica A. Tujan; Ma. Ricci R. Gomez; Mayan Uy-Lumandas; Othoniel Jan T. Onza; Simon Daldry; Timothy John R. Dizon; Vina Lea F. Arguelles; Yao-Tsun Li
EPI_ISL_13247839	MDI Limbach Berlin GmbH; MVZ Labor Berlin	Robert Koch Institute	
EPI_ISL_13332769, EPI_ISL_13332770, EPI_ISL_13332771, EPI_ISL_13332773, EPI_ISL_13332774, EPI_ISL_13332775	MRC/UVRI & LSHTM Uganda Research Unit	MRC/UVRI & LSHTM Uganda Research Unit	Bernard Mpairwe; Joseph Mugisha; Matthew Cotten; My V.T. Phan; Robert Newton
EPI_ISL_13269831	MVZ Dr. Eberhard & Partner Dortmund	Robert Koch Institute	
EPI_ISL_13268448	MVZ Labor Krone GbR	Robert Koch Institute	
EPI_ISL_13359967	Mater Pathology, South Brisbane—Mater Hospital Brisbane	Public Health Virology - Forensic and Scientific Services (PHV-FSS)	Chenwei Wang on behalf of Q-PHIRE Genomics
EPI_ISL_12954406	Mbabane Public Health Unit	National Institute for Communicable Diseases of	Amoako DG; Bhiman JN; Everatt J; Ismail A; Kekana D; Mahlangu B; Maphalala G; Mnguni A; Mohale T; Ntuli N; Scheepers C; Wolter N

		the National Health Laboratory Service	
EPI_ISL_12954176	Mbabane city council	National Institute for Communicable Diseases of the National Health Laboratory Service	Amoako DG; Bhiman JN; Everatt J; Ismail A; Kekana D; Mahlangu B; Maphalala G; Mnguni A; Mohale T; Ntuli N; Scheepers C; Wolter N
EPI_ISL_13238615	Medizinische Laboratorien Düsseldorf	Robert Koch Institute	
EPI_ISL_12915494	Microbiology Department, Laboratori Clínic Metropolitana Nord. Hospital Universitari Germans Trias i Pujol	Can Ruti SARS-CoV-2 Sequencing Hub (HUGTiP/IrsiCaixa/IGTP)	Alexia París; Ana Blanco; Andreu Coello; Antoni E Bordoy; Bonaventura Clotet; David Panisello; Francesc Catala-Moll; Gemma Clara; Ignacio Blanco; Laia Soler; Marc Noguera-Julian; Montserrat Giménez; Pere-Joan Cardona; Pilar Armengol; Roger Paredes; Sara González; Verónica Saludes; and Elisa Martró on behalf of the Can Ruti SARS-CoV-2 Sequencing Hub
EPI_ISL_13229987, EPI_ISL_13230291, EPI_ISL_13312308, EPI_ISL_13312310	Microbiology Department. Complexo Hospitalario Universitario de Vigo	Microbiology Department. Complexo Hospitalario Universitario de Vigo	Cabrera JJ; Cortizo S; Daviña C; Gonzalez-Dominguez M; Martinez L; Pena I; Perez-Castro S; Potel C; Rey S; Vasallo FJ; del-Campo V
EPI_ISL_13113071, EPI_ISL_13113222, EPI_ISL_13176029	Ministry of Health Turkey	Ministry of Health Turkey	Arzu İrvem; Cemal Kazezoğlu; Feride Velaei; Gülay Korukluoğlu; Gültekin Ünal; Meral Kaya; Rabia Can Sarınoğlu; Serap Demir Tekol; Şemsinur Karabela
EPI_ISL_12972929, EPI_ISL_12972930, EPI_ISL_12972931, EPI_ISL_12972932, EPI_ISL_12972933	Molecular Biology and Virology	Molecular Biology and Virology	Dr.Moh''D Borhan Al-Zghoul; Dr.Mustafa Ababneh; Mohammad Alboom
EPI_ISL_13244309	Molecular Genetic Monitoring Group	Molecular Genetic Monitoring Group	Anna S. Gladkikh; Areg A.Totolian; Ekaterina O. Klyuchnikova; Valerya A. Sbarzaglia; Vladimir G. Dedkov
EPI_ISL_13259127, EPI_ISL_13259128	NIH	National Institute of Hygiene	Abderrahman Bimouhen; Fatima El Falaki; Hassan Ihazmade; Hicham Oumzil; Zakia Regragui
EPI_ISL_12837921	Nastavni zavod za javno zdravstvo Splitsko- Dalmatinske županije	Hrvatski zavod za javno zdravstvo	Anita Jurić; Dragan Jurić; Irena Tabain; Ivana Ferenčak; Josipa Kuzle
EPI_ISL_12845590	National Health Laboratory Services	CERI, Centre for Epidemic Response and Innovation, Stellenbosch University and KRISP, KZN Research Innovation and Sequencing Platform, UKZN.	Anyaneji UJ; Giandhari J; Maharaj A; Moir M; Naidoo Y; Nokukhanya Mdlalose; Pillay S; San JE; Sanko TJ; Tegally H; Tshiabuila D; Van Wyk S; Wilkinson E; de Oliveira T
EPI_ISL_11763529, EPI_ISL_12097409	National Health Laboratory Services	CERI, Centre for Epidemic Response and Innvoation, Stellenbosch University and KRISP, KZN Research Innovation and Sequencing Platform, UKZN.	Anyaneji UJ; Giandhari J; Maharaj A; Mdlalose N; Moir M; Naicker D; Naidoo Y; Nokukhanya Mdlalose; Pillay S; San JE; Tegally H; Tshiabuila D; Van Wyk S; Wilkinson E; de Oliveira T
EPI_ISL_12474479	National Health Laboratory Services, Virology	National Health Laboratory Services, Virology	Ashlyn S. C. Davis; Florette K. Treurnicht; Kathleen Subramoney; Nkhensani Mtileni
EPI_ISL_13345427	National Laboratory for Health, Environment and Food, OMM, Maribor	NLZOH (National Laboratory for Health, Environment and Food)	Aleksander Mahnic; Alenka Štorman; Andrej Golle; Kaja Tominc; Leon Marič; Maja Rupnik; Maša Jarčič; Mojca Cimerman; Nika Gobec; Nika Volmajer; Sabina Mlakar; Sandra Janezic; Tanja Vrabič; Tjaša Žohar Čretnik; Urška Dobovišek
EPI_ISL_13102212	National Platform bis COVID ULB-IBC	National Platform bis COVID ULB-IBC	Arnaud Marchant; Coralie Henin; Lionel Schiavolin; Marie-Luce Delforge; Mathilde Le Garrec
EPI_ISL_12835609	National Platform bis UMONS/Jolimont	National Platform bis UMONS/Jolimont	Caroline Debecker; Clothilde Claus; Eric Tarantino; Florian Juszczak; Gautier Detry; Laetitia Gheysen; Ruddy Wattiez
EPI_ISL_13094168, EPI_ISL_13150735, EPI_ISL_13150742, EPI_ISL_13259913	National Public Health Laboratory, National Centre for Infectious Diseases	National Public Health Laboratory, National Centre for Infectious Diseases	BeiBei Chen; Benny Yeo; Chen Shi Ling; Grace Ngan; Jesslin Tan; Lin Cui; Raymond Tzer Pin Lin; Royce Ang; Samuel Loo; Yichen Ding; Zhenyang Zhou
EPI_ISL_13186247, EPI_ISL_13186619, EPI_ISL_13186641, EPI_ISL_13252794, EPI_ISL_13252801, EPI_ISL_13298834	National Virus Reference Laboratory	National Virus Reference Laboratory	Charlene Bennett; Cillian F De Gascun; Gabriel Gonzalez; Jonathan Dean; Michael Carr; Zoe Yandle
EPI_ISL_12954169	Nhlangano Health Centre	National Institute for Communicable Diseases of the National Health Laboratory Service	Amoako DG; Bhiman JN; Everatt J; Ismail A; Kekana D; Mahlangu B; Maphalala G; Mnguni A; Mohale T; Ntuli N; Scheepers C; Wolter N
EPI_ISL_13066528, EPI_ISL_13298415, EPI_ISL_13326086, EPI_ISL_13326108, EPI_ISL_13362168	Originating lab: Wales Specialist Virology Centre Sequencing lab: Pathogen Genomics Unit	Public Health Wales Microbiology Cardiff Wales Specialist Virology Centre	Alec Birchley; Alexander Adams; Amy Gaskin; Angela Marchbank; Bree Gatica-Wilcox; Catherine Moore; Jason Coombes; Joanne Watkins; Joel Southgate; Johnathan Evans; Laura Gifford; Lauren Gilbert; Lee Graham; Malorie Perry; Matthew Bull; Nicole Pacchiarini; Sally Corden; Sara Kumziene-Summerhayes; Sara Rey; Sarah Taylor; Simon Cottrell; Sophie Jones; Tom Connor
EPI_ISL_12851724, EPI_ISL_13140805, EPI_ISL_13140809, EPI_ISL_13331766, EPI_ISL_13331779	Outre Mer	Institut Pasteur	Angela Brisebarre; Camille Capel; Christophe Malabat; Corinne Maufrais; Etienne Simon-Lorière; Frédéric Lemoine; Julien Fumey; Louise Lefrançois; Marie-Hélène GLAUDON LOUVEAU DE LA GUIGNERAYE; Marion Barbet; Maud Vanpeene; Méline Bizard; Slim El Khiari; Sylvie Behillil; Sylvie Van der Werf; Vincent Enouf
EPI_ISL_13324310	Outre Mer	National Reference Center for Viruses of Respiratory Infections, Institut Pasteur, Paris	Angela Brisebarre; Camille Capel; Christophe Malabat; Corinne Maufrais; Etienne Simon-Lorière; Frédéric Lemoine; Julien Fumey; Laurence FAGOUR; Louise Lefrançois; Marion Barbet; Maud Vanpeene; Méline Bizard; Slim El Khiari; Sylvie Van der Werf; Vincent Enouf
EPI_ISL_13137131	PASIG CITY CHILDREN'S HOSPITAL	Research Institute for Tropical Medicine	Alexander Sadiasa; Amalea Dulcene D. Nicolasora; Angela Kae T. Chang; Anne Pauline A. Alpino; Ardiane Ysabelle M. Dolor; Catalino S. Demetria; Charalyn Babida; Claudette Lee S. Navarro; Criselda T. Bautista; Dodge R. Lim; Francisco Gerardo M. Polotan; Gerald Ivan S. Sotelo; Jefferson Earl J. Halog; Joseph Hughes; Joy Mariette L. Parayray; Katie Hampson; Kirstyn Brunker; Lei Lanna M. Dancel; Ma. Angelica A. Tujan; Ma. Ricci R. Gomez; Mayan Uy-Lumandas; Othoniel Jan T. Onza; Simon Daldry; Timothy John R. Dizon; Vina Lea F. Arguelles; Yao-Tsun Li
EPI_ISL_13355613	PCR Laboratory, Divisional Head Quarters Teaching Hospital, Mirpur, AJ&K	Department of Virology, National Institute of Health, Islamabad, Pakistan	Aamer Ikram; Massab Umair; Muhammad Ammar; Muhammad Salman; Nazish Badar; Qasim Ali and Najma Majeed; Syed Adnan Haider; Zaira Rehman
EPI_ISL_13369867	PRENETICS LIMITED	Hong Kong Department of Health	Alan K.L. Tsang; Edman T.K. Lam; Ken H.L. Ng; Patricia K. L. Leung; Rickjason C.W. Chan
EPI_ISL_13198072	PSSE Lowicz	1. Academic Center for Pathomorphological and Genetic-Molecular Diagnostics ltd, Bialystok, Poland 2. National Institute of Public Health - National Institute of Hygiene, Warsaw, Poland	Anetta Sulewska; Jacek Niklinski; Janusz DzięcioL; Joanna Kiśluk; Katarzyna Zacharczuk; Konrad Raczkowski; MaLgorzata Sadkowska-Todys; Magdalena Nowakowska; Piotr Karabowicz; Piotr Majewski; PrzemysLaw Biecek. Joanna Reszeć; RadosLaw Charkiewicz; Tomasz WoLkowicz
EPI_ISL_13371984	Pardubicka nemocnice	University Hospital Hradec Kralove	Helena Parova; Lenka Rysava; Marketa Gancarcikova; Monika Berankova
EPI_ISL_12871865	Pathcare	CERI, Centre for Epidemic Response and Innovation, Stellenbosch University and KRISP, KZN Research Innovation and Sequencing Platform, UKZN.	Anyaneji UJ; Claassen M; Giandhari J; Maharaj A; Maponga T; Moir M; Naidoo Y; Pillay S; Preiser W; San JE; Sanko TJ; Stander T; Tegally H; Tshiabuila D; Van Wyk S; Wilkinson E; Wilson S; de Oliveira T; van Zyl G
EPI_ISL_13231360	Pathologist Lancet Kenya	KEMRI-Wellcome Trust Research Programme,Kilifi	Agoti C.; D.J.Nokes; Githinji G.; Lambisia A.; Makori T.; Mburu M.W.; Mohamed K.S.; Morobe J.; Mukadam R; Munoko A.; Ndwiga L.; Ngari C.; Ochola I; Ongera E.; de Laurent Z.
EPI_ISL_13107189	Pathology Queensland and Forensic Scientific Services	Public Health Virology - Forensic and Scientific Services (PHV-FSS)	Son Nguyen on behalf of Q-PHIRE Genomics
EPI_ISL_13359899	Plateforme de testing Namuroise	Plateforme de testing Namuroise	Degosserie Jonathan; Denis Olivier; Drugmand Jonathan; Janssens Louise; Laurent Hélène; Maschietto Céline; Mullier François; Otto Gaetan; Renguet Edith
EPI_ISL_13251640	Platform BIS UZA/UAntwerpen	Labo Klinische Biologie, UZA	Basil Britto Xavier; Christine Lammens; Herman Goossens; Ines Verbesselt; Jasmine Coppens; Kathleen Holemans; Marie Le Mercier; Silke Liers; Veerle Matheeussen
EPI_ISL_13177706	Public Health Authority of the Slovak Republic	Laboratory of Genomics and Bioinformatics, Comenius University Science Park	Anna Kaliňáková; Barbora Kotvasová; Diana Rusňáková; Elena Tichá; Jaroslav Budiš; Lucia Ševčíková; Miroslav Böhmer; Pavol Mišenko; Terézia Vrabľová; Tomáš Szemes
EPI_ISL_13149262, EPI_ISL_13149283, EPI_ISL_13149290, EPI_ISL_13149292, EPI_ISL_13149299, EPI_ISL_13149302, EPI_ISL_13149306, EPI_ISL_13149324			
see above	Public Health Laboratory: COVID-19 Lab	International Livestock Research Institute	Collins Muli; Daniel Ouso; Edward Kiritu; Edward O. Abworo; Gilbert Kibet; Gugu Maphalala; Mncedisi Hlophe; Nomcebo Phungwayo; Patrick Amoth; Paul Dobi; Samuel O. Oyola; Shebbar Osiany; Siphesihle Langwenya; Sonal P. Henson; Susan Kamalizeni; Vishvanath Nene

EPI_ISL_13053559, EPI_ISL_13053622, EPI_ISL_13135210, EPI_ISL_13270696, EPI_ISL_13270939	Public Health Ontario Laboratory	Public Health Ontario Laboratory	Aimin Li; Alex Marchand-Austin; Andre Villegas; Anna Puzinovici; Ashleigh Sullivan; Brandon Ye; Candice Schreiber; Carla Duncan; Christina Rampertab; Christine Seah; Claudia Chu; Dean Maxwell; Dhiraj Gaglani; Doonia Bajovic; Esther Nagai; Fatemeh Shaeri; Fatima Merza; Grace Jeong; Hadia Hussain; Himeshi Samarsinghe; Jacob Afelskie; Jason Iraheta; Jesse Wang; John Palmer; Karthikeyan Sivaraman; Kirby Cronin; Lisa Kim; Lisa McTaggart; Maria Mariscal; Mark Horsman; Narisha Shakuralli; Nataliya Potapova; Natasha Sing; Nobish Varghese; Philip Banh; Rachelle DiTullio; Rebecca Azzaro; Rima Palencia; Samir N Patel; Sarah Teatero; Semra Tibebu; Sophie Yu; Surendra Kumar; Sushma Kavikondala; Vincent Su Bin Cha; Zarah Rajaei
EPI_ISL_13249923, EPI_ISL_13249972	Puskesmas Pasirkaliki	West Java Health Laboratory; School of Life Sciences and Technology, Institut Teknologi Bandung	Azzania Fibriani; Cut Nur Cinthia Alamanda; Ema Rahmawati; Hadiana; Karimatu Khoirunnisa; Miftahul Faridl; Rifky Waluyajati Rachman; Rini Robiani; Ryan Bayusantika Ristandi
EPI_ISL_13261955, EPI_ISL_13291443, EPI_ISL_13316310	Quest Diagnostics Incorporated	Centers for Disease Control and Prevention Division of Viral Diseases, Pathogen Discovery	A. Gerasimova; A. Perez; B. Anderson; Benjamin Rambo-Martin; Christopher Gulvick; Clinton Paden; Dakota Howard; Dhwani Batra; Duncan MacCannell; Erisa Sula; F. Lacbawan; I. Shlyakhter; Jason Caravas; K. Livingston; Kristine Lacek; L. Bernstein; M. Hua; Matthew Schmerer; P. Tanpaiboon; Peter Cook; R. Kagan; R. Owen; R. Rolando; S. Rosenthal; Scott Sammons; Shatavia Morrison; Tymeckia Kendall; Victoria Caban Figueroa; Y. Liu; Yvette Unoarumhi
EPI_ISL_12704285, EPI_ISL_12704369, EPI_ISL_12810659, EPI_ISL_12810724, EPI_ISL_13157528	Regional Virus Laboratory, Belfast Health and Social Care Trust; and: Genomics Core Technology Unit, Queen's University Belfast.	COVID-19 Genomics UK (COG-UK) Consortium	Alan; Alison Watt; Arun Mahesh; BHSCT]: Conall McCaughey; Ciara Cox; Clara Radulescu; David Simpson; Deborah Lavin; Derek Fairley; Evan Troendle; Fiona Rogan; James McKenna; Jana Gazdova; Julia Miskelly; Mairead Connor; Miao Tang; QUB]: Marc Fuchs; Rice; Sarah Sonner; Stephen Bridgett; Susan Feeney; Syed Umbreen; Tanya Curran; Timofey Skvortsov; Zoltan Molnar; [Genomics Core Technology Unit; [Regional Virus Laboratory
EPI_ISL_13298307	Respiratory Virus Unit, Microbiology Services Colindale, Public Health England	COVID-19 Genomics UK (COG-UK) Consortium	PHE Covid Sequencing Team
EPI_ISL_12838667, EPI_ISL_12920374, EPI_ISL_13106591, EPI_ISL_13231623, EPI_ISL_13242371, EPI_ISL_13311352, EPI_ISL_13311641, EPI_ISL_13311704, EPI_ISL_13338284			
see above	Rosalind Franklin Laboratory	Wellcome Sanger Institute for the COVID-19 Genomics UK (COG-UK) Consortium	Cordelia Langford; David K. Jackson; Dominic Kwiatkowski; Donald Fraser; Ewan Harrison; Ian Johnston; Jeffrey Barrett; John Sillitoe on behalf of the Wellcome Sanger Institute COVID-19 Surveillance Team; Rob Howes; Roberto Amato; Sonia Goncalves; Suki Lee; The Rosalind Franklin Laboratory and Alex Alderton
EPI_ISL_13249967	Rumah Sakit TK II Dustira	West Java Health Laboratory; School of Life Sciences and Technology, Institut Teknologi Bandung	Azzania Fibriani; Cut Nur Cinthia Alamanda; Ema Rahmawati; Hadiana; Karimatu Khoirunnisa; Miftahul Faridl; Rifky Waluyajati Rachman; Rini Robiani; Ryan Bayusantika Ristandi
EPI_ISL_13262714, EPI_ISL_13371523, EPI_ISL_13371628, EPI_ISL_13371646	SALUD DIGNA	Instituto Nacional de Medicina Genomica	Abraham Campos-Romero; Cedro-Tanda A; Escobar-Arrazola MA; Garcia-Garcia FE; Garnica-Lopez Dora; Herrera-Montalvo LA.; Hidalgo-Miranda A; Luna-Ruiz Marco; Mendoza-Vargas A; Moreno-Camacho José Luis; Ramirez-Vega O; Rangel-DeLeon D; Reyes-Grajeda JP; Rodriguez-Gallegos Jorge; Sanchez-Xochipa S; Yair Alfaro-Mora
EPI_ISL_13292855, EPI_ISL_13301003	SARS-CoV-2 Sequencing Castilla y Leon-Spain Consortium	SARS-CoV-2 Sequencing Castilla y Leon-Spain Consortium	Antonio Orduña-Domingo; Carlos Fuster Foz; Carmen Aldea-Mansilla; Carmen Gimeno Crespo; David Abad; Gabriel March Rosello; Gregoria Megías Lobón; Jose María Eiros Bouza; M. Isabel Fernandez-Natal; Marta Dominguez-Gil; Marta Hernandez; María Antonia García Castro; Mª Fe Brezmes-Valdivieso; Noelia Arenal Andrés; Silvia Rojo; Sonsoles Garcinuño Pérez
EPI_ISL_13027465, EPI_ISL_13167988, EPI_ISL_13217382, EPI_ISL_13217556, EPI_ISL_13217726, EPI_ISL_13217784	SARS-CoV-2 testing team, National Institute of Infectious Diseases	Pathogen Genomics Center, National Institute of Infectious Diseases	Hazuka Y Furihata; Kentaro Itokawa; Makoto Kuroda; Masanori Hashino; Masumichi Saito; Naomi Nojiri; Nozomu Hanaoka; Rina Tanaka; Tsuguto Fujimoto; Tsuyoshi Sekizuka
EPI_ISL_13372121	SC (UCO) Igiene e Sanità Pubblica, ASUGI, Trieste	SC (UCO) Igiene e Sanità Pubblica, ASUGI, Trieste	Basaglia G; Busetti M; D'Agaro P; Fontana F; Forciniti G; Koncan R; Pipan C; Piscianz E; Segat L
EPI_ISL_12863427, EPI_ISL_13369107	SESARAM	Instituto Nacional de Saude Doutor Ricardo Jorge (INSA)	Borges et al
EPI_ISL_12903160, EPI_ISL_13076019	SYNLAB	University Hospital Brno, CMBG	Bezdicek Matej; Dolejska Monika; Kristyna Dufkova; Lengerova Martina; Svaton Jan
EPI_ISL_12190609, EPI_ISL_13143504	SYNLAB MVZ Weiden	Robert Koch Institute	
EPI_ISL_13155840	Servicio Virosis Respiratorias-Departamento Virología-INEI	Instituto Nacional Enfermedades Infecciosas C.G.Malbran	Avaro M.; Baumeister E.; Benedetti E.; Campos J.; Cisterna D.; Dattero ME; De Belder D.; Haim MS.; Mallou F.; Molina V.; Perandones C.; Poklepovich T.; Pontoriero A.; Russo M.; Sanchez Loria J.; Tuduri E.
EPI_ISL_13250911	Servicio de Microbiologia Hospital Ramon y Cajal	Servicio de Microbiologia Hospital Ramon y Cajal	Galan JC; Martinez-García L.; Ponce-Alonso M
EPI_ISL_13302920, EPI_ISL_13303076, EPI_ISL_13313392, EPI_ISL_13313420, EPI_ISL_13313628, EPI_ISL_13316622, EPI_ISL_13316807			
see above	Shamir Medical Center (Asaf Harofe)	Shamir Medical Center (Asaf Harofe)	Abu Hamad Ramzia; Adina Bar Chaim; Anna Vishnevsky; Chen Weiner; Nir Rainy; Patricia Benveniste-Lekovitz; Reut Sorek Abramovich; Yevgeni Yegorov
EPI_ISL_13208025	Sirindhorn Hospital	Medical Genomic Centre,Medical Life Sciences Institute,Department of Medical Sciences, Ministry of Public Health, Thailand	Archawin Rojanawiwat; Jirapha Pakdee; Naphatcha Thawong; Natthakul Bunneang; Nuanjun Wichukchinda; Pilailuk Akkapaiboon Okada; Pundharika Piboonsiri; Surakameth Mahasirimongkol; Waritta Sawaengdee
EPI_ISL_13339041	Stadtspital Triemli	Department of Biosystems Science and Engineering, ETH Zürich	Alexandra Trkola; Chaoran Chen; Christian Beisel; David Dreifuss; Elodie Burcklen; Franziska Singer; Guido Bloemberg; Ina Nissen; Ivan Topolsky; Kevin Steiner; Kim Philipp Jablonski; Lara Fuhrmann; Louis du Plessis; Maryam Zaheri; Matteo Carrara; Michael Huber; Mirjam Feldkamp; Natascha Santacroce; Niko Beerenwinkel; Pelin Burcak Icer; Rebecca Denes; Riccarda Capaul; Sarah Nadeau; Shuqing Yu; Stefan Schmutz; Tanja Stadler; Tobias Schär; Verena Kufner
EPI_ISL_13273973	SuperCare Medical Services, Inc	Research Institute for Tropical Medicine	Alexander Sadiasa; Amalea Dulcene D. Nicolasora; Angela Kae T. Chang; Anne Pauline A. Alpino; Ardiane Ysabelle M. Dolor; Catalino S. Demetria; Charalyn Babida; Claudette Lee S. Navarro; Criselda T. Bautista; Dodge R. Lim; Francisco Gerardo M. Polotan; Gerald Ivan S. Sotelo; Jefferson Earl J. Halog; Joseph Hughes; Joy Mariette L. Parayray; Katie Hampson; Kirstyn Brunker; Lei Lanna M. Dancel; Ma. Angelica A. Tujan; Ma. Ricci R. Gomez; Mayan Uy-Lumandas; Othoniel Jan T. Onza; Simon Daldry; Timothy John R. Dizon; Vina Lea F. Arguelles; Yao-Tsun Li
EPI_ISL_13184257	Swedish national genomic surveillance program of SARS-CoV-2	The Public Health Agency of Sweden	Alma Brolund; Emmi Andersson; Maria Lind Karlberg; Swedish national genomic surveillance program of SARS-CoV-2
EPI_ISL_13313928	Switch Health	National Microbiology Laboratory (NML)	Adrian Zetner; Anna Majer; Anneliese Landgraff; CanCOGeN's metadata curation team; Carmen Lia Murall; Chanchal Yadav; Connor Chato; Darian Hole; Elsie Grudeski; Emily Haidl; Gary Van Domselaar; Gordon Jolly; Grace Seo; Jeff Tuff; Jennifer Tanner; Katherine Eaton; Kirsten Biggar; Kristyn Burak; Madison Chapel; Morag Graham; Natalie Knox; Nathalie Bastien; Philip Mabon; Public Health Agency of Canada's CCGP and Scientific Informatics Services team; Rhiannon Huzarewich; Russell Mandes; Shari Tyson; Timothy Booth; Yan Li
EPI_ISL_13029236, EPI_ISL_13029256, EPI_ISL_13066210, EPI_ISL_13133837, EPI_ISL_13133857, EPI_ISL_13133875, EPI_ISL_13133904			
see above	Synlab Eesti OÜ	1. Laboratory of Communicable Diseases (Estonia); 2. Eurofins Genomics Europe Sequencing GmbH	Abroi A.; Avi R.; Dotsenko L.; Epštein J.; Hoidmets D.; Huik K.; Härma M-A.; Jaaniso E.; Kaarna K.; Kallas E.; Koppel I.; Kuzmin I.; Lahesaare A.; Lutsar I.; Metspalu M.; Milani L.; Naaber P.; Niglas H.; Oopkaup O.E.; Pauskar M.; Peterson H.; Päll T.; Ratnik K.; Raudvere U.; Reisberg T.; Sadikova O.; Sepp H.; Shablinskaja A.; Suija H.; Talas U.G.; Truusalu K.
EPI_ISL_13369821	Temporary Specimen Collection Centre at the AsiaWorld-Expo	Hong Kong Department of Health	Alan K.L. Tsang; Edman T.K. Lam; Ken H.L. Ng; Patricia K. L. Leung; Rickjason C.W. Chan
EPI_ISL_12954411	The Luke Commission Hospital	National Institute for Communicable Diseases of the National Health Laboratory Service	Amoako DG; Bhiman JN; Everatt J; Ismail A; Kekana D; Mahlangu B; Maphalala G; Mnguni A; Mohale T; Ntuli N; Scheepers C; Wolter N
EPI_ISL_13156680	Tokyo Metropolitan Institute of Public Health	Tokyo Metropolitan Institute of Public Health	Ai Suzuki; Akane Negishi; Arisa Amano; Fumi Kasuya; Hirofumi Miyake; Kenji Sadamasu; Kenshiro Kuroki; Mami Nagashima; Maya Isogai; Ryota Kumagai; Sachiko Harada; Takushi Fujiwara
EPI_ISL_13369104	ULS Castelo Branco	Instituto Nacional de Saude Doutor Ricardo Jorge (INSA)	Borges et al
EPI_ISL_13345811, EPI_ISL_13345825, EPI_ISL_13345829, EPI_ISL_13345841	Unilabs Eskilstuna Laboratorium	Unilabs Eskilstuna Laboratorium	Emma Arvidsson
EPI_ISL_13372013, EPI_ISL_13372032, EPI_ISL_13372036	University Hospital Hradec Kralove	University Hospital Hradec Kralove	Helena Parova; Lenka Rysava; Marketa Gancarcikova; Monika Berankova
EPI_ISL_13202480, EPI_ISL_13202485	University Hospital Ostrava	University Hospital Ostrava	Chorzempa; Matějová; Špulerová
EPI_ISL_13202591	University Hospitals of Geneva, Laboratory of Virology	HUG, Laboratory of Virology and the Health2030 Genome Center	Aline Mamin; Ana Rita Goncalves; Cedric Howald; Deborah Penet; Francisco Perez; Henri Pegeot; Ioannis Xenarios; Keith Harshman; Laurent Kaiser; Lorenzo Cerutti; Melyssa Elies; Samuel Cordey
EPI_ISL_13286168	Utah Public Health Laboratory	Utah Public Health Laboratory	Erin L. Young; John Arnn; Kelly F. Oakeson; Olinto Linares-Perdomo; Pooja Gupta; Tom Iverson
EPI_ISL_13343324, EPI_ISL_13343396, EPI_ISL_13343462	Viollier AG	Department of Biosystems Science and Engineering, ETH Zürich	Andrea Patrizia Salzmann; Chaoran Chen; Christian Beisel; Christiane Beckmann; Christoph Noppen; David Dreifuss; Elodie Burcklen; Franziska Singer; Henriette Kurth; Ina Nissen; Ivan Topolsky; Kim Philipp Jablonski; Lara Fuhrmann; Louis du Plessis; Matteo Carrara; Maurice Redondo; Mirjam Feldkamp; Natascha Santacroce; Niko Beerenwinkel; Olivier Kobel; Pelin Burcak Icer; Rebecca Denes; Sarah Nadeau; Sebastian Kurscheid; Shuqing Yu; Tanja Stadler; Tobias Schär
EPI_ISL_13228472	ZNPHL	CHAZ Laboratory	CHAZ Lab Staff; Chipango. C; Muyombo. A; Sandala. D; Shempela. D; Sikalima. J
