# Supplementary material for: Tracing the international arrivals of SARS-CoV-2 Omicron variants after Aotearoa New Zealand reopened its border
Source: Nat Commun. 2022 Oct 29;13:6484. doi: 10.1038/s41467-022-34186-9 (PMC9617600; doi:10.1038/s41467-022-34186-9)
Supplement: Supplementary file 9 — Reporting Summary [file 41467_2022_34186_MOESM9_ESM.pdf]

nature portfolio

Corresponding author(s): Jordan Douglas

Last updated by author(s): Oct 12, 2022

Reporting Summary

Nature Portfolio wishes to improve the reproducibility of the work that we publish. This form provides structure for consistency and transparency in reporting. For further information on Nature Portfolio policies, see our Editorial Policies and the Editorial Policy Checklist.

Statistics

For all statistical analyses, confirm that the following items are present in the figure legend, table legend, main text, or Methods section.

n/a	Confirmed	
☐	☒	The exact sample size (*n*) for each experimental group/condition, given as a discrete number and unit of measurement
☐	☒	A statement on whether measurements were taken from distinct samples or whether the same sample was measured repeatedly
☒	☐	The statistical test(s) used AND whether they are one- or two-sided<br>*Only common tests should be described solely by name; describe more complex techniques in the Methods section.*
☐	☒	A description of all covariates tested
☐	☒	A description of any assumptions or corrections, such as tests of normality and adjustment for multiple comparisons
☐	☒	A full description of the statistical parameters including central tendency (e.g. means) or other basic estimates (e.g. regression coefficient) AND variation (e.g. standard deviation) or associated estimates of uncertainty (e.g. confidence intervals)
☒	☐	For null hypothesis testing, the test statistic (e.g. *F*, *t*, *r*) with confidence intervals, effect sizes, degrees of freedom and *P* value noted<br>*Give P values as exact values whenever suitable.*
☐	☒	For Bayesian analysis, information on the choice of priors and Markov chain Monte Carlo settings
☐	☒	For hierarchical and complex designs, identification of the appropriate level for tests and full reporting of outcomes
☒	☐	Estimates of effect sizes (e.g. Cohen's *d*, Pearson's *r*), indicating how they were calculated

Our web collection on statistics for biologists contains articles on many of the points above.

Software and code

Policy information about availability of computer code

Data collection	No software was used for data collection.
Data analysis	We used open source software: BEAST v2.6.7, PANGO v4.0.6, NextAlign v.2.3.0, and the latest version ARTIC bioinformatics pipeline (https://github.com/artic-network/artic-ncov2019; accessed 30 Jun 2022). We used the BICEPS v1.0.1, ORC v.1.0.3, and BEASTLabs v1.9.7 packages for BEAST 2.

For manuscripts utilizing custom algorithms or software that are central to the research but not yet described in published literature, software must be made available to editors and reviewers. We strongly encourage code deposition in a community repository (e.g. GitHub). See the Nature Portfolio guidelines for submitting code & software for further information.

Data

Policy information about availability of data

All manuscripts must include a data availability statement. This statement should provide the following information, where applicable:

- Accession codes, unique identifiers, or web links for publicly available datasets
- A description of any restrictions on data availability
- For clinical datasets or third party data, please ensure that the statement adheres to our policy

Note to editor: we have submitted our new New Zealand based genomes to GenBank and are still awaiting confirmation from NCBI. In the interest of time, we would appreciate if the remainder of this submission can be screened during the interim. The GenBank accession codes will be provided when available.

Formal statement: All global SARS-CoV-2 genomic sequence data are available on GISAID. Case, death, hospitalisation, and vaccination data used in the Introduction were taken from a New Zealand Ministry of Health GitHub repository (https://github.com/minhealthnz/nz-covid-data; accessed 30 Jun 2022). New Zealand passenger arrival data were taken from the Statistics New Zealand International travel provisional records (https://www.stats.govt.nz/indicators/international-travel-provisional; accessed 30 Jun 2022).

Human research participants

Policy information about studies involving human research participants and Sex and Gender in Research.

Reporting on sex and gender	Genomes were sampled from positive nasopharyngeal samples from PCR positive cases. The sex and gender of cases were not considered during genome sequencing.
Population characteristics	Genomes were sampled from positive nasopharyangeal samples from PCR positive cases. The age, genotypical background, and medical histories of cases were not considered during genome sequencing. However, we tried to achieve balanced sampling across the 20 district health boards of New Zealand, as presented in Figure S1.
Recruitment	Participants were not recruited. Genomes were sampled from positive nasopharyngeal samples from PCR positive cases.
Ethics oversight	Nasopharyngeal samples that had positive results for SARS-CoV-2 by real-time reverse transcription PCR were obtained from medical diagnostic laboratories located throughout New Zealand. Under contract for the New Zealand Ministry of Health, the Institute of Environmental Science and Research has approval to conduct genomic sequencing and phylogenetic analysis for surveillance of notifiable diseases.

Note that full information on the approval of the study protocol must also be provided in the manuscript.

Field-specific reporting

Please select the one below that is the best fit for your research. If you are not sure, read the appropriate sections before making your selection.

☒ Life sciences ☐ Behavioural & social sciences ☐ Ecological, evolutionary & environmental sciences

For a reference copy of the document with all sections, see nature.com/documents/nr-reporting-summary-flat.pdf

Life sciences study design

All studies must disclose on these points even when the disclosure is negative.

Sample size	The 10,403 genomes were sequenced from over 1 million positive COVID-19 cases. This sample size was not selected but rather it reflects the high quality genome sequencing capacity over the time period (~500 genomes per week). Genomic samples were largely random, however cases with recent overseas travel and those under hospital care were oversampled (as described in main article). We also tried to sample uniformly across district health boards (Figure S1).
Data exclusions	Nucleotide positions that were less than 20x coverage were masked to N in the final consensus genome. Positions with an alternative allele frequency between 20-79% were also masked. Genomes that contains more than 10% of Ns did not pass quality control and were excluded from the analysis. The exclusion criteria was pre-established.
Replication	We ran 3 MCMC chains under for each of the 5 Omicron subvariants and ensured that each chain converged to the same distribution (within a variant). Thus, our attempts at replication were successful.
Randomization	Our analysis included 1,056 genomes used in the phylogenetic analysis. These genomes are from both the New Zealand samples reported here as well as globally from GISAID. The sample of 1,056 genomes was random, and sampled uniformly across locations (global sample) and uniformly through time (New Zealand sample). Genomes were classified into Omicron subvariants using PANGO and one sample was taken per lineage.
Blinding	Blinding was not relevant to this study since data included de-identified genomes and is therefore not applicable.

Reporting for specific materials, systems and methods

We require information from authors about some types of materials, experimental systems and methods used in many studies. Here, indicate whether each material, system or method listed is relevant to your study. If you are not sure if a list item applies to your research, read the appropriate section before selecting a response.

Materials & experimental systems

n/a	Involved in the study
☒	☐ Antibodies
☒	☐ Eukaryotic cell lines
☒	☐ Palaeontology and archaeology
☒	☐ Animals and other organisms
☒	☐ Clinical data
☒	☐ Dual use research of concern

Methods

n/a	Involved in the study
☒	☐ ChIP-seq
☒	☐ Flow cytometry
☒	☐ MRI-based neuroimaging
